# Synthesis, Microbiology, and Biophysical Characterization of Mutanofactins from the Human Oral Microbiome

**DOI:** 10.1021/acscentsci.4c02184

**Published:** 2025-03-27

**Authors:** Lukas Lüthy, Leon Gabor Sacha Thies, Konstantin Nikolaus Beitl, Moritz Hansen, Joshua McManus, Muhammad Afzal, Lukas Schrangl, Susanne Bloch, Guruprakash Subbiahdoss, Erik Reimhult, Christina Schäffer, Erick M. Carreira

**Affiliations:** † Department of Chemistry and Applied Biosciences, Laboratory of Organic Chemistry, ETH Zürich, 8093 Zürich, Switzerland; ‡ Institute of Biochemistry, NanoGlycobiology Research Group, BOKU University, 1190 Vienna, Austria; § Institute of Colloid and Biointerface Science, BOKU University, 1190 Vienna, Austria; ∥ Institute of Biophysics, BOKU University, 1190 Vienna, Austria

## Abstract

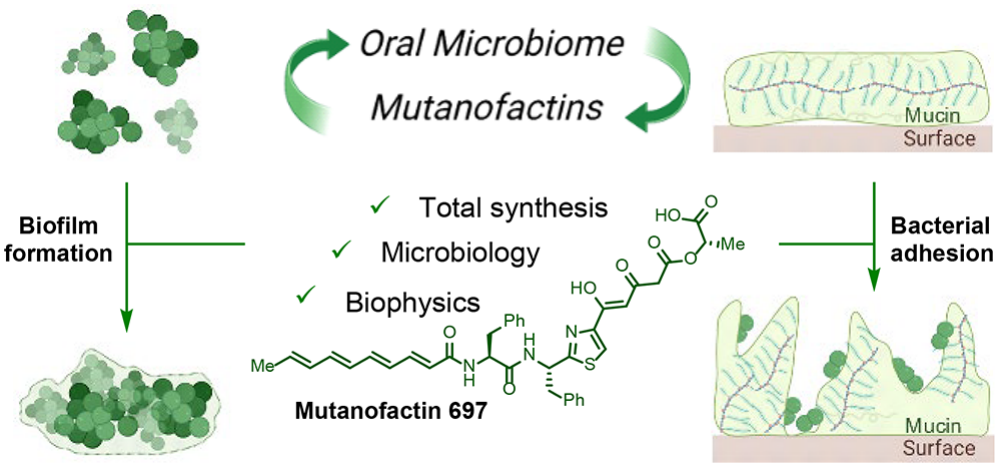

Mutanofactins are a family of natural products produced by *Streptococcus mutans* from the human oral microbiome. We report a unified approach to all mutanofactins by developing a total synthesis amenable to diversification. The key to success for the most complex members, mutanofactins 607 and 697, was an acyl ketene based strategy. Access to the family enabled comprehensive biological profiling, where we demonstrate that all mutanofactins are biofilm promoting in *Streptococcus mutans*. Experiments were extended to other inhabitants of the oral microbiome for the first time: *Streptococcus gordonii* and *Streptococcus oralis*, two early colonizers, were similarly affected with mutanofactins being biofilm promoting. Conversely, *Veillonella dispar* and *Fusobacterium nucleatum* showed little to no reaction to mutanofactins. Biophysical investigations based on quartz crystal microbalance with dissipation monitoring and atomic force microscopy reveal a previously unknown mucin–mutanofactin 697 interaction. Incubation of a mucin layer with mutanofactin 697 induces a morphology change within the mucin layer, which promotes bacterial adhesion and biofilm formation. This unique property of mutanofactin 697 might be key to early stages of biofilm formation in the human oral microbiome. Combined, an interdisciplinary approach consisting of total synthesis, microbiology and biophysical characterization provides insight into the roles of mutanofactins in the oral microbiome.

## Abstract 2

Mutanofactins are metabolites from the human oral microbiome. Our study includes synthesis, microbiology and biophysics. Key results are effects on mucin and biofilms of commensal bacteria.

## Introduction

**Figure 1:**
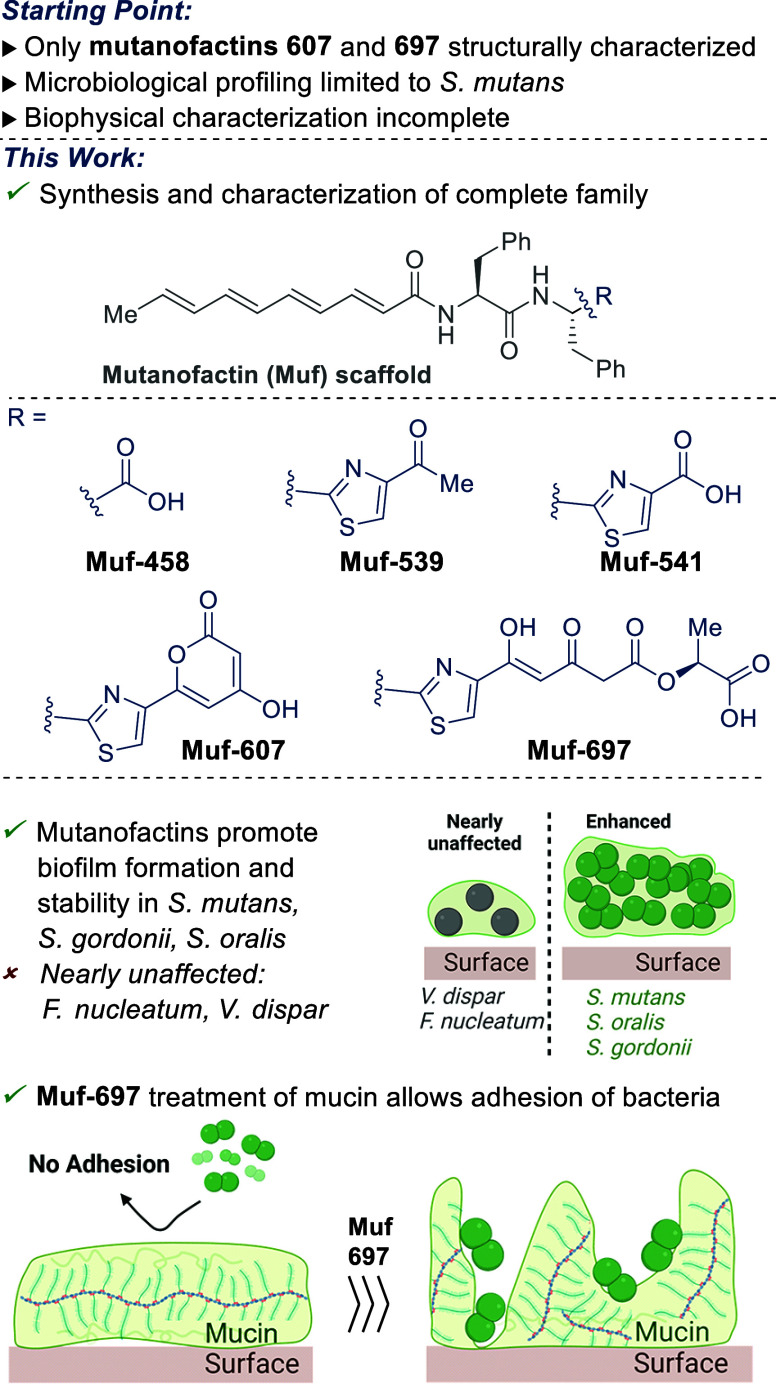
Prior work and our findings for mutanofactins.

The human oral microbiome is a diverse microcosm, estimated to be populated by more than 700 distinct species.^1^ It constitutes a rich source of bioactive secondary metabolites,^2,3^ with relevance to human health.^4,5^*Streptococcus mutans* is a member of the human oral microbiome and plays a key role in the establishment of dysbiotic oral biofilms. It is thereby closely linked to the development of dental caries, one of the most prevalent global chronic diseases.^6,7^ Despite its relatively small genome, *S. mutans* has proven a rich source of bioactive secondary metabolites.^8−10^ Recently, the mutanofactins, a new family of natural products, were discovered from *S. mutans* NMT4863 using a mass spectrometry based metabolomics approach (Figure 1).^11^ In that study, it was found that the high biofilm forming capabilities of some *S. mutans* clinical isolates are associated with the presence of a biosynthetic gene cluster encoding five amphiphilic lipopeptides: Mutanofactins 458, 539, 541, 607, and 697. Importantly, structural characterization of these compounds is limited, as NMR data was reported for only two out of five mutanofactins, and then of impure samples isolated from bacterial culture supernatants. The remaining mutanofactins were only assigned a putative structure based on a comparative analysis of HRMS/MS fragmentation. Li et al. concluded that mutanofactin 697 (Muf-697) in *S. mutans* is involved in biofilm formation.^11^ The inability to isolate the remaining mutanofactins precluded their study.

Our groups have a longstanding interest in human oral microbiome derived natural products, their chemistry,^12^ microbiology,^13^ as well as structural and physical properties of biofilms.^14,15^ The challenges of accessing pure compounds in the isolation work, as well as the ambiguous and lacking structural characterization for the family, compelled us to study the mutanofactins. The ability to synthesize these in pure form would enable full characterization and biophysical as well as microbiological investigations, especially in the context of other members of the human oral microbiome, coexisting with *S. mutans*. Such studies are paramount to shed light on these secondary metabolites in biofilm formation and, more broadly, on the role of *S. mutans* in its ecological niche.

Herein, first we report the total synthesis of all members of the natural product family, namely mutanofactins 458, 539, 541, 607, and 697. Having access to all reported mutanofactins, we carried out a number of experiments to elucidate their role within the human oral microbiome. We explore their impact on *S. mutans* with respect to bacterial growth, biofilm formation and integrity. Additionally, for the first time we explore the impact of mutanofactins on other cohabiting oral bacteria: *Streptococcus oralis*, *Streptococcus gordonii*, *Veillonella dispar*, and *Fusobacterium nucleatum*. Our studies led to some key findings: (1) All mutanofactins promote biofilm formation and stabilization in *S. mutans*. (2) Mutanofactins showed species-specific effects on coinhabitants of the human oral microbiome. The early colonizers *S. gordonii* and *S. oralis*, were similarly affected as *S. mutans*, whereas *F. nucleatum* and *V. dispar* showed little to no effect to mutanofactin exposure. (3) Biophysical investigations based on quartz crystal microbalance with dissipation monitoring (QCM-D) and atomic force microscopy (AFM) revealed a previously unknown interaction of mucin with Muf-697. We propose that the association of Muf-697 with a mucin layer renders its morphology more heterogeneous and thereby allows *S. mutans* to adhere onto this otherwise protective layer.

## Background

The human oral cavity harbors a complex and diverse ecosystem collectively known as the oral microbiome. It consists of bacterial, archaeal, fungal, and viral species.^16^ The interactions among the members of the oral microbiome and the host are crucial to maintain oral and, more broadly, systemic health.^1^ One of the defining features of the human oral microbiome is the prevalence of biofilms,^16,17^ highly structured communities of microorganisms encased in extracellular polymeric substances.^18^ Biofilms play a crucial role in oral microbial ecology, serving as the primary mode of microbial colonization on oral surfaces such as enamel, gums, tongue, and mucosal tissues.^19^ Biofilm formation is a dynamic and multistep process consisting of initial adhesion, microcolony formation, maturation, and eventual dispersal.^17^ Of importance for biofilm formation are mucins, heavily glycosylated proteins that are a major component of mucus.^20^ Mucins have been implicated in the modulation of bacterial composition^21^ and heavily influence the adhesion of bacteria to surfaces.^22^

The relevance of biofilm formation in the mouth extends beyond oral health, as disruptions in the balance of the oral microbiome and dysregulated biofilm formation have been implicated in the pathogenesis of various diseases. These include common oral conditions such as caries^7^ and periodontal diseases^23^ as well as systemic conditions,^24^ for example cardiovascular diseases,^25^ Alzheimer’s disease,^26^ and cancer.^27^ A comprehensive understanding of biofilm dynamics in the oral cavity is therefore paramount for developing preventative and therapeutic strategies that go beyond the traditional disinfection of the oral cavity.

Among the microorganisms inhabiting the oral cavity, *S. mutans* has emerged as a key player in biofilm formation.^28^ Oral biomacromolecules, such as bacterial surface-associated proteins, have been identified to play essential roles in biofilm production, structure and integrity.^28,29^ In contrast, small molecules implicated in the same tasks are much rarer, with the best studied examples indirectly involved via quorum sensing.^30^ Mutanofactins are a notable example of secondary metabolites tied to biofilm formation in *S. mutans*.^11^ They have been identified following a phenotypical screen from various strains of *S. mutans* and are polyketide/nonribosomal peptide synthetase derived natural products. Following isolation, Li et al. demonstrated that Muf-697 can affect *S. mutans* biofilm formation. This was attributed to a presumed increase in cell surface hydrophobicity. As no changes in bacterial gene expression were found, the possibility of Muf-697 acting as a signaling molecule was ruled out. Notably, only two out of five mutanofactins that were identified could be isolated and characterized. The intriguing preliminary study of the biological activity of a subset of mutanofactins and their connection to the human microbiome compelled us to probe the chemistry and biology of these natural products. By undertaking a total synthesis program that would provide pure, well characterized samples of all mutanofactins, we were keen on conducting an in-depth study to elucidate their roles in biofilm modulation. As such, we aim to increase understanding of the role of lipopeptides in bacterial adhesion, the dynamic interplay between microbial communities, and more broadly, the relationship between homeostasis and dysbiosis.

## Results and Discussion

### Synthesis Considerations

**Figure 2:**
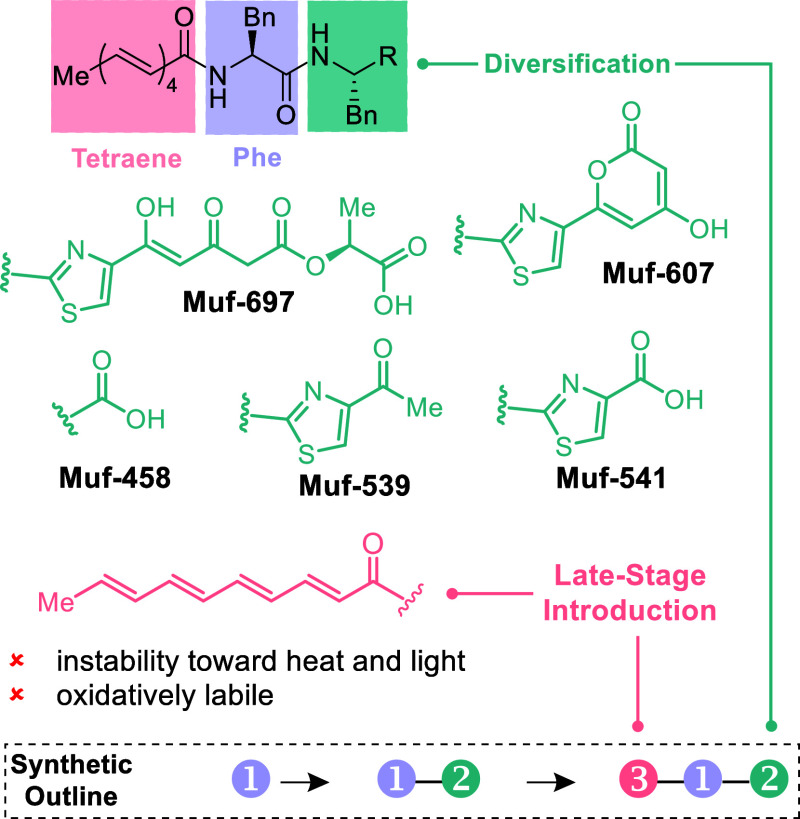
Retrosynthetic and strategic considerations.

Our analysis of the various mutanofactins (Figure 2) recognizes three domains, two of which are invariant, the tetraene (pink) and the phenylalanine (purple) motifs. The individual mutanofactins are differentiated by the variable terminal (turquoise) domain (carboxy, 4-acetyl thiazole, 4-carboxy thiazole, thiazolyl pyrone and polyketide substituted thiazole). Accordingly, we designed a synthesis starting from a core phenylalanine (purple) where the variable region is assembled from a second phenylalanine (Muf-458) or its thiazole derivative (Mutanofactins 539, 541, 607, 697). Muf-607 and Muf-697 incorporate thiazoles substituted with a hyroxypyranone or formally the product resulting of opening of the latter with lactic acid, leading us to envision a common precursor. Due to concerns regarding stability of the tetraenoic acid side chain toward light and oxygen,^31^ we decided to introduce this moiety late in the synthesis.^32^

### Synthesis of the Thiazole Core

**Scheme 1:**
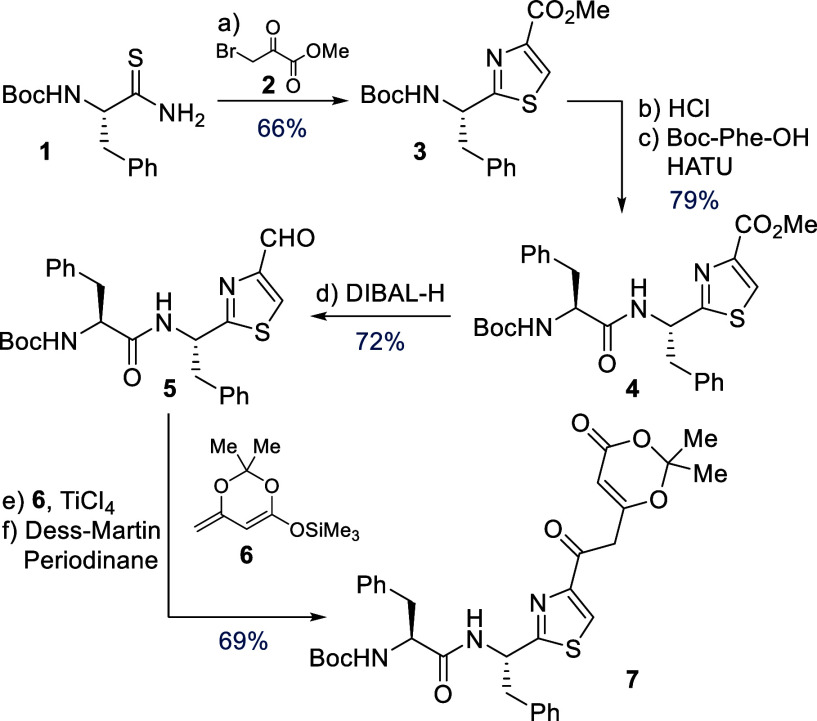
Synthesis of Dioxinone **7** Reagents and Conditions: (a) **2**, KHCO_3_, DME, r.t., 30 s, then 0 °C, 30 s, TFAA, pyridine, DME, 0 °C, 0.5 h, 66%; (b) HCl (4 M in dioxane), MeOH, 2 h; (c) Boc-Phe-OH, HATU, DIPEA, DMF, 0 °C, 1 h, 79% over two steps. (d) DIBAL-H, CH_2_Cl_2_, −78 °C, 3.5 h, 72%; (e) TiCl_4_, CH_2_Cl_2_, −78 °C, 20 min, then **6**, −78 °C, 3 h, 91%; (f) DMP, CH_2_Cl_2_, 0 °C, 2 h, 76%.

^1^H NMR and ^13^C NMR spectroscopic data have been reported only for Muf-607 and Muf-697, with the caveat that neither spectra were acquired from pure samples.^11^ Therefore, we initially focused on unambiguously assigning the structures of Muf-607 and Muf-697 through synthesis.^33^ Our synthetic studies commenced with the preparation of the thiazole core, present in all but one mutanofactin. To this end, thiourea **1** (>99% ee) was subjected to Hantzsch thiazole synthesis conditions with bromopyruvate **2**, to give thiazole **3** upon dehydration with (CF_3_CO)_2_O (Scheme 1). Initial experiments gave the thiazole in excellent yield, however with complete racemization, a frequent issue in the Hantzsch thiazole synthesis.^34^ Optimization included control of temperature and stoichiometry of reagents. Importantly, we found that rapid addition of bromopyruvate **2** to the solution of thiourea **1** was crucial to prevent racemization on large scale (see the Supporting Information for details). Under these conditions, desired product **3** could be obtained in 66% yield with minimal epimerization (>90% ee). *N*-Boc deprotection (HCl) followed by HATU mediated amide bond formation gave dipeptide **4** in 79% yield over two steps. DIBAL-H reduction furnished aldehyde **5** in 72% yield, along with 18% of the corresponding alcohol, which could be oxidized to **5** (see the Supporting Information for details). Based on the work by Sato, Kaneko and others,^35−37^ dienol silane **6** was used in a vinylogous Mukaiyama aldol reaction with aldehyde **5** and TiCl_4_ as Lewis acid. This protocol cleanly furnished the aldol product in 91% yield as an inconsequential mixture of diastereomers, which were oxidized in a subsequent step by DMP to yield dioxinone **7** in 76% yield.

**Scheme 2:**
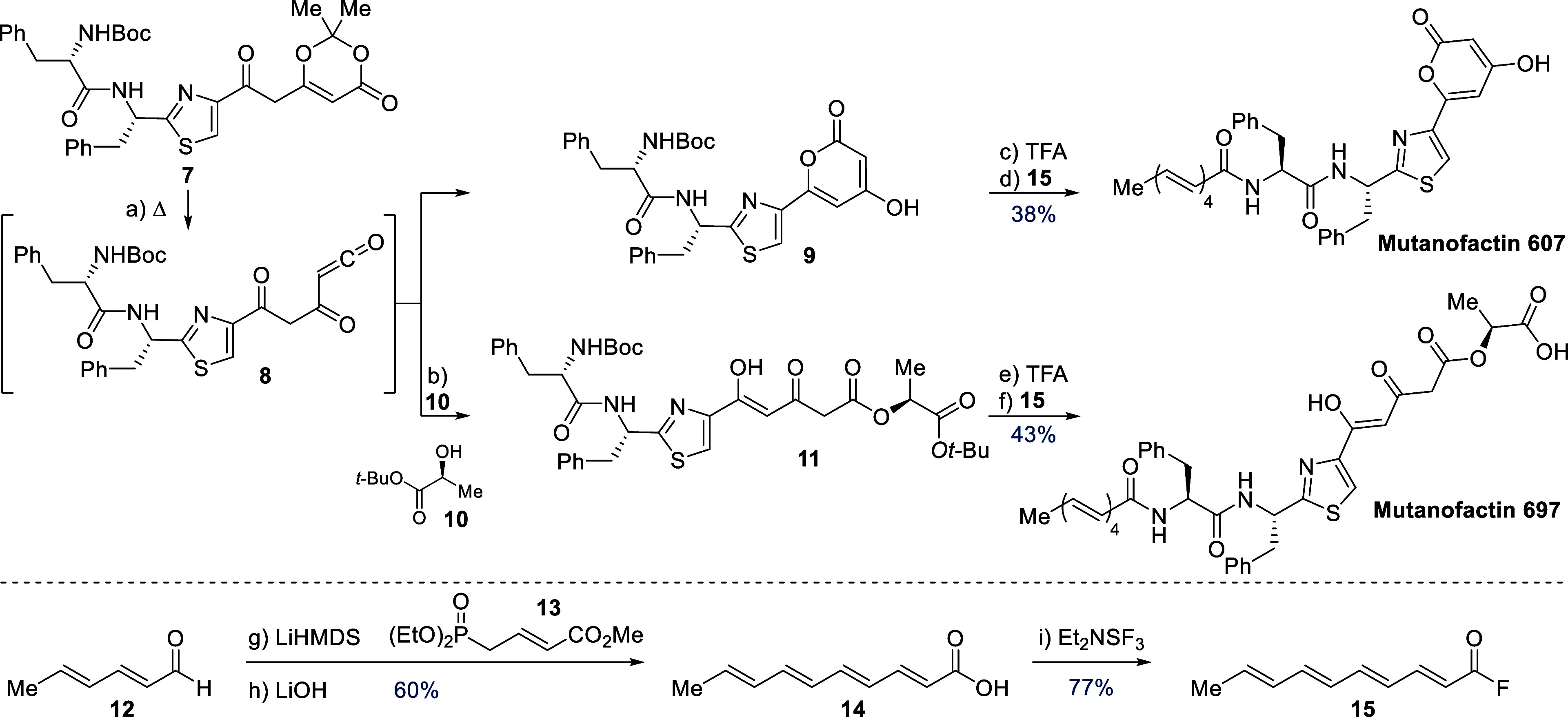
Synthesis of Mutanofactin 607 and Mutanofactin 697 Reagents and Conditions: (a) PhMe, 120 °C, 30 min; (b) **10**, PhMe, 120 °C, 30 min; (c) TFA, CH_2_Cl_2_, H_2_O, 0 °C to r.t., 2 h; (d) **15**, DIPEA, DMF, 0 °C to r.t., 16 h, 38% over three steps; (e) TFA, CH_2_Cl_2_, H_2_O, 0 °C to r.t., 6 h; (f) **15**, DIPEA, DMF, 0 °C to r.t., 16 h, 43% over three steps; (g) LiHMDS, THF, −10 °C, 10 min, then HMPA, −65 °C, 10 min, then **13**, −78 °C to r.t., 2 h, 60%; (h) LiOH, THF-MeOH (1:1), 50 °C, 3 h, quant; (i) DAST, Et_2_O, 0 °C, 1 h, 77%.

With dioxinone **7** in hand, we turned our attention to explore it as a precursor to both Muf-607 and 697 (Scheme 2). When a solution of dioxinone **7** was refluxed in toluene, putative acyl ketene **8** was formed via acetone extrusion.^38−40^ In the absence of an exogenous nucleophile, **8** cyclized to give pyrone **9**, en route to Muf-607. Alternatively, by addition of *tert*-butyl lactate **10** (available via a short sequence, see the SI) to the thermolysis reaction of **7**, the intermolecular addition product **11** was formed quantitatively.

With the key motifs of both Muf-607 and 697 installed, we focused on the synthesis and attachment of the tetraenoic acid fragment. Horner–Wadsworth–Emmons reaction of sorbaldehyde **12** with allylic phosphonate **13** gave the methyl ester, which was readily saponified with LiOH to yield tetraenoic acid **14** in 60% over two steps. *N*-Boc-deprotection of pyrone **9** and diketoester **11** with TFA proceeded smoothly, as monitored by TLC and LC-MS. Initial attempts at amide bond formation were however not met with success. Reactions between tetraenoic acid **14** and the free amine derived from either deprotected **9** or **11** under a variety of conditions (HATU, T3P, BOP,^41^ allenone^42^) led to either unreacted starting materials or decomposition. Minor product formation for Muf-607 was observed in a procedure with isobutylchloroformate and NMM that proceeds via the mixed anhydride.^43^ However, under these conditions we could not access Muf-697, which prompted us to pursue further investigations.

### Completion of the Syntheses

Acyl fluorides have long been used as reactive but selective coupling reagents,^44−46^ and, importantly, retinoyl fluoride has been reported as a stable but competent electrophile in amide bond formation.^47^ Accordingly, we prepared acyl fluoride **15** via DAST-mediated deoxyfluorination of the parent tetraenoic acid **14** in 77% yield.^48,49^ Gratifyingly, acyl fluoride **15** proved to be a suitable coupling partner to access Muf-607 (38%) and Muf-697 (43%) from dioxinone **7** over three steps. The spectroscopic data for both natural products were in accordance with the assigned structures.^11^

**Scheme 3:**
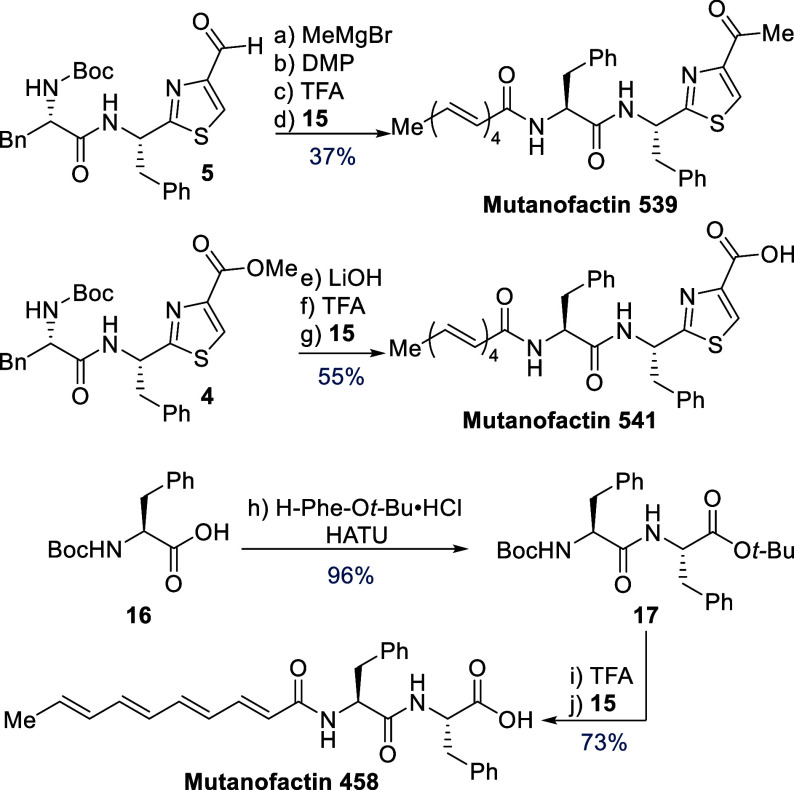
Synthesis of Muf-458, 539 and 541 Reagents and Conditions: (a) MeMgCl, THF, −78 °C to r.t., 3 h, 65%; (b) DMP, CH_2_Cl_2_, 0 °C to r.t., 3 h, 86%; (c) TFA, CH_2_Cl_2_, H_2_O, 0 °C to r.t., 2 h; (d) **15**, DIPEA, DMF 0 °C to r.t., 16 h, 66% over two steps.; (e) LiOH, THF, MeOH, 94%; (f) TFA, CH_2_Cl_2_, H_2_O, 0 °C to r.t., 30 min; (g) **15**, DIPEA, DMF, 0 °C to r.t., 16 h, 59% over two steps; (h) H-Phe-O*t*Bu·HCl, HATU, DIPEA, DMF,0 °C to r.t., 16 h, 96%; (i) TFA, CH_2_Cl_2_, PhOMe, 0 °C to r.t., 6 h; (j) **15**, DIPEA, DMF 0 °C to r.t., 16 h, 73% over two steps.

We next turned our attention to the remaining three members of the natural product family, capitalizing on suitable intermediates developed en route to Mutanofactins 607 and 697 (Scheme 3). The methyl ketone present in Muf-539 was accessed via the addition of methylmagnesium chloride to aldehyde **5**, yielding the corresponding alcohol as an inconsequential mixture of diastereomers in 65% yield. Oxidation with DMP cleanly furnished a methyl ketone in 86% yield. Subjecting this ketone to the established two step sequence consisting of TFA mediated *N*-Boc deprotection followed by amide bond formation with acyl fluoride **15** gave Muf-539 in 66% yield over two steps. The carboxylic acid in Muf-541 was accessed via saponification of methyl ester **4**, delivering the corresponding carboxylic acid in 96% yield. *N*-Boc deprotection followed by acyl fluoride coupling proved a suitable solution, delivering Muf-541 in 59% yield over the two steps. Finally, we turned our attention to the remaining member of the family, Muf-458. The Phe-Phe motif was constructed via HATU mediated amide bond formation, delivering dipeptide **17** in 96% yield. Utilizing our established two step sequence consisting of *N*-Boc and concomitant *tert*-butyl deprotection (TFA) followed by amide bond formation with acyl fluoride **15** gave rise to Muf-458 in 73% yield over two steps. With preparative amounts of these compounds in pure form, we provide full spectroscopic characterization for the first time (^1^H, ^13^C, HRMS, FTIR, OR), thus securing their structural assignment.^11^

### Mutanofactin Deficiency Yields Fragile Biofilm

With access to all five mutanofactins, we proceeded to evaluate their effects in various biologically relevant settings. In the experimental setup, bacteria are grown for 24 h in microplate wells using a biofilm promoting medium. Total biomass (growth) is measured in each well by optical density measurement at 595 nm (OD_595_). The wells are subsequently washed with PBS buffer, to remove planktonic bacteria, fixed and stained with crystal violet, which allows for the determination of the biofilm mass via the OD_595_. First, we tested an *S. mutans* NMT4863 deletion mutant lacking the putative mutanofactin biosynthetic genes *mufD*, *mufE*, *mufF*, and *mufG* (Δ*mufD–G*). A Δ*mufD–G* mutant was shown to be deficient in mutanofactin production,^11^ making this strain an ideal starting point for complementation studies with the synthetic compounds.

**Figure 3:**
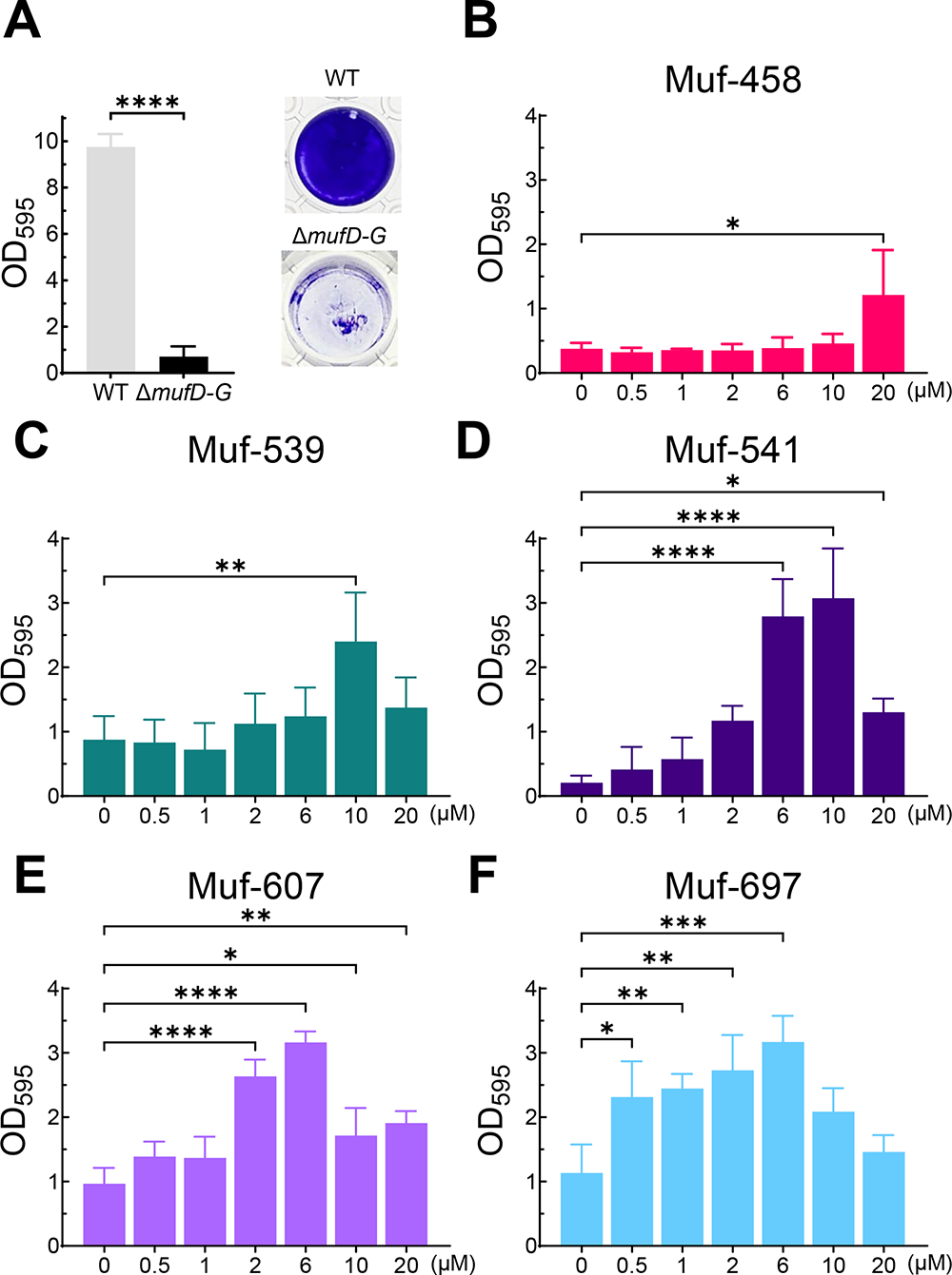
(A) Biofilm mass of *S. mutans* NMT4863 Δ*mufD–G* compared to the parent wild-type. (B–F) Effect of external provision of mutanofactins on biofilm mass of *S. mutans* Δ*mufD–G*. Bacteria were grown for 24 h, and biofilm mass was determined by OD_595_ upon crystal violet staining. Data are shown as the mean and SD of three biological replicates (*n* = 3), each tested in three technical replicates. Significance was tested using (A) a two-sample *t* test or (B–F) a one-way ANOVA for each mutanofactin with Dunnett’s test against the 0 μM condition (* = *p* ≤ 0.05, ** = *p* ≤ 0.01, *** = *p* ≤ 0.001, **** = *p* ≤ 0.0001).

Compared to the *S. mutans* NMT4863 wild-type strain, significantly less biofilm mass was determined for the mutanofactin deficient mutant (Figure 3A). We attribute this observation to more fragile biofilms, as even minor movements such as the plate movement during microplate reader measurements can disrupt the biofilms (see Supporting Information, Figure S1). This is in line with the report by Li et al.^11^ who noted that biofilms from mutanofactin deficient *S. mutans* were easily disrupted by shaking. We hypothesized that the measured reduction in biofilm mass of the Δ*mufD–G* mutant is a result of reduced biofilm stability and/or attachment, rather than the overall capacity to form biofilms. To test this, we investigated the influence of the washing steps, which impose mechanical stress on the formed biofilms. Without washing, there was no significant difference in observed biofilm mass between the *S. mutans* NMT4863 wild-type and the Δ*mufD–G* mutant, while with each additional washing step (1–3 washings), the difference in biofilm mass became more pronounced (see Supporting Information, Figure S2). Thus, the ability to produce mutanofactins leads to a more mechanically resistant biofilm formation.

### Exogenous Mutanofactins May Restore Biofilm Mass in a Mutanofactin-Deficient Mutant

Next, we assessed the effect of exogenous mutanofactins (Muf-458, Muf-539, Muf-541, Muf-607 and Muf-697) on the mutanofactin deficient mutant (Δ*mufD–G*). We measured overall growth and the biofilm mass after two washing steps, quantifying the residual biofilm mass after shearing. The compounds were added from the start at concentrations ranging from 0.5 to 20 μM. Interestingly, we found that all five mutanofactins conferred a significant increase in biofilm mass of the mutanofactin deficient mutant (Figure 3B–F). The addition of Muf-697 increased the biofilm mass already at a concentration of 0.5 μM, whereas Muf-539 and Muf-458 had a biofilm-promoting effect at concentrations of 10 μM and 20 μM, respectively.^50^

Remarkably, the total biomass (growth) of the Δ*mufD–G* mutant remained largely unaffected by the mutanofactins at any concentration range tested (see Supporting Information, Figure S3). This indicates that the observed increase in biofilm mass upon exogenous supply of mutanofactins is not due to enhanced growth but rather because of altered biofilm structure.^11^ Our results clearly demonstrate that each mutanofactin results in a dose-dependent response of residual biofilm mass, namely all mutanofactins are biofilm promoting. We further observed a hormetic effect, namely, at increased concentrations, the biofilm promoting effect decreases for mutanofactins 539, 541, 607, and 697 (Figure 3C–F).

### Exogenous Mutanofactins Decrease Biofilm Mass of the Producer Strain

**Figure 4:**
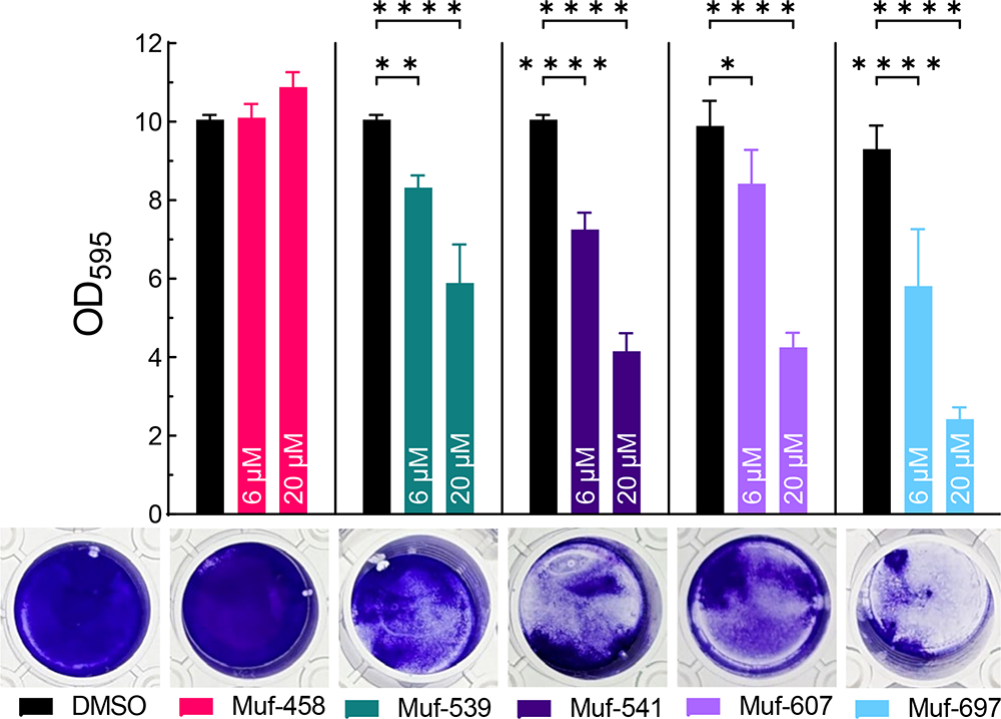
Effect of mutanofactins (6 μM and 20 μM) on the biofilm mass of the natural mutanofactin producer *S. mutans* NMT4863. Bacteria were grown for 24 h, and biofilm mass was determined by OD_595_ upon crystal violet staining. Data are shown as the mean and SD of three biological replicates (*n* = 3), each tested in three technical replicates. Significance was tested using a two-way ANOVA with Dunnett’s test against the DMSO control (* = *p* ≤ 0.05, ** = *p* ≤ 0.01, **** = *p* ≤ 0.0001). Representative images of stained biofilms for each mutanofactin at 20 μM and the DMSO control are shown below.

Our finding that higher concentrations (e.g., 20 μM for Muf-697) prove less efficacious at biofilm enhancement prompted us to study the effect of exogenous mutanofactins on a natural mutanofactin producer, *S. mutans* NMT4863. Supplementing the growth medium with Muf-539, Muf-541, Muf-607, or Muf-697 at 6 μM and 20 μM resulted in a significant reduction of biofilm mass (Figure 4). This effect was more pronounced at 20 μM and strongest with Muf-697, followed by Muf-541 and Muf-607. The total biomass (growth) according to optical density measurement (OD_595_) was not significantly reduced (see Supporting Information, Figure S4). Concomitantly, there was a disruption in the *S. mutans* NMT4863 biofilms at 20 μM of Muf-541, Muf-607, and Muf-697 (see Supporting Information, Figure S5) during the microplate reader measurement, comparable to our findings with the mutanofactin deficient strain Δ*mufD–G*. Therefore, we hypothesize that *S. mutans* NMT4863 might employ an autoregulating mutanofactin production mechanism for adaptation to its niche. While this requires further investigation, it is conceivable that the amphiphilic nature of mutanofactins disrupts biofilm structure at distinct concentrations, which has been reported for various amphiphiles.^51,52^

### Mutanofactins Influence Biofilm Formation of Cohabiting Oral Bacteria

**Figure 5:**
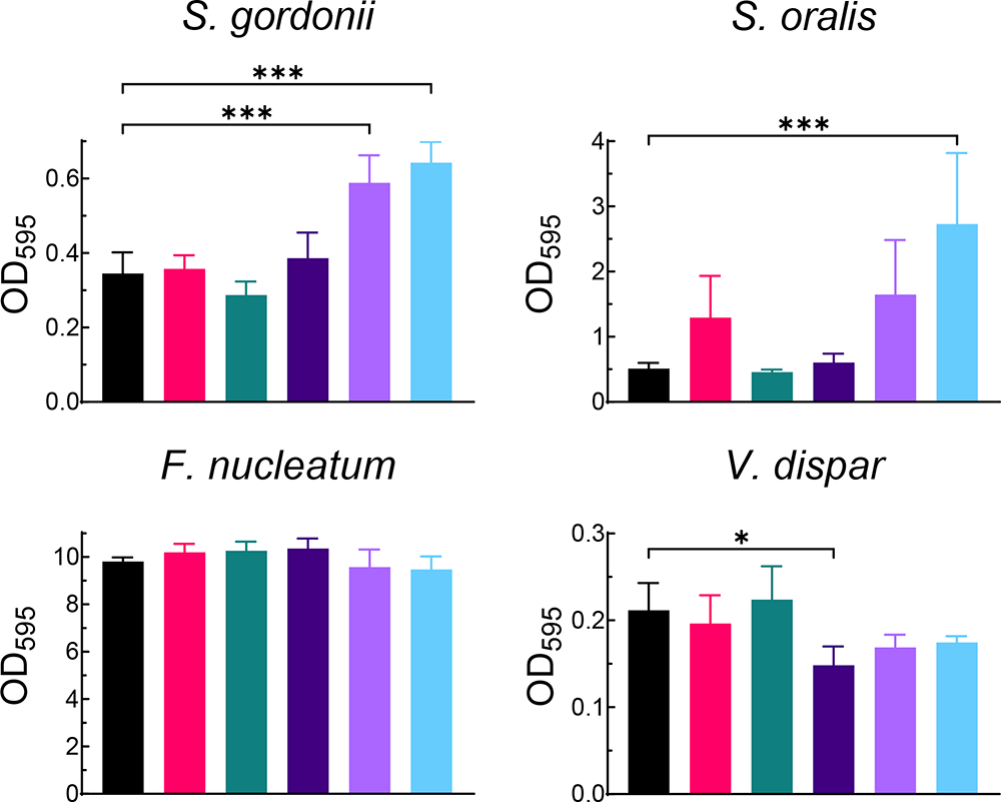
Biofilm mass of oral cohabitants of *S. mutans*, including *S. gordonii*, *S. oralis*, *F. nucleatum*, and *V. dispar* in response to mutanofactins (6 μM). Bacteria were grown for 24 h, and biofilm mass was determined by OD_595_ upon crystal violet staining. Data are shown as the mean and SD of three to four biological replicates (*n* = 3–4), each tested in three technical replicates. Significance was analyzed using a one-way ANOVA for each bacterium with Dunnett’s test against the DMSO control (* = *p* ≤ 0.05, *** = *p* ≤ 0.001).

Next, we explored the effect of mutanofactins at 6 μM on cohabiting oral bacteria which are known for their interaction with *S. mutans* and, therefore, might be exposed to secreted mutanofactins (Figure 5). *S. gordonii* and *S. oralis*, both early biofilm colonizers and antagonists of *S. mutans*, play a crucial role in limiting its proliferation and maintaining oral health.^53−57^ Both antagonist-bacteria responded similarly to mutanofactin exposure as the mutanofactin deficient *S. mutans* NMT4863 Δ*mufD–G* strain. Biofilm mass was significantly increased upon cultivation in the presence of 6 μM Muf-607 or Muf-697. For *S. gordonii*, the biofilm appeared more stable in both cases, as evident by less biofilm disruption during the microplate reader measurement (see Supporting Information, Figure S6). Among the bacteria tested within the framework of this study, *S. oralis* was unique. When treated with 6 μM Muf-697, both biofilm mass and total biomass were increased significantly (Figures 5 and S6). This suggests that treatment with Muf-697 impacts biofilm formation in *S. oralis* also through enhanced growth.

We additionally included *F. nucleatum* and *V. dispar* in our experiments, which are known for their mutualistic relationship with *S. mutans*.^58,59^ For *F. nucleatum*, neither total biomass nor biofilm mass were measurably affected by exposure to mutanofactins (Figure 5). *V. dispar* showed a minimal, though statistically significant, reduction in biofilm mass in response to 6 μM Muf-541, with no effect on the total biomass.^60^ Our study highlights for the first time the species-specific effect of mutanofactins on members of the oral microbiome.

### Muf-697 Does Not Increase CSH of Planktonic *S. mutans*

In an aqueous environment such as the oral cavity, cell surface hydrophobicity is implicated in increased bacterial aggregation, surface adhesion, and biofilm formation.^61,62^ Li et al. proposed in their work that Muf-697 promotes biofilm formation via coating of the surface of *S. mutans* strains, resulting in increased cell surface hydrophobicity (CSH).^11^ The increased CSH of *S. mutans* producing Muf-697 was only determined by contact angle measurements on bacterial lawns of cells extracted from mature biofilms,^11^ where other factors such as extracellular proteins or matrix fragments from biofilm growth can also be present. In order to determine if mutanofactins influence biofilm formation via CSH during early stages of biofilm formation, we designed an experiment to probe directly whether mutanofactins alter the wettability of *S. mutans* cells. Accordingly, planktonic *S. mutans* exposed to Muf-697 was investigated with contact angle (CA) and microbial adhesion to hydrocarbons (MATH) before and after the mutanofactin exposure. Both strains that can and cannot express mutanofactins were included. CA measurements on mature colony biofilms were also performed. Neither CA nor MATH measurements were generally affected by the presence of Muf-697 (see the Supporting Information for details). This implies that an increased CSH of planktonic *S. mutans* is irrelevant to the increased biofilm formation resulting from Muf-697 exposure.

Hence, we were prompted to explore alternative mechanisms by which mutanofactins could promote biofilm formation. Generally, formation of biofilms proceed through bacterial adhesion to a surface and subsequent formation of an extracellular matrix.^63^ We hypothesized that improved adhesion and stability of the biofilm could also be achieved by conditioning of the environment by *S. mutans*. One possible mechanism would be the association of mutanofactins onto a substrate surface, rendering it more hydrophobic and thus priming the surface for bacterial, specifically *S. mutans*,^64^ adhesion.

### Muf-697 Uniquely Affects Mucin Structure

Salivary mucins are a natural defense barrier against adhesion by cariogenic bacteria, including *S. mutans*,^22^ primarily as part of the salivary pellicle. Their bottle-brush structure with highly hydrated glycan side chains provides a protective coating that suppresses bacterial attachment.^65^ We envisioned three potential mechanisms for mutanofactins’ influence on bacterial adhesion: (1) Binding to mucin and presenting their lipophilic part to promote adhesion. (2) Physical detachment or partial displacement of mucin from the substrate surface. (3) Changes in the morphology of the mucin layer, such as exposing the more hydrophobic backbone.

Commercially available porcine type II mucin was used as a model for salivary mucin in our studies. Mucin adsorbed on hydroxyapatite (mimicking tooth enamel^66^) and silica surfaces, formed hydrophilic coatings showing typical water drop pinning for polymer brushes in contact angle measurements.^67^ Contact angle measurements performed on mucin layers revealed that Muf-697 increases the contact angle of water on mucin compared to untreated mucin (see Supporting Information, Figure S14). The results for mucin layers formed on silica and hydroxyapatite were very similar. These findings enable multitechnique investigations using silica instead of hydroxyapatite as a representative substrate, vide infra.^68^

**Figure 6:**
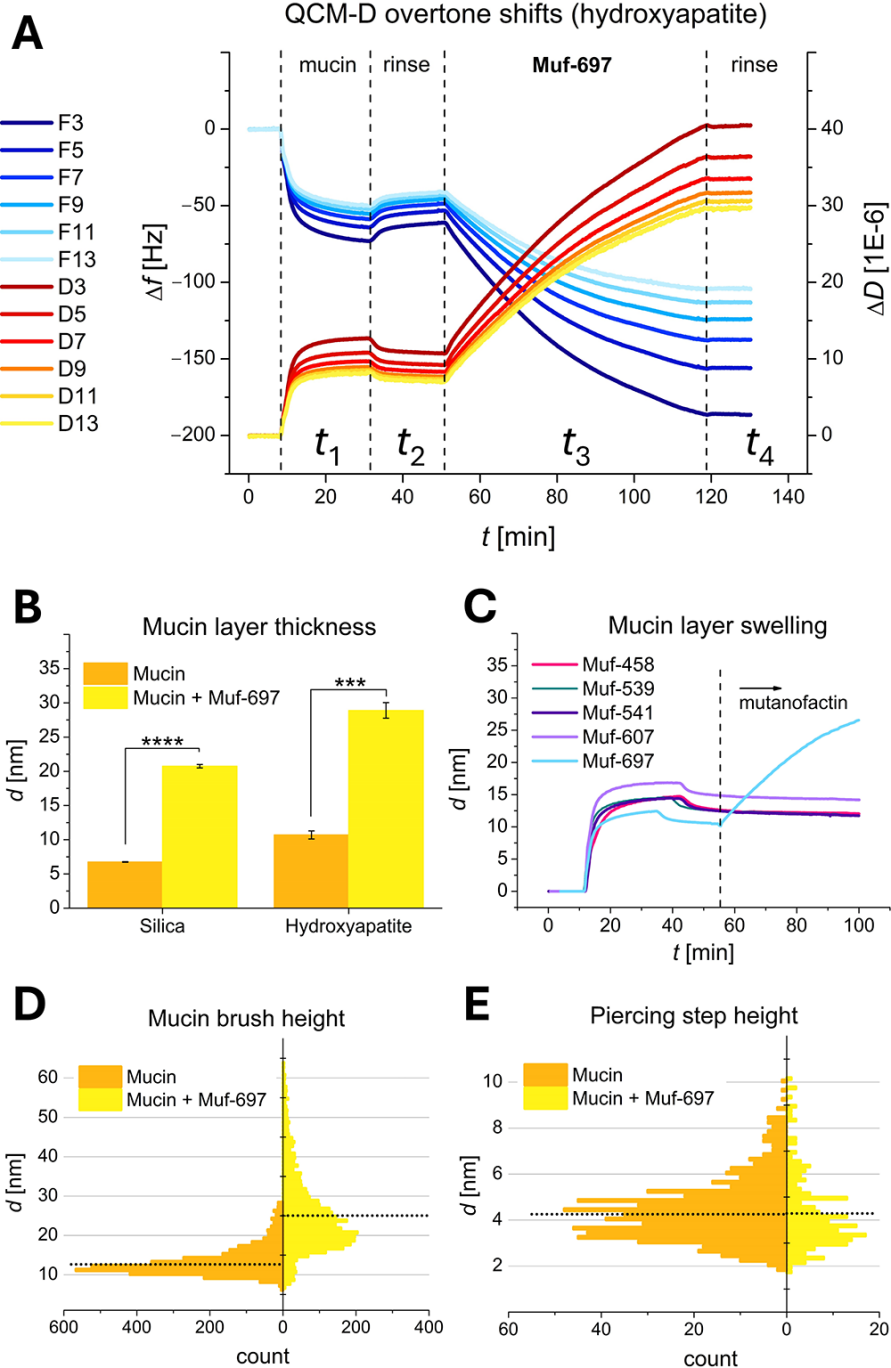
Investigation of mucin layers and mutanofactin-incubated mucin layers on silica and hydroxyapatite using QCM-D and AFM force spectroscopy. (A) Example frequency (*Δf*, cold colors) and dissipation (*ΔD*, warm colors) changes for sensor overtones 3–13 on hydroxyapatite. (B) Adsorbed layer thickness of mucin (orange) and Muf-697 incubated mucin (yellow). Statistical significance of technical triplicates calculated with Pair-Sample *t* tests (*** = *p* ≤ 0.001, **** = *p* ≤ 0.0001). (C) QCM-D measurements of mucin exposed to mutanofactins. Dashed lines mark when a component is introduced into the system. (D) Layer thickness measured by AFM on different positions on mucin-coated borosilicate glass coverslips. (E) Layer piercing step height measured by AFM. Orange: mucin layer; yellow: Muf-697 incubated mucin layer. Dotted lines mark mean values.

QCM-D is an ideally suited technique to investigate changes in the mucin layer upon incubation with Muf-697.^69^ This acoustic technique registers small changes in the resonance frequency of an oscillating coated quartz crystal sensor as a function of mass adsorbed onto the sensor surface. Adsorption of molecules (e.g., mucin or mutanofactin) on the crystal results in decreased resonance frequency and increased damping (dissipation) for viscous layers. Frequency changes and changes in the energy dissipation per oscillation are recorded in real-time. In the experiment, a hydroxyapatite-coated QCM-D sensor in a flow cell is sequentially exposed to solutions of mucin (*t*_1_), PBS buffer (*t*_2_), followed by Muf-697 (*t*_3_). As shown in Figure 6A, drops in the resonance frequencies (cold colors) and increases in the oscillation dissipations (warm colors) are observed, when a mucin solution is introduced to the system (*t*_1_). This indicates adhesion of a strongly hydrated mucin layer on the surface.^70^ After washing, weakly attached mucin is removed (*t*_2_). Fitting the QCM-D response to the frequency-dependent Voigt model for a viscoelastic solid layer allows us to determine the thickness of the remaining mucin layer as ∼11 nm (Figure 6B).^71^

When 6 μM Muf-697 is introduced into the system (*t*_3_), there is a second, very pronounced drop in the sensor resonance frequencies and an increase in dissipation values, implying a large increase in the thickness of the adsorbed layer. The small molecular weight (697 Da) and size of Muf-697 cannot account for the increased layer thickness, and control experiments confirmed that Muf-697 does not bind directly to the hydroxyapatite-coated sensor surface (see Supporting Information, Figure S15). The increased thickness or apparent swelling of the mucin layer exposed to Muf-697 effect is irreversible, as no further shifts in frequencies or dissipation values are recorded after rinsing (*t*_4_). The mucin layer is, on average, three times thicker than before Muf-697 exposure (Figure 6B).

Interestingly, the increased layer thickness is only observed for Muf-697, while the other mutanofactins do not affect the mucin layer (Figure 6C). This points to a unique interaction between Muf-697 and the mucin layer. Recently, in a variety of structures, specific hydrogen-bonding arrays have been found to be privileged in the interaction of small molecules with mucin.^72^ Similar hydrogen-bonding patterns may be accessible to Muf-697 via tautomerization of the diketoester, consistent with spectroscopic observations.^73^ We thus propose that the effect of Muf-697 on the mucin layer is unique, because the remaining mutanofactins cannot display similar hydrogen bonding arrays. Another possible mechanism is the complexation of Muf-697 with divalent cations and thereby association with the anionic polysaccharides.

When the experiments were performed on silica instead of hydroxyapatite substrates, similar mucin adsorption and layer thickness increases after Muf-697 exposure were observed. Both are hydrophilic and negatively charged substrates, and mucin layer thickness also roughly triples from 6.8 to 20.8 nm on silica upon exposure to Muf-697, reinforcing the interpretation that Muf-697 interacts directly with the mucin layer (see Figure 6B and Figure S13).

We used AFM force–distance mapping to get complementary, detailed information on the structural changes in mucin layers exposed to Muf-697. With AFM, we can measure the lateral heterogeneity of thickness, hydrated polymer repulsive force and the force required to displace mucin by probing different points on the surface. Individual mucin molecules are laterally displaced in response to the pressure exerted locally by the AFM tip. Two different features are captured in the histograms in Figure 6, namely mucin brush height (Figure 6D) and piercing step height (Figure 6E). The hydrated mucin layer height was determined to be on average 13 nm by fitting a modified Alexander–de Gennes model,^74^ representing the compression of a polymer brush on a displaceable layer. This average height is similar but slightly higher than the hydrated layer height estimated by QCM-D. The results after Muf-697 treatment also align with the QCM-D results, as the average brush height almost doubles to approximately 25 nm. The height distribution also becomes broader, indicating a more heterogeneous mucin layer. The increase in heterogeneity is also visible in the piercing step height and force histograms. The average step height for pure mucin layers is about 4 nm, following a symmetrical distribution (Figure 6E). The average step height is almost the same after exposure to Muf-697, however, fewer force curves exhibited a piercing step through the mucin layer, and the observed piercing steps are skewed to lower heights. This indicates that loci exposing the substrate of similar size to the tip (radius ∼20 nm) resulted from the rearrangement of the mucin layer. The distribution of the piercing force was broadened after treatment with Muf-697 and increased from approximately 76 pN to 199 pN (see Supporting Information, Figure S16). Counterintuitively, a higher displacement force after Muf-697 exposure suggests a strengthened interaction between mucin molecules or increased surface adhesion in areas where the mucin layer has not formed gaps of exposed substrate. In summary, the QCM-D and AFM measurements suggest that Muf-697 uniquely swells the adhered mucin layer. We propose this is due to a change in mucin layer morphology and topography, with some mucins extending from the surface while reducing layer thickness homogeneity and exposing the underlying surface. Hence these findings could support hypotheses (2) and (3).

### Muf-697 Promotes *S. mutans* Adhesion on Mucin-Coated Surfaces

We followed up the mechanistic results with studying the effect of Muf-697 on the adhesion of *S. mutans* on a mucin-coated surface. *S. mutans* NMT4863 Δ*mufC–J* adhesion was imaged in real-time by bright field microscopy in a flow cell,^75^ exposed to three different conditions: (a) bare silica as a control, (b) mucin-coated silica, and (c) a mucin layer on silica treated with Muf-697.

**Figure 7:**
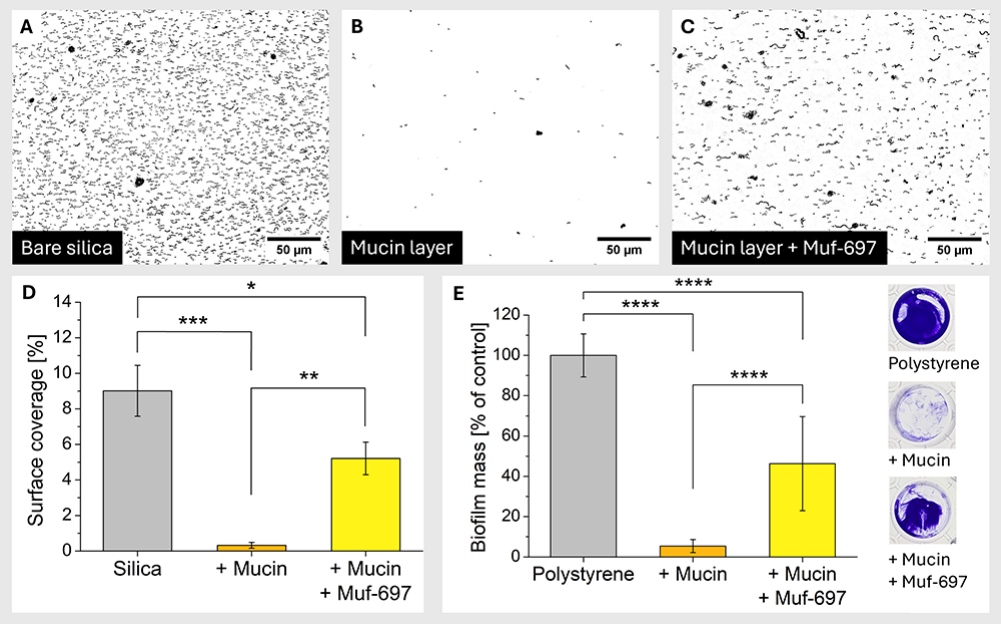
*S. mutans* Δ*mufC–J* adhesion in flow (50 μL min^–1^) investigated using a microfluidics setup and brightfield microscope. (A–C) Exemplary micrographs of bacterial surface coverage after 1 h on (A) bare silica, (B) mucin-coated silica, and (C) 6 μM Muf-697-incubated mucin layer on silica. The images are background-subtracted and contrast-enhanced using ImageJ software. (D) Bacterial surface coverage after 1 h. Each bar represents the mean and SD of biological triplicates. (E) Biofilm mass of *S. mutans* Δ*mufC–J* after 24 h. Each bar represents the normalized mean (noncoated polystyrene set to 100%) and SD of a minimum of nine biological replicates (*n* ≥ 9) tested in three technical replicates. The photo inset shows representative crystal violet stained biofilms. Significance was analyzed using two one-way ANOVA with Tukey’s multiple comparisons tests (* = *p* ≤ 0.05, ** = *p* ≤ 0.01, *** = *p* ≤ 0.001, **** = *p* ≤ 0.0001).

The results show that bacteria attach to the bare glass substrate (Figure 7A). Mucin coating effectively prevents bacteria from adhering to the surface (Figure 7B), as other studies investigating the protective properties of mucin layers have similarly shown.^22^ The surface coverage drops from around 9% on uncoated glass to below 0.5% on mucin-coated glass (Figure 7D). However, if the mucin layer is incubated with 6 μM Muf-697 before bacteria are introduced, *S. mutans* NMT4863 Δ*mufC–J* surface coverage increases significantly compared to the mucin-coated surface to around 5% (Figure 7C and 7D). Hence, Muf-697 interaction with the mucin layer allows bacteria to directly adsorb to the mucin layer or, more likely, to exposed areas of the underlying substrate. Next, we investigated, whether Muf-697 treatment of a mucin coating would also allow for biofilm formation upon adhesion of bacteria. Accordingly, polystyrene well plates were treated with mucin, and biofilm formation of *S. mutans* NMT4863 Δ*mufC–J* was measured. The mucin-coating led to substantially reduced biofilm formation (Figure 7E), in line with prior observations.^22^ If the mucin-coated wells were treated with a 6 μM Muf-697 solution prior to introduction of *S. mutans* NMT4863 Δ*mufC–J*, a substantial increase in biofilm mass from 5% to 46% was observed compared to unexposed mucin-coated surfaces. We thus conclude that the Muf-697–mucin interaction not only allows for adhesion of bacteria to the treated surface, but also for subsequent biofilm growth. It is important to note that this interaction is a distinct mode of action and an addition to the already known ways how mutanofactins elicit their function in a biological context.

## Conclusions

The entire mutanofactin family, mutanofactins 458, 539, 541, 607, and 697 was synthesized in a concise fashion, accessing the natural products analytically pure, allowing for unambiguous characterization. Our modular approach enabled early diversification, and provides a blueprint for the synthesis of related NRPS-polyketide natural products as well as analogues and chemical probes. A key acyl-ketene intermediate gave access to the two most complex members, namely Muf-607 and Muf-697 from a common precursor. For the final amide bond formation, a tetraenoic acid-derived acyl fluoride proved crucial for success. The late-stage introduction of the polyene proved important due to its sensitivity toward oxidation. With the synthesized natural products in hand, we were able to demonstrate that all mutanofactins promote biofilm formation or stability when exogenously applied to a mutanofactin-deficient mutant *S. mutans* strain. Importantly, we demonstrate that mutanofactins improve biofilm formation by *S. oralis* and *S. gordonii*, while not or only marginally influencing *V. dispar* and *F. nucleatum*. The differing effect of mutanofactins on inhabitants of the oral microbiome is intriguing, and it highlights the key role of *S. mutans* in its habitat. While a role for mutanofactins in directly increasing CSH to promote biofilm formation was ruled out, we found a conditioning effect of mucin-coated surfaces. Muf-697, most likely due to the availability of a hydrogen bonding array lacking in other mutanofactins, bound to and introduced morphological and topographical changes in mucin layers. *S. mutans* adhesion and subsequent biofilm formation was strongly promoted by the Muf-697 induced structural changes, which holds implications for early stage biofilm formation in the oral microbiome. To the best of our knowledge, Muf-697 is the first small molecule that primes a mucin layer to allow bacterial adhesion. Our microbiological and biophysical studies support that *S. mutans* conditions the local environment through mutanofactins, influencing its ability to adhere to surfaces and form stable biofilms. In a broader sense, in-depth understanding of the role of secondary metabolites within the human oral microbiome holds promise for modulating oral homeostasis, thus preventing dysbiosis and disease.

## Supporting Information Available

Experimental procedures and analytical characterization of all synthesized compounds; details of microbiological experiments and biophysical measurements (PDF)

## Abbreviations

NMR: nuclear magnetic resonance
TLC: thin layer chromatography
LC-MS: liquid chromatography-mass spectrometry
TFAA: trifluoroacetic anhydride
TFA: trifluoroacetic acid
DMP: Dess–Martin periodinane
HATU: hexafluorophosphate azabenzotriazole tetramethyl uronium
NMM: *N*-methylmorpholine
DAST: diethylaminosulfur trifluoride
CSH: cell surface hydrophobicity
QCM-D: quartz crystal microbalance with dissipation monitoring
AFM: atomic force microscopy
FTIR: Fourier-transform infrared spectroscopy
OR: optical rotation
