# Supplementary material for: Synthesis, Microbiology, and Biophysical Characterization of Mutanofactins from the Human Oral Microbiome
Source: ACS Cent Sci. 2025 Mar 27;11(4):601–11. doi: 10.1021/acscentsci.4c02184 (PMC12022917; doi:10.1021/acscentsci.4c02184)

Supporting Information

Synthesis, Microbiology and Biophysical Characterization of Mutanofactins from the Human Oral Microbiome

Lukas Lüthy,[a,‡] Leon G. S. Thies,[b,‡] Konstantin N. Beitl,[c,‡] Moritz E. Hansen,[a] Joshua B. McManus,[a] Muhammad Afzal,[b] Lukas Schrangl,[d] Susanne Bloch,[b] Guruprakash Subbiahdoss,[c] Erik Reimhult,*[,c] Christina Schäffer*[,b] and Erick M. Carreira*[,a]

[a] Department of Chemistry and Applied Biosciences, Laboratory of Organic Chemistry, ETH Zürich, 8093 Zürich, Switzerland

[b] Inistitute of Biochemistry, *NanoGlycobiology* Research Group, Boku University, 1190 Vienna, Austria

[c] Institute of Colloid and Bioinointerface Science, Boku University, 1190 Vienna, Austria

[d] Institute of Biophysics, Boku University, 1190 Vienna, Austria

Table of Contents

1 Safety Statement 3

2 General Considerations 4

3 Experimental Procedures 5

Optimization of Hantzsch’ Thiazole Synthesis: 8

Comparison of Synthetic and isolated Mutanofactin 607 21

Comparison of Synthetic and isolated Mutanofactin 697 33

HRMS/MS Comparison for Mutanofactin 43

HRMS/MS Comparison for Mutanofactin 541 47

HRMS/MS Comparison for Mutanofactin 458 51

4 Microbiology 52

Bacterial Growth Conditions 52

Mutanofactin Stock solutions 53

Construction of *S. mutans* NMT4863 Δ*mufD-G* and Δ*mufC-J* 54

Biofilm growth assays 56

Washing assay 58

Growth Curves 63

Biofilm Assays on Mucin-Coating 64

5 Physicochemical Characterization 65

General Considerations: 65

Cell Surface Hydrophobicity - Microbial Adhesion to Hydrocarbons (MATH) 66

Cell Surface Hydrophobicity – Contact Angle 67

Biofilm Wetting Assays 68

Bacteria Adhesion Under Flow 69

Water Contact Angles 70

Quartz Crystal Microbalance with Dissipation Monitoring (QCM-D) 71

Atomic Force Microscopy 73

6 NMR Spectra 75

1 Safety Statement

No unexpected hazards or dangers were encountered during the synthetic work presented within this manuscript. Following risk mitigation strategies were pursued for general work in the laboratory, and specific chemicals:

- **Standard PPE** (Personal protective Equipment), including safety goggles, protective gloves as well as a laboratory coat was worn at all times during experimentation. All experiments were carried out in a well-ventilated fumehood.
- **Lawesson's Reagent:** Used in large quantities in the first steps of the synthesis (25.0 g), which could result in the formation of large amounts of malodorous byproducts. This was avoided by a careful quench. Furthermore, all glassware and aqueous phases that came into contact with Lawesson's reagent were oxidized with bleach and left standing in a well-ventilated fumehood overnight.
- **Coupling Reagents:** (i.e. HATU, T3P, etc.) are all potentially sensitizing compounds,[1] and were thus used with minimal personal exposure and weighed out on a balance in a well-ventilated fumehood.
- **DAST:** ((Diethylamino)sulfur trifluoride, CAS: 38078-09-0) is a toxic and corrosive liquid that may release toxic HF upon exposure to water, and is known to decompose into explosive substances upon heating.[2] Accordingly, the material was, once distilled, kept in a plastic vial in the freezer, and never heated. Reactions involving DAST were carefully quenched with sat. $NaHCO_3$, and the pH was checked to be above >7.
- **Mutanofactins 458, 539, 541, 607 and 697:** Since all five natural products are bioactive, special care was taken to prevent exposure, especially when handling lyophilized powders.
- **Biosafety Levels:** *S. gordonii*, *S. oralis* and *F. nucleatum* are biosafety level 2 classified. Accordingly extra care was taken with their handling, and BSL-2 protocols were adhered to strictly.

[1] McKnelly, K. J.; Sokol, W.; Nowick, J. S. Anaphylaxis Induced by Peptide Coupling Agents: Lessons Learned from Repeated Exposure to HATU, HBTU, and HCTU. *J. Org. Chem.* **2020**, *85* (3), 1764–1768. DOI: 10.1021/acs.joc.9b03280.
[2] Messina, P. A.; Mange, K. C.; Middleton, W. J. Aminosulfur trifluorides: relative thermal stability [1]. *J. Fluor. Chem.* **1989**, *42* (1), 137–143. DOI: https://doi.org/10.1016/S0022-1139(00)83974-3.

2 General Considerations

General Practice: Unless otherwise noted, all non-aqueous reactions were carried out under Nitrogen atmosphere, in oven dried glassware. Reagents were purchased from commercial suppliers (ABCR, ACROS, Sigma Aldrich, Fluka, TCI, Strem, Alfa, Combi-Blocks or Fluorochem) and where appropriate purified according to known methods.[3] Anhydrous solvents over molecular sieves were purchased from Acros and used as received. Analytical thin layer chromatography (TLC) was performed on Merck silica gel 60 F254 TLC glass plates and visualized with 254 nm light and potassium permanganate (1.50 g $KMnO_4$, 10.0 g K_2CO_3, 1.25 mL 10% NaOH, 200 mL water), ceric ammonium molybdate (10.0 g Cerium(IV)sulfate, 25.0 g phosphomolybdic acid, 940 mL water 60.0 mL conc. sulfuric acid) or ninhydrin (1.50 g ninhydrin, 200 mL *n*-butanol, 3.00 mL AcOH) staining solutions followed by heating. Organic solutions were concentrated by rotary evaporation at 40 °C. Chromatographic purification of reaction products was carried out by flash chromatography using Brunschwig silica 32-63, 60Å under 0.3–0.5 bar overpressure.

HPLC purification: Carried out on a Waters Auto Purification System using a Dr. Maisch Reprosil Gold 120 C4 150 x 20 mm column, using a flowrate of 26.5 mL/min. Gradients of water and acetonitrile were used, both with 0.1% Formic acid (v/v) as additive. The column was warmed to 40 °C during purification. Fractions containing product (as determined via MS, UV_{330} or LC-MS analysis) were combined, and lyophilized for 2 days, shielded from ambient light.

NMR Spectroscopy: ^{1}H NMR spectra were recorded on a Bruker AVIII 600 MHz spectrometer with He or prodigy N_2 cryoprobes, Bruker AVIII HD 500 MHz and 400 spectrometers as well as Bruker Neo 500 MHz and 400 MHz spectrometers, and are reported in ppm with the solvent resonance as the reference ($CDCl_3$ at 7.26 ppm, DMSO-*d₆* at 2.50 ppm, CD_3OD at 3.31 ppm). Peaks and their apparent multiplicities are reported as (s = singlet, d = doublet, t = triplet, q = quartet, m = multiplet, br = broad signal, coupling constant(s) in Hz, integration). ^{13}C NMR spectra were recorded with ^{1}H-decoupling on Bruker AVIII 150 MHz spectrometers with He or prodigy N_2 cryo-probes, Bruker AVIII HD 125 MHz and 100 MHz spectrometers as well as Bruker Neo 125 MHz and 100 MHz spectrometers, and are reported in ppm with the solvent resonance as the reference unless noted otherwise ($CDCl_3$ at 77.16 ppm, DMSO-d_6 at 39.52 ppm, CD_3OD at 49.00 ppm). ^{31}P NMR spectra were recorded with ^{1}H-decoupling on Bruker AVIII HD 162 MHz spectrometers as well as Bruker Neo 162 MHz spectrometers, and are reported in ppm. ^{19}F NMR spectra were recorded with ^{1}H-decoupling on Bruker AVIII HD 282 MHz spectrometers as well as Bruker Neo 282 MHz spectrometers, and are reported in ppm.

IR Spectroscopy: Infrared spectra were recorded neat on a Perkin-Elmer Spectrum Two FT-IR spectrometer. The peaks are reported as absorption maxima (cm^{-1}).

HRMS: High resolution mass spectrometric data were obtained at the mass spectrometry service operated by the Laboratory of Organic Chemistry at the ETHZ on VG-TRIBRID for electron impact ionization (EI), Varian IonSpec Spectrometer for electrospray ionization (ESI), or IonSpec Ultima Fourier Transform Mass Spectrometer for matrix-assisted laser desorption/ionization (MALDI) and are reported as (m/z). High resolution MS/MS (Quadrupole-time-Of-Flight) were obtained on a Bruker Maxis 1 or 2.

Optical Rotation: Specific rotations [α]T were measured on a Jasco P-2000 digital polarimeter at the sodium D line with a 10 cm cell length. Data are reported as follows: [α]T in parentheses concentration (c = 1.00 corresponds to 10.0 mg/mL), and solvent. The temperature (T) at which the measurement was made is given as the superscript number (°C).

[3] Wilfred L. F. Armarego, C. C. *Purification of Laboratory Chemicals 6th Edition*; Elsevier Inc. , 2009.

3 Experimental Procedures

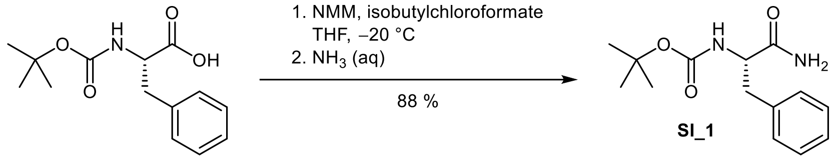

Boc-Phe-NH$_2$ SI_1: A 2.00 L round bottom flask was charged with 0.750 L THF and stirred. Boc-Phe-OH (30.0 g, 113 mmol, 1.00 equiv) was added and stirred until dissolved. The reaction mixture was cooled to –20 °C with a dry ice-acetone cooling bath. Sequentially, isobutylchloroformate (15.5 mL, 119 mmol, 1.05 equiv) and N-methylmorpholine(1) (13.2 mL, 158 mmol, 1.05 equiv) were added to the mixture, resulting in a white suspension. The mixture was stirred for 1 hour at –20 °C, then NH_3 (375 mL, 25 % aqueous) was added at once, and the reaction allowed to warm to r.t. Upon stirring for 1 hour at r.t. the reaction was quenched by the addition of sat. $NaHCO_3$, and extracted three times with EtOAc. The combined organic phases were washed twice with 1M HCl, then dried over Na_2SO_4, filtered and evaporated to a crude, which was purified by recrystallization from Hexane/EtOAc, yielding primary amide **SI_1** as a white solid (26.4 g, 99.9 mmol, 88%).

Note (1): Dried over sodium, then distilled in vacuo and stored in a schlenk flask over dry 4 Å MS.

Rf =0.40 (100% EtOAc, ninhydrin stain)

^{1}H NMR (500 MHz, $CDCl_3$) δ 7.30 (ddt, *J* = 7.4, 6.6, 1.1 Hz, 2H), 7.25 – 7.18 (m, 3H), 5.97 (s, 1H), 5.71 (s, 1H), 5.14 (d, *J* = 8.1 Hz, 1H), 4.41 – 4.37 (m, 1H), 3.06 (d, *J* = 6.4 Hz, 2H), 1.40 (s, 9H).

^{13}C NMR (126 MHz, $CDCl_3$) δ 173.9, 155.6, 136.8, 129.5, 128.8, 127.1, 80.4, 55.5, 38.6, 28.4.

Spectral data were consistent with literature.[4]

[4] Inman, M.; Dexter, H. L.; Moody, C. J. Total Synthesis of the Cyclic Dodecapeptides Wewakazole and Wewakazole B. *Org. Lett.* **2017**, *19* (13), 3454–3457. DOI: 10.1021/acs.orglett.7b01393.

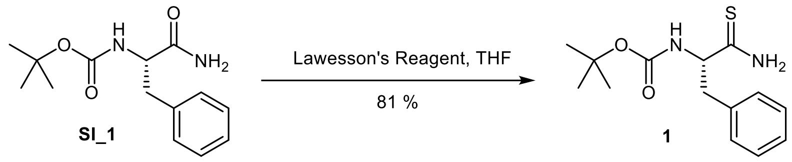

Thioamide 1: **SI_1** (27.2 g, 103 mmol, 1.00 equiv) was dissolved in 1.35 L THF was added, and the suspension stirred under N_2 until a clear solution was obtained. Lawesson's reagent (25.0 g, 61.8 mmol, 0.600 equiv) was added portionwise, and the resulting solution stirred overnight. The mixture was then diluted with EtOAc, sat. $NaHCO_3$ was added, and the biphasic mixture stirred for one hour. The mixture was transferred to a separatory funnel and the aqueous phase extracted three times with EtOAc. The combined organic phases were washed four times with sat. $NaHCO_3$, dried over Na_2SO_4, filtered and evaporated to a crude. Purification by column chromatography (dry load onto celite, 5-80% Ether/Pentane) gave the desired thioamide **1** as a white solid (23.4 g, 83.3 mmol, 81%).

Rf = 0.62 (50% EtOAc/Hexane, Ceric ammonium molybdate stain, UV)

^{1}H NMR (500 MHz, $CDCl_3$) δ 7.63 (s, 1H), 7.50 (s, 1H), 7.31 – 7.26 (m, 2H), 7.25 – 7.20 (m, 3H), 5.38 (d, J = 8.2 Hz, 1H), 4.67 (q, J = 7.5 Hz, 1H), 3.12 (s, 2H), 1.38 (s, 9H).

^{13}C NMR (126 MHz, $CDCl_3$) δ z, 208.6, 155.5, 136.6, 129.4, 128.8, 127.2, 80.7, 61.4, 41.9, 28.4.

Spectral data were consistent with literature.[5]

[5] Wahyudi, H.; Tantisantisom, W.; Liu, X.; Ramsey, D. M.; Singh, E. K.; McAlpine, S. R. Synthesis, Structure–Activity Analysis, and Biological Evaluation of Sanguinamide B Analogues. *J. Org. Chem.* **2012**, *77* (23), 10596–10616. DOI: 10.1021/jo3017499.

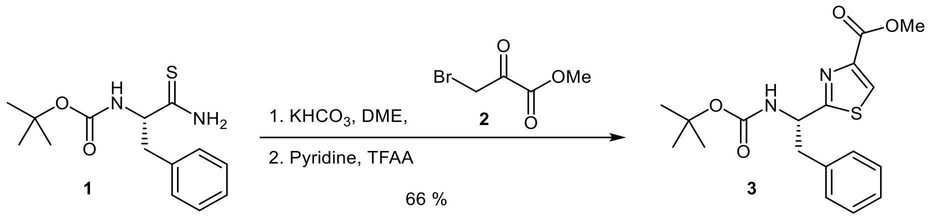

Thiazole 3: Conditions omptimized based on literature[6]. Thioamide **1** (1.00 g, 3.57 mmol, 1.00 equiv.) was added to a 250 mL round bottom flask equipped with a stirring bar and 30.0 mL dry DME was added, and the suspension stirred until a clear solution was obtained. $KHCO_3$ (2.86 g, 28.5 mmol, 8.00 equiv.) was added and stirred vigorously for 5 minutes (750 rpm). In order to dissipate heat during the reaction,[(1)] the flask was placed into a room temperature water bath. Dropwise, bromomethylpyruvate **2** (1.90 mL, 17.8 mmol, 5.00 equiv) was added during ca. 10 seconds, and the resulting solution stirred for 30 seconds at r.t. then ice was added to the water bath and stirring continued for further 30 seconds. A precooled (ice-water bath) mixture of TFAA (1.98 mL, 14.3 mmol, 4.00 equiv.) and Pyridine (2.59 mL, 32.1 mmol, 9.00 equiv) in 5.60 mL DME was added dropwise during 10 seconds, and the resulting mixture stirred for 30 minutes at 0 °C. The reaction was poured into a separatory funnel containing water, and the contents of the flask thoroughly rinsed with EtOAc. The aqueous phase was extracted three times with EtOAc, the combined organic phases washed with 5% citric acid solution, sat. $NaHCO_3$ solution and brine, then dried over Na_2SO_4, filtered and evaporated to a crude. The crude was purified by column chromatography (dry load onto celite, 5-30% EtOAc/Hexane) yielding the Thiazole **3** as an off-white solid (857 mg, 2.37 mmol, 66 %).

Note (1): Temperature control during the addition is important to prevent epimerization (as documented in the literature. For discussion of Mechanism of epimerization, consult [7]

Rf = 0.69 (50% EtOAc/Hexanes, ninhydrin stain)

^{1}H NMR (500 MHz, $CDCl_3$) δ 8.06 (s, 1H), 7.32 – 7.19 (m, 3H), 7.12 – 7.06 (m, 2H), 5.37 – 5.19 (m, 2H), 3.96 (s, 3H), 3.38 – 3.22 (m, 2H), 1.39 (s, 9H).

^{13}C NMR (126 MHz, $CDCl_3$) δ 173.4, 162.0, 155.1, 147.1, 136.3, 129.5, 128.8, 127.6, 127.2, 80.5, 54.0, 52.6, 41.7, 28.4.

HRMS (ESI) m/z calculated for C18H22N2NaO4S $[M+H]^+$: 385.1192, found 385.1187

FTIR (thin film) 2977, 1716, 1486, 1455, 1392, 1244, 1166, 1022, 991, 856, 754, 701

O.R. $[a]_D^{25}$ = -14.4 (c = 0.82, $CHCl_3$)

Spectral data were consistent with literature.[8]

[6] Bredenkamp, M. W.; Holzapfel, C. W.; van Zyl, W. J. The Chiral Synthesis of Thiazole Amino Acid Enantiomers. *Synth. Commun.* **1990**, *20* (15), 2235–2249. DOI: 10.1080/00397919008053164.
[7] Holzapfel, C. W.; Pettit, G. R. Antineoplastic agents. Par 108. Structural biochemistry. Part 23. Synthesis of the dolastatin thiazole amino acid component (gln)Thz. *J. Org. Chem.* **1985**, *50* (13), 2323–2327. DOI: 10.1021/jo00213a024.
[8] Liu, Y.; He, P.; Zhang, Y.; Zhang, X.; Liu, J.; Du, Y. One-Pot Enantiomeric Synthesis of Thiazole-Containing Amino Acids: Total Synthesis of Venturamides A and B. Ibid.**2018**, *83* (7), 3897–3905. DOI: 10.1021/acs.joc.8b00244.

Optimization of Hantzsch' Thiazole Synthesis:

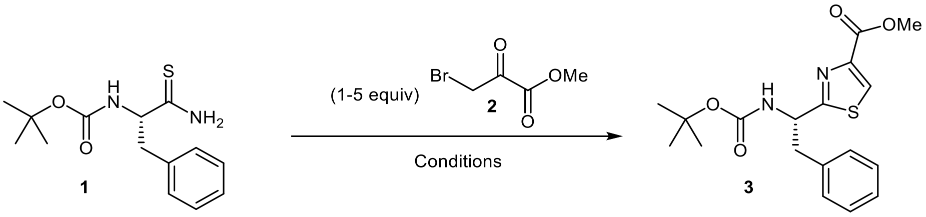

Entry	Conditions	Yield	ee
1[a]	$CaCO_3$/EtOH, –20 °C to r.t.	92%	40%
2[b]	1.$KHCO_3$, DME, 4h, 0 °C<br>2. TFAA, pyridine	98%	6%
3[b]	1.$KHCO_3$, DME, 3h, –20 °C<br>2. TFAA, pyridine	99%	70%
4[b]	1.$KHCO_3$, DME, 5 min r.t.<br>2. TFAA, pyridine	67%	84%
5[b]	1.$KHCO_3$, DME, 1 min r.t.<br>2. TFAA, pyridine	36%	>99%
6[c]	1.$KHCO_3$, DME, 1 min r.t.<br>2. TFAA, pyridine	67%	95%
7[c,d]	1.$KHCO_3$, DME, 1 min r.t.<br>2. TFAA, pyridine	66%	90%

[a]1.1. equiv.; [b] 3.0 equiv.; [c]5.0 equiv. methyl bromopyruvate **2**; [d] 1.00 g starting material

Erosion of stereochemistry was assessed by analytical HPLC (Stationary Phase: Chiral AM-R; Mobile Phase: isocractic run with 40% water in acetonitrile, UV-detector at 236 nm). A racemic sample was prepared by combining equal amounts of L, and D-phenylalanine derived thioamides **1**, and subsequent Hantsch thiazole synthesis as described above. D-Thiazole **3** was prepared analogous to L-thiazole **3**, using Boc-D-phenylalanine as starting material.

HPLC trace of racemic thiazole **3**:

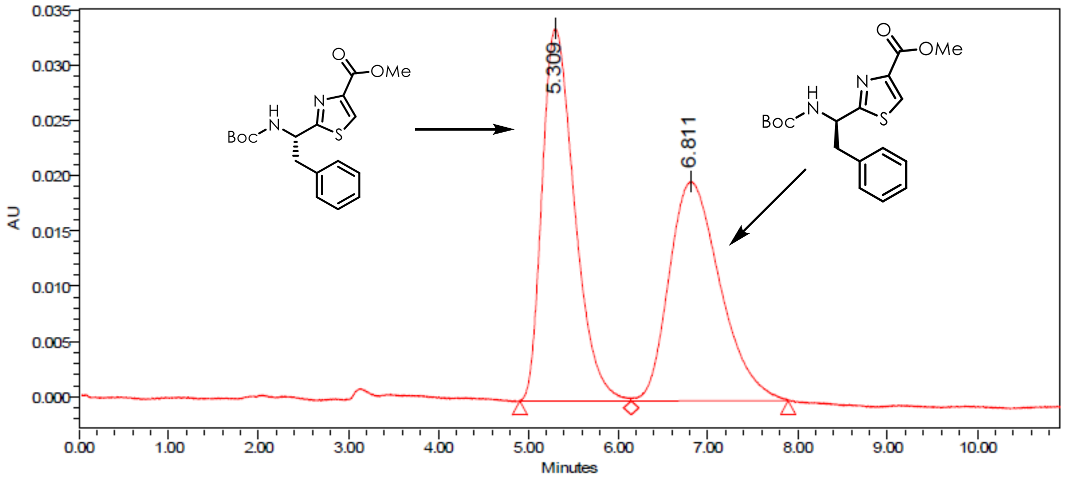

Peak Results

	RT	Area	% Area
1	5.309	844018	51.87
2	6.811	783098	48.13

HPLC-trace of S-thiazole **3**, with 90%ee:

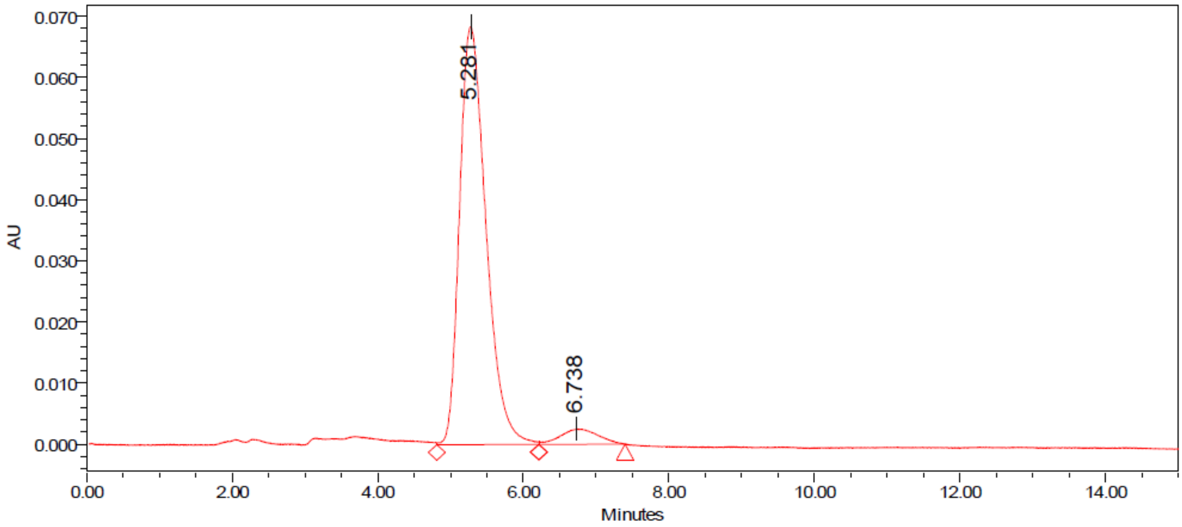

Peak Results

	RT	Area	% Area
1	5.281	1726886	94.99
2	6.738	91148	5.01

HPLC-trace of R-thiazole **3**, with 87 %ee:

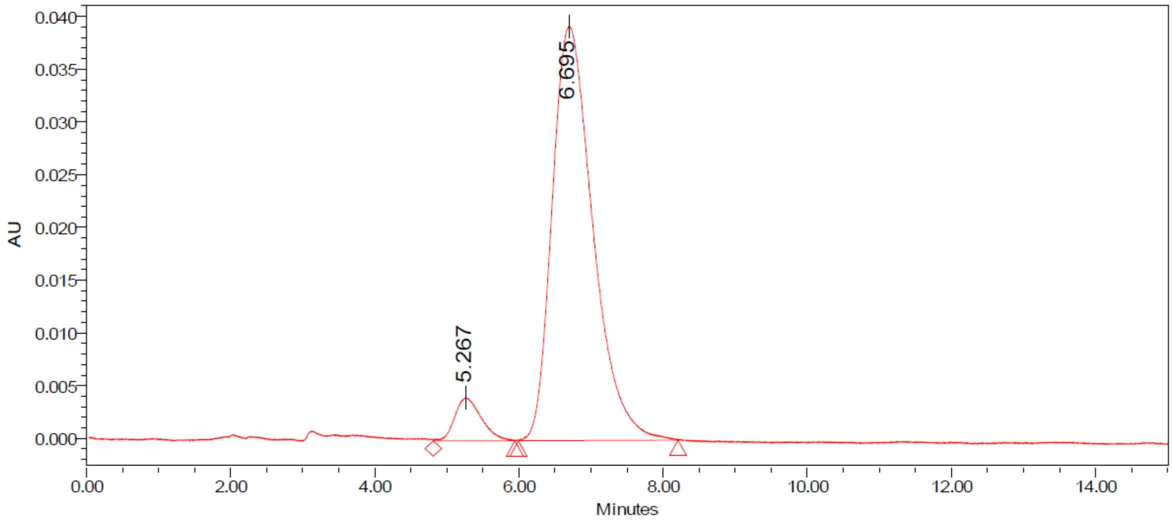

Peak Results

	RT	Area	% Area
1	5.267	104500	6.34
2	6.695	1543043	93.66

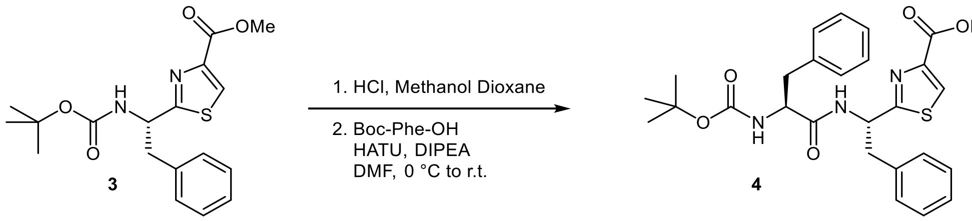

Dipeptide 4: Thiazole **3** (1190 mg, 3.28 mmol, 1.00 equiv) was dissolved in a mixture of 9.30 mL methanol and 21.0 mL 1,4-dioxane and stirred. Slowly, HCl (4 M in 1,4-dioxane, 12.3 mL, 49.3 mmol, 15.0 equiv) was added to the solution (entire addition took ca. 2 minutes). The reaction was stirred for two hours, the volatiles were then removed under reduced pressure, azeotroped once with EtOAc to remove residual HCl and the resulting crude solid redissolved in 26.0 mL DMF and cooled using an ice-water bath. Sequentially, Boc-Phe-OH (1.05 g, 3.94 mmol, 1.20 equiv), DIPEA(1) (2.29 mL, 13.1 mmol, 4.00 equiv) and HATU (1.74 g, 4.60 mmol, 1.40 equiv) were added to the flask. The reaction was stirred at 0 °C for one hour, then quenched by pouring into water/EtOAc. The layers were separated, and the aqueous phase extracted twice with EtOAc. The combined organic phases were washed three times with 5% LiCl solution and once with brine. The organic phase was dried with Na_2SO_4, filtered through cotton and evaporated to a crude, which was purified by column chromatography (dry load onto celite, 0.1-1.5% MeOH in CH_2Cl_2, product starts eluting at 1% MeOH) to give desired product **4** as a cream colored solid (1.31 g, 2.58 mmol, 79% over two steps).

Note (1): DIPEA was freshly distilled from CaH_2 under Nitrogen atmosphere before use.

Note (2): In case not enatiopure thiazole was used, column chromatography was not possible to separate the resulting diastereomers, however a single recrystallization (boiling in Toluene/EtOAc, ca. 20:1, then stored in the freezer overnight) gave diastereomerically pure product from 80 %ee Thiazole. The procedure above was carried out with >95 %ee SM.

Note (3): Epimerization of the stereogenic center α to the thiazole (conveniently monitored by ^{1}H-NMR analysis) was never observed in subsequent transformations.

Rf = 0.45 (40% EtOAc/Hexanes, UV-active, brown stain with ninhydrin)

^{1}H NMR (500 MHz, $CDCl_3$) δ 8.01 (s, 1H), 7.25 – 7.15 (m, 6H), 7.11 (d, *J* = 7.4 Hz, 2H), 6.98 (d, *J* = 6.6 Hz, 2H), 6.67 (d, *J* = 8.2 Hz, 1H), 5.52 (q, *J* = 7.4 Hz, 1H), 4.94 – 4.84 (m, 1H), 4.33 (s, 1H), 3.95 (s, 3H), 3.33 – 3.18 (m, 2H), 2.99 (d, *J* = 7.1 Hz, 2H), 1.37 (s, 9H).

^{13}C NMR (126 MHz, $CDCl_3$) δ 171.4, 170.9, 161.8, 155.3, 147.0, 136.4, 136.0, 129.4, 129.4, 128.8, 128.7, 127.7, 127.2, 127.1, 55.9, 52.6, 52.3, 41.2, 38.1, 29.8, 28.3.

HRMS (ESI) m/z calculated for C27H32N3O5S $[M+H]^+$: 510.2057, found 510.2056

FTIR (neat) 3288, 2978, 1658, 1497, 1367, 1241, 1167, 752, 699

O.R. $[\alpha]_D^{25}$ = -27.9 (c = 0.35, $CHCl_3$)

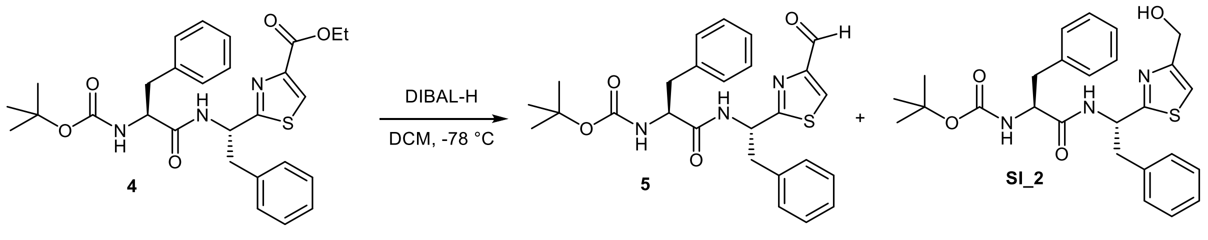

Aldehyde 5: Dipeptide **4** (1.29 g, 2.53 mmol, 1.00 equiv) was dissolved in 26.0 mL dry DCM and cooled to –78 °C using an acetone-dry ice bath. DIBAL-H (1M in DCM, 8.60 mL, 8.60 mmol, 3.40 equiv) was added dropwise during 2.5 hours via syringe pump. Upon complete addition, the reaction was stirred for one hour then quenched by the addition of 2.00 mL of Methanol during one hour via syringe pump. The reaction was warmed to r.t., diluted with Et_2O and stirred with sat. Rochelle solution for two hours (until good phase separation was visible). The phases were separated, the aqueous phase was extracted three times with Et_2O and the combined organic phases dried over Na_2SO_4, filtered through cotton and evaporated to a crude. Purification by column chromatography (dry load onto celite, 0.1-5% MeOH/ CH_2Cl_2 gradient. Product elutes at ca. 1.5-2% MeOH, overreduced alcohol at 3-5% MeOH) yields the aldehyde **5** as a cream colored solid (0.870 g, 1.82 mmol, 72%). And alcohol **SI_2** (217 mg, 0.46 mmol, 18%) as a white foam.

Analytical data for Aldehyde **5**:

Rf = 0.63 (60 % EtOAc/Hexane, $KMnO_4$ or ninhydrin stain)

^{1}H NMR (400 MHz, $CDCl_3$) δ 9.97 (s, 1H), 8.01 (s, 1H), 7.25 – 7.16 (m, 6H), 7.16 – 7.10 (m, 2H), 7.04 – 6.95 (m, 2H), 6.65 (d, *J* = 8.1 Hz, 1H), 5.52 (q, *J* = 7.2 Hz, 1H), 4.89 (s, 1H), 4.33 (d, *J* = 8.4 Hz, 1H), 3.30 (dd, *J* = 13.6, 6.6 Hz, 1H), 3.19 (dd, *J* = 13.6, 7.3 Hz, 1H), 2.98 (m, 2H), 1.38 (s, 9H).

^{13}C NMR (126 MHz, $CDCl_3$) δ 184.6, 171.8, 171.0, 155.4, 154.8, 136.4, 135.9, 129.5, 129.4, 128.9, 128.8, 127.9, 127.3, 127.2, 80.4, 56.0, 52.3, 41.3, 38.2, 28.4.

HRMS (ESI) m/z calculated for C26H29N3NaO4S $[M+Na]^+$: calculated: 502.1771, found: 502.1763

FTIR (thin film): 328, 3029, 2977, 2927, 2854, 1698, 1658, 1521, 1497, 1248, 1169, 1050, 1031, 856, 750, 699

O.R. $[\alpha]_D^{28}$ = -18.2 (c = 0.5, $CHCl_3$)

Analytical Data for alcohol **SI_2**:

Rf = 0.26 (60 % EtOAc/Hexane, $KMnO_4$ or ninhydrin stain) overreduced alcohol **SI_2**

^{1}H NMR (500 MHz, $CDCl_3$) δ 7.25 – 7.15 (m, 6H), 7.14 – 7.09 (m, 2H), 7.03 (t, *J* = 0.9 Hz, 1H), 6.97 (d, *J* = 6.7 Hz, 2H), 6.65 (s, 1H), 5.49 (q, *J* = 7.2 Hz, 1H), 4.93 (s, 1H), 4.71 (s, 2H), 4.33 (s, 1H), 3.22 (dd, *J* = 13.6, 6.6 Hz, 1H), 3.16 (dd, *J* = 13.6, 7.1 Hz, 1H), 3.01 (d, *J* = 6.9 Hz, 2H), 2.33 (s, 1H), 1.44 – 1.35 (m, 9H).

^{13}C NMR (126 MHz, $CDCl_3$) δ 170.8, 170.4, 156.2, 155.4, 136.6, 136.3, 129.6, 129.4, 128.8, 128.6, 127.1, 127.1, 114.6, 80.4, 61.1, 55.9, 52.3, 41.8, 38.3, 28.4.

HRMS (ESI) m/z calculated for C26H32N3O4S $[M+H]^+$: 482.2108, found: 482.2100

FTIR (thin film) 3288, 3063, 3029. 2929, 1660, 1604, 1520, 1497, 1455, 1392, 1249, 1166, 1029, 910, 856, 731

O.R. $[\alpha]_D^{27}$ = -25.5 (c = 1.0, $CHCl_3$)

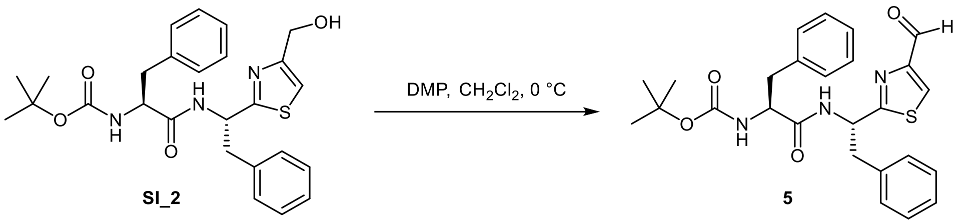

Usually, multiple fractions of overreduced alcohol were combined, and reoxidised: Alcohol **SI_2** (2.10 g, 4.36 mmol, 1.00 equiv) was dissolved in 30.0 mL CH_2Cl_2 and cooled to 0 °C using an ice-water bath. DMP (2.77 g, 6.54 mmol, 1.50 equiv.) was added at once, and the reaction stirred while slowly allowed to warm to r.t. The reaction was found to be complete by TLC after 2 hours and quenched by the addition of 1:1 sat. $NaHCO_3$ and sat. $Na_2S_2O_3$, stirred for 5 minutes and then extracted three times with Et_2O. The combined organic phases were then dried over Na_2SO_4, filtered and concentrated in vacuo. The crude was purified by column chromatography (Dry load with celite, 0.1-3% MeOH in CH_2Cl_2, product starts eluting at 1.5-2%) to yield aldehyde **5** as cream colored solid (1.87 g, 3.89 mmol, 89%). Analytical data identical to above.

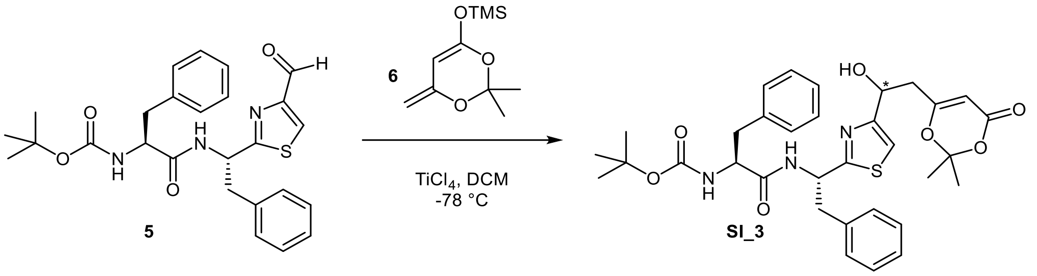

Mukaiyama-adduct SI_3: Aldehyde **5** (1180 mg, 2.46 mmol, 1.00 equiv) was dissolved in 42.0 mL CH_2Cl_2 and cooled to –78 °C. $TiCl_4$ (1M solution in CH_2Cl_2, 2.46 mL, 2.46 mmol, 1.00 equiv.) was added during 10 minutes via syringe pump, and the resulting solution stirred at –78 °C for 20 minutes. Silyl-ketene-acetal(1) (553 mg, 2.58 mmol, 1.05 equiv) was added as a solution in 12.0 mL CH_2Cl_2 during one hour via syringe pump. The reaction was continued for three hours upon complete addition at –78 °C, then quenched by a rapid injection of sat. $NaHCO_3$ solution at –78 °C. The mixture was warmed to r.t., the phases separated and the aqueus phase extracted three times with CH_2Cl_2. The combined organic phases were dried over Na_2SO_4, filtered through cotton and evaporated to a crude. The crude was purified by column chromatography (dry load, 5x weight of crude in celite, 1-3% MeOH/CH_2Cl_2, product elutes at ca. 3% MeOH/CH_2Cl_2) yielded desired Mukaiyama-adduct **SI_3** as a slightly yellow foam (1390 mg, 2.23 mmol, 91%)(2).

Note (1): Prepared according to our earlier Publication,[9] and stored in a schlenk flask under nitrogen atmosphere in the freezer.

Note (2): the product is formed as an inconsequential, inseparable mixture of diastereomers, ca. 1:1 according to 1H NMR. In the spectra below, signals denoted with an asterisk (*) correspond to the different diastereomers.

Rf = 0.19 (50% EtOAc/Hexane, UV active, $KMnO_4$/Ninhydrin stain)

^{1}H NMR (500 MHz, $CDCl_3$) δ 7.25 – 7.21 (m, 2H), 7.20 (tq, *J* = 4.1, 2.4, 1.8 Hz, 4H), 7.15 (m 2H) 7.05* (s, 0.5H), 7.04* (s, 0.5H)– 7.02 (m, 1H), 6.94 (d, *J* = 6.0 Hz, 2H), 6.65 (d, *J* = 7.8 Hz, 1H), 5.49 (q, *J* = 6.5, 5.7 Hz, 1H), 5.34* (s, 0.5H), 5.33* (s, 0.5H), 5.04 (dd, *J* = 8.7, 4.3 Hz, 1H), 4.90 (s, 1H), 4.33 (s, 1H), 3.19 (qd, *J* = 13.7, 6.7 Hz, 2H), 3.04 – 3.00 (m, 2H), 2.78 (dddd, *J* = 14.6, 13.7, 4.3, 0.9 Hz, 1H), 2.73 – 2.62 (m, 1fH), 1.70* (d, *J* = 0.7 Hz, 3H), 1.69* (s, 3H), 1.44 – 1.36 (s, 9H).

^{13}C NMR (126 MHz, $CDCl_3$) δ 170.8, 168.13*, 168.09, 161.00*, 160.97, 157.5*, 157.4, 155.3, 136.4, 135.92*, 135.91, 129.4, 129.3, 128.7, 128.6, 128.5, 127.1, 127.0, 114.2, 106.76*, 106.75, 95.61*, 95.59, 80.4, 67.6*, 67.5, 55.8, 52.2*, 52.1, 41.7*, 41.6, 41.5, 38.0, 28.3, 25.50*, 25.45, 24.75*, 24.70.

HRMS (ESI) m/z calculated for C33H40N3O7S $[M+H]^+$: 622.2581, found: 622.2568

FTIR (thin film): 3289, 3029, 2980, 2856, 1710, 1660, 1635, 1521, 1497, 1455, 1441, 1391, 1376, 1368 1275, 1168, 1080, 1049, 1015, 858, 808, 753

O.R. $[\alpha]_D^{28}$ = -20.2 (c = 1.0, $CHCl_3$)

[9] Fettes, A.; Carreira, E. M. Leucascandrolide A: Synthesis and Related Studies. Ibid.**2003**, *68* (24), 9274–9283. DOI: 10.1021/jo034964v.

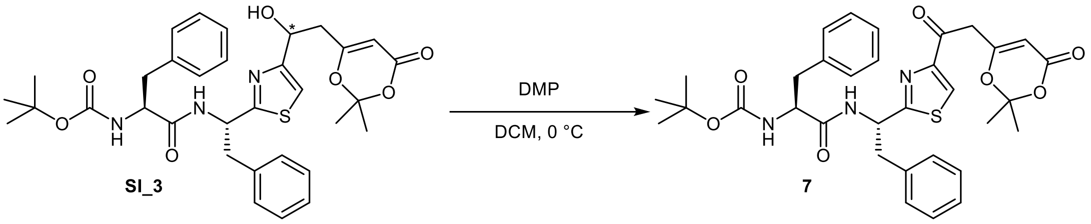

SI_3 → 7

Ketone 7: Mukajiama-adduct **SI_3** (1.30 g, 2.09 mmol, 1.00 equiv) was dissolved in 15.5 mL CH_2Cl_2 and cooled to 0 °C using an ice-water bath. DMP (1330 mg, 3.14 mmol, 1.50 equiv) was added at once, and the resulting solution stirred for two hours. The reaction was quenched by the simultaneous addition of sat. $Na_2S_2O_3$ and $NaHCO_3$. The mixture was stirred for 5 minutes, diluted with Et_2O, the phases separated and the aqueus phase extracted three times with more ether. The combined organic phases were dried over Na_2SO_4, filtered through cotton and evaporated to a crude. The crude was purified by column chromatography (dry load on celite, 0.1-2% MeOH/DCM, product starts eluting at 1.5%) to give a yellow foam (0.990 g, 1.60 mmol, 76%).

Rf = 0.55 (1:1 EtOAc/Hexane, Ninhydrin stain)

^{1}H NMR (400 MHz, $CDCl_3$) δ 8.04 (s, 1H), 7.26 – 7.18 (m, 6H), 7.17 – 7.09 (m, 2H), 7.01 – 6.93 (m, 3H), 6.68 (d, *J* = 8.1 Hz, 1H), 5.51 (q, *J* = 7.1 Hz, 1H), 4.93 (s, 1H), 4.33 (d, *J* = 7.5 Hz, 1H), 3.97 (d, *J* = 0.6 Hz, 2H), 3.26 (dd, *J* = 13.7, 6.5 Hz, 1H), 3.18 (dd, *J* = 13.7, 7.1 Hz, 1H), 3.02 (d, *J* = 6.9 Hz, 2H), 1.73 (s, 6H), 1.38 (s, 9H).

^{13}C NMR (101 MHz, $CDCl_3$) δ 188.0, 171.2, 171.0, 165.2, 161.0, 155.5, 153.3, 136.5, 135.7, 129.4, 129.4, 128.8, 128.8, 127.4, 127.1, 126.5, 107.4, 97.1, 80.6, 56.0, 52.3, 44.7, 41.3, 38.1, 28.4, 25.2, 25.1.

HRMS (ESI) m/z calculated for C33H38N3O7S $[M+H]^+$: 620.2425, found 620.2421

FTIR (thin film): 3340, 3279, 3025, 2927, 2854, 1720, 1682, 1664, 1519, 1455, 1391, 1368, 1165, 1015, 752, 697, 622

O.R. $[\alpha]_D^{27}$ = -18.2 (c = 1.0, $CHCl_3$)

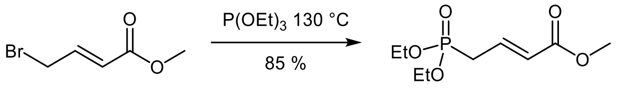

13

Methyl (E)-4-(diethoxyphosphoryl)but-2-enoate 13: Prepared according to a modified literature procedure:[10] Triethyl phosphite (21.0 mL, 122 mmol, 1.20 equiv) and methyl (E)-4-bromobut-2-enoate (12.0 mL, 102 mmol, 1.00 equiv) were added to a flask and set under N_2. The reaction was heated to 130 °C and stirred for 16 hours. The reaction was allowed to cool to ambient temperature and directly purified by column chromatography (40-100% EtOAc/Hexanes). Evaporation gave a clear oil that crystallized upon storage at –20 °C (20.4 g, 86.4 mmol, 85%).

Rf =0.27 (100% EtOAc, UV active, $KMnO_4$-stain)

^{1}H NMR (400 MHz, $CDCl_3$) δ 6.88 (dq, *J* = 15.4, 7.7 Hz, 1H), 5.96 (ddt, *J* = 15.6, 5.0, 1.4 Hz, 1H), 4.11 (dqt, *J* = 8.3, 7.2, 2.8 Hz, 4H), 3.73 (s, 3H), 2.74 (ddd, *J* = 22.9, 7.9, 1.4 Hz, 2H), 1.32 (t, *J* = 7.1 Hz, 6H).

^{13}C NMR (101 MHz, $CDCl_3$) δ 166.2 (d, *J* = 2.8 Hz), 137.9 (d, *J* = 11.1 Hz), 125.5 (d, *J* = 13.7 Hz), 62.4 (d, *J* = 6.6 Hz), 51.7, 30.8 (d, *J* = 138.8 Hz), 16.5 (d, *J* = 6.0 Hz).

^{31}P NMR (162 MHz, $CDCl_3$) δ 24.2.

[10] Isamu Shiina, Y. U., Takatsugu Murata, Kyohei Suzuki, Takayuki Tonoi Asymmetric Total Synthesis of (+)-Coprophilin. *Synthesis* **2018**, *50*, 1301–1306. DOI: 10.1055/s-0036-1591866.

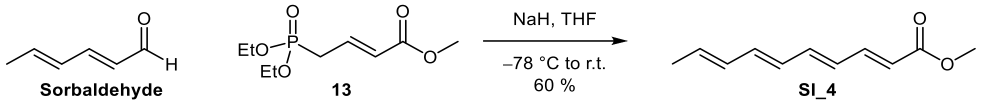

Methyl ester SI_4: Phosphonate **13** (0.630 mL, 3.00 mmol, 1.50 equiv.) was dissolved in 92.0 mL THF and cooled to –10 °C, using an acetone-dry ice bath. LiHMDS (3.30 mL, 3.30 mmol, 1.65 equiv. 1.0 M in THF) was added dropwise and the mixture stirred at –10 °C for 10 minutes. The reaction was cooled to –65 °C, HMPA (1.04 mL, 6.00 mmol, 3.00 equiv.) was added dropwise, and the reaction stirred for 10 minutes at –65 °C. The mixture was then cooled further to –78 °C and sorbaldehyde (0.210 mL, 2.00 mmol, 1.00 equiv.) was added as a solution in 3.00 mL THF. The reaction mixture stirred for 1.5 h at –78 °C, the cooling bath removed and the reaction stirred an additional 30 minutes at room temperature. The reaction mixture was quenched by addition of saturated aqueous NH_4Cl and the organics were extracted with diethyl ether. The organic layer was dried using $MgSO_4$ and concentrated before being purified by column chromatography (0-5% EtOAc/Hexanes) to give the desired product **SI_4** as a white solid (215 mg, 1.20 mmol, 60 % yield)[(1)].

Note (1): Similar products are known to be light-sensitive, so the product was stored in amber vials in the dark, regular manipulations (reaction, column chromatography, concentration on rotavap) were carried out with minimal exposure to light.

Rf =0.73 (20% EtOAc/Hexanes, UV-active, $KMnO_4$-stain)

^{1}H NMR (400 MHz, $CDCl_3$) δ 7.32 (dd, *J* = 15.3, 11.3 Hz, 1H), 6.56 (dd, *J* = 14.8, 11.0 Hz, 1H), 6.43 – 6.32 (m, 1H), 6.33 – 6.24 (m, 1H), 6.20 – 6.05 (m, 2H), 5.86 (dd, *J* = 14.8, 8.0 Hz, 2H), 3.75 (d, *J* = 2.6 Hz, 3H), 1.82 (dt, *J* = 7.1, 1.9 Hz, 3H).

^{13}C NMR (101 MHz, $CDCl_3$) δ 167.8, 145.0, 141.4, 137.8, 133.4, 131.7, 129.5, 129.2, 119.7, 51.6, 18.7.

Spectral data were consistent with literature.[11]

[11] Turner, C. I.; Paddon-Row, M. N.; Willis, A. C.; Sherburn, M. S. Double Diels−Alder Reactions of Linear Conjugated Tetraenes. *J. Org. Chem.* **2005**, *70* (4), 1154–1163. DOI: 10.1021/jo048108a.

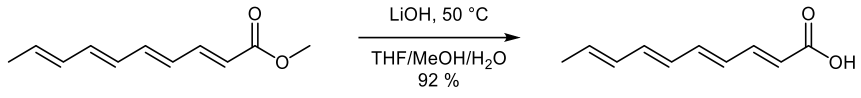

Tetraenoic acid scheme labels: **SI_4** → **14**

Tetraenoic acid 14: Methyl ester **SI_4** (0.210 g, 1.18 mmol, 1.00 equiv) was dissolved in a mixture of 8.00 mL THF and 6.00 mL methanol. Lithium hydroxide solution (1.47 mL, 5.89 mmol, 5.00 equiv, 4.00 M in water) was added and the resulting solution was heated to 50 °C for 30 minutes, then allowed to cool to ambient temperature. The mixture was quenched with a mixture of 2M HCl and brine (pH <2), and the resulting solution was extracted five times with CH_2Cl_2. The combined organic phases were dried over Na_2SO_4, filtered through cotton and evaporated to give desired tetraenoic acid **14** as a yellow solid (178 mg, 1.08 mmol, 92%),[(1)] which was used without further purification.

An analytical Sample for characterization was obtained by recrystallization (EtOAc/Hexane, crystallization at – 20 °C overnight).

Note (1): Similar products are known to be light-sensitive, so the product was stored in amber vials in the dark, regular manipulations (reaction, column chromatography, concentration on rotavap) were carried out with minimal exposure to light.

Rf = 0.04-0.33 (100% EtOAc, UV-active, $KMnO_4$-stain)

^{1}H NMR (500 MHz, DMSO) δ 12.14 (s, 1H), 7.21 (ddd, *J* = 15.2, 11.4, 0.7 Hz, 1H), 6.71 (ddd, *J* = 15.0, 11.1, 0.8 Hz, 1H), 6.42 (ddd, *J* = 18.9, 14.8, 11.0 Hz, 2H), 6.26 (ddt, *J* = 15.0, 11.1, 0.8 Hz, 1H), 6.22 – 6.13 (m, 1H), 5.93 – 5.87 (m, 1H), 5.85 (d, *J* = 15.0 Hz, 1H), 1.78 (dd, *J* = 7.0, 1.6 Hz, 3H).

^{13}C NMR (126 MHz, DMSO) δ 167.6, 144.2, 140.8, 137.3, 132.8, 131.7, 129.6, 129.4, 121.0, 18.3.

HRMS (ESI) m/z calculated for C10H12NaO2 $[M+Na]^+$: 187.0730, found: 187.0725

FTIR (thin film) 3020, 2919, 2847, 1684, 1618, 1594, 1424, 1311, 1263, 1161, 1009

Spectral data were consistent with literature.[12]

[12] Pini, E.; Bertacche, V.; Molinari, F.; Romano, D.; Gandolfi, R. Direct conversion of polyconjugated compounds into their corresponding carboxylic acids by Acetobacter aceti. *Tetrahedron* **2008**, *64* (37), 8638–8641. DOI: https://doi.org/10.1016/j.tet.2008.07.011.

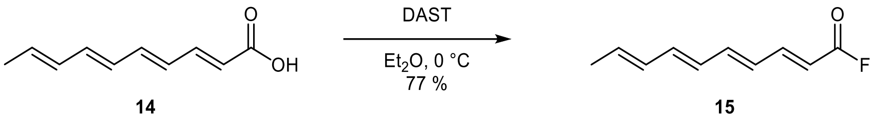

tetraenoyl fluoride 15: tetraenoic acid **14** (0.800 g, 4.87 mmol, 1.00 equiv) was suspended in 30.0 mL diethyl ether and cooled to 0 °C. DAST(1) (0.800 mL, 6.53 mmol, 1.34 equiv) was added dropwise, resulting in a yellow solution. The reaction was stirred at 0 °C in the dark for one hour, then quenched by the addition of sat. $NaHCO_3$. The phases were separated, and the aqueous phase extracted three times with diethyl ether. The combined organic phases were dried over Na_2SO_4, filtered and evaporated. The crude was purified by column chromatography (1-5-10% Et_2O/Pentane) to give the desired product **16** as a pale yellow solid(2) (0.620 g, 3.73 mmol, 77%).

Note (1): DAST was distilled in vacuo prior to usage, following distillation it was stored in the dark at – 20 °C *in a plastic vial.*

Note (2): Due to limited stability the product was stored as a stock solution with 10mg/mL in PhH at – 20 °C in the dark. Similar products are known to be light-sensitive, regular manipulations (reaction, column chromatography, concentration on rotavap) were carried out with minimal exposure to light.

Rf =0.73 (20% EtOAc/Hexanes, UV active, $KMnO_4$-stain)

^{1}H NMR (300 MHz, $CDCl_3$) δ 7.5 – 7.4 (m, 1H), 6.8 – 6.6 (m, 1H), 6.5 (dd, *J* = 14.9, 10.5 Hz, 1H), 6.3 (dd, *J* = 14.8, 11.4 Hz, 1H), 6.3 – 6.1 (m, 2H), 5.9 (dq, *J* = 14.0, 6.8 Hz, 1H), 5.8 (dd, *J* = 15.2, 8.0 Hz, 1H), 1.9 – 1.8 (m, 3H).

^{13}C NMR (126 MHz, $CDCl_3$) δ 157.5 (d, *J* = 336.0 Hz), 151.3 (d, *J* = 6.2 Hz), 144.9, 140.2, 135.2, 131.6, 129.0, 128.1, 113.2 (d, *J* = 66.9 Hz), 18.8.

^{19}F NMR (282 MHz, $CDCl_3$) δ 23.8.

HRMS (EI) m/z calculated for C10H11OF $[M]^{\bullet+}$: 166.0794, found 166.0788

FTIR (thin film) 3023, 2911, 1781, 1615, 1593, 1437, 1212, 1150, 1011, 956, 870, 785, 662

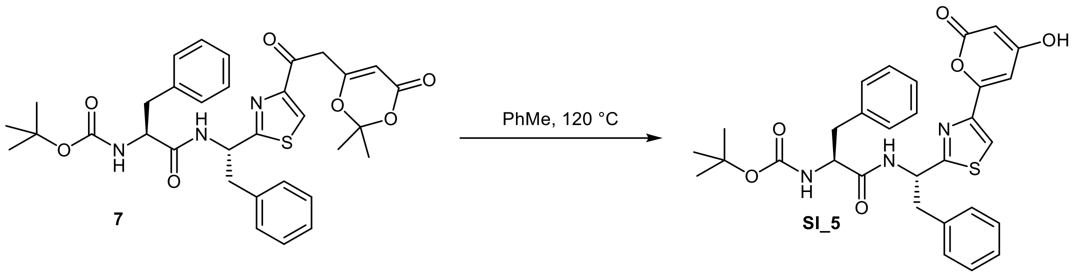

Pyrone SI_5: Starting Material (10.0 mg, 0.016 mmol, 1.00 equiv) was dried by azeotropic distillation (3x) with toluene, then dissolved in 2.00 mL dry toluene and stirred under N_2. The flask was then immersed in a preheated Sand bath (145 °C) and stirred for 45 minutes. The flask was then cooled to r.t., and volatiles removed under a stream of N_2. Ca. 1 mL CH_2Cl_2 was added, and the volatiles again removed under N_2. This process was repeated three times in total, giving product **SI_5** as a pale yellow solid, which was used without further purification.

For analytical purposes, the reaction product could be purified by column chromatography (Dry load onto celite, CH_2Cl_2/MeOH, 1-5%) to yield desired product as a tan solid.

Rf = 0.25 (5% MeOH in CH_2Cl_2, UV active, stains with $KMnO_4$)

^{1}H-NMR (500 MHz, DMSO-d$_6$) δ 11.91 (s, 1H), 8.83 (d, *J* = 8.6 Hz, 1H), 8.11 (s, 1H), 7.42 – 7.06 (m, 11H), 6.89 (d, *J* = 8.4 Hz, 1H), 6.74 (s, 1H), 5.39 (d, *J* = 2.3 Hz, 2H), 4.14 (d, *J* = 52.4 Hz, 1H), 3.43 – 3.36 (m, 1H), 3.27 – 3.13 (m, 1H), 2.85 (t, *J* = 6.8 Hz, 1H), 2.69 (s, 1H), 1.29 (s, 9H).

^{13}C-NMR (126 MHz, DMSO-d$_6$) δ 174.2, 171.7, 170.3, 162.7, 155.7, 155.1, 146.1, 137.8, 137.4, 129.3, 129.1, 128.2, 128.0, 126.5, 126.2, 120.7, 98.8, 90.0, 78.1, 55.8, 52.3, 40.0*, 37.4, 28.1.

*Extracted from HSQC spectrum, due to overlap with DMSO-d6

FTIR (neat) 1663, 1514, 1368, 1276, 1260, 1166, 764, 750, 699

HRMS (ESI) m/z calculated for C30H31N3NaO6S $[M+Na]^+$: 584.1826, found 584.1824

O.R. $[\alpha]_D^{27}$ = -3.2 (c = 0.50, 9:1 CH_2Cl_2/MeOH)

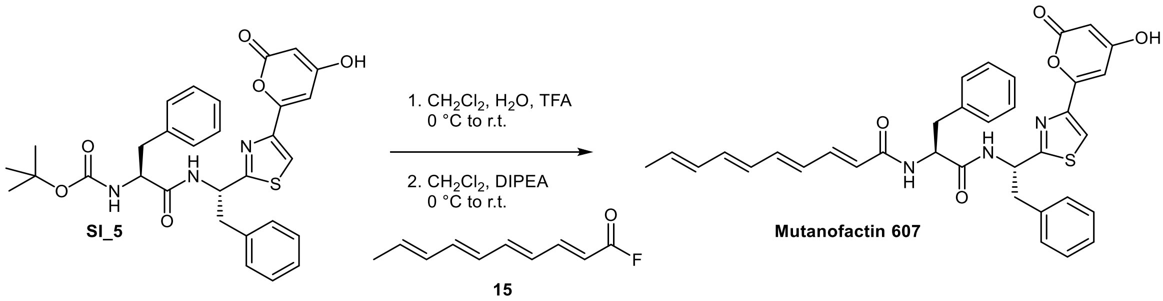

Mutanofactin 607: Pyrone **SI_5** (assumed quantitative, 0.016 mmol, 1.00 equiv) was suspended in 0.30 mL CH_2Cl_2 and cooled to 0 °C using an ice-water bath. Sequentially, 1 drop of distilled water and TFA (0.150 mL, 1.94 mmol, 120 equiv) were added resulting in a clear solution. The mixture was stirred for 2 hours, then the volatiles removed under a stream of nitrogen. Ca. 1 mL CH_2Cl_2 was added, and the volatiles again removed, this process was repeated three times in total. The residue was dried under high vacuum for 5 minutes, then redissolved in 0.30 mL DMF and cooled to 0 °C using an ice-water bath. Sequentially, DIPEA (14.0 microliters, 0.081 mmol, 5.00 equiv.) and acyl fluoride **15** (5.4 mg, 0.032 mmol, 2.00 equiv. as a solution in 0.10 mL DMF) were added, and the resulting solution stirred overnight, while slowly warming to room temperature. To this mixture was added 2-3 drops of methanol, followed by 2-3 drops of formic acid. DMF was added to homogenize the mixture, then the solution filtered and directly purified by preparative HPLC, to give Mutanofactin 607 as a white powder upon lyophilization (3.7 mg, 0.006 mmol, 38% over 3 steps).

Note: Similar products are known to be light-sensitive, so the product was stored in amber vials in the dark, regular maniulations (reaction, lyophilization) were carried out with minimal exposure to light.

Retention Time: Gradient: 60% Water for 1 minute, then linear decrease to 20% Water over 18 minutes. Product elutes at 10.1 min, based on MS and UV_{330}, collected from 9.4-10.5 minutes.

^{1}H NMR (500 MHz, DMSO) δ 8.99 (dd, *J* = 8.1, 4.9 Hz, 1H), 8.24 (d, *J* = 8.6 Hz, 1H), 8.04 (d, *J* = 2.8 Hz, 1H), 7.30 – 7.11 (m, 11H), 7.07 – 6.98 (m, 1H), 6.65 (d, *J* = 2.1 Hz, 1H), 6.60 (dd, *J* = 14.8, 10.9 Hz, 1H), 6.39 (dd, *J* = 15.1, 10.7 Hz, 2H) 6.25 (dd, *J* = 14.9, 10.9 Hz, 1H), 6.21 – 6.12 (m, 1H), 6.03 (dd, *J* = 15.0, 9.0 Hz, 1H), 5.84 (dq, *J* = 14.1, 7.0 Hz, 1H), 5.37 – 5.28 (m, 1H), 5.21 (s, 1H), 4.65 (ddd, *J* = 9.7, 8.5, 4.9 Hz, 1H), 3.38 (d, *J* = 5.4 Hz, 1H), 3.16 (dd, *J* = 13.9, 9.4 Hz, 1H), 2.96 (dd, *J* = 13.8, 5.0 Hz, 1H), 2.78 – 2.68 (m, 1H), 1.77 (dd, *J* = 6.9, 1.6 Hz, 3H).

^{13}C NMR (151 MHz, DMSO) δ 173.8, 172.0, 171.2, 164.9, 163.2, 155.1, 146.5, 139.4, 139.1, 137.7, 137.4, 136.1, 132.0, 131.8, 129.9, 129.8, 129.3, 129.1, 128.2, 128.0, 126.4, 126.3, 124.1, 120.0, 100.3, 89.3, 53.9, 52.4, 39.9*, 37.6, 18.3.

HRMS (ESI) m/z calculated for C35H34N3O5S $[M+H]^+$:608.2214, found 608.2209

FTIR (thin film) 3292, 3263, 2925, 1666, 1645, 1619, 1596, 1536, 1422, 1236, 1160, 1082, 900, 817, 696

O.R. $[\alpha]_D^{27}$ = -55.6 (c = 0.10, MeOH)

Comparison of Synthetic and isolated Mutanofactin 607

High Resolution MS/MS of synthetic mutanofactin 607 and proposed fragment structures

Acquisition Parameter

Method:	ETH_HyStar_HPLC_QTOF_POS_LowMass_Loop-AS.m			Acquisition Date:	
File Name:	D:\Data\bmax0157xx\BMAX015742.d			Operator:	
Source Type	ESI	Ion Polarity	Positive	Set Nebulizer	0.4 Bar
Focus	Active	Set Capillary	4500 V	Set Dry Heater	200 °C
Scan Begin	50 m/z	Set End Plate Offset	-500 V	Set Dry Gas	4.0 l/min
Scan End	1300 m/z	Set Collision Cell RF	200.0 Vpp	Set Divert Valve	Source

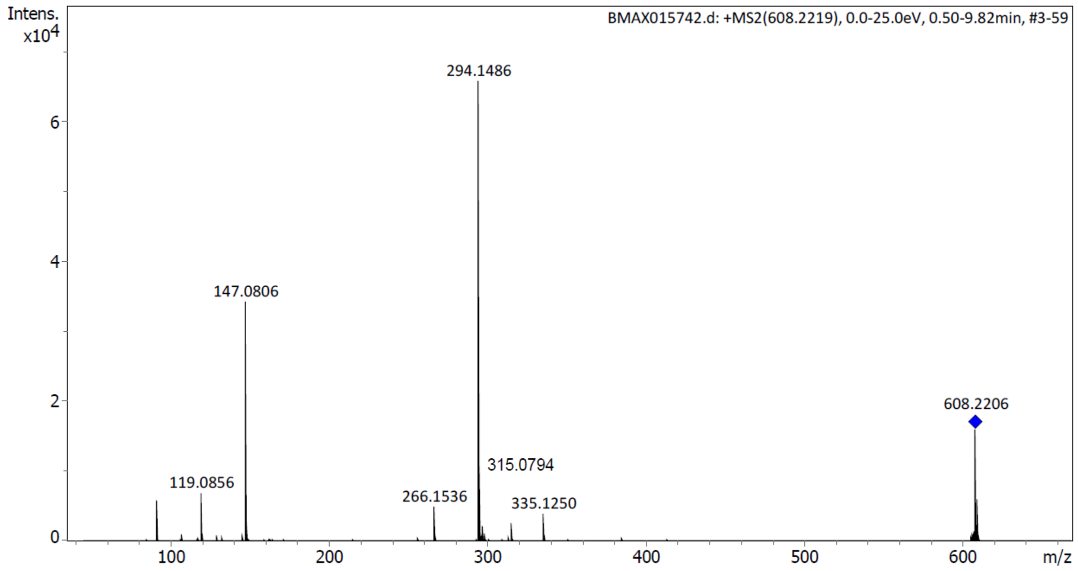

Comparison with Isolation MS-MS studies, reproduced from [13]:

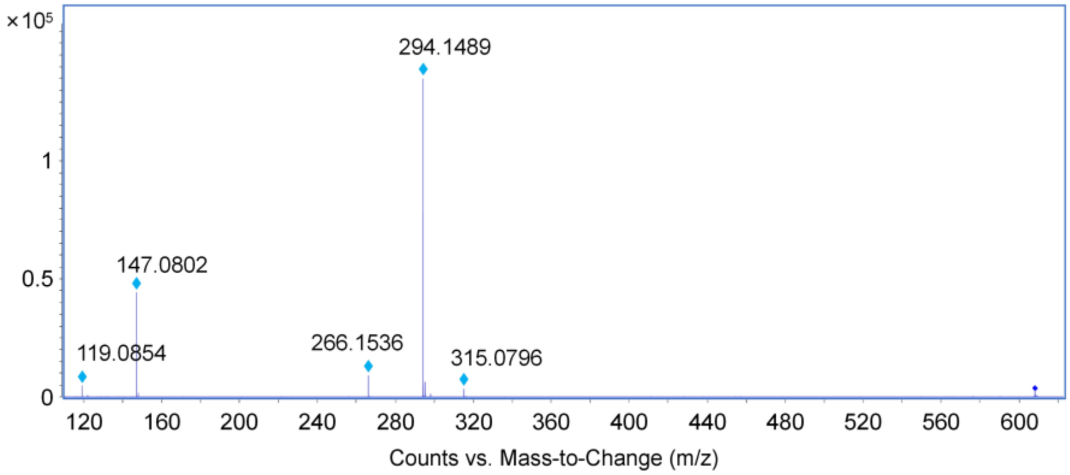

HR-MS/MS fragmentation pattern of **4**. Fragmentation was acquired with collision energy of 15 V.

[13] Li, Z.-R.; Sun, J.; Du, Y.; Pan, A.; Zeng, L.; Maboudian, R.; Burne, R. A.; Qian, P.-Y.; Zhang, W. Mutanofactin promotes adhesion and biofilm formation of cariogenic *Streptococcus mutans*. *Nat. Chem. Biol.* **2021**, *17* (5), 576–584. DOI: 10.1038/s41589-021-00745-2.

Proposal for Observed Fragments in MS-MS:

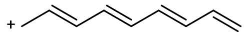

m/z
calculated: 119.0855
found: 119.0856

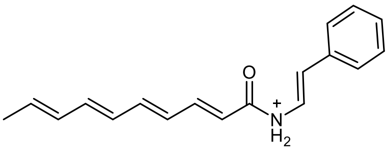

m/z
calculated: 266.1539
found: 266.1536

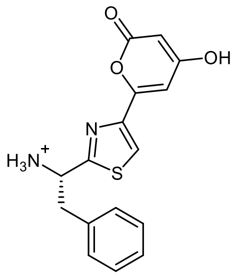

m/z
calculated: 315.0798
found: 315.0794

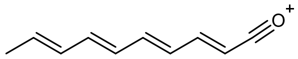

m/z
calculated: 147.0804
found: 147.0806

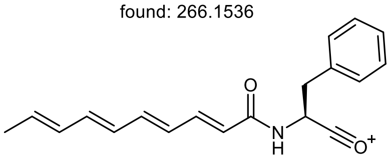

m/z
calculated: 294.1489
found: 294.1486

NMR comparison between isolated material[14] and synthetic Muf-607:

^{1}H-NMR reproduced from Zhang *et al*:

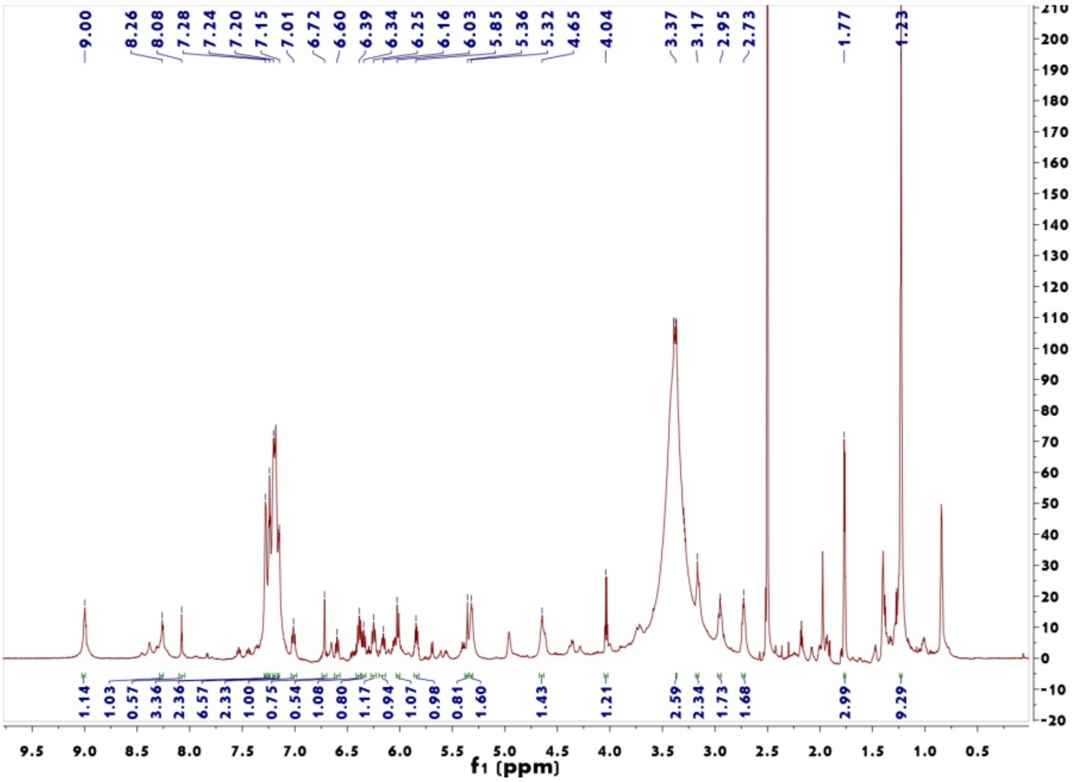

^{1}H NMR of Synthetic Material:

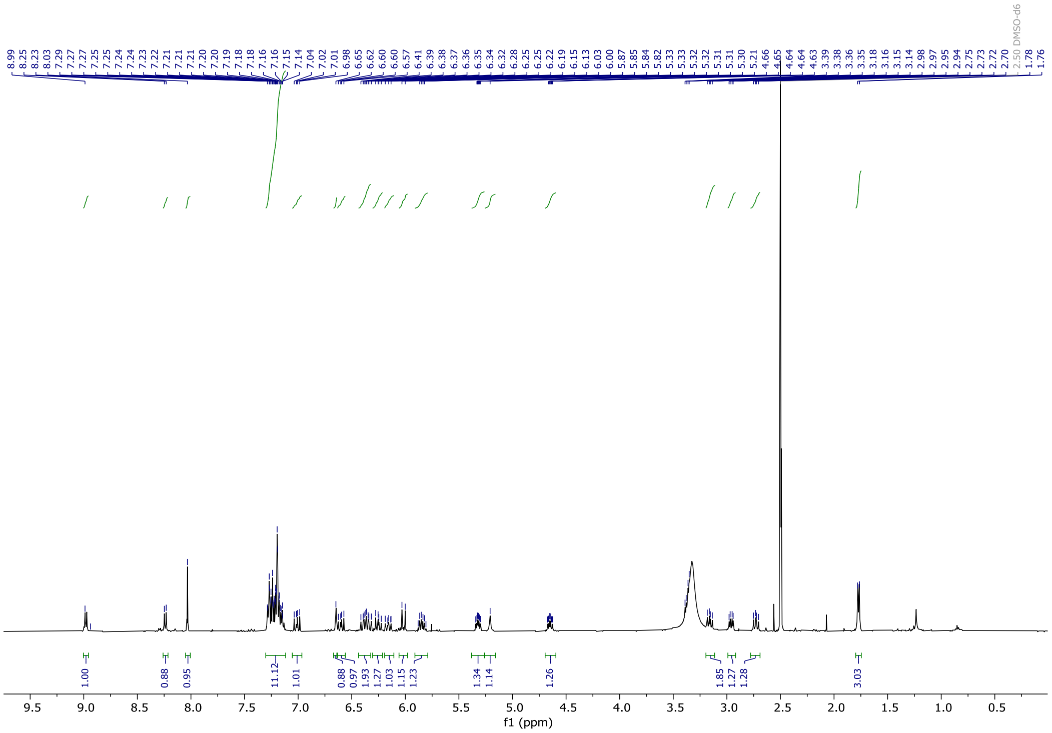

[14] Li, Z.-R.; Sun, J.; Du, Y.; Pan, A.; Zeng, L.; Maboudian, R.; Burne, R. A.; Qian, P.-Y.; Zhang, W. Mutanofactin promotes adhesion and biofilm formation of cariogenic *Streptococcus mutans*. *Nat. Chem. Biol.* **2021**, *17* (5), 576–584. DOI: 10.1038/s41589-021-00745-2.

^{13}C-NMR reproduced from Zhang *et al:* [15]

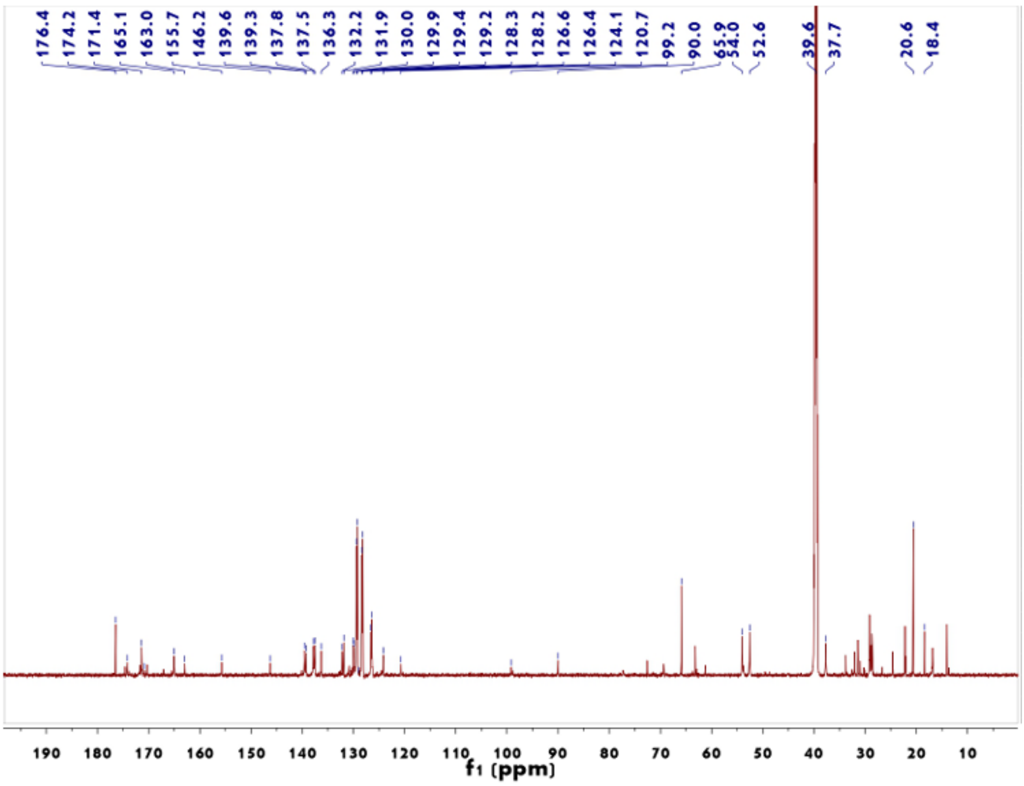

^{13}C NMR of Synthetic Material:

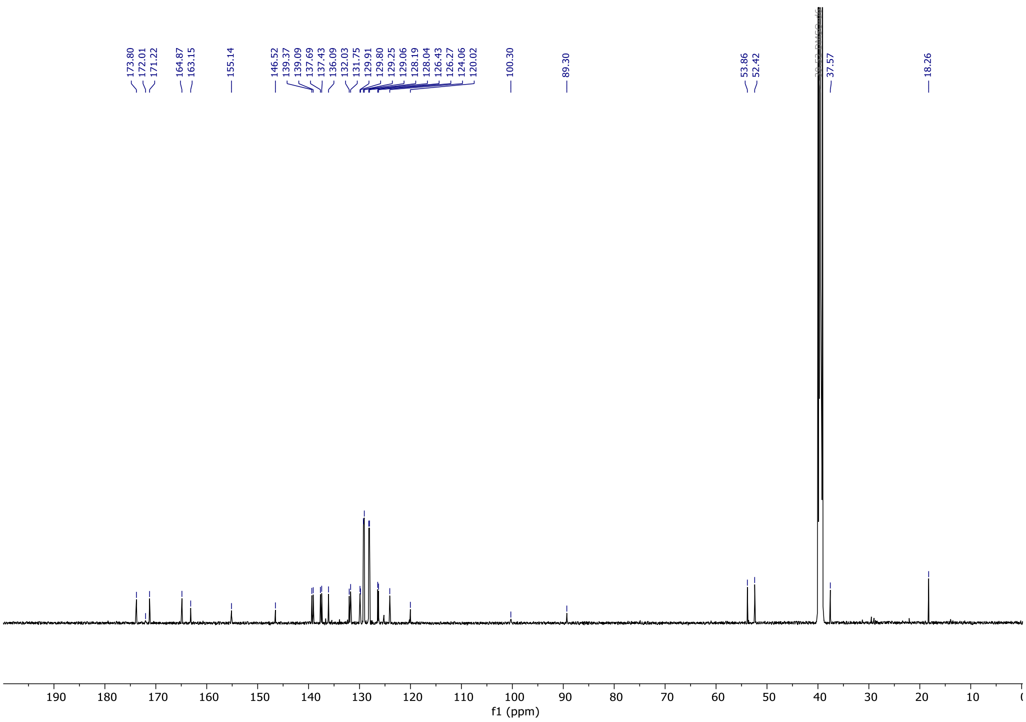

[15] Li, Z.-R.; Sun, J.; Du, Y.; Pan, A.; Zeng, L.; Maboudian, R.; Burne, R. A.; Qian, P.-Y.; Zhang, W. Mutanofactin promotes adhesion and biofilm formation of cariogenic *Streptococcus mutans*. *Nat. Chem. Biol.* **2021**, *17* (5), 576–584. DOI: 10.1038/s41589-021-00745-2.

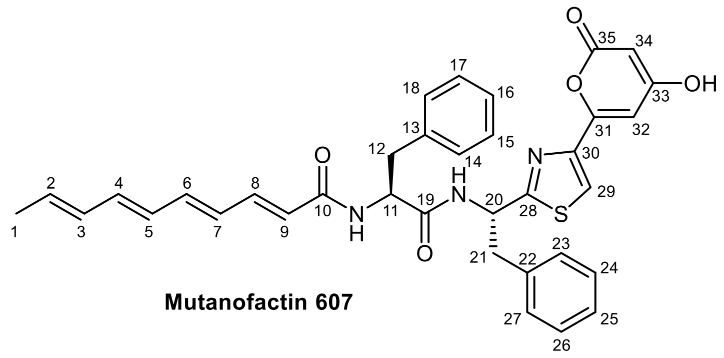

Mutanofactin 607

#	Isolation ^{13}C	Synthetic ^{13}C	Difference [ppm]	Isolation ^{1}H	Synthetic ^{1}H	Difference [ppm]
1	18.4	18.3	-0.1	1.77 (d, *J* = 6.9 Hz)	1.77, (d, 6.7 Hz)	0.00
2	132.2	132.0	-0.2	5.84 (dq, *J* = 14.9, 6.9 Hz)	5.84 (dq, *J* = 14.0, 6.9 Hz)	0.00
3	131.9	131.8	-0.1	6.16 (dd, *J* = 14.9, 11.1 Hz)	6.16 (ddq, *J* = 15.2, 10.7, 1.5 Hz)	0.00
4	136.3	136.1	-0.2	6.39 (dd, *J* = 15.6, 11.1)	6.39 (dd, *J* = 15.1, 10.7 Hz)	0.00
5	130.0	129.9	-0.1	6.25 (dd, *J* = 15.6, 11.1)	6.25 (dd, *J* = 14.9, 11.0 Hz, 1H)	0.00
6	139.3	139.1	-0.2	6.60 (dd, *J* = 14.8, 11.1)	6.60 (dd, *J* = 14.8, 11.0 Hz, 1H)	0.00
7	129.9	129.8	-0.1	6.34 (dd, *J* = 14.8, 11.4)	6.34 (dd, *J* = 15.2, 11.8 Hz)	0.00
8	139.6	139.4	-0.2	7.01 (dd, *J* = 15.1, 11.4)	7.01 (dd, *J* = 14.7, 11.2 Hz)	0.00
9	124.1	124.1	0.0	6.03 (d, *J* = 15.1)	6.02 (d, *J* = 15.1 Hz)	-0.01
10	165.1	164.9	-0.2	-	-	-
10-NH	-			8.26 (d, *J* = 8.5)	8.24 (d, *J* = 8.6 Hz)	-0.02
11	54.0	53.9	-0.1	4.64 (m)	4.65 (ddd, *J* = 9.8, 8.5, 4.9 Hz)	0.01
12	37.7	37.6	-0.1	2.95 (m)	2.96 (dd, *J* = 13.8, 4.9 Hz)	0.01
	-			2.73 (m)	2.73 (dd, *J* = 13.8, 9.7 Hz)	0.00
13	137.8	137.7	-0.1	-	-	-
14	129.4	129.3	-0.1	7.28 (m)	7.28 (m)	0.00
15	128.3	128.2	-0.1	7.20-7.24 (m)	7.20-7.24 (m)	0.00
16	126.4	126.4	0.0	7.15 (m)	7.15 (m)	0.00
17	128.3	128.2	-0.1	7.20-7.24 (m)	7.20-7.24 (m)	0.00
18	129.4	129.3	-0.1	7.28 (m)	7.28 (m)	0.00
19	171.4	171.2	-0.2	-	-	-

19-NH	-			9.01 (brs)	8.98 (d, *J* = 8.1 Hz)	-0.03
20	52.6	52.4	-0.2	5.32 (m)	5.32 (ddd, *J* = 9.5, 8.1, 5.4 Hz)	0.00
21	39.6	39.9	0.3	3.37 (m)	3.37 (dd, *J* = 14.1, 5.5 Hz)	0.00
				3.17 (m)	3.16 (dd, *J* = 14.0, 9.4 Hz)	-0.01
22	137.5	137.4	-0.1	-	-	-
23	129.2	129.1	-0.1	7.28 (m)	7.28 (m)	0.00
24	128.2	128.0	-0.2	7.20-7.24 (m)	7.20-7.24 (m)	0.00
25	126.6	126.3	-0.3	7.15 (m)	7.15 (m)	0.00
26	128.2	128.0	-0.2	7.20-7.24 (m)	7.20-7.24 (m)	0.00
27	129.2	129.1	-0.1	7.28 (m)	7.28 (m)	0.00
28	174.2	173.8	-0.4	-	-	-
29	120.7	120.0	-0.7	8.08 (s)	8.03 (s)	-0.05
30	146.2	146.5	0.3	-	-	-
31	155.7	155.1	-0.6	-	-	-
32	99.2	100.3	1.1	6.72 (s)	6.65 (s)	-0.07
33	170.8	172.0	1.2	-	-	-
34	90.0	89.3	-0.7	5.36 (s)	5.21 (s)	-0.15
35	163.0	165.2	2.2		-	-

Note: the ^{1}H NMR matches very well, except for the resonance corresponding to C-H_{34} (-0.15 ppm difference). We have however seen concentration dependence of this signal in various occasions, which is likely the cause of the deviation. Below sections of ^{1}H NMR spectra of Muf-607 at four different concentrations, where C_{34}-H shifts over a range of 0.4 ppm in the 1H NMR:

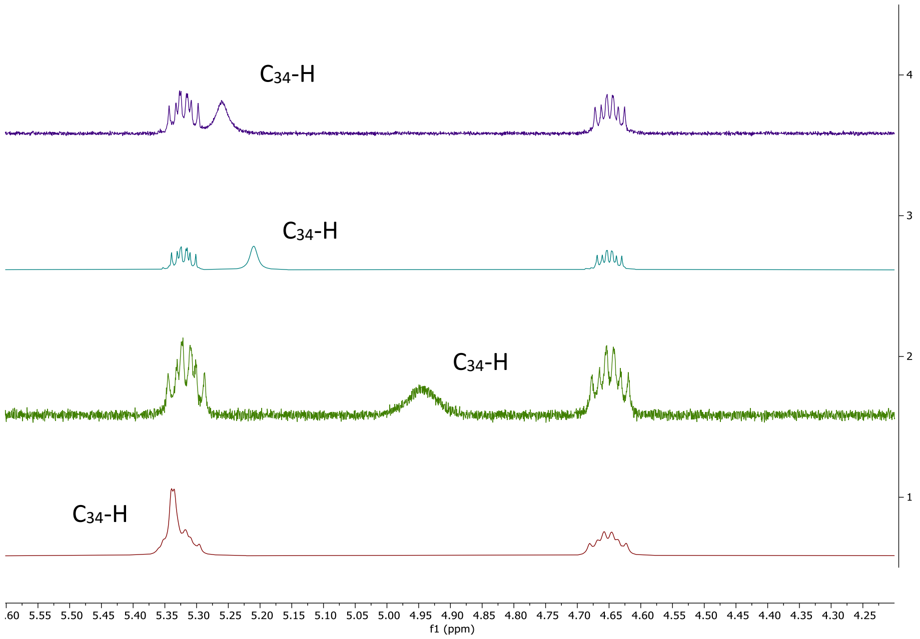

Similarly, ^{13}C shifts from C_{29}-C_{35} deviate from the isolation report. We believe this is due to either the same concentration effects as above, or due to lower quality spectra and impurities present in the isolation report. The assignment of ^{13}C signals in our case is unambiguous with the high quality 2D NMR spectra available.

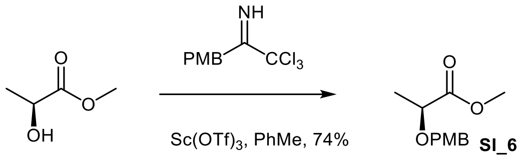

PMB-Ether SI_6: 1,1,1-trichloro-3-(4-methoxyphenyl)propan-2-imine[(1)] (13.3 g, 47.2 mmol, 1.50 equiv) was added to a dried round bottom flask, and dissolved in 80.0 mL dry toluene. Methyl (S)-2-hydroxypropanoate (3.00 mL, 31.5 mmol, 1.00 equiv) was added, followed by $Sc(OTf)_3$ (465 mg, 0.944 mmol, 0.03 equiv). Upon addition of the $Sc(OTf)_3$, crashing out of a solid was observed. The suspension was stirred for 1 hour at r.t., then quenched by the addition of 2 mL sat. $NaHCO_3$, followed by 1.00 g of solid $NaHCO_3$. The mixture was filtered over celite, the filter cake washed with 100 mL toluene and the filtrate evaporated to a crude. The crude was purified by column chromatography (dry load on celite, Ether/Pentane, 5-25%) giving the desired product **SI_6** as a yellow oil (5.20 g, 23.2 mmol, 74 %).

Note (1): Prepared according to Ikeuchi et al [16].

Rf = 0.49 (40% Ether in hexanes, UV-active, purple with Ceric ammonium molybdate stain)

^{1}H NMR (400 MHz, $CDCl_3$) δ 7.33 – 7.26 (m, 2H), 6.91 – 6.85 (m, 2H), 4.64 – 4.57 (d, *J* = 11.3 Hz, 1H), 4.39 (d, *J* = 11.3 Hz, 1H), 4.05 (q, *J* = 6.9 Hz, 1H), 3.80 (s, 3H), 3.75 (s, 3H), 1.42 (d, *J* = 6.9 Hz, 3H).

^{13}C NMR (101 MHz, $CDCl_3$) δ 173.8, 159.4, 129.6, 113.9, 73.7, 71.7, 55.3, 51.9, 18.7.

Spectral data matched previously reported: [17]

---

[16] Kazutada Ikeuchi, K. M., Hidetoshi Yamada. A Simple Method for the Preparation of Stainless and Highly Pure Trichloroacetimidates. *Synlett* **2019**, *30* (11), 1308–1312. DOI: 10.1055/s-0037-1611551.
[17] Curti, C.; Zanardi, F.; Battistini, L.; Sartori, A.; Rassu, G.; Pinna, L.; Casiraghi, G. Streamlined, Asymmetric Synthesis of 8,4′-Oxyneolignans. *J. Org. Chem.* **2006**, *71* (22), 8552–8558. DOI: 10.1021/jo061521t.

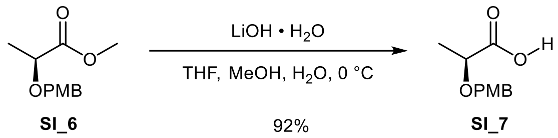

SI_6 SI_7

Acid SI_7: PMB ether **SI_6** (5.20 g, 23.2 mmol, 1.00 equiv) was dissolved in a mixture of 20.0 mL THF, 5.00 mL MeOH, 15.0 mL water and cooled to 0 °C. LiOH•H_2O (2.92 g, 69.6 mmol, 1.00 equiv) was added at once and the solution stirred for 1 hour. Found to be complete based on TLC, so worked up by pouring into 125 mL water. The mixture was extracted three times with hexane, and the hexane phases discarded. The aqueous phase was then acidified to pH 1 by the addition of 2 M HCl, then extracted five times with ether. The combined ether-phases were dried over Na_2SO_4, filtered and evaporated to give desired **SI_7** (4.47 g, 21.3 mmol, 92%), which was used without further purification.

Rf = 0.13-0.46 (1:1 hexane/EtOAc, 1% AcOH, ceric ammonium molybdate, UV active)

^{1}H NMR (400 MHz, DMSO) δ 12.65 (s, 1H), 7.29 – 7.22 (m, 2H), 6.93 – 6.87 (m, 2H), 4.50 (d, *J* = 11.2 Hz, 1H), 4.32 (d, *J* = 11.2 Hz, 1H), 3.95 (q, *J* = 6.9 Hz, 1H), 3.74 (s, 3H), 1.28 (d, *J* = 6.9 Hz, 3H).

^{13}C NMR (101 MHz, DMSO) δ 174.2, 158.7, 130.0, 129.3, 113.6, 73.1, 70.4, 55.0, 18.5.

Spectral data matched previously reported.[18]

[18] Balog James Aaron, S. S., David Williams Andappan Murugaiah Subbaiah Murugaiah. WO2019/136112 - INHIBITORS OF INDOLEAMINE 2,3-DIOXYGENASE AND METHODS OF THEIR USE. 2019.

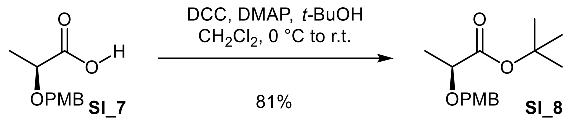

***Tert*-butyl ester SI_8**: Acid **SI_7** (4.47 g, 21.3 mmol, 1.00 equiv) was dissolved in 54.0 mL of CH_2Cl_2 and cooled to 0 °C using an ice-water bath. Sequentially, *t*-BuOH (4.47 mL, 46.8 mmol, 2.20 equiv) was added to the stirred solution, followed by DCC (5.70 g, 27.6 mmol, 1.30 equiv) as a solution in 18.0 mL of CH_2Cl_2. To the resulting suspension, DMAP (857 mg, 7.02 mmol, 0.33 equiv) was added, and the reaction stirred overnight, while warming to ambient temperature. The reaction was then filtered over celite, the filter cake washed with CH_2Cl_2, and the filtrate evaporated to a crude. The crude was purified by column chromatography (Hexane/EtOAc), yielding product **SI_8** as a clear oil (4.56 g, 17.1 mmol, 81%).

Rf = 0.75 (30% EtOAc/Hexanes, UV active, Ceric ammonium molybdate stain)

^{1}H NMR (400 MHz, $CDCl_3$) δ 7.36 – 7.29 (m, 2H), 6.94 – 6.86 (m, 2H), 4.64 (d, *J* = 11.2 Hz, 1H), 4.39 (d, *J* = 11.1 Hz, 1H), 3.93 (q, *J* = 6.8 Hz, 1H), 3.82 (s, 3H), 1.52 (s, 9H), 1.40 (d, *J* = 6.9 Hz, 3H).

^{13}C NMR (101 MHz, $CDCl_3$) δ 172.6, 159.3, 130.0, 129.6, 113.8, 81.2, 74.2, 71.5, 55.3, 28.1, 18.7.

HRMS (ESI) m/z calculated for C15H22NaO4 $[M+Na]^+$: 289.1410, found: 289.1403

FTIR (thin film): 2980, 2936, 2838, 1740, 1614, 1514, 1248, 1139, 1115, 1064, 1036, 847, 823

O.R. $[a]_D^{29}$ = -66.9 (c = 1.0, $CHCl_3$)

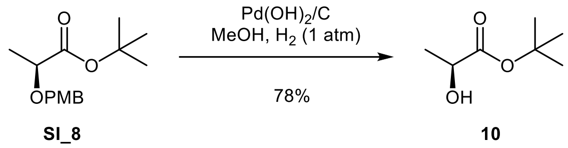

tert-butyl (S)-2-hydroxypropanoate SI_9: *Tert*-butyl ester **SI_8** (1.00 g, 3.76 mmol, 1.00 equiv) was dissolved in 20.0 mL MeOH and set under N_2. $Pd(OH)_2$ /C, 50% aq (104 mg, 0.075 mmol, 2.00 mol% based on [Pd]) was added at once, and the mixture stirred vigourously. The atmosphere was exchanged (3x) for hydrogen, and then the mixture stirred under an atmosphere of H_2 for 3 hours. The mixture was then diluted with CH_2Cl_2, filtered over celite, the filter cake rinsed with CH_2Cl_2 and evaporated to a crude. Purification by column chromatography (5-20% Ether/Pentane) gave the desired product **10** as a crystalline solid (430 mg, 2.94 mmol, 78%).

Rf = 0.55 (1:1 EtOAc/Hexanes, $KMnO_4$)

^{1}H NMR (400 MHz, $CDCl_3$) δ 4.14 (qd, *J* = 6.9, 5.3 Hz, 1H), 2.83 (d, *J* = 5.3 Hz, 1H), 1.49 (s, 9H), 1.37 (d, *J* = 6.9 Hz, 3H).

^{13}C NMR (101 MHz, $CDCl_3$) δ 175.3, 82.4, 67.1, 28.2, 20.7.

Spectral data matched previously reported: [19]

[19] Chojnacka, M. W.; Batey, R. A. Total Synthesis of (+)-Prunustatin A: Utility of Organotrifluoroborate-Mediated Prenylation and Shiina MNBA Esterification and Macrolactonization To Avoid a Competing Thorpe–Ingold Effect Accelerated Transesterification. *Org. Lett.* **2018**, *20* (18), 5671–5675. DOI: 10.1021/acs.orglett.8b02396.

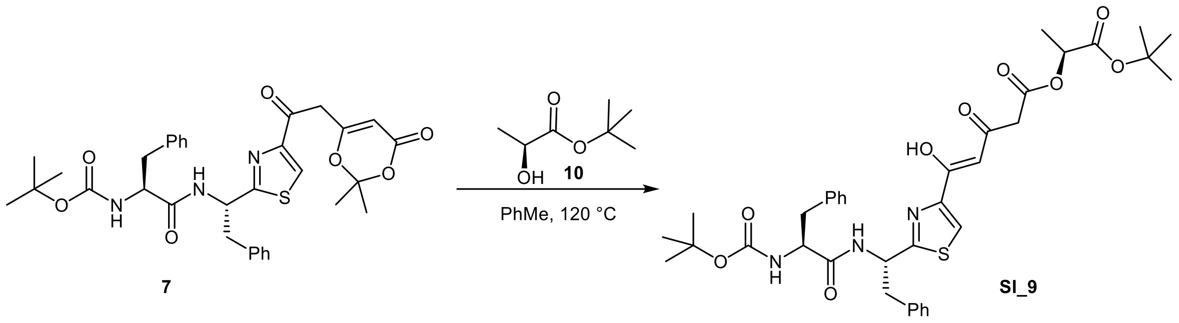

Lactate adduct SI_9: Ketone **7** (10.0 mg, 0.0161 mmol, 1.00 equiv) was added to an oven dried schlenk flask and dissolved in dry toluene (0.5 mL). *t*-Bu-Lactate (11.8 mg, 0.081 mmol, 5.00 equiv) was added, and the schlenk flask immersed in a pre heated sand bath (125 °C) and stirred for 1 hour, until TLC showed consumption of SM. The flask was then removed from the sand bath and cooled to .r.t, the toluene evaporated under a stream of nitrogen overnight. Dry CH_2Cl_2 was added (ca. 1 mL) and evaporated in vacuo, removing traces of *t*ert-butyllactate **10**. This process was repeated three times. Usually the product was carried forward crude, but preparative HPLC gave an analytical sample:

Retention Time: Gradient: 60% Water for 1 minute, then linear decrease to 10% Water over 27 minutes. Product elutes at 15.5 min, based on MS and UV_{254}, collected form 14.9-16.0 minutes.

^{1}H NMR (500 MHz, DMSO) δ 8.83 (d, *J* = 8.3 Hz, 1H), 8.47 (s, 1H), 8.40 (s, 1H), 7.34 – 7.25 (m, 4H), 7.22 (m, 3H), 7.19 – 7.13 (m, 3H), 6.89 (t, *J* = 8.1 Hz, 1H), 6.66 (s, 1H), 5.35 (q, *J* = 8.4 Hz, 1H), 4.88 (p, *J* = 6.8 Hz, 1H), 4.18 (td, *J* = 9.4, 4.8 Hz, 1H), 3.73 (s, 2H), 3.40 (dd, *J* = 14.0, 5.0 Hz, 1H), 3.19 (dd, *J* = 14.0, 9.7 Hz, 1H), 2.84 (dd, *J* = 13.7, 4.7 Hz, 1H), 2.72 – 2.64 (m, 1H), 1.39 (m, 12H), 1.29 (d, *J* = 1.7 Hz, 9H).

^{13}C NMR (126 MHz, DMSO) δ 190.8, 174.7, 173.9, 171.7, 169.1, 166.9, 155.0, 149.4, 137.8, 137.4, 129.2, 129.1, 128.2, 128.0, 126.5, 126.21, 126.20, 98.7, 81.5, 78.0, 69.7, 55.8, 52.3, 44.9, 39.2*, 37.4, 28.1, 27.5, 16.5.

*Extracted from HSQC spectra, due to overlap with DMSO-d6 signal in 13C.

Note: The product likely exists as a mixture of tautomers, see discussion for Muf 697

HRMS (ESI) m/z calculated for C37H46N3O9S $[M+H]^+$:, 708.2949 found: 708.2934

FTIR (thin film): 3337, 3287, 3029, 2933, 2980, 1742, 1665, 1604, 1495, 1454, 1320, 1240, 1165, 698

O.R. $[\alpha]_D^{25}$ = -22.6 (c = 0.50, $CHCl_3$)

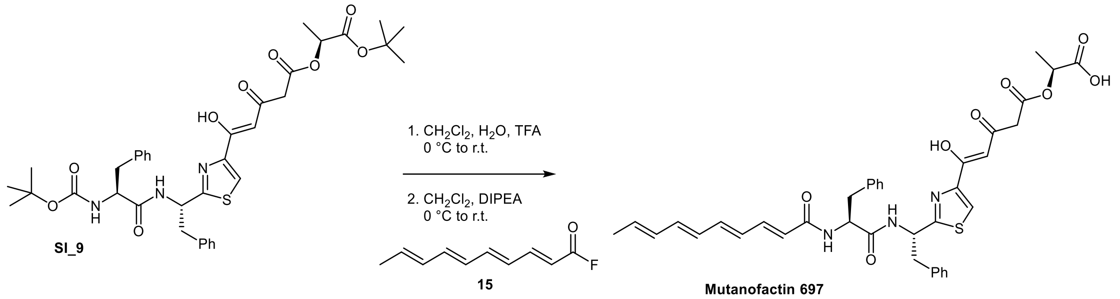

Mutanofactin 697: **SI_9** (Assumed quantitative, 0.0161 mmol, 1.00 equiv) was dissolved in 0.30 mL CH_2Cl_2 and cooled to 0 °C. One drop of water was added, followed by slow addition of TFA (0.125 mL, 1.61 mmol, 100 equiv.). The resulting solution was allowed to warm to ambient temperature, after a total reaction time of 6 hours (deprotection was monitored by LC-MS), the mixture was concentrated under a stream of nitrogen. The solid was redissolved in ca. 0.5 mL CH_2Cl_2 and evaporated to remove remaining TFA (repeated 3x in total.). The residue was dissolved in 0.20 mL DMF and cooled to 0 °C, followed by the addition of DIPEA(1)(0.015 mL, 0.081 mmol, 5.00 equiv.) and Acyl fluoride 15 (5.4 mg, 0.032 mmol, 2.00 equiv, dissolved in 0.10 mL DMF). The reaction was allowed to warm to ambient temperature and stirred overnight. The reaction was cooled to 0 °C and quenched by the sequential addition of 3-5 drops of aq. NH_3, and stirred for 2-3 minutes followed by 0.1 mL Formic acid. Dropwise, DMF was added until a homogeneous solution was obtained, which was directly purified by preparative HPLC, yielding Mutanofactin 697 (4.8 mg, 0.0068 mmol, 43% over three steps) as a white powder upon lyophilization(2).

Note (1): DIPEA was freshly distilled from CaH_2 under N_2 atmosphere before use.Note (2): Similar products are known to be light-sensitive, so the product was stored in amber vials in the dark, regular maniulations (reaction, lyophilization) were carried out with minimal exposure to light.

*Note(3): The product exists as a mixture of multiple tautomers, below the NMR data for the major tautomer are shown, where applicable peaks due to the 2nd tautomer are marked with an *.*

Retention Time: Gradient: 60% Water for 1 minute, then linear decrease to 20% Water over 17 minutes. Product elutes at 11.3 min, based on MS and UV_{330}, collected from 10.8-11.8 minutes.

^{1}H NMR (600 MHz, DMSO) δ 13.10 (s, 1H), δ 8.99 (d, *J* = 8.2 Hz, 1H), 8.39 (s, 1H), 8.25 (d, *J* = 8.8 Hz, 1H), 7.30 – 7.13 (m, 10H), 7.01 (dd, *J* = 15.0, 11.5 Hz, 1H), 6.65 (s, 1H), 6.60 (dd, *J* = 14.8, 11.0 Hz, 1H), 6.39 (dd, *J* = 14.8, 10.7 Hz, 1H), 6.34 (dd, *J* = 15.1, 12.0 Hz, 1H), 6.25 (dd, *J* = 15.0, 11.0 Hz, 1H), 6.16 (ddd, *J* = 14.8, 10.8, 1.6 Hz, 1H), 6.02 (d, *J* = 15.0 Hz, 1H), 5.85 (dq, *J* = 14.1, 6.9 Hz, 1H), 5.36 – 5.28 (m, 1H), 4.97 (q, *J* = 7.0 Hz, 1H), 4.64 (td, *J* = 9.1, 5.0 Hz, 1H), 3.74 (q, *J* = 15.8 Hz, 2H), 3.38 (dd, *J* = 14.0, 5.2 Hz, 1H), 3.16 (dd, *J* = 14.0, 9.6 Hz, 1H), 2.95 (dt, *J* = 13.9, 3.6 Hz, 1H), 2.72 (dd, *J* = 13.8, 9.6 Hz, 1H), 1.77 (d, *J* = 6.9 Hz, 1H), 1.41 (d, *J* = 6.9 Hz, 2H).

^{13}C NMR (151 MHz, DMSO) δ 197.9*, 190.8, 188.1*, 174.6, 173.7, 171.4, 171.3, 166.9, 164.9, 149.3, 139.4, 139.1, 137.6, 137.4, 136.1, 132.0, 131.7, 129.9, 129.8, 129.2, 129.1, 128.2, 128.1, 126.5, 126.4, 126.3, 124.1, 98.7, 69.1, 69.0*, 53.9, 52.4, 49.4*, 44.9, 39.2, 37.6, 18.3, 16.6.

HRMS (ESI) m/z calculated for C38H40N3O8S $[M+H]^+$: 698.2531, measured: 698.2522

FTIR (thin film) 3271, 2925, 2860, 1716, 1664, 1644, 1617, 1593, 1525, 1250, 1003, 698, 584

O. R. $[a]_D^{26}$ = -73.3 (c = 0.10, DMSO)

Comparison of Synthetic and isolated Mutanofactin 697

High Resolution MS/MS of synthetic mutanofactin 697 and proposed fragment structures

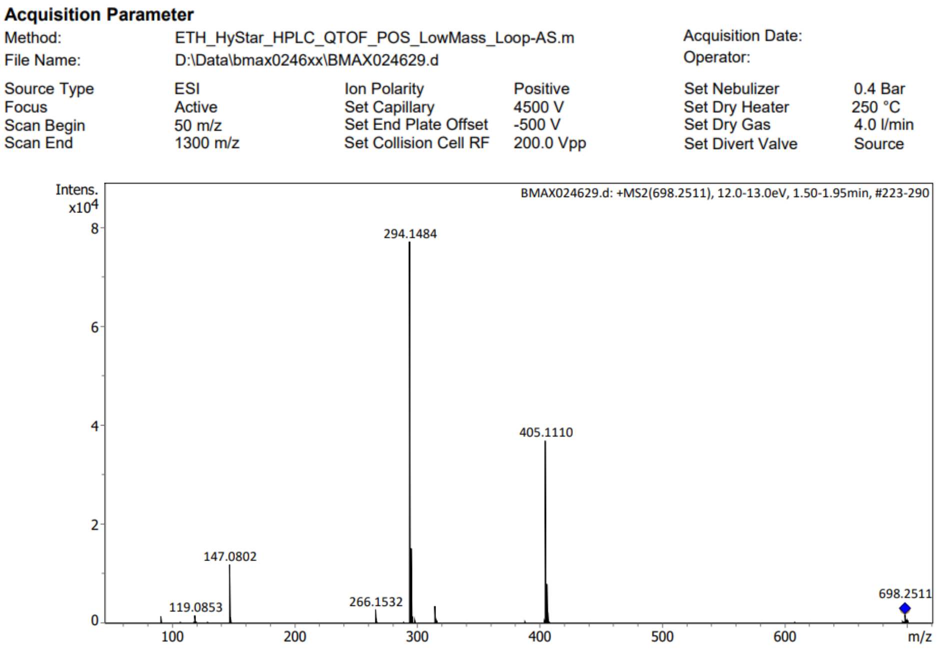

Comparison with Isolation MS-MS studies, reproduced from [20]:

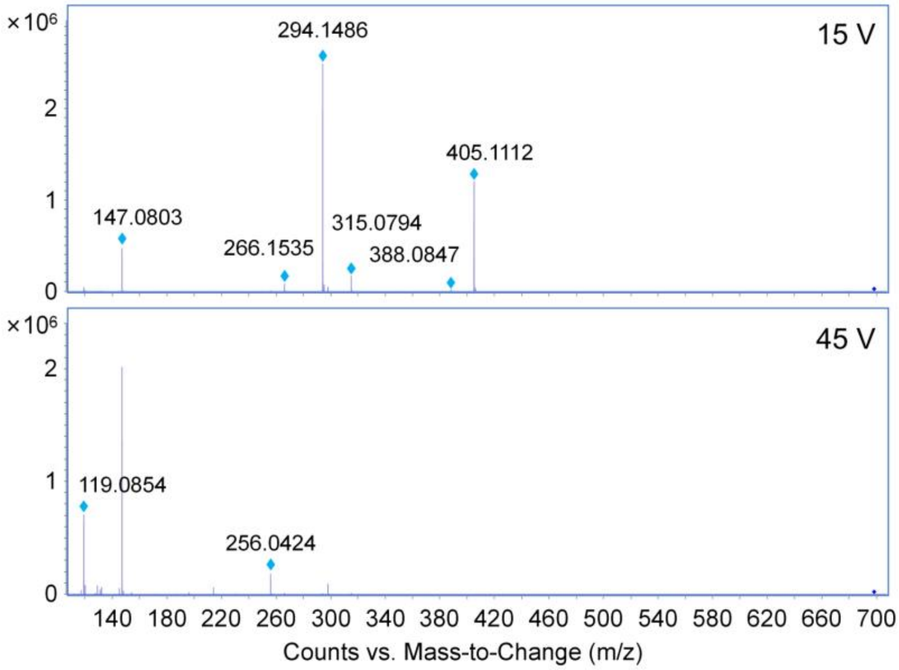

HR-MS/MS fragmentation pattern of **5**. Fragmentation was acquired with collision energies of 15 V or 45 V.

[20] Li, Z.-R.; Sun, J.; Du, Y.; Pan, A.; Zeng, L.; Maboudian, R.; Burne, R. A.; Qian, P.-Y.; Zhang, W. Mutanofactin promotes adhesion and biofilm formation of cariogenic *Streptococcus mutans*. *Nat. Chem. Biol.* **2021**, *17* (5), 576–584. DOI: 10.1038/s41589-021-00745-2.

Proposed Fragments in MS-MS:

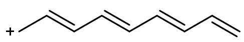

m/z
calculated: 119.0855
found: 119.0853

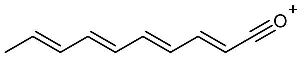

m/z
calculated: 147.0804
found: 147.0802

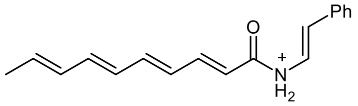

m/z
calculated: 266.1539
found: 266.1532

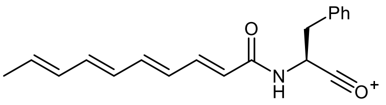

m/z
calculated: 294.1489
found: 294.1484

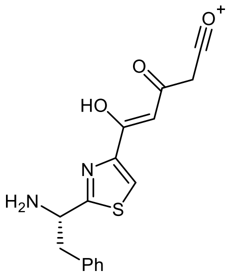

m/z
calculated: 315.0798
found: 315.0792

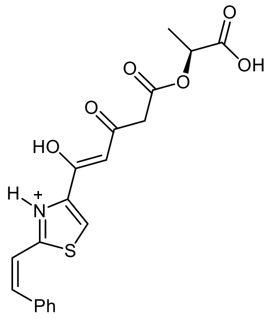

m/z
calculated: 388.0849
found: 388.0842

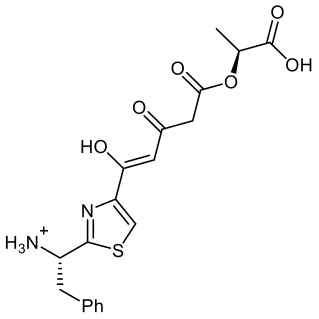

m/z
calculated: 405.1115
found: 405.1110

Comparison of isolated material with synthetic mutanofactin 697:

^{1}H-NMR reproduced from Zhang *et al*:[21]

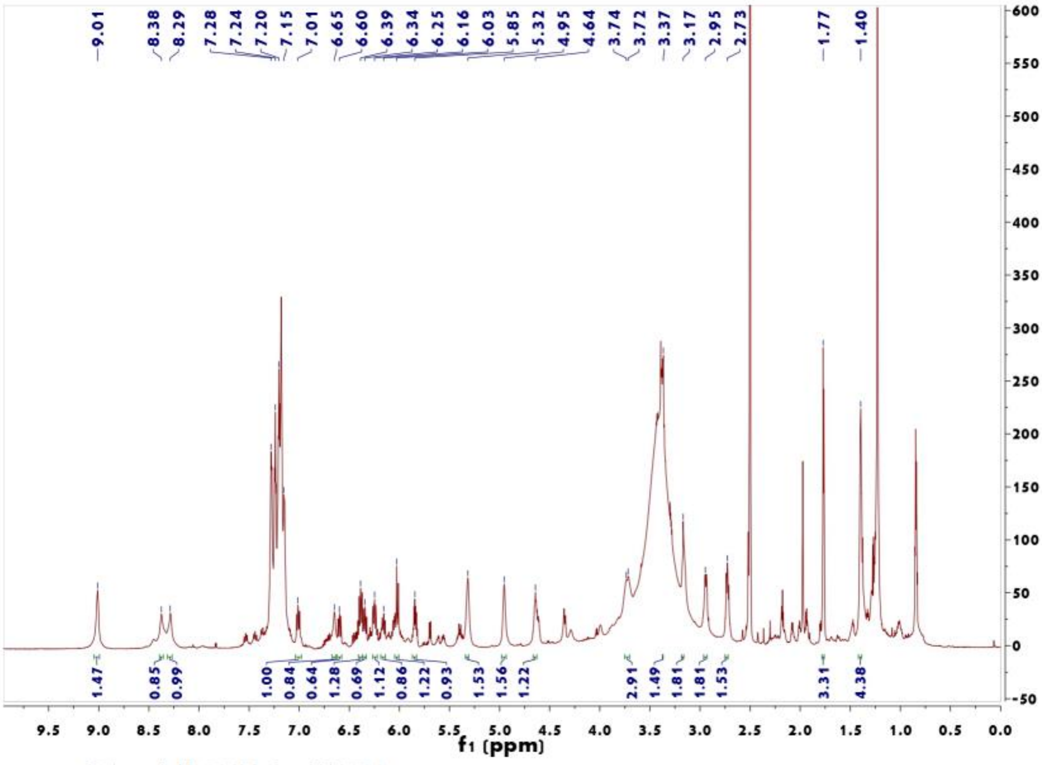

^{1}H NMR of Synthetic Material (this work):

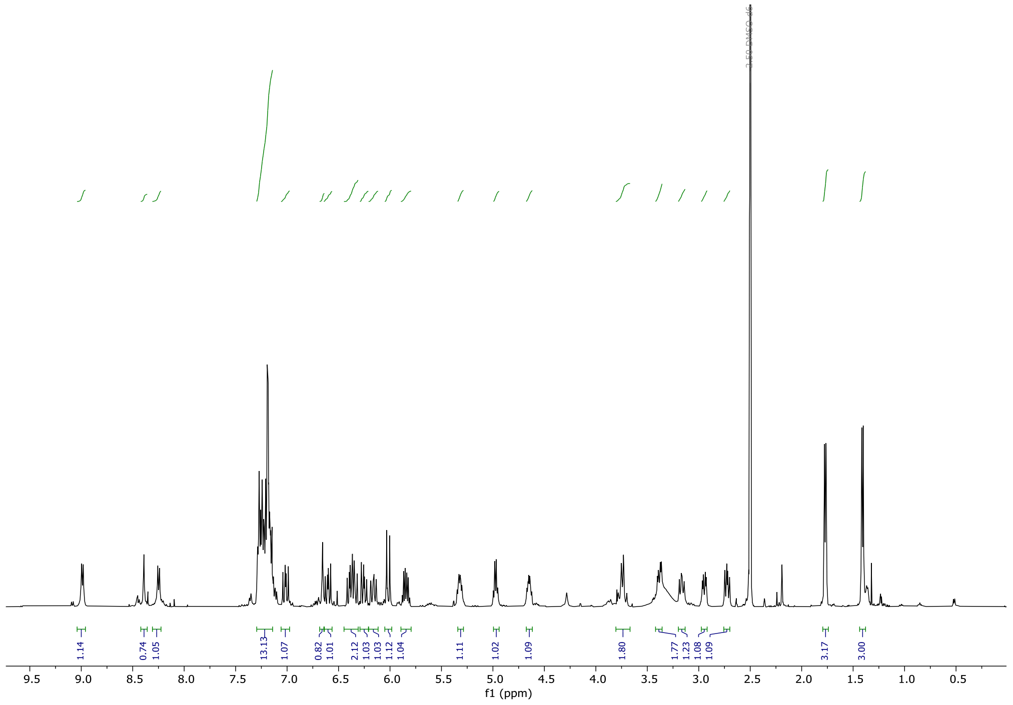

[21] Li, Z.-R.; Sun, J.; Du, Y.; Pan, A.; Zeng, L.; Maboudian, R.; Burne, R. A.; Qian, P.-Y.; Zhang, W. Mutanofactin promotes adhesion and biofilm formation of cariogenic *Streptococcus mutans*. *Nat. Chem. Biol.* **2021**, *17* (5), 576–584. DOI: 10.1038/s41589-021-00745-2.

^{1}H-NMR reproduced from Zhang *et al*:[22]

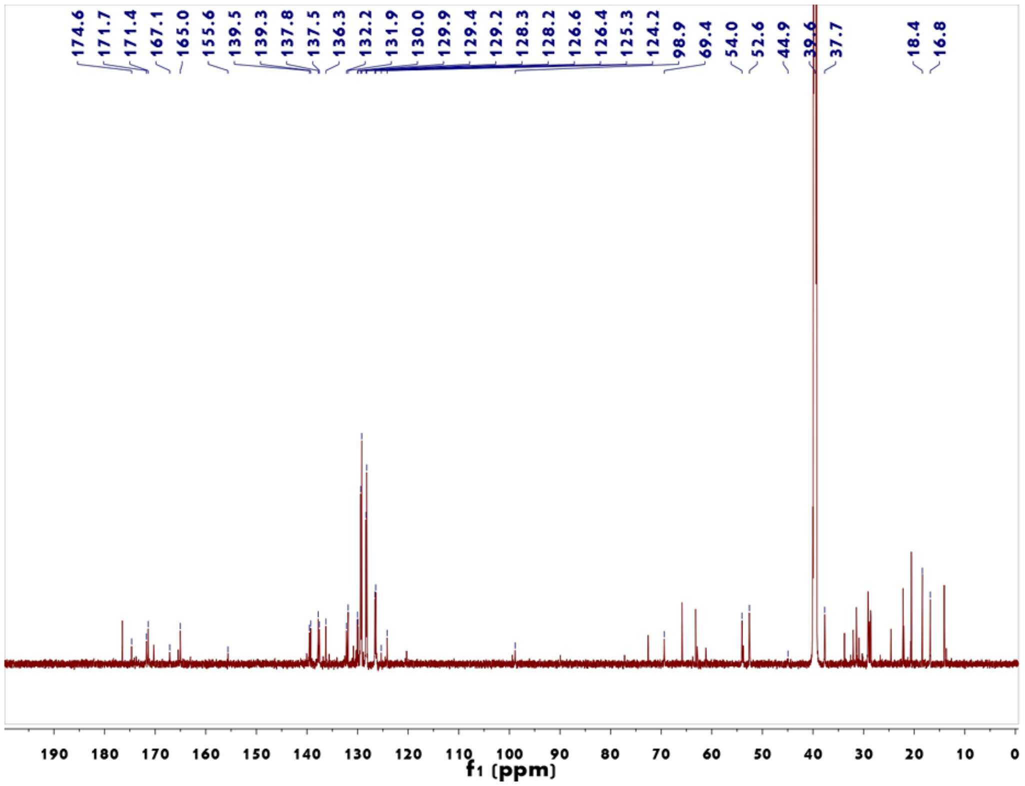

^{13}C NMR of Synthetic Material (this work):

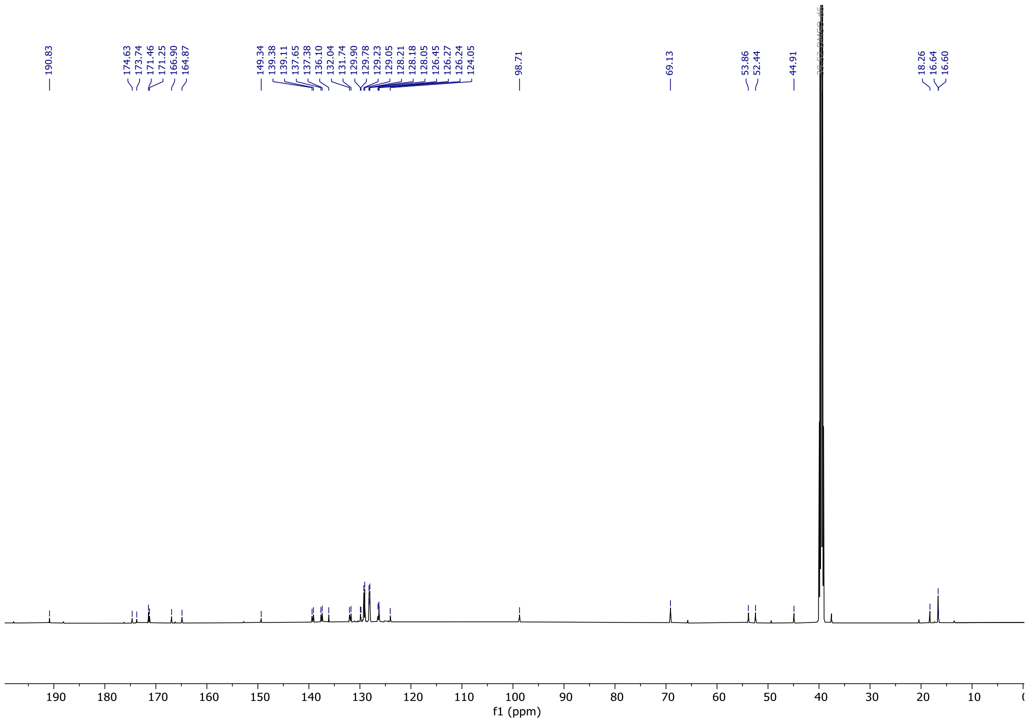

[22] Li, Z.-R.; Sun, J.; Du, Y.; Pan, A.; Zeng, L.; Maboudian, R.; Burne, R. A.; Qian, P.-Y.; Zhang, W. Mutanofactin promotes adhesion and biofilm formation of cariogenic *Streptococcus mutans*. *Nat. Chem. Biol.* **2021**, *17* (5), 576–584. DOI: 10.1038/s41589-021-00745-2.

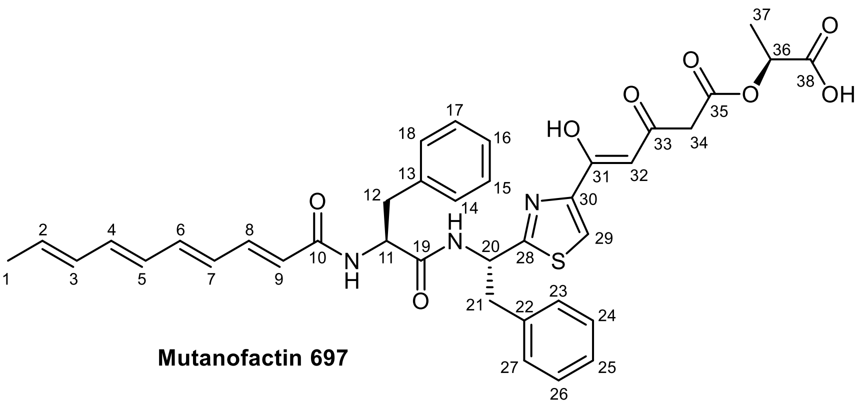

Mutanofactin 697

#	Isolation ^{13}C	Synthetic ^{13}C	Δ [ppm]	Isolation ^{1}H	Synthetic ^{1}H	Δ [ppm]
1	18.4	18.3	-0.1	1.77 (d *J* = 7.6 Hz)	1.77 (d, *J* = 6.9 Hz)	0.00
2	132.2	132.0	-0.2	5.85 (dq, *J* = 14.8, 7.6 Hz)	5.85 (dq, *J* = 14.1, 6.9 Hz)	0.00
3	131.9	131.7	-0.2	6.16 (dd, *J* = 14.8, 11.1 Hz)	6.16 (ddd, *J* = 14.8, 10.8, 1.6 Hz)	0.00
4	136.3	136.1	-0.2	6.39 (dd, *J* = 15.6, 11.1 Hz)	6.39 (dd, *J* = 14.8, 10.7 Hz)	0.00
5	130.0	129.9	-0.1	6.25 (dd, *J* = 15.6, 11.1 Hz)	6.25 (dd, *J* = 15.0, 11.0 Hz)	0.00
6	139.3	139.1	-0.2	6.60 (dd, *J* = 14.8, 11.1 Hz)	6.60 (dd, *J* = 14.8, 11.0 Hz)	0.00
7	129.9	129.9	0.0	6.34 (dd, *J* = 14.8, 11.4 Hz)	6.34 (dd, *J* = 15.1, 12.0 Hz)	0.00
8	139.5	139.4	-0.1	7.01 (dd, *J* = 15.1, 11.4 Hz)	7.01 (dd, *J* = 15.0, 11.5 Hz)	0.00
9	124.2	124.1	-0.1	6.03 (d, *J* = 15.1 Hz)	6.02 (d, *J* = 15.0 Hz)	-0.01
10	165.0	164.9	-0.1	-	-	
10-NH	-	-	-	8.29 (d, *J* = 8.8 Hz)	8.25 (d, *J* = 8.8 Hz)	-0.04
11	54.0	53.9	-0.1	4.64 (m)	4.64 (td, *J* = 9.1, 5.0 Hz)	0.00
12	37.7	37.6	-0.1	2.95 (m)	2.95 (dt, *J* = 13.9, 3.6 Hz)	0.00
	-		-	2.74 (m)	2.72 (dd, *J* = 13.8, 9.6 Hz)	-0.02
13	137.8	137.7	-0.1	-	-	
14	129.2	129.1	-0.1	7.28 (m)	7.28 (m)	0.00
15	128.2	128.1	-0.1	7.20-7.24 (m)	720-7.24 (m)	0.00
16	126.4	126.3	-0.1	7.15 (m)	7.15 (m)	0.00
17	128.2	128.1	-0.1	7.20-7.24 (m)	7.20-7.24 (m)	0.00
18	129.2	129.1	-0.1	7.28 (m)	7.28 (m)	0.00
19	171.4	171.4	0.0	-	-	
19-NH	-	-	-	9.01 (brs)	8.99 (d, *J* = 8.2 Hz)	-0.02
20	52.6	52.4	-0.2	5.32 (m)	5.32 (m)	0.00
21	39.6	39.2*	-0.4	3.37 (m)	3.38 (dd, *J* = 14.0, 5.2 Hz)	0.01

			0.0	3.17 (m)	3.16 (dd, *J* = 14.0, 9.6 Hz)	-0.01
22	137.5	137.4	-0.1	-	-	
23	129.4	129.2	-0.1	7.28 (m)	7.28 (m)	0.00
24	128.3	128.2	-0.1	7.20-7.24 (m)	7.20-7.24 (m)	0.00
25	126.6	126.5	-0.1	7.15 (m)	7.15 (m)	0.00
26	128.3	128.2	-0.1	7.20-7.24 (m)	7.20 (m)	0.00
27	129.4	129.2	-0.2	7.28 (m)	7.28 (m)	0.00
28	174.6	174.6	0.0	-	-	
29	125.3	126.4	0.9	8.38 (s)	8.39 (s)	0.01
30	NT	149.3	-	-	-	
31	NT	173.7	-	-	-	
31-OH					13.10 (s)	-
32	98.9	98.7	-0.2	6.65 (s)	6.65 (s)	0.00
33	NT	190.8	-	-	-	
34	44.9	44.9	0.0	3.72 (m)	3.74 (q, *J* = 15.8 Hz)	0.02
			-	3.72 (m)		
35	167.1	166.9	-0.2	-	-	
36	69.4	69.1	-0.3	4.95 (m)	4.97 (q, *J* = 7.0 Hz)	0.02
37	16.8	16.6	-0.2	1.40 (d, *J* = 7.3 Hz)	1.41 (d, *J* = 6.9 Hz)	0.01
38	171.1	171.3	0.2	-	-	

Note: Some peaks were not assigned by the isolation team (C_{30}, C_{31}, $C_{31\text{-OH}}$, C_{33}). For synthetic material, the signal corresponding to C_{21} overlaps with the solvent signal (DMSO-d6), accordingly the shift was extracted from the HSQC-spectra and marked with an *.

The spectra are in good agreement between synthetic and authentic material, deviations are only significant for two peaks: -0.4 ppm for C_{21}, and 0.9 ppm for C_{29}. Since in our case C_{21} is extracted from the HSQC spectra due to overlap with DMSO-d6 (no comment given by the isolation team), small deviations are to be expected. For C_{29}, the significant discrepancy is hypothesized to be due the small relative peak intensity (caused likely by keto-enol tautomerism at C_{31}-C_{33}, see discussion below), resulting in potential missasignment by the isolation team. In our case we are confident in the assigment based on the higher purity, and higher quality 2D spectra.

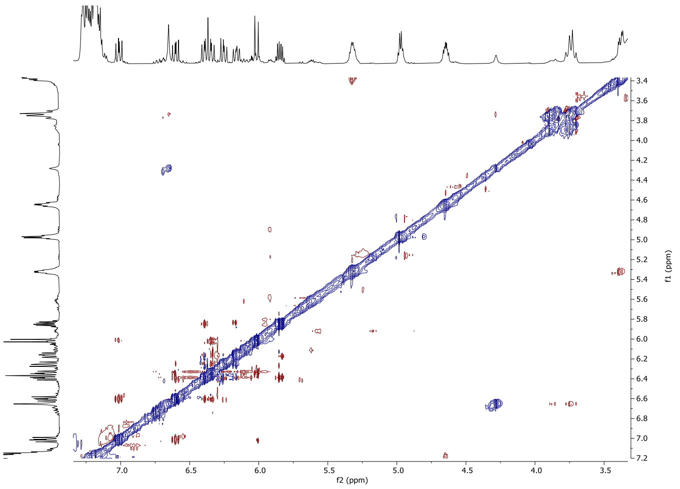

The Following ROESY Crosspeak between the singlet at 6.65 ppm (Assigned as C-H- of Enol tautomer, highlighted in blue on the structure on the left) and the singlet at 4.28 ppm (putative CH_2 of the Keto tautomer, shown in blue on the structure on the right) is presumably due to chemical exchange and thereby evidence for Keto-Enol Tautomerism. Further tautomerism (i.e. between the second ketone and the ester) is proposed to be present in low quantities (<5% of species).

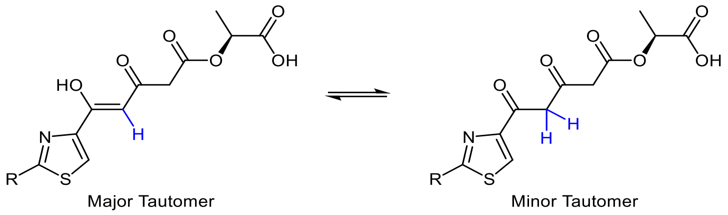

The Keto-Enol Tautomerism is concentration-dependant, influenced by the water content in the DMSO-d6, or both. The three 1H NMR spectra provided below are all Mutanofactin 697 with high purity based on LC-MS analysis, with however different concentrations/water content, and thus varying ratios of keto-enol tautomers.

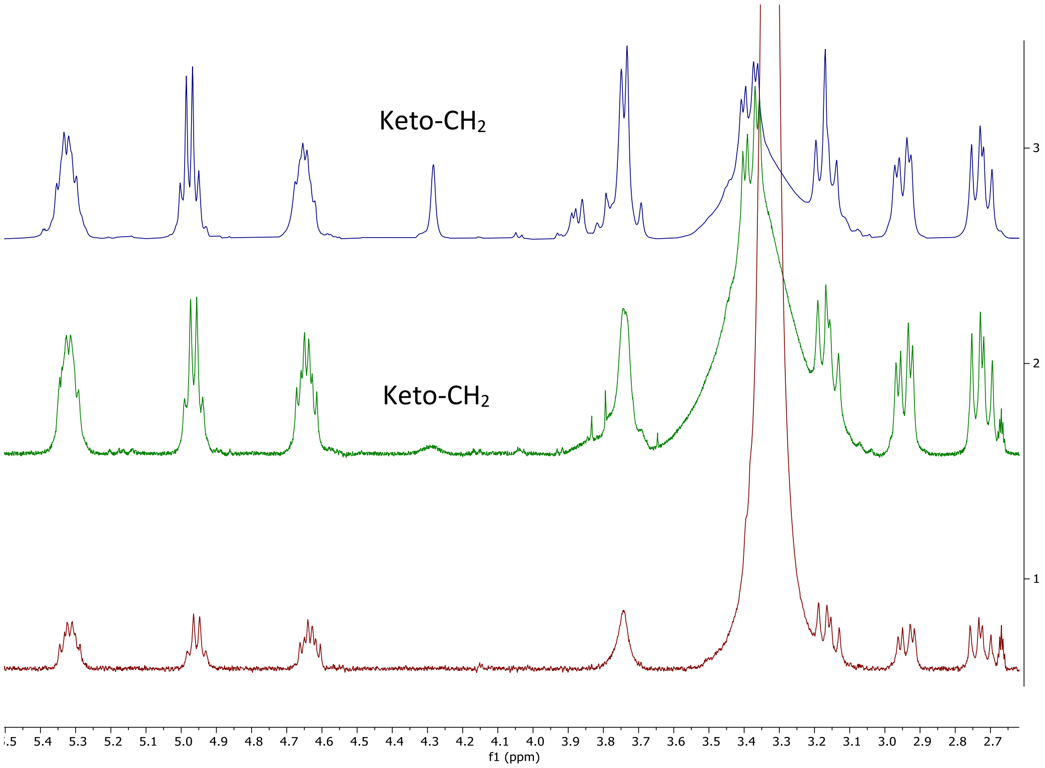

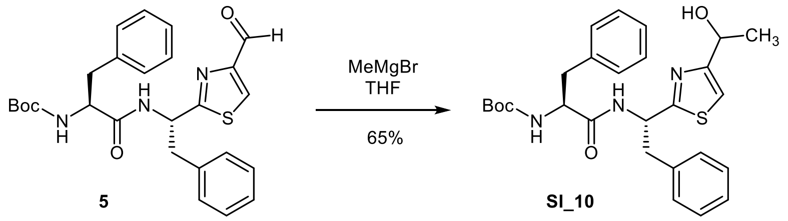

Alcohol SI_10: Aldehyde **5** (58.0 mg, 0.121 mmol, 1.00 equiv) was dissolved in 4.00 mL dry THF and cooled to –78 °C. Then, MeMgCl (0.240 mL, 0.730 mmol, 6.00 equiv, 3M in THF) was added dropwise, and the resulting solution slowly warmed to r.t. over the course of 3 hours. The reaction was cooled to 0 °C and quenched by the dropwise addition of sat. NH_4Cl. Extraction of the mixture 3x with CH_2Cl_2, followed by drying over Na_2SO_4, filtration and evaporation gave the crude. Purification by column chromatography (dry load on celite, 0.1-3% MeOH in CH_2Cl_2) gave the desired product **SI_10** as a foam (39.0 mg, 0.079 mmol, 65 %, inconsequential mixture of two diastereomers, ca. 1:1 according to 1H NMR analysis of the unpurified reaction mixture).

Rf = 0.30 (1:1 EtOAc/Hexanes, UV active, ninhydrin stain brown)

^{1}H NMR (500 MHz, $CDCl_3$) δ 7.24 – 7.16 (m, 6H), 7.16 – 7.10 (m, 2H), 6.99 – 6.92 (m, 3H), 6.72 (d, *J* = 8.2 Hz, 1H), 5.48 (q, *J* = 7.2 Hz, 1H), 4.97 (bs, 1H), 4.92 (q, *J* = 6.5 Hz, 1H), 4.35 (s, 1H), 3.20 (tdd, *J* = 12.3, 6.8, 2.3 Hz, 2H), 3.01 (d, *J* = 6.9 Hz, 2H), 2.69 (bs, 1H), 1.53 (d, *J* = 6.5 Hz, 1.5H), 1.53 (d, *J* = 6.5 Hz, 1.5H)* 1.39 (m, 9H).

*Nore: Peaks corresponding to the 2nd diasteromer are marked with an asterisk **

^{13}C NMR (126 MHz, $CDCl_3$) δ 170.8, 170.1, 160.73, 160.68*, 155.4, 136.6, 136.3*, 129.6, 129.4, 128.8, 128.7, 128.5, 127.0, 112.8, 80.4, 66.7, 66.6*, 55.9, 52.3, 41.7, 38.3, 28.44, 28.37, 23.2, 23.1*.

HRMS (ESI) m/z calculated for C27H34N3O4S $[M+H]^+$: 496.2265, 496.2259

FTIR (thin film): 3284, 3063, 3029, 2977, 2928, 1683, 1657, 1520, 1497, 1366, 1167, 1022, 856

O.R. $[\alpha]_D^{27}$ = -27.3 (c = 1.0, $CHCl_3$)

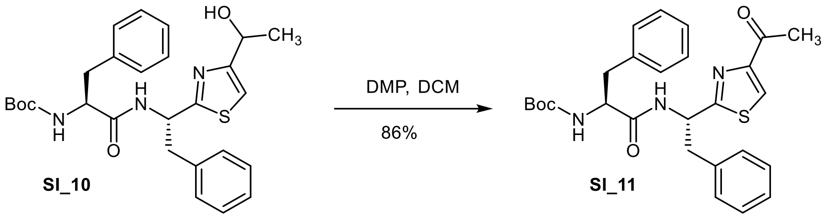

Methyl ketone SI-11: Alcohol **SI_10** (34.0 mg, 0.069 mmol, 1.00 equiv) was dissolved in 2.00 mL CH_2Cl_2 and cooled to 0 °C using an ice-water bath. DMP (43.6 mg 0.103 mmol, 1.50 equiv) was added at once, and the solution gradually warmed to r.t. After 3 hours, the reaction quenched by the simultaneous addition of sat. $NaHCO_3$ and sat. $Na_2S_2O_3$. The mixture was stirred for 3 minutes, then extracted four times with diethyl ether. The combined organic phases were dried over Na_2SO_4, filtered and evaporated to a crude. The crude was purified by column chromatography (0.1-1.5% MeOH in CH_2Cl_2) to yield methyl ketone **SI_11** as a white solid (29.0 mg, 0.059 mmol, 86%).

Rf = 0.66 (1:1 Hexanes/EtOAc, UV active, stains brown with ninhydrin stain)

^{1}H NMR (400 MHz, $CDCl_3$) δ 7.96 (s, 1H), 7.24 – 7.09 (m, 8H), 7.01 – 6.95 (m, 2H), 6.62 (d, *J* = 8.3 Hz, 1H), 5.50 (q, *J* = 7.2 Hz, 1H), 4.95 (s, 1H), 4.34 (d, *J* = 6.9 Hz, 1H), 3.28 (dd, *J* = 13.6, 6.4 Hz, 1H), 3.18 (dd, *J* = 13.6, 7.3 Hz, 1H), 3.01 (d, *J* = 7.3 Hz, 2H), 2.61 (s, 3H), 1.39 (s, 9H).

^{13}C NMR (126 MHz, $CDCl_3$) δ 193.1, 170.9, 170.2, 155.4, 154.9, 136.5, 136.0, 129.5, 129.4, 128.8, 128.7, 127.2, 127.1, 125.4, 80.5, 56.0, 52.3, 41.4, 38.3, 28.4, 28.0

HRMS (ESI) m/z calculated for C27H32N3O4S $[M+H]^+$: 494.2108, found: 494.2110

FTIR (thin film) 3336, 2978, 1786, 1687, 1664, 1520, 1367, 1295, 1242, 1167, 1021, 737, 698

O.R. $[\alpha]_D^{28}$ = -26.0 (c = 0.45, $CHCl_3$)

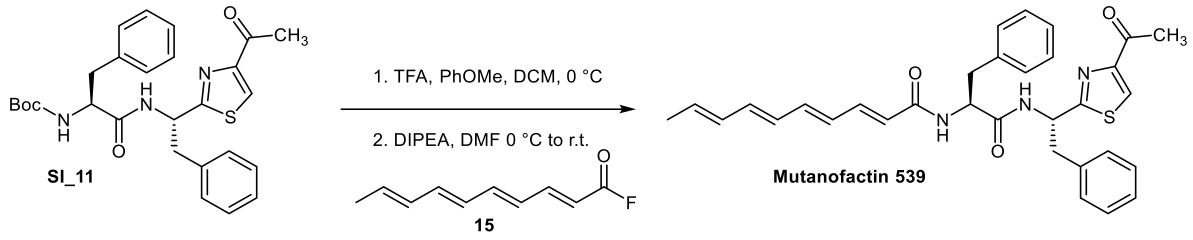

Mutanofactin 539: Methyl ketone **SI_11** (16.6 mg, 0.033 mmol, 1.00 equiv) was dissolved in 0.420 mL CH_2Cl_2, and cooled to 0 °C using an ice-water bath. 1 drop of distilled water was added, followed by TFA (0.260 mL, 3.36 mmol, 100 equiv.). The mixture was left stirring for two hours, slowly warming to ambient temperature. The mixture was then concentrated under a stream of N_2. Ca. 1 mL of CH_2Cl_2 was added and again concentrated (the process was repeated three times in total, then the residue dried under high vacuum for 15 min). The mixture was redissolved in 0.420 mL DMF, cooled to 0 °C and DIPEA(1) (0.03 mL, 0.17 mmol, 5.00 equiv) was added. Then, a solution of Acyl fluoride (11.2 mg, 0.066 mmol, 2.00 equiv.) in 0.200 mL DMF was added and the mixture stirred overnight, while allowing to warm to ambient temperature. The resulting suspension poured into 14.0 mL diethyl ether, the suspension agitated and subsequently centrifuged (3 min, 4000 rpm). The supernatant was decanted and discarded. The solids were resuspended in fresh diethyl ether, and the procedure repeated in total three times with diethyl ether. The resulting white powder was suspended in 1:1 water/MeCN and lyophilized to give desired product Mutanofactin 539 as a white solid (12.1 mg, 0.022 mmol, 66% yield).

Note (1): DIPEA was freshly distilled from CaH_2 under nitrogen atmosphere before use.

Note (2): Similar products are known to be light-sensitive, so the product was stored in amber vials in the dark, regular maniulations (reaction, lyophilization) were carried out with minimal exposure to light.

^{1}H NMR (500 MHz, DMSO) δ 9.00 (d, *J* = 8.1 Hz, 1H), 8.41 (s, 1H), 8.26 (d, *J* = 8.5 Hz, 1H), 7.30 – 7.11 (m, 10H), 7.05 – 6.97 (m, 1H), 6.60 (dd, *J* = 14.8, 10.9 Hz, 1H), 6.37 (ddd, *J* = 23.8, 14.9, 11.0 Hz, 2H), 6.25 (dd, *J* = 14.9, 11.0 Hz, 1H), 6.16 (ddq, *J* = 14.9, 10.6, 1.3 Hz, 1H), 6.02 (d, *J* = 15.0 Hz, 1H), 5.84 (dq, *J* = 14.1, 6.9 Hz, 1H), 5.32 (ddd, *J* = 9.5, 8.1, 5.4 Hz, 1H), 4.65 (ddd, *J* = 9.6, 8.5, 5.1 Hz, 1H), 3.38 – 3.34 (m, 1H), 3.16 (dd, *J* = 13.9, 9.4 Hz, 1H), 2.95 (dd, *J* = 13.7, 5.0 Hz, 1H), 2.76 – 2.67 (m, 1H), 2.54 (s, 3H), 1.81 – 1.74 (m, 3H).

^{13}C NMR (126 MHz, DMSO) δ 192.0, 172.8, 171.2, 164.9, 153.8, 139.4, 139.1, 137.7, 137.4, 136.1, 132.0, 131.7, 129.9, 129.8, 129.3, 129.1, 128.2, 128.1, 127.3, 126.4, 126.3, 124.1, 53.9, 52.4, 39.9*, 37.6, 27.7, 18.3.

*: Exctracted from HSQC, due to overlap with DMSO-d6 in ^{13}C spectra.

HRMS (ESI) m/z calculated for C32H34N3O3S $[M+H]^+$: 540.2315, found 540.2312

FTIR (thin film) 3359, 3262, 2923, 2852, 1685, 1666, 1645, 1535, 1454, 1380, 1269, 1217, 1002, 793, 699

O.R. $[\alpha]_D^{24}$ = -99.3 (c = 0.1, 20% MeOH in CH_2Cl_2)

HRMS/MS Comparison for Mutanofactin

High Resolution MS/MS of synthetic mutanofactin 539 and proposed fragment structures

Acquisition Parameter

Method:	ETH_HyStar_HPLC_QTOF_POS_LowMass_Loop-AS.m			Acquisition Date:	
File Name:	D:\Data\bmax0157xx\BMAX015708.d			Operator:	
Source Type	ESI	Ion Polarity	Positive	Set Nebulizer	0.4 Bar
Focus	Active	Set Capillary	4500 V	Set Dry Heater	200 °C
Scan Begin	50 m/z	Set End Plate Offset	-500 V	Set Dry Gas	4.0 l/min
Scan End	1300 m/z	Set Collision Cell RF	200.0 Vpp	Set Divert Valve	Source

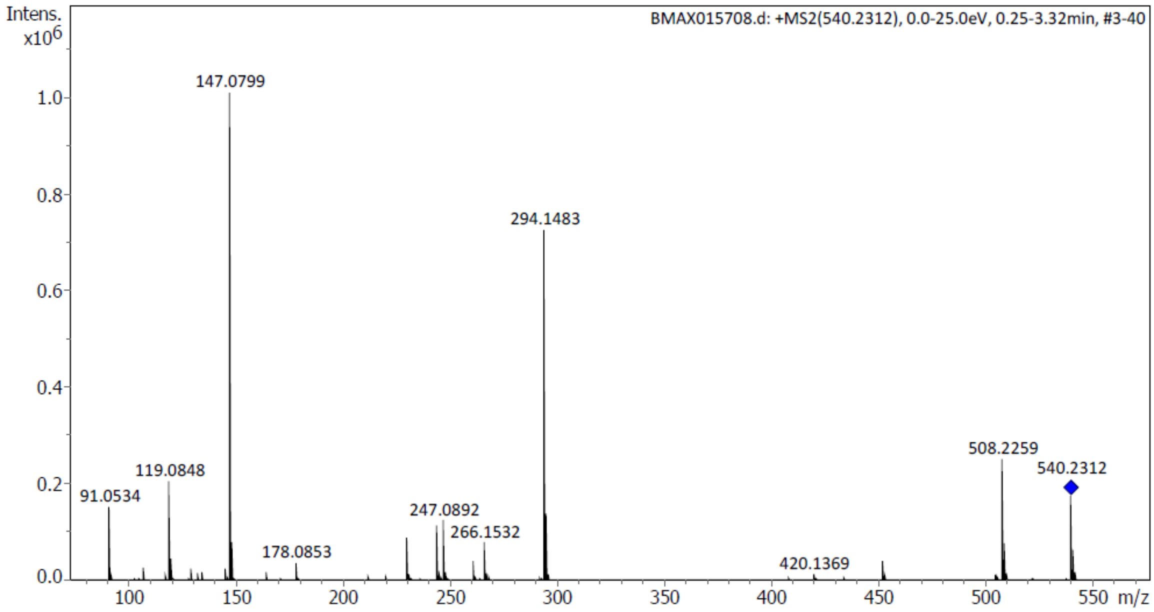

Comparison with Isolation MS-MS studies, reproduced from [23]:

Structure characterization data of mutanofactin-539 (**3**)

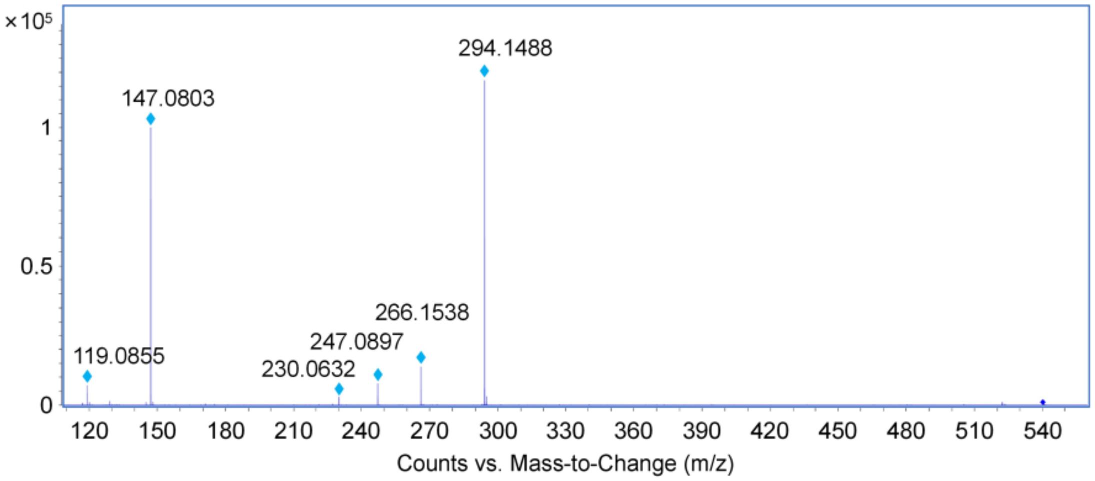

HR-MS/MS fragmentation pattern of mutanofactin-539 (**3**). Fragmentation was acquired with collision energy of 15 V.

[23] Li, Z.-R.; Sun, J.; Du, Y.; Pan, A.; Zeng, L.; Maboudian, R.; Burne, R. A.; Qian, P.-Y.; Zhang, W. Mutanofactin promotes adhesion and biofilm formation of cariogenic *Streptococcus mutans*. *Nat. Chem. Biol.* **2021**, *17* (5), 576–584. DOI: 10.1038/s41589-021-00745-2.

Proposed Fragments observed in MS-MS:

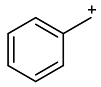

m/z
calculated: 91.0542
found: 91.0534

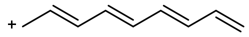

m/z
calculated: 119.0855
found: 119.0848

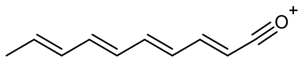

m/z
calculated: 147.0804
found: 147.0799

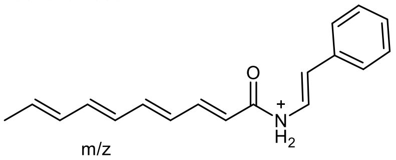

m/z
calculated: 266.1539
found: 266.1532

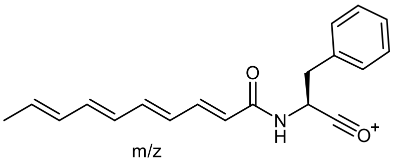

m/z
calculated: 294.1489
found: 294.1483

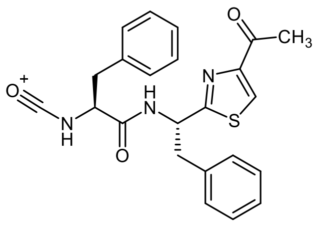

m/z
calculated: 420.1376
found: 420.1369

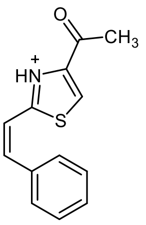

m/z
calculated: 230.0634
found: 230.0625

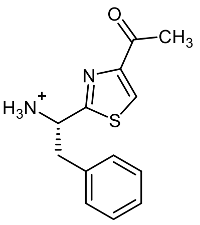

m/z
calculated: 247.0900
found:247.0892

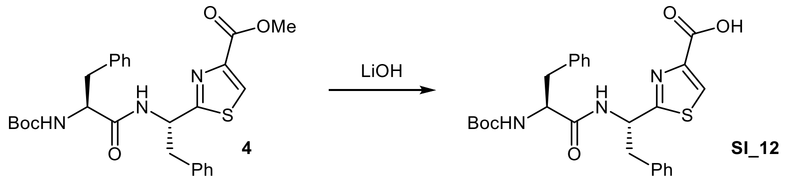

Acid SI_12: To a solution of methyl ester **4** (25.3 mg, 0.04 mmol, 1.00 equiv.) in 0.800 mL THF/MeOH/water (5:2:1) was added LiOH•H_2O (10.4 mg, 0.24 mmol, 5.00 equiv.). After stirring at room temperature for 2.5 h LC-MS indicated full consumption of starting material. After addition of Et_2O and acidification with 0.5 M $KHSO_4$, the phases were separated and the aqueous one extracted twice more with ether. The combined organic phases were dried over Na_2SO_4, filtered and concentrated in vacuo to give Acid **SI_12** as a colorless solid, which was used without further purification (23.2 mg 0.04 mmol, 94%).

Rf = 0.35 (CH_2Cl_2, 5% MeOH, 1% AcOH, UV active, stains brown with ninhydrin stain)

^{1}H NMR (400 MHz, MeOD) δ 8.21 (s, 1H), 7.31 – 7.09 (m, 10H), 5.45 (dd, *J* = 8.6, 6.1 Hz, 1H), 4.30 (dd, *J* = 8.5, 6.4 Hz, 1H), 3.41 (dd, *J* = 13.8, 6.1 Hz, 1H), 3.18 (dd, *J* = 13.8, 8.7 Hz, 1H), 2.94 (dd, *J* = 13.7, 6.3 Hz, 1H), 2.73 (dd, *J* = 13.7, 8.6 Hz, 1H), 1.36 (s, 9H).

^{13}C NMR (101 MHz, MeOD) δ 173.9, 173.6, 164.1, 157.3, 148.5, 138.3, 130.4, 130.3, 129.6, 129.5, 129.4, 129.0, 127.8, 127.7, 80.6, 57.5, 54.0, 41.5, 39.3, 28.7.

HRMS (ESI) m/z calculated for C26H30N3O5S $[M+H]^+$: 496.1901, found 496.1899

FTIR (thin film) 3339, 3127, 2980, 1684, 1654, 1519

O.R. $[\alpha]_D^{28}$ = -28.7 (c = 1.00, $CHCl_3$)

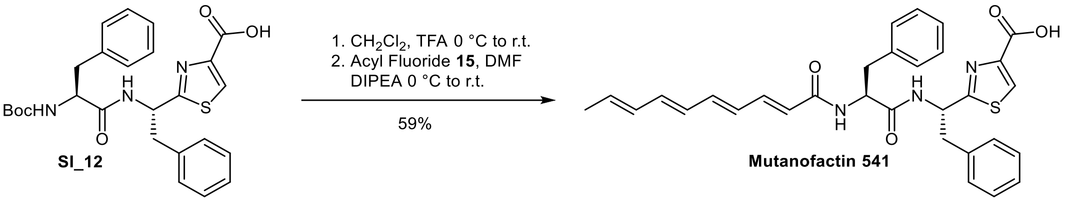

Mutanofactin 541: To a solution of Carboxylic acid **SI_12** (23.0 mg, 0.046 mmol, 1.00 equiv.) in 2.50 mL CH_2Cl_2 was added one drop of water. After cooling to 0 °C, TFA (2.50 mL) was added. The solution was slowly allowed to warm to ambient temperature, and stirring continued at room temperature for 2 hours. Volatiles were removed by concentration under a stream of N_2, the solids were redissolved in ca. 1 mL CH_2Cl_2, and again removed under a stream of N_2, the entire procedure was repeated three times with CH_2Cl_2 in total, to give a colorless solid. This material was dissolved in 0.800 mL dry DMF and cooled to 0 °C. DIPEA (50.0 µL, 0.29 mmol, 6.30 equiv.) was added, followed by the addition of Acyl fluoride **15** (18.0 mg, 0.11 mmol, 2.33 mmol) as a solution in 0.300 mL DMF, and the resulting mixture was allowed to warm to ambient temperature and stirred overnight. The resulting solution was poured into 14.0 mL chilled diethyl ether, the suspension agitated and subsequently centrifuged (3 min, 4000 rpm). The supernatant was decanted and discarded. The solids were resuspended in fresh diethyl ether, and the procedure repeated in total three times with diethyl ether. The resulting white powder was suspended in 1:1 water/MeCN and lyophilized to give desired product as a white solid (14.9 mg, 0.028 mmol, 59% over two steps).

Note (1): Similar products are known to be light-sensitive, so the product was stored in amber vials in the dark, regular maniulations (reaction, lyophilization) were carried out with minimal exposure to light.

Note (2): Alternatively, the product can also be purified via preparative HPLC, with the conditions outlined below. The material is identical with respect to analytical data (NMR-spectroscopy), and biological activity.

Retention Time: Gradient: 70% Water for 1 minute, then linear decrease to 10% Water over 22 minutes. Product elutes at 10.4 min, based on MS and UV_{330}, collected from 10.1-10.7 minutes.

^{1}H NMR (400 MHz, DMSO) δ 8.97 (d, *J* = 8.1 Hz, 1H), 8.33 (s, 1H), 8.22 (d, *J* = 8.5 Hz, 1H), 7.31 – 7.12 (m, 10H), 7.01 (dd, *J* = 14.5, 11.6 Hz 1H), 6.60 (dd, *J* = 14.8, 10.8 Hz, 1H), 6.44 – 6.31 (m, 2H), 6.33 – 6.24 (m, 1H), 6.23 – 6.11 (m, 1H), 6.00 (dd, *J* = 15.0, 1H), 5.84 (dq, *J* = 13.9, 6.9 Hz, 1H), 5.29 (ddd, *J* = 9.8, 8.2, 5.0 Hz, 1H), 4.63 (td, *J* = 9.2, 5.0 Hz, 1H), 3.36 (dd, *J* = 14.0, 4.9 Hz, 1H), 3.14 (dd, *J* = 14.0, 9.8 Hz, 1H), 2.94 (dd, *J* = 13.8, 5.0 Hz, 1H), 2.81 – 2.65 (m, 1H), 1.79 – 1.72 (d, *J* = 6.9 Hz, 3H).

^{13}C NMR (101 MHz, DMSO) δ 172.7, 171.2, 164.9, 162.2, 139.4, 139.1, 137.7, 137.5, 136.1, 132.0, 131.8, 129.9, 129.8, 129.2, 129.1, 128.5, 128.4, 128.2, 128.0, 126.4, 126.3, 124.1, 53.9, 52.4, 39.0,* 37.6, 18.3.

*: Extracted from HSQC spectra, due to overlap with DMSO-d6 in ^{13}C-NMR

HRMS (ESI) m/z calculated for C31H32N3O4S $[M+H]^+$: 542.2108, found: 542.2100

FTIR (thin film) 3279, 3027, 2925, 1646, 1496, 1220

O.R. $[a]_D^{27}$ = 60.0 (c = 0.1, 10:1 MeCN/DMSO)

HRMS/MS Comparison for Mutanofactin 541

High Resolution MS/MS of synthetic mutanofactin 541 and proposed fragment structures:

Acquisition Parameter

Method:	ETH_HyStar_HPLC_QTOF_POS_LowMass_Loop-AS.m			Acquisition Date:			
File Name:	D:\Data\bmax0157xx\BMAX015707.d			Operator:			
Source Type	ESI	Ion Polarity	Positive	Set Nebulizer	0.4 Bar		
Focus	Active	Set Capillary	4500 V	Set Dry Heater	200 °C		
Scan Begin	50 m/z	Set End Plate Offset	-500 V	Set Dry Gas	4.0 l/min		
Scan End	1300 m/z	Set Collision Cell RF	200.0 Vpp	Set Divert Valve	Source		

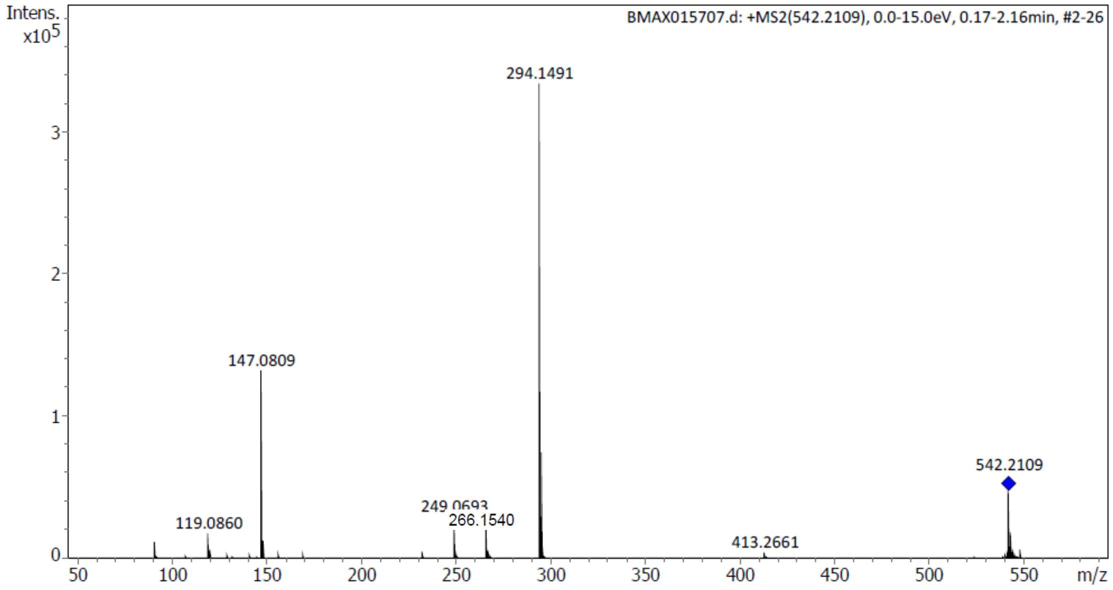

Comparison with Isolation MS-MS studies, reproduced from [24]:

Structure characterization data of mutanofactin-541 (**2**)

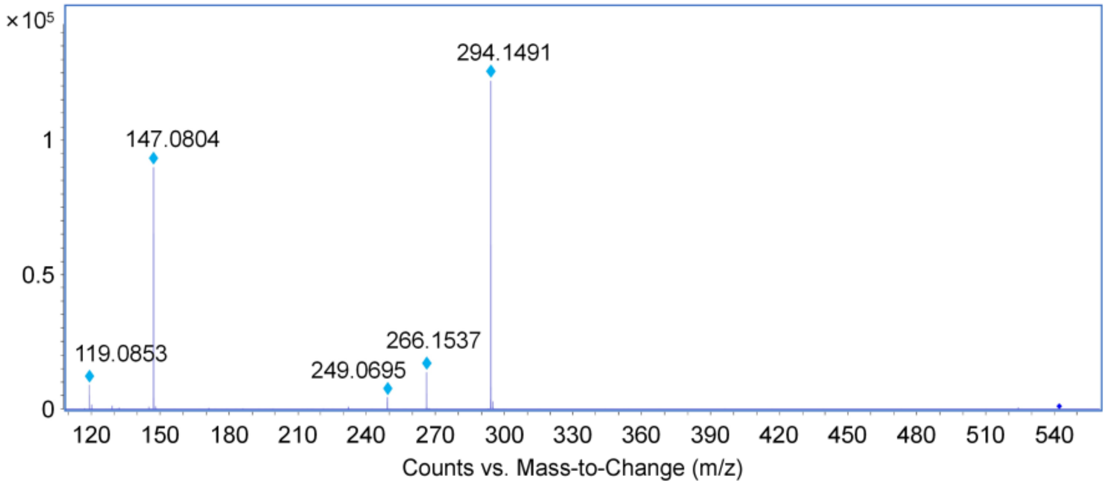

HR-MS/MS fragmentation pattern of mutanofactin-541 (**2**). Fragmentation was acquired with collision energy of 15 V.

[24] Li, Z.-R.; Sun, J.; Du, Y.; Pan, A.; Zeng, L.; Maboudian, R.; Burne, R. A.; Qian, P.-Y.; Zhang, W. Mutanofactin promotes adhesion and biofilm formation of cariogenic *Streptococcus mutans*. *Nat. Chem. Biol.* **2021**, *17* (5), 576–584. DOI: 10.1038/s41589-021-00745-2.

Propsed fragments in MS-MS:

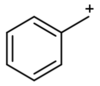

m/z
calculated: 91.0542
found: 91.0546

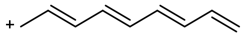

m/z
calculated: 119.0855
found: 119.0860

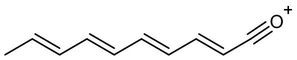

m/z
calculated: 147.0804
found: 147.0809

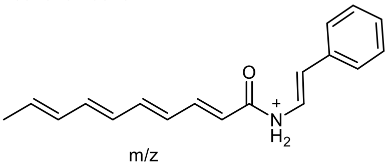

m/z
calculated: 266.1539
found: 266.1540

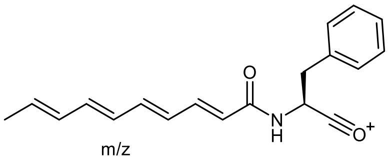

m/z
calculated: 294.1489
found: 294.1491

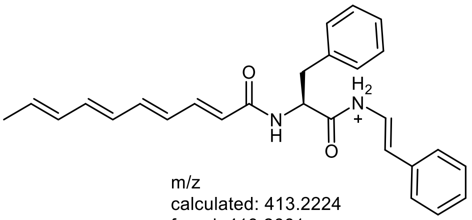

m/z
calculated: 413.2224
found: 413.2661

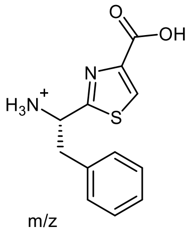

m/z
calculated: 249.0692
found: 249.0693

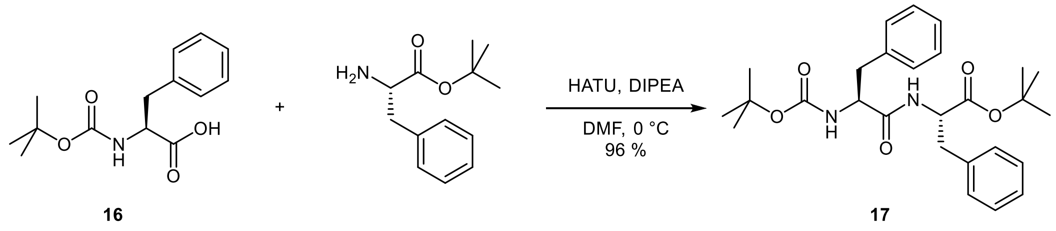

Boc-Phe-Phe-*t*Bu 17: Boc-Phe-OH **16** (2.00 g, 7.54 mmol, 1.00 equiv) and H-Phe-O*t*Bu•HCl (2.43 g, 9.42 mmol, 1.25 equiv) were added to a dried 100 mL round bottom flask equipped with a stirring bar and set under N_2. 20.0 mL DMF was added and the resulting suspension cooled to 0 °C. DIPEA(1) (3.91 mL, 22.6 mmol, 3.00 equiv) was added and the mixture stirred until a clear solution was obtained (ca. 5 minutes). HATU (3.58 g, 9.42 mmol, 1.25 equiv) was added at once, and the resulting yellow solution stirred for 16 hours while slowly warming to ambient temperature. The reaction was poured into water, diluted with EtOAc, the phases separated and the aqueous phase extracted four times with EtOAc. The combined organic phases were washed sequentially with NH_4Cl, $NaHCO_3$, 5% LiCl solution and brine. The organic phase was dried over Na_2SO_4, filtered through cotton and evaporated to a crude. Purification by column chromatography (Hexane/EtOAc) gave dipeptide **17** as a white, crystalline powder. (3.40 g, 7.26 mmol, 96 %)

Note (1): DIPEA was freshly distilled from CaH_2 under N_2 atmosphere before use.

Rf = 0.86 (50% EtOAc/Hexanes, UV-active, ninhydrin red stain)

^{1}H NMR (400 MHz, DMSO) δ 8.23 (d, *J* = 7.5 Hz, 1H), 7.33 – 7.13 (m, 10H), 6.86 (d, *J* = 8.8 Hz, 1H), 4.37 (q, *J* = 7.3 Hz, 1H), 4.19 (ddd, *J* = 10.5, 8.7, 3.9 Hz, 1H), 2.98 (dd, *J* = 7.2, 2.4 Hz, 2H), 2.92 (dd, *J* = 13.8, 4.0 Hz, 1H), 2.69 (dd, *J* = 13.8, 10.6 Hz, 1H), 1.31 (s, 9H), 1.28 (s, 9H).

^{13}C NMR (101 MHz, DMSO) δ 171.7, 170.4, 155.15, 138.1, 137.1, 129.3, 129.2, 128.2, 128.0, 126.5, 126.2, 80.7, 78.0, 55.5, 54.1, 37.4, 36.9, 28.1, 27.5

HRMS (ESI) m/z calculated for C27H36N2NaO5 $[M+Na]^+$: 491.2516, found: 491.2506

FTIR (thin film) 3301, 3030, 2978, 2924, 2853, 1732, 1655, 1605, 1522, 1455, 1392, 1250, 1224, 1022, 887, 754, 699

O. R. $[a]_D^{25}$ = 32.4 (c = 1.0, $CHCl_3$)

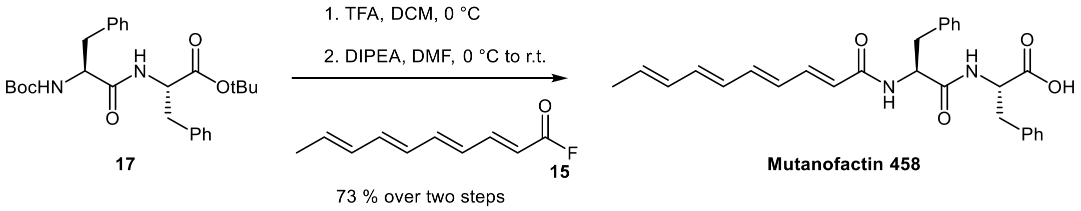

Mutanofactin 458: Dipeptide **17** (50.0 mg, 0.107 mmol, 1.00 equiv) was added to a 10.0 mL schlenk flask and dissolved in 1.20 mL CH_2Cl_2. Anisole (0.0580 mL, 0.534 mmol, 5.00 equiv) was added, and the mixture cooled to 0 °C using an ice-water bath. TFA (0.820 mL, 10.7 mmol, 100.0 equiv) was added dropwise and the resulting mixture slowly warmed to ambient temperature. Upon stirring for 6 hours the deprotection was complete, accordingly volatiles were removed under a stream of dry Nitrogen. CH_2Cl_2 (ca. 1 mL) was added and again removed under a stream of nitrogen, this procedure was repeated three times to remove residual TFA. The crude solid was dried under high vacuum for 5 minutes, then dissolved in 1.20 mL DMF and cooled to 0 °C using an ice-water bath. Sequentially, DIPEA(1) (0.092 mL, 0.534 mmol, 5.00 equiv) and Acyl Fluoride **15** (35.5 mg, 0.213 mmol, 2.0 equiv, as a solution in 0.200 mL DMF) was added. The resulting yellow solution was stirred overnight, while slowly warming to ambient temperature. After 16 hours of stirring, 3-5 drops of each MeOH and Formic Acid were added, and the resulting solution directly purified by preparative HPLC. Lyophilization gave Mutanofactin 458 as a white powder (35.8 mg, 0.078 mmol, 73% yield)(2).

Note (1): DIPEA was freshly distilled from CaH_2 under N_2 atmosphere before use.

Note (2): Similar products are known to be light-sensitive, so the product was stored in amber vials in the dark, regular manipulations (reaction, lyophilization) were carried out with minimal exposure to light.

Retention Time: Gradient: 60% Water for 1 minute, then linear decrease to 20% Water over 18 minutes. Product elutes at 9.5 min, based on MS and UV_{330}, collected from 9.0-10.0 minutes.

^{1}H NMR (500 MHz, DMSO) δ 12.76 (s, 1H), 8.30 (d, *J* = 7.8 Hz, 1H), 8.20 (d, *J* = 8.7 Hz, 1H), 7.28 – 7.10 (m, 10H), 6.98 (ddd, *J* = 15.1, 11.5, 0.7 Hz, 1H), 6.59 (dd, *J* = 14.8, 10.9 Hz, 1H), 6.36 (ddd, *J* = 24.1, 14.8, 11.0 Hz, 2H), 6.24 (ddt, *J* = 15.0, 11.0, 0.8 Hz, 1H), 6.19 – 6.12 (m, 1H), 6.01 (d, *J* = 15.0 Hz, 1H), 5.84 (dq, *J* = 14.1, 6.9 Hz, 1H), 4.63 (ddd, *J* = 10.3, 8.6, 4.1 Hz, 1H), 4.45 (ddd, *J* = 8.7, 7.8, 5.2 Hz, 1H), 3.07 (dd, *J* = 13.9, 5.2 Hz, 1H), 3.01 (dd, *J* = 14.0, 4.1 Hz, 1H), 2.93 (dd, *J* = 13.9, 8.7 Hz, 1H), 2.72 (dd, *J* = 14.0, 10.3 Hz, 1H), 1.7 (dd, *J* = 6.9, 1.6 Hz, 3H).

^{13}C NMR (126 MHz, DMSO-d6) δ 172.7, 171.4, 164.9, 139.4, 139.1, 138.0, 137.4, 136.1, 132.1, 131.8, 129.9, 129.8, 129.2, 129.2, 128.2, 128.0, 126.4, 126.2, 124.0, 53.7, 53.5, 37.4, 36.6, 18.3.

HRMS (ESI) m/z calculated for C28H31N2O4 $[M+H]^+$: 459.2278, found 459.2276

FTIR (thin film) 3379, 3295, 2927, 2861, 1454, 1323, 1095, 1029, 976, 947, 883, 617

O. R. $[a]_D^{25}$ = -10.7 (c = 0.15, 9:1 CH_2Cl_2/MeOH)

HRMS/MS Comparison for Mutanofactin 458

High resolution MS/MS of synthetic mutanofactin 458 and proposed fragment structures:

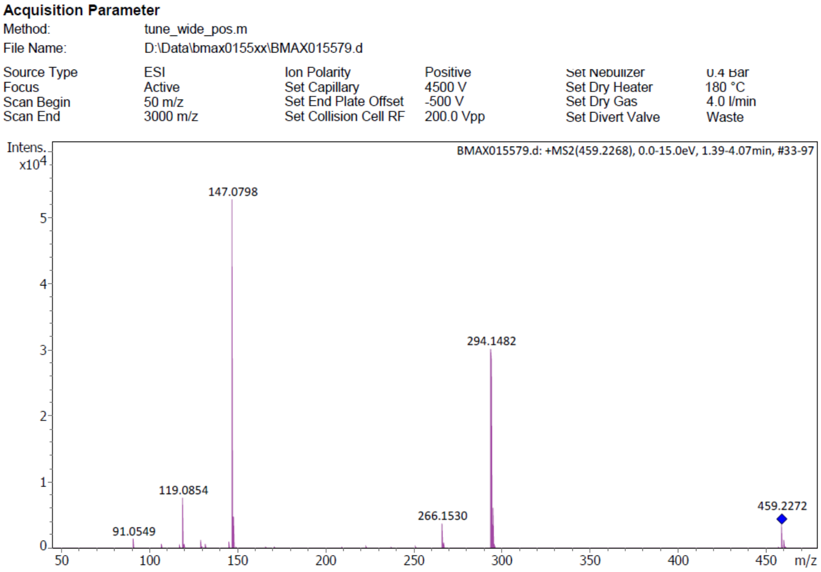

Comparison with Isolation MS-MS studies, reproduced from [25]:

Structure characterization data of mutanofactin-458 (**1**)

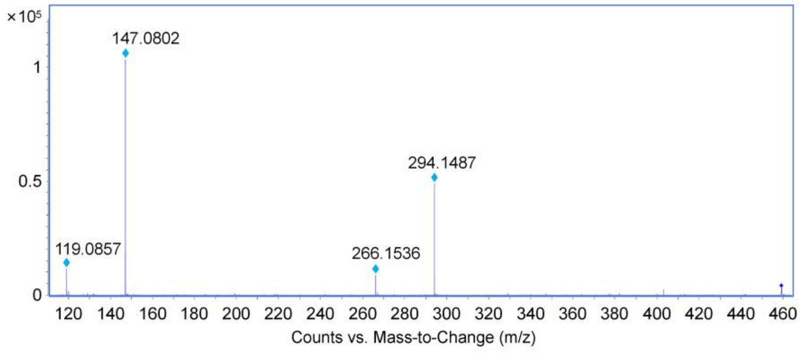

HR-MS/MS fragmentation pattern of mutanofactin-458 (**1**). Fragmentation was acquired with collision energy of 15 V.

Proposed Fragments for MS-MS:

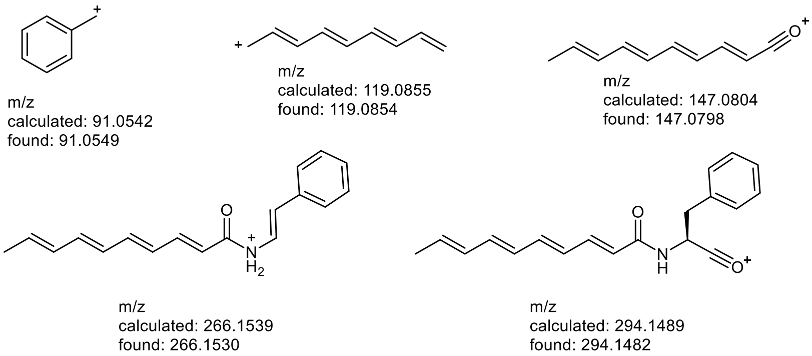

[25] Li, Z.-R.; Sun, J.; Du, Y.; Pan, A.; Zeng, L.; Maboudian, R.; Burne, R. A.; Qian, P.-Y.; Zhang, W. Mutanofactin promotes adhesion and biofilm formation of cariogenic *Streptococcus mutans*. *Nat. Chem. Biol.* **2021**, *17* (5), 576–584. DOI: 10.1038/s41589-021-00745-2.

4 Microbiology

Bacterial Growth Conditions

Bacteria used in this study are listed in Table S1. Unless stated otherwise, incubation was carried out at 37 °C without agitation, in a 5%-CO_2 atmosphere for *S. gordonii*, *S. mutans*, and *S. oralis,* or an anaerobic atmosphere for *F. nucleatum* and *V. dispar*. Prior to growth in liquid culture, bacteria were streaked out from cryo-stocks (25% glycerol) onto Brain Heart Infusion (BHI) plates and incubated for a minimum of 48 hours. Single colonies were picked and inoculated into eprouvettes containing 5 mL of medium and grown overnight. Liquid medium was BHI for the streptococci, BHI supplemented with 5.0 g/L yeast extract, 0.5 g/L L-cysteine, 2.0 mg/L hemine, and 2.0 mg/L menadione for *F. nucleatum,*[26] and additional 0.5% sodium lactate for *V. dispar*.[27]

Biofilm formation of *S. gordonii*, *S. mutans*, and *S. oralis* was tested using a semi-defined biofilm medium (BM) originating based on the work of Carlsson,[28] Jenkinson[29] and modified glucose concentration according to Burne *et al*.[30]

Accordingly, the BM contained 58 mM K_2HPO_4, 15 mM KH_2PO_4, 10 mM $(NH_4)_2SO_4$, 35 mM NaCl, 20 mM D-glucose, 0.2% casamino acids, 100 µM $MnCl_2{\cdot}4H_2O$ and was set to pH 7.4 with HCl prior to addition of amino acids (1 mM L-arginine-HCl, 1.3 mM L-cysteine-HCl, 4 mM L-glutamic acid, 100 µM L-tryptophan), vitamins (0.05 µM biotin, 10 µM calcium pantothenate, 40 µM niacin, 100 µM pyridoxine-HCl, 1 µM riboflavin, 0.3 µM thiamine-HCl), and 2 mM $MgSO_4 \cdot 7H_2O$. All supplements were filter-sterilized. Biofilm formation of *F. nucleatum* and *V. dispar* was tested in BHI added with the bacteria specific supplements.

---

[26] Tomek, M. B.; Neumann, L.; Nimeth, I.; Koerdt, A.; Andesner, P.; Messner, P.; Mach, L.; Potempa, J. S.; Schäffer, C. The S-layer proteins of Tannerella forsythia are secreted via a type IX secretion system that is decoupled from protein O-glycosylation. *Mol. Oral Microbiol.* **2014**, *29* (6), 307–320. DOI: https://doi.org/10.1111/omi.12062.

[27] Li, J.; Wang, H.; Li, N.; Zhang, Y.; Lü, X.; Liu, B. Antibiotic susceptibility and biofilm-forming ability of Veillonella strains. *Anaerobe* **2022**, *78*, 102667. DOI: https://doi.org/10.1016/j.anaerobe.2022.102667.

[28] Carlsson, J. Nutritional requirements of *Streptococcus sanguis*. *Arch. Oral Biol.* **1972**, *17* (9), 1327–1332. DOI: https://doi.org/10.1016/0003-9969(72)90165-3.

[29] Jenkinson, H. F. Cell-surface Proteins of Streptococcus sanguis Associated with Cell Hydrophobicity and Coaggregation Properties. *Microbiology* **1986**, *132* (6), 1575–1589. DOI: https://doi.org/10.1099/00221287-132-6-1575.

[30] Palmer, S. R.; Miller, J. H.; Abranches, J.; Zeng, L.; Lefebure, T.; Richards, V. P.; Lemos, J. A.; Stanhope, M. J.; Burne, R. A. Phenotypic Heterogeneity of Genomically-Diverse Isolates of *Streptococcus mutans*. *PLOS ONE* **2013**, *8* (4), e61358. DOI: 10.1371/journal.pone.0061358.

Table S1. Bacteria used in this study.

Species	Strain & ATCC No.*	Mutation	Reference
Fusobacterium nucleatum subsp. *nucleatum*	KP-F8		Turnheer *et al*[31]
Streptococcus gordonii	SK3 (ATCC 10558)		Kilian *et al*[32]
Streptococcus mutans	NMT4863		Cornejo *et al*[33]
Streptococcus mutans	NMT4863 Δ*mufC-J*	Δ*mufCDEFGHIJ*::*kan*	this study
Streptococcus mutans	NMT4863 Δ*mufD-G*	Δ*mufDEFG*::*kan*	this study
Streptococcus mutans	U2A		Cornejo *et al*[33]
Streptococcus mutans	UA159 (ATCC 700610)		Ajdić *et al*[34]
Streptococcus oralis	SK248		Guggenheim *et al*[35]
Veillonella dispar	ERN (ATTC 17748)		Mays *et al*[37]

* American Type Culture Collection (https://www.atcc.org/)

Among the investigated strains, only *S. mutans* NMT4863 is known to possess the BGC encoding mutanofactin biosynthesis According to a BLAST analysis of mutanofactin genes, no strains of *F. nucleatum* or *S. oralis* are predicted to code for mutanofactin biosynthesis. Notably, due to a lack of available genome sequences, no antiSMASH analysis could be performed which would have resulted in more reliable data.

Mutanofactin Stock solutions

Synthetic mutanofactins were stored as lyophilized powders at –80 °C until first usage. Stock solutions were prepared in DMSO at a final concentration of 10 mM and stored in aliquots at –80 °C. Mutanofactin 539 is only sparingly soluble, so the stock solution was prepared at a concentration of 5 mM.

---

[31] Thurnheer, T.; Guggenheim, B.; Gruica, B.; Gmür, R. Infinite Serovar and Ribotype Heterogeneity Among Oral Fusobacterium nucleatum Strains? *Anaerobe* **1999**, *5*, 79-92. DOI: 10.1006/anae.1999.0188.

[32] Kilian, M.; Mikkelsen, L.; Henrichsen, J. Taxonomic Study of Viridans Streptococci: Description of Streptococcus gordonii sp. nov. and Emended Descriptions of Streptococcus sanguis (White and Niven 1946), Streptococcus oralis (Bridge and Sneath 1982), and Streptococcus mitis (Andrewes and Horder 1906). *International Journal of Systematic and Evolutionary Microbiology* **1989**, *39*, 471-484. DOI: 10.1099/00207713-39-4-471.

[33] Cornejo, O. E.; Lefébure, T.; Pavinski Bitar, P. D.; Lang, P.; Richards, V. P.; Eilertson, K.; Do, T.; Beighton, D.; Zeng, L.; Ahn, S.-J.; et al. Evolutionary and Population Genomics of the Cavity Causing Bacteria *Streptococcus mutans*. *Molecular Biology and Evolution* **2012**, *30*, 881-893. DOI: 10.1093/molbev/mss278.

[34] Ajdić, D.; McShan, W. M.; McLaughlin, R. E.; Savić, G.; Chang, J.; Carson, M. B.; Primeaux, C.; Tian, R.; Kenton, S.; Jia, H.; et al. Genome sequence of *Streptococcus mutans* UA159, a cariogenic dental pathogen. *Proc. Natl. Acad. Sci. U.S.A.* **2002**, *99*, 14434-14439. DOI: 10.1073/pnas.172501299.

[35] Thurnheer, T.; Gmür, R.; Shapiro, S.; Guggenheim, B. Mass Transport of Macromolecules within an In Vitro Model of Supragingival Plaque. *Appl. Environ. Microbiol.* **2003**, *69* (3), 1702-1709. DOI: 10.1128/AEM.69.3.1702-1709.2003 (acccessed 2025/02/25).

[37] Mays, T. D.; Holdeman, L. V.; Moore, W. E. C.; Rogosa, M.; Johnson, J. L. Taxonomy of the Genus Veillonella Prévot. *International Journal of Systematic and Evolutionary Microbiology* **1982**, *32*, 28-36. DOI: 10.1099/00207713-32-1-28.

Construction of *S. mutans* NMT4863 Δ*mufD-G* and Δ*mufC-J*

The genomic regions of interest were deleted by homologous recombination with an antibiotic resistance gene using the protocols outlined by Petersen *et al*.[38] Unless stated otherwise, chromosomal DNA of *S. mutans* NMT4863 was used as a template for PCR amplifications; oligonucleotide primers (Thermo Fisher Scientific) are listed in Table S2.

For the construction of a *S. mutans* NMT4863 Δ*mufDEFG* (Δ*mufD-G*) deletion mutant, allelic replacement was conducted using a kanamycin-resistance cassette (aminoglycoside phosphotransferase, *kan*; 816bp). The size and region of the gene deletion is based on the work by Li and coworkers,[39] and was chosen for this study for best comparability and for its mutanofactin-deficiency. Briefly, the primer pairs *mufD-G*_up_F/R and *mufD-G*_down_F/R with an incorporated *AscI* and *NotI* restriction site, respectively, were used to PCR amplify up- and downstream fragments (~1-kbp) flanking the deletion region. The *kan* gene was amplified from the pET-28a vector (Novagen) using the primer pair Kan_F/Kan_R containing the same restriction sites. Subsequently, the up- and downstream fragments were fused to the kanamycin-resistance gene by restriction and ligation (ligation mixture).

The *S. mutans* NMT4863 Δ*mufCDEFGHIJ* (Δ*mufC-J*) deletion mutant, additional lacking the transcriptional regulator gene *mufC* and the three transporter genes *mufH*, *mufI*, and *mufJ*, was created as described for *S. mutans* NMT4863 Δ*mufD-G* except for the use of the primers *mufC-J*_up_F/R and *mufC-J*_down_F/R to amplify ~1-kbp PCR fragments of the up- and downstream region flanking the target region.

For transformation, an overnight-culture of *S. mutans* NMT4863 grown in BHI medium was inoculated into fresh BHI medium and grown to the mid-exponential growth phase (OD_{600} ~0.6). This culture was then inoculated into chemically-defined medium,[40] and grown to an optical density at 600 nm (OD_{600}) of ~0.1. Subsequently, 1 mL of the culture was transferred into a 1.5 mL tube and synthetic SigX-inducing peptide (sXIP, amino acid sequence: GLDWWSL) was added at a final concentration of 1 µM.[27] Cells were incubated at 37 °C for ~10 minutes before addition of the ligation mixture. The cells were allowed to grow for 3 hours at 37 °C and subsequently, the culture was spun for 1 min at 7,000 rpm, and 900 µL of the supernatant was discarded. The cell pellet was dissolved in the remaining medium (~100 µL) and plated onto BHI agar plates containing 300 µg/mL of kanamycin.

The *S. mutans* NMT4863 Δ*mufD-G* and Δ*mufC-J* deletion mutants were confirmed by colony PCR and DNA sequencing (Microsynth), using the primer pairs *mufD-G*_seq_F/R and *mufC-J*_seq_F/R (Table S2), respectively.

---

[38] Salvadori, G.; Junges, R.; Khan, R.; Åmdal, H. A.; Morrison, D. A.; Petersen, F. C. Natural Transformation of Oral Streptococci by Use of Synthetic Pheromones. In *Oral Biology: Molecular Techniques and Applications*, Seymour, G. J., Cullinan, M. P., Heng, N. C. K. Eds.; Springer New York, 2017; pp 219–232.
[39] Li, Z.-R.; Sun, J.; Du, Y.; Pan, A.; Zeng, L.; Maboudian, R.; Burne, R. A.; Qian, P.-Y.; Zhang, W. Mutanofactin promotes adhesion and biofilm formation of cariogenic *Streptococcus mutans*. *Nat. Chem. Biol.* **2021**, *17* (5), 576–584. DOI: 10.1038/s41589-021-00745-2.
[40] van de Rijn, I.; Kessler, R. E. Growth characteristics of group A streptococci in a new chemically defined medium. *Infect. Immun.* **1980**, *27* (2), 444–448. DOI: 10.1128/iai.27.2.444-448.1980 (acccessed 2024/11/27).

Table S2. Oligonucleotide primers used in this study.

Name	Nucleotide Sequence (5’→3’)	Restriction site*
*mufD-G*_up_F	CTCGATGCAATTGTTTCAGTTCTTAATTCCTCAAAAATCT	-
*mufD-G*_up_R	GCTATGGCGCGCCGACTTACTTTCTCTTTCGATTAT	*AscI*
*mufD-G*_down_F	CGATTGCGGCCGCTATCTGATTATTTAAATCATACTCACAC	*NotI*
*mufD-G*_down_R	CATATGTCTGTATTTCCTGTTTTTCCATAGCAGATAAAATTTTAGA	-
*mufC-J*_up_F	GAATGGGAAATACTATAGAAGATACC	-
*mufC-J*_up_R	GCTATGGCGCGCCCATCTCTTGCCATAATTACCTC	*AscI*
*mufC-J*_down_F	CGATTGCGGCCGCCACTTATAGTACTTGTAGTGTATAC	*NotI*
*mufC-J*_down_R	ATGAAAGGCAATGCCAGTCACG	-
Kan_F	GCTATGGCGCGCCCAAGGGGTGTTATGAGCCA	*AscI*
Kan_R	CGATTGCGGCCGCCCGCTCATGAATTAATTCTTAG	*NotI*
*mufC-J*_seq_F	GTTCATATATAGTTATCACTTTGA	-
*mufC-J*_seq_R	GATATTATTCATCTACTCTTCG	-
*mufD-G*_seq_F	CAGCTAGCAAAGCCCGAGTTTAT	-
*mufD-G*_seq_R	TCTCCACCTAACTCATAAATGTTACC	-

*Restriction sites are underlined.

Biofilm growth assays

Biofilm growth was determined based on Li *et al.*[41], with modifications. 100 µL of an overnight-culture were inoculated into 5 mL of fresh BHI medium and upon reaching the mid-exponential growth phase (OD600 ~0.5), this pre-culture was used as an inoculum (1:70) for the biofilm assay. For *V. dispar*, the assay was inoculated directly from the overnight-culture not using a pre-culture.[42]

To develop biofilms, bacterial cultures were grown in TC-treated 24 well plates (CC7682-7524, Starlab), with 1 mL of growth medium per well. BM and BHI including supplements were used for the streptococci and for *F. nucleatum* and *V. dispar*, respectively. The medium was added *ab initio* with mutanofactins at different concentrations, using the DMSO stock solutions. The amount of DMSO was kept constant at 0.2% for all tested concentration ranges and for the control without mutanofactin, with the exception of Muf-539 at 20 µM, because of the halved stock concentration (see above).

The bacteria were grown statically for 24 hours and then measured for total biomass using a Tecan Infinite F200 microplate reader at 595 nm, taking a total of 25 different points (5 by 5 grid) per well to cover a maximum surface area. The medium was then aspirated using a vacuum pump and each well was gently washed once (*S. gordonii*, *S. oralis*, and *V. dispar*) or twice (*S. mutans* and *F. nucleatum*) with 1 mL sterile PBS. After washing, the biofilms were fixed for 1 hour at 60 °C,[43] and then stained with 500 µL of 0.1% crystal violet for 15 minutes at room temperature. To remove excess crystal violet, the wells were washed twice with 1 mL of Milli-Q water, air-dried and photographed using a light pad. For quantification, crystal violet was mobilized in 1 mL 30% acetic acid per well,[44] thoroughly mixed and 200 µL were transferred into a fresh 96-well plate (655101, Greiner). The absorption was measured at 595 nm in the Tecan microplate reader and diluted with Milli-Q water if necessary (OD values above 1).

[41] Li, Z.-R.; Sun, J.; Du, Y.; Pan, A.; Zeng, L.; Maboudian, R.; Burne, R. A.; Qian, P.-Y.; Zhang, W. Mutanofactin promotes adhesion and biofilm formation of cariogenic *Streptococcus mutans*. *Nat. Chem. Biol.* **2021**, *17* (5), 576–584. DOI: 10.1038/s41589-021-00745-2.

[42] Li, J.; Wang, H.; Li, N.; Zhang, Y.; Lü, X.; Liu, B. Antibiotic susceptibility and biofilm-forming ability of Veillonella strains. *Anaerobe* **2022**, *78*, 102667. DOI: https://doi.org/10.1016/j.anaerobe.2022.102667.

[43] Baldassarri, L.; Simpson, W. A.; Donelli, G.; Christensen, G. D. Variable fixation of staphylococcal slime by different histochemical fixatives. *Eur. J. Clin. Microbiol. Infect. Dis.* **1993**, *12* (11), 866–868. DOI: 10.1007/BF02000411.

[44] Stepanović, S.; Vuković, D.; Dakić, I.; Savić, B.; Švabić-Vlahović, M. A modified microtiter-plate test for quantification of staphylococcal biofilm formation. *J. Microbiol. Methods* **2000**, *40* (2), 175–179. DOI: https://doi.org/10.1016/S0167-7012(00)00122-6.

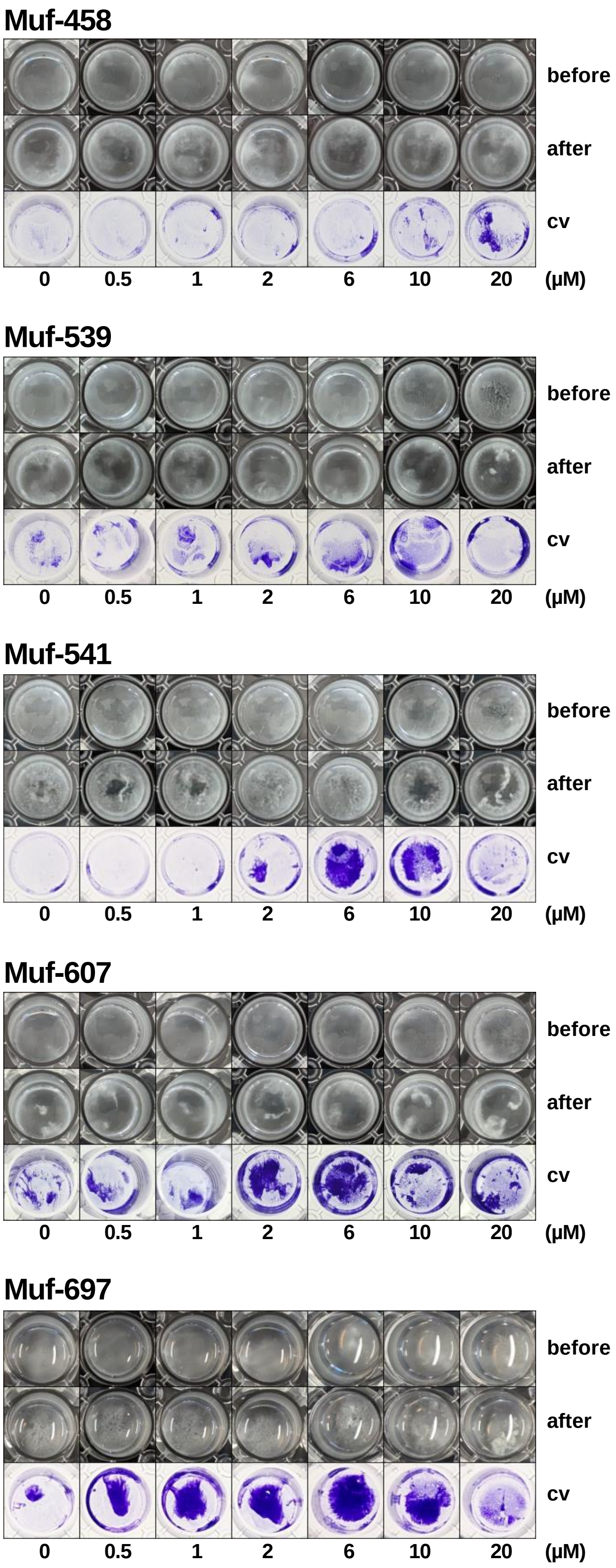

Figure S1. Images of typical *S. mutans* Δ*mufD-G* biofilms grown for 24 hours at 0-20 µM of the different mutanofactins. Images were taken before (before) and after (after) measurement in the microplate reader and upon the crystal violet staining (cv).

Washing assay

The *S. mutans* NMT4863 wild-type and Δ*mufD-G* were grown as above ("Biofilm growth assays"), using BM medium with 0.2% DMSO. The total biomass measurement in the microplate reader was skipped to reduce the amount of mechanical stress on the biofilms. The number of PBS washing steps was altered to four different washing conditions (0-3 washing steps, each compromising of rinsing the biofilm with 1 mL sterile PBS and subsequent aspiration by vacuum pump). All following steps, i.e. heat fixation of the biofilms and crystal violet staining were performed as described in the biofilm assay protocol.

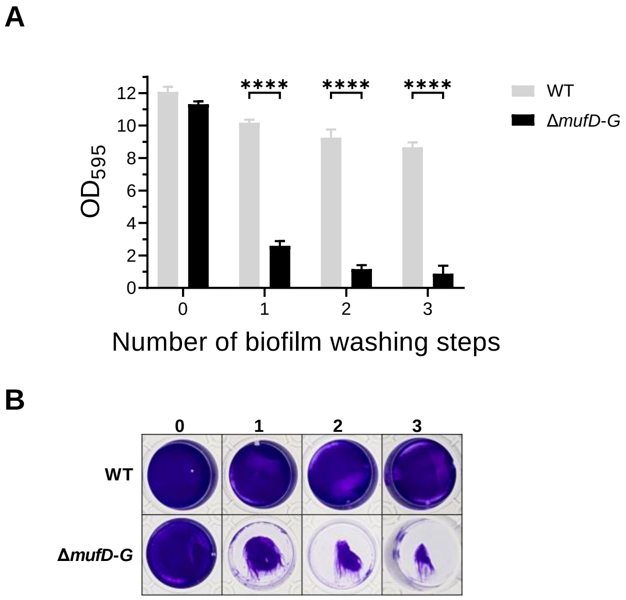

Figure S2. Washing of biofilm revealing the difference in biofilm stability and/ or attachment between the *S. mutans* NMT4863 wild-type (WT) and the Δ*mufD-G* deletion mutant. The biofilms were grown for 24 hours and then washed with sterile PBS one- to three-times (1, 2, 3) or not washed at all (0) before being quantified by crystal violet staining (A) and being photographed (B). Data are shown as the mean and SD of three to four biological replicates (n = 3) tested in three technical replicates. Significance was analyzed using a two-way ANOVA with Šídák's multiple comparisons test for pairwise comparisons (**** = $p \leq 0.0001$).

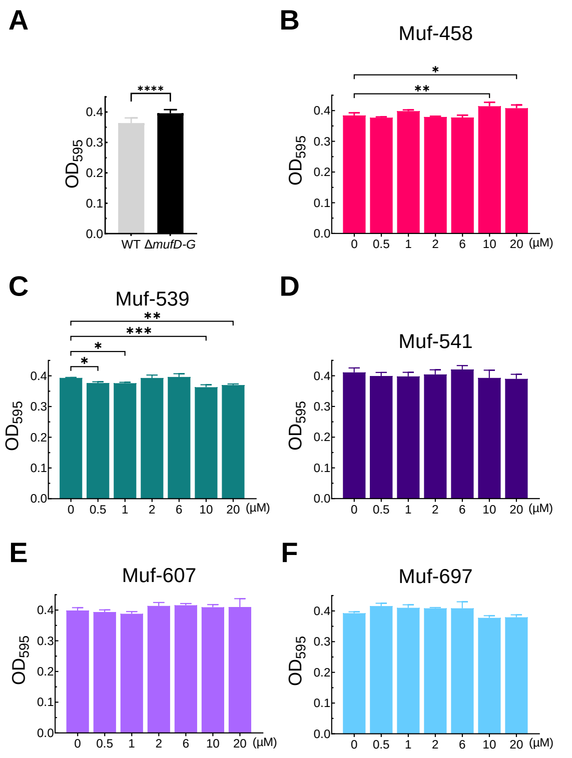

Figure S3. Total biomass of *S. mutans* NMT4863 wild-type and Δ*mufD-G* without addition of mutanofactin (A) and of Δ*mufD-G* grown at 0-20 µM of mutanofactins (B-F). Data are shown as the mean and SD of at least three biological replicates ($n \geq 3$) tested in three technical replicates. Significance was tested using (A) a two-sample t-test or (B) a one-way ANOVA for each mutanofactin with Dunnett's test against the 0-µM condition (* = $p \leq 0.05$, ** = $p \leq 0.01$, *** = $p \leq 0.001$).

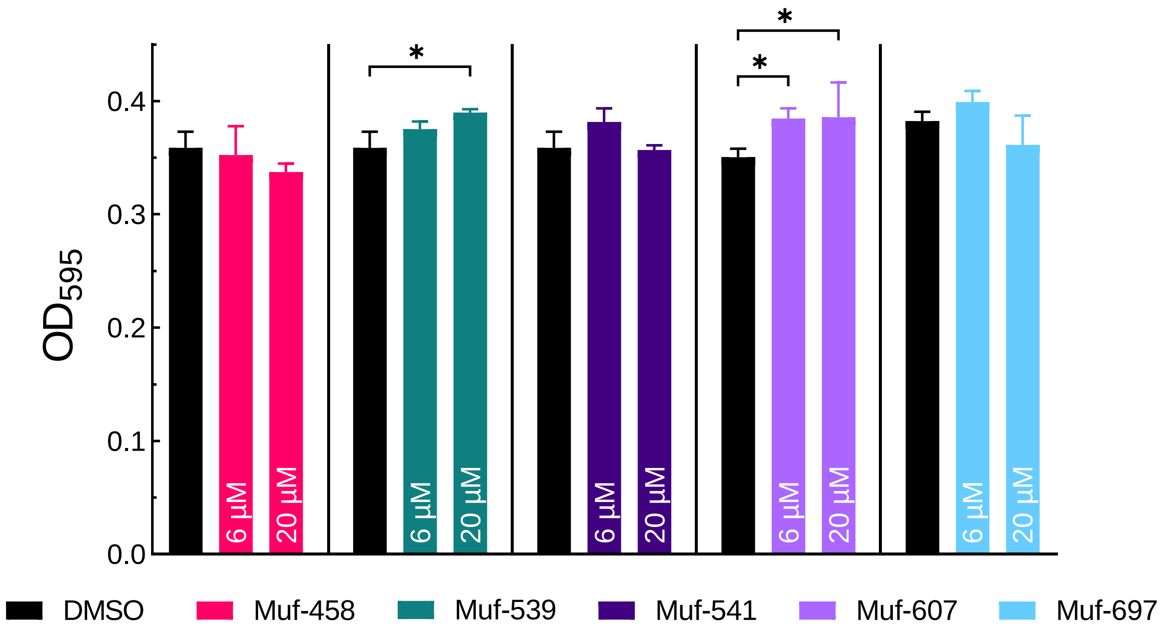

Figure S4. Total biomass of *S. mutans* NMT4863 wild-type upon addition of 6 µM and 20 µm of mutanofactins. Data are shown as the mean and SD of three biological replicates ($n = 3$) tested in three technical replicates. Significance was tested using a two-way ANOVA with Dunnett's test against the DMSO control (* = $p \leq 0.05$).

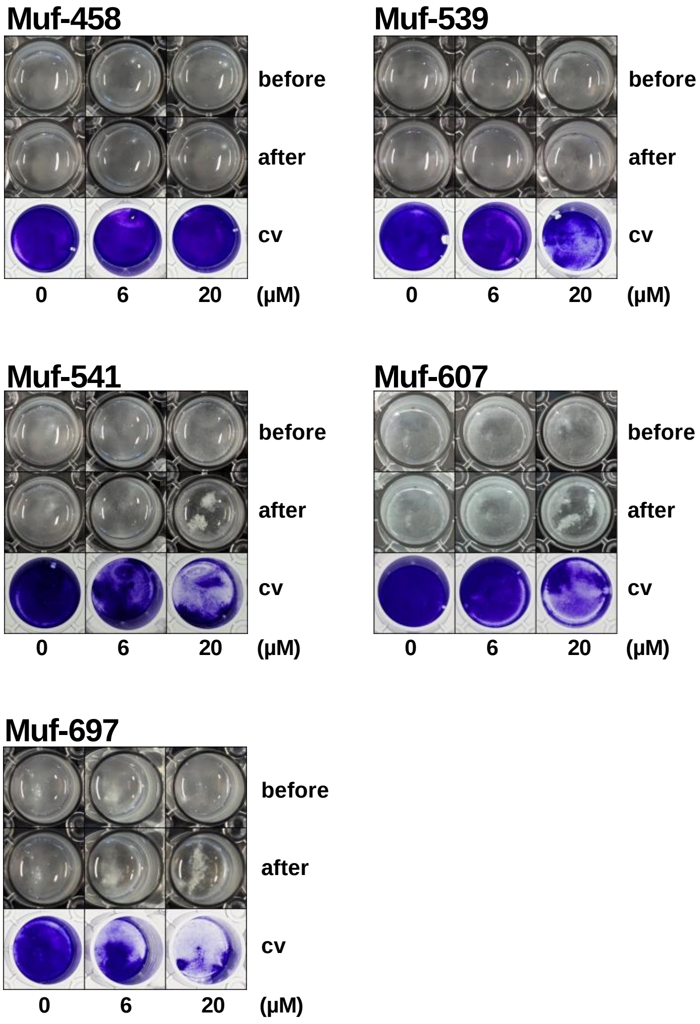

Figure S5. Images of *S. mutans* NMT4863 wild-type biofilms grown for 24 hours upon mutanofactin concentration of 6 µM and 20 µM. Images were taken before (before) and after (after) the measurement in the microplate reader, and upon crystal violet staining (cv).

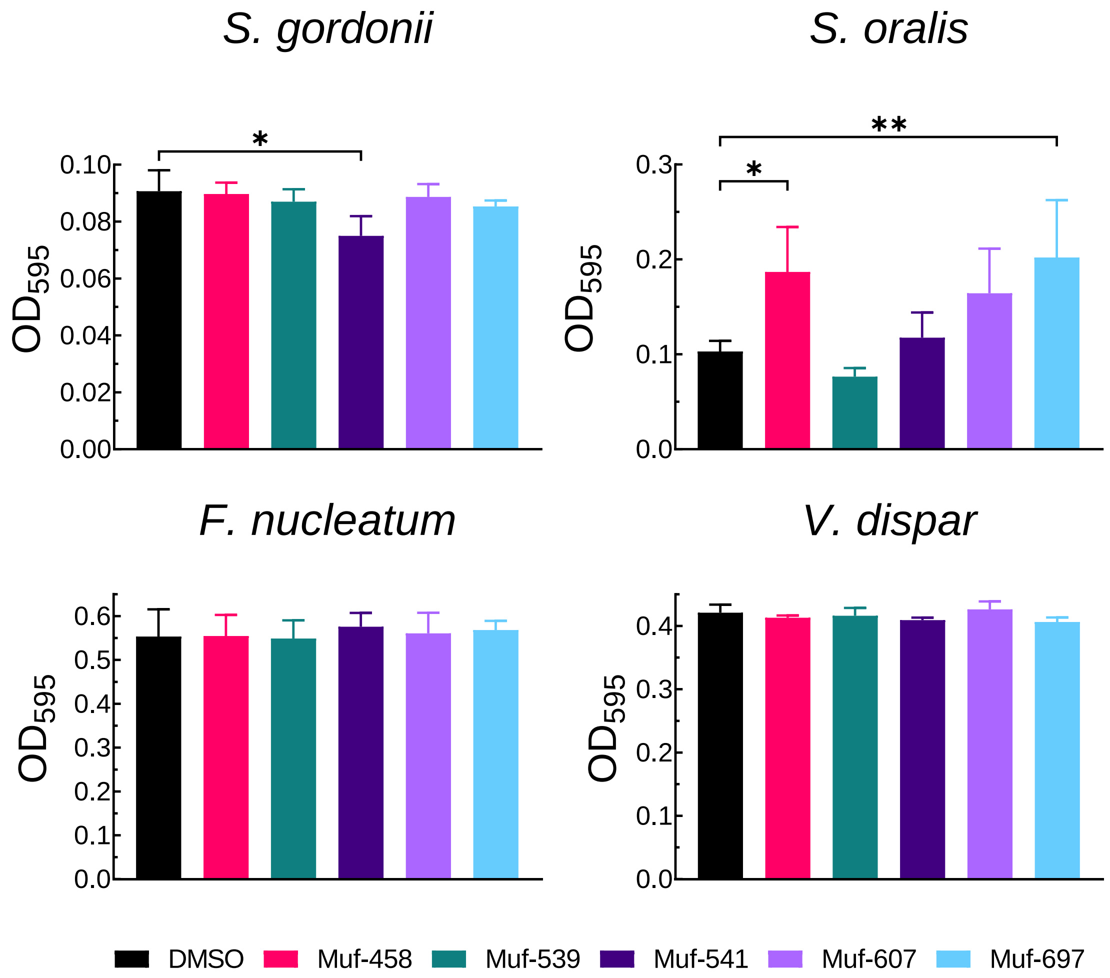

Figure S6. Total biomass of cohabitants of *S. mutans*, including *S. gordonii*, *S. oralis*, *F. nucleatum*, and *V. dispar*, upon provision of mutanofactins at a concentration of 6 µM. Bacteria were grown for 24 hours, and total biomass was determined measuring the optical density at 595 nm. Data are shown as the mean and SD of three to four biological replicates (n = 3-4) tested in three technical replicates. Significance was analysed using a one-way ANOVA for each bacterium with Dunnett's test against the DMSO control (* = $p \leq 0.05$, ** = $p \leq 0.01$).

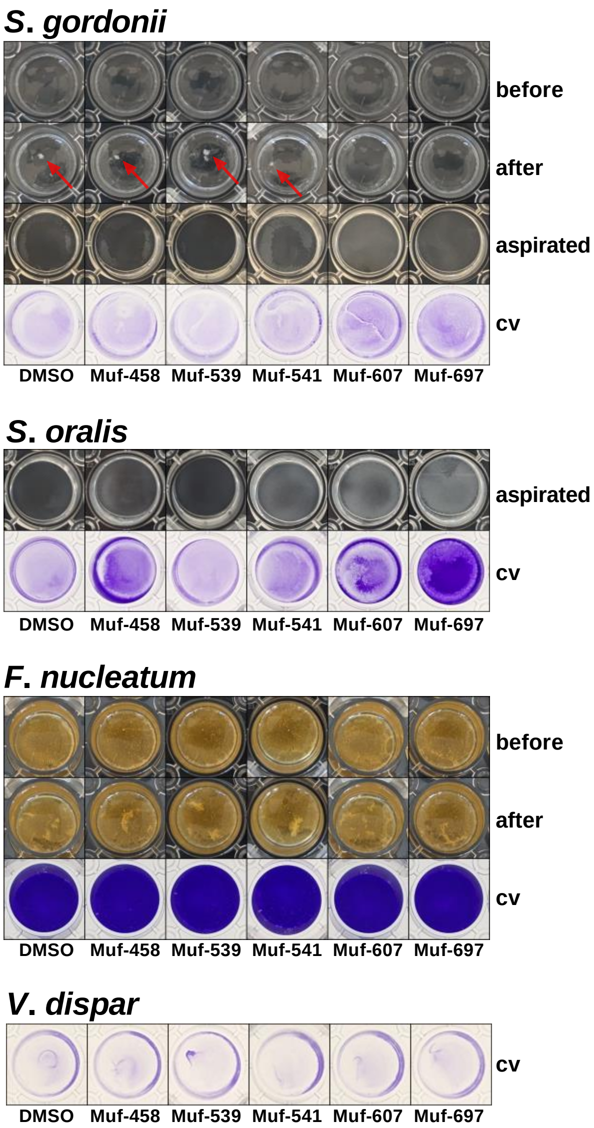

Figure S7. Images of biofilms of *S. gordonii*, *S. oralis, F. nucleatum*, and *V. dispar* grown for 24 hours at a concentration of 6 µM of all five mutanofactins. Images were taken before (before) and after (after) the measurement in the microplate reader, after the aspiration of the liquid medium (aspirated), and after the crystal violet staining (cv). Red arrows highlight biomass aggregates that formed in some cases after the microplate reader measurement. Please note that the yellow color in the wells showing *F. nucleatum* is due to the growth medium used for the bacterium.

Growth Curves

Growth curves of *S. mutans* NMT4863 and the two muf-cluster deletion strains (i.e., Δ*mufD-G* and Δ*mufC-J*) were grown in 24-well plates as described above ("Biofilm growth assays"), (CC7682-7524, Starlab) using 1 mL of BHI medium. The pre-culture conditions and ration of inoculation was as described above for the biofilm assay. The plates were incubated in the Tecan Infinite F200 plate reader at a temperature of 37 °C under ambient air for 24 hours. The absorption at 595 nm was measured at 25 different points (5 by 5 grid) per well, directly after inoculation and subsequently in 1-hour intervals.

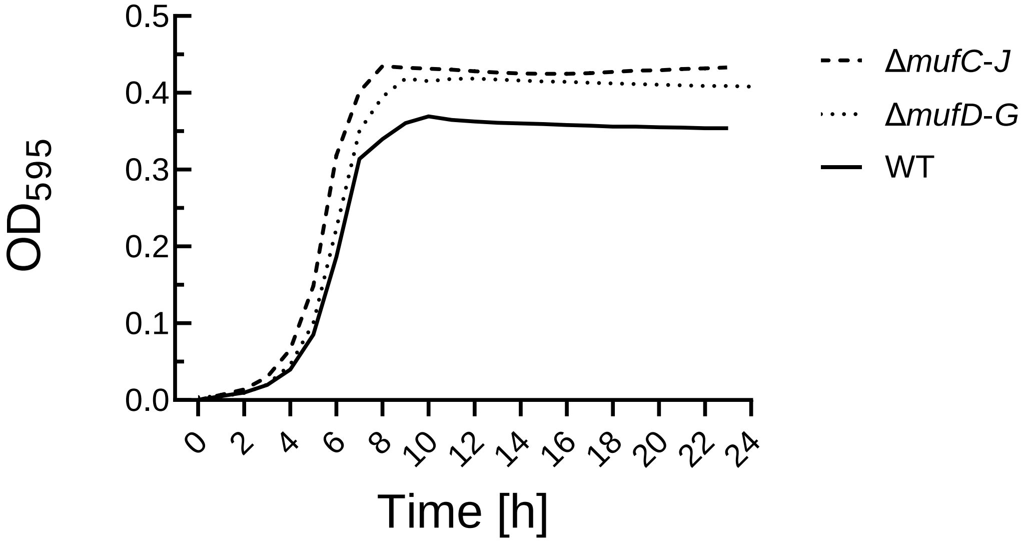

Figure S8. Growth curve of *S. mutans* NMT4863 wild-type (WT) in comparison to *S. mutans* NMT4863 Δ*mufC-J* and Δ*mufD-G*. Bacteria were grown aerobically in microplates at 37 °C in the Tecan Infinite F200 microplate reader and the optical density was measured at 595 nm in 1-hour intervals. Data represent the mean of three biologicals (*n* = 3) tested in three technical triplicates, each.

Biofilm Assays on Mucin-Coating

Polystyrene well-plates were incubated with 800 µL of 1 mg/mL porcine mucin type II (Sigma Aldrich) in PBS, or only PBS for the control, for 24 hours at r.t., shaking with 180 rpm (Adapted from Shi *et al*[45]). After 24 hour, each well was gently washed twice with 1 mL PBS to remove unbound mucin, followed by 1 mL solution of 6 µM mutanofactin-697 (prepared as described above), or the same concentration of DMSO (0.06%) as control. After shaking for one hour at r.t. with 180 rpm each well was aspirated gently using a vacuum pump and washed one last time with 1 mL PBS before adding 1 mL BM (no mutanofactin-697 present).

The biofilm growth on mucin-coated polystyrene was performed with *S. mutans* NMT4863 Δ*MufC-J* according to the protocol above ("Biofilm growth assays"), using only a single PBS washing step prior to heat fixation, as one washing step already removed all biomass on the mucin-coated control.

The assay was also repeated with Muf-607, which showed no change in biofilm formation on a Muf-607 treated mucin layer. This is consistent with our finding (based on QCM-D) that Muf-607 does not interact with mucin.

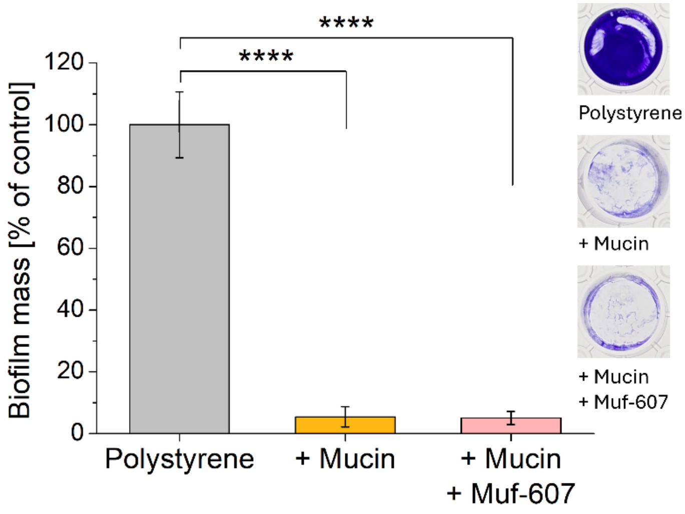

Figure S9. Biofilm mass of *S. mutans* Δ*mufC-J* after 24 h. Each bar represents the normalized mean (non-coated polystyrene set to 100%) and SD of a minimum of six biological replicates (n ≥ 6) tested in three technical replicates. Concentration of Muf-607: 6 µM. The photo inset shows representative crystal violet stained biofilms. Significance was analyzed using a one-way ANOVAs with Tukey's multiple comparisons test (**** = $p \leq 0.0001$).

[45] Shi, L.; Ardehali, R.; Caldwell, K. D.; Valint, P. Mucin coating on polymeric material surfaces to suppress bacterial adhesion. *Colloids Surf. B: Biointerfaces* **2000**, *17* (4), 229-239. DOI: https://doi.org/10.1016/S0927-7765(99)00121-6.

5 Physicochemical Characterization

General Considerations:

Following Chemicals were bought from commercial suppliers, and used as received: brain heart infusion broth (BHI, ≥99% Oxoid), decane (≥99% Sigma-Aldrich), ethanol (≥99.98% Sigma-Aldrich), mucin from porcine stomach type II (Sigma-Aldrich), mucin from bovine submaxillary glands (Sigma-Aldrich) phosphate buffered saline (PBS, Sigma-Aldrich), sodium dodecyl sulfate (SDS, Roth).

Optical Density: measured using a U-2001 spectrophotometer (Methrom Inula GmbH). Data are reported as OD_{hv} where hv denotes the wavelength in nm used for the measurement.

Atomic Force Microscopy: Measurements were performed on a JPK NanoWizard 3 (Bruker). Fitting and evaluation of the recorded force curves was performed with a custom-made Python code, which together with the recorded data is available on the data repository Zenodo (DOI:10.5281/zenodo.14525552).

Cell Surface Hydrophobicity - Microbial Adhesion to Hydrocarbons (MATH)

Microbial adhesion to hydrocarbons (MATH) was performed similarly to as described by Rosenberg *et al.* [46] Bacteria from overnight cultures (37 °C, 5% CO_2) in BHI medium containing 0 µM or 6 µM Muf-697 were recovered by centrifugation (3000 rpm, 4 minutes, 4 °C) and resuspended in 10 mM PBS. The OD_{600} was adjusted to 0.2 in PBS. The bacteria suspension was mixed with a ratio of 1:1.2 with decane and emulsified by vortexing for 2 minutes. The resulting emulsion was left to equilibrate for 15 minutes before the OD_{600} of the aqueous phase was measured again. Microbial adhesion was calculated as a percentage of the OD_{600} values before and after mixing, as shown in Equation 1. Statistical significance of biological triplicates was calculated with a two-Sample t-Test ($p \leq 0.05$). Each biological replicate consists of three technical replicates.

$$\mathrm{MATH}(\%) = 100 * \left(1 - \frac{\mathrm{OD}_{600,\,t_2}}{\mathrm{OD}_{600,\,t_1}}\right) \quad \text{Eq. 1}$$

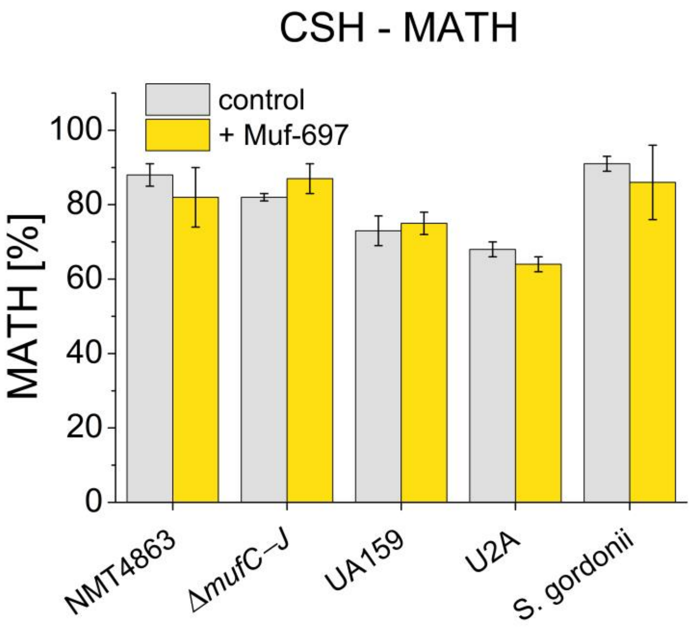

Figure S10. Cell surface hydrophobicity measurements of planktonic cells using (A) microbial adhesion to hydrocarbons (MATH) Yellow: Incubation with 6 µM Muf-697; grey: control samples without Muf-697.

[46] Rosenberg, M.; Rosenberg, E. Bacterial adherence at the hydrocarbon-water interface. *Oil and Petrochemical Pollution* **1985**, *2* (3), 155–162. DOI: https://doi.org/10.1016/S0143-7127(85)90178-4.

Cell Surface Hydrophobicity – Contact Angle

Contact angle measurements were performed following a modified protocol described by Busscher *et al*.[47] Bacteria from overnight cultures (37 °C, 5% CO_2) in BHI medium containing 0 µM or 6 µM Muf-697 were recovered by centrifugation (3000 rpm, 4 minutes, 4 °C) and resuspended in 10 mM PBS. The OD_{600} was adjusted to 0.9 in PBS. A bacteria lawn was obtained by filtering 5 mL bacteria suspension through a 0.22 µm membrane filter (Triton®-free MCE, Merck), creating a round spot of bacteria lawn of approximately 2 cm diameter. The membranes were then dried and fixed to glass slides before the contact angle was measured three times per sample using a drop shape analyser. To that end, a 2.8 µL droplet (MQ-H_2O) suspended from a syringe was applied to the bacteria lawn and an image immediately taken for analysis. Statistical significance of biological triplicates was calculated with a two-Sample t-test ($p \leq 0.05$). Each biological replicate consists of three technical replicates.

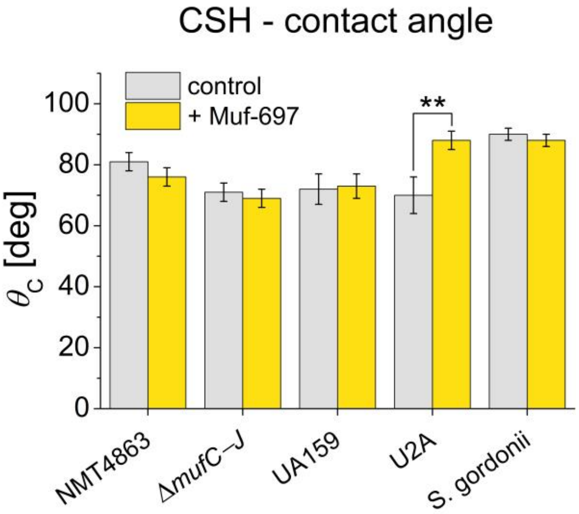

Figure S11. Cell surface hydrophobicity measurements of planktonic cells via water contact angle tests. Yellow: Incubation with 6 µM Muf-697; grey: control samples without Muf-697. The statistical significance of biological triplicates (*n*=3) was calculated with a two-sample t-test (** = $p \leq 0.01$,).

[47] Busscher, H. J.; Weerkamp, A. H.; van der Mei, H. C.; van Pelt, A. W.; de Jong, H. P.; Arends, J. Measurement of the surface free energy of bacterial cell surfaces and its relevance for adhesion. *Appl. Environ. Microbiol.* **1984**, *48* (5), 980–983. DOI: 10.1128/aem.48.5.980-983.1984 (acccessed 2024/07/04).

Biofilm Wetting Assays

Biofilms were cultivated as described above ("Biofilm growth assays") with the difference of using hydroxyapatite discs as substrate (#161120, HiMed Inc). A single sterile disc (9.5 mm diameter) was placed on the bottom of each microplate well. No changes to the growth media, incubation steps, or used volumes were made compared to the biofilm growth assays described above. After 24 hours of biofilm growth the discs were gently removed from the well using a pincer and then air dried for 30 minutes.

Contact angle measurements were performed using a drop shape analyzer (EasyDrop, Krüss). To that end, a 2.8 µL droplet (MQ-H_2O) suspended from a syringe was applied to the hydroxyapatite-supported biofilms and images taken immediately for analysis with Drop Shape Analysis software (version 1.91.0.2, Krüss).

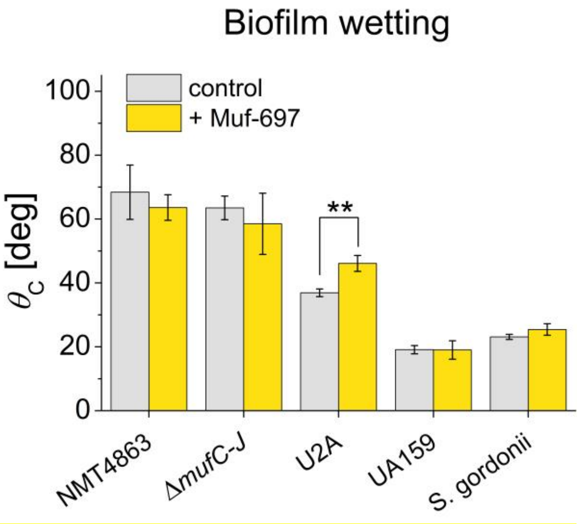

Figure S12. Water contact angle measurements of biofilms formed on solid-liquid interface. Yellow: Incubation with 6 µM Muf-697; grey: control samples without Muf-697. The statistical significance of biological triplicates (n=3) was calculated with a two-sample t-test (** = $p \leq 0.01$).

Bacteria Adhesion Under Flow

Bacteria adhesion was investigated by comparing how much surface area the cells covered on different substrates. To this end, a transparent glass flow cell (Ibidi sticky slide) was fixed to a cleaned glass slide (sonication in 2% SDS, EtOH, and MQ-H_2O for 5 minutes each, followed by UV/ozone treatment for 15 min in a UV/ozone cleaner and connected to a microfluidics setup. The flow cell was placed under a brightfield microscope (Nikon eclipse TE2000-S). First, a mucin layer was allowed to form inside the flow cell by flushing it with a mucin suspension (2.5 mg mL^{-1} in 10 mM PBS) for 30 minutes at a flow rate of 50 µL min^{-1}. After a rinsing step with PBS, a 6 µM Muf-697 solution was pumped through the system to incubate the mucin layer for one hour, followed by another rinsing step. Finally, a suspension of *S. mutans* NMT4863 Δ*mufC–J* was flushed through the system for one hour. The bacteria were obtained from an overnight culture (37 °C, 5% CO_2) in BHI medium. They were recovered by centrifugation (3000 rpm, 4 °C, 4 min) and resuspended in 10 mM PBS. The OD_{600} was adjusted to 0.5 in PBS.

Images for analysis were taken after one hour and the surface coverage of the cells determined with ImageJ (version 1.53t). The results were compared to two controls: Bacteria adhesion to a mucin layer unexposed to Muf-697, and adhesion to a bare glass flow cell. Statistical significance of biological triplicates was calculated with a two-Sample t-test ($p \leq 0.05$).

In addition to the results presented in the main text (Figure 7), we also performed the flow-cell experiment with the *S. mutans* WT under otherwise identical conditions as a control. As Figure S12 shows, almost no adhesion was observed, underscoring that mucin layer exposure to Muf-697 is crucial for *S. mutans* adhesion.

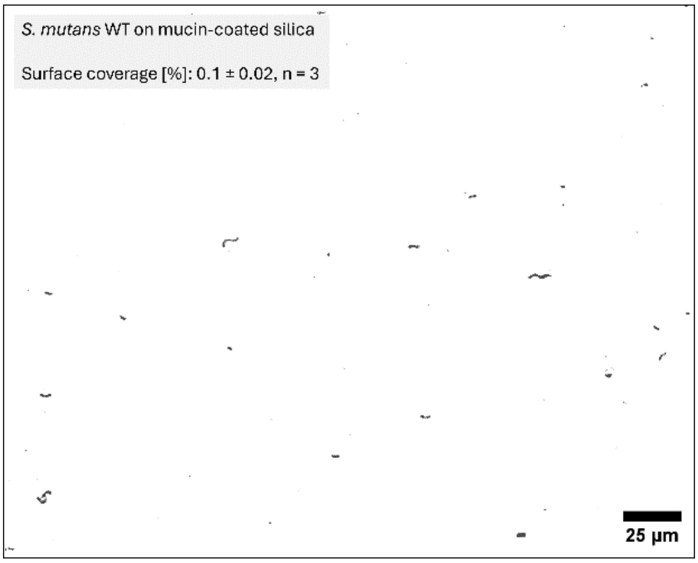

Figure S13. *S. mutans* NMT4863 WT adhesion in flow (50 µL min^{-1}) investigated using a microfluidics setup and brightfield microscope. Exemplary micrograph of bacterial surface coverage after 1 h on mucin-coated silica. The images was background-subtracted and contrast-enhanced using ImageJ software. Surface coverage was calculated from a biological triplicate.

Water Contact Angles

Quartz crystal microbalance sensors (coated with either hydroxyapatite or silicon dioxide) were cleaned by sonicating them for 5 minutes in 2% SDS, EtOH, and MQ-H_2O, respectively, followed by UV/ozone treatment for 15 min in a UV/ozone cleaner. The contact angle was measured using a drop shape analyser. To that end, a 75 µL MQ-H_2O droplet suspended from a syringe was applied to the cleaned sensor surface and an image immediately taken for analysis (Drop Shape Analysis version 1.91.0.2, Krüss). The sensors were then dried with nitrogen and the contact angle of two more droplets measured. Next, the sensors were submerged in a mucin suspension (2.5 mg mL^{-1}) and placed on a rotary shaker at 70 rpm for one hour. The sensors were then carefully rinsed with 10 mM PBS, dried with nitrogen and the contact angle of three individual droplets measured. Finally, the mucin layer was incubated with a 6 µM solution of Muf-697 protected from light on a rotary shaker at 70 rpm for one hour, rinsed with PBS, and the contact angle of three individual droplets measured. Statistical significance of biological triplicates was calculated with a Two-Sample t-Test ($p \leq 0.05$). Each biological replicate consists of three technical replicates.

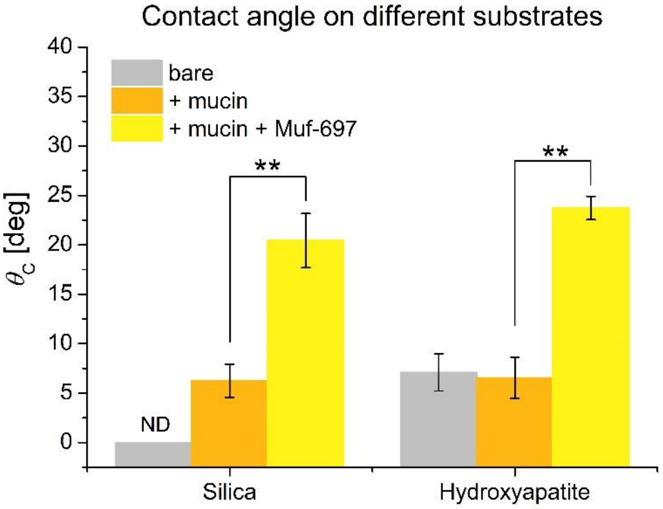

Figure S14. Water contact angle measurements on QCM-D crystals: bare substrate (grey), mucin coated substrate (orange), mucin coated substrate incubated with 6 µM Muf-697 (yellow). Statistical significance of technical triplicates was calculated with a Pair-Sample t-Test (** = $p \leq 0.01$). ND denotes a not detectable contact angle due to full wetting of the surface.

Quartz Crystal Microbalance with Dissipation Monitoring (QCM-D)

The effect of different mutanofactin species on mucin layer properties was measured with Quartz crystal microbalance with dissipation monitoring (QCM-D). A QCM-D (QSense® E4, Biolin Scientific) connected to a microfluidics setup was used to monitor the formation of a mucin layer on QCM-D crystals (coated with either hydroxyapatite or silicon dioxide, Biolin Scientific) and how mutanofactin affects the layer in real time. The sensors were cleaned by sonicating them for 5 minutes in 2% SDS, EtOH, and MQ-H_2O, respectively, followed by UV/ozon treatment for 15 minutes in a UV/ozon cleaner (ProCleaner™, BioForce Nanosciences). First, a mucin suspension (2.5 mg mL^{-1} in 10 mM PBS) was pumped through three flow cells simultaneously at 50 µL min^{-1} until shifts in frequency and dissipation overtones were plateauing. Then, a rinsing step with 10 mM PBS was performed before switching to the respective mutanofactin solution (6 µM in 10 mM PBS). The mutanofactin solution was pumped through the flow cells until frequency and dissipation overtones reached a plateau or stayed unchanged for 30 minutes, followed by final rinsing step with PBS. The quantification of layer thicknesses was performed using QTools (version 3.1.32.562, Biolin Scientific) by fitting the data using the extended viscoelastic model. Statistical significance of technical triplicates was calculated with a Paired-Sample t-Test ($p \leq 0.05$).

QCM-D measurement with Muf-697 on Silica:

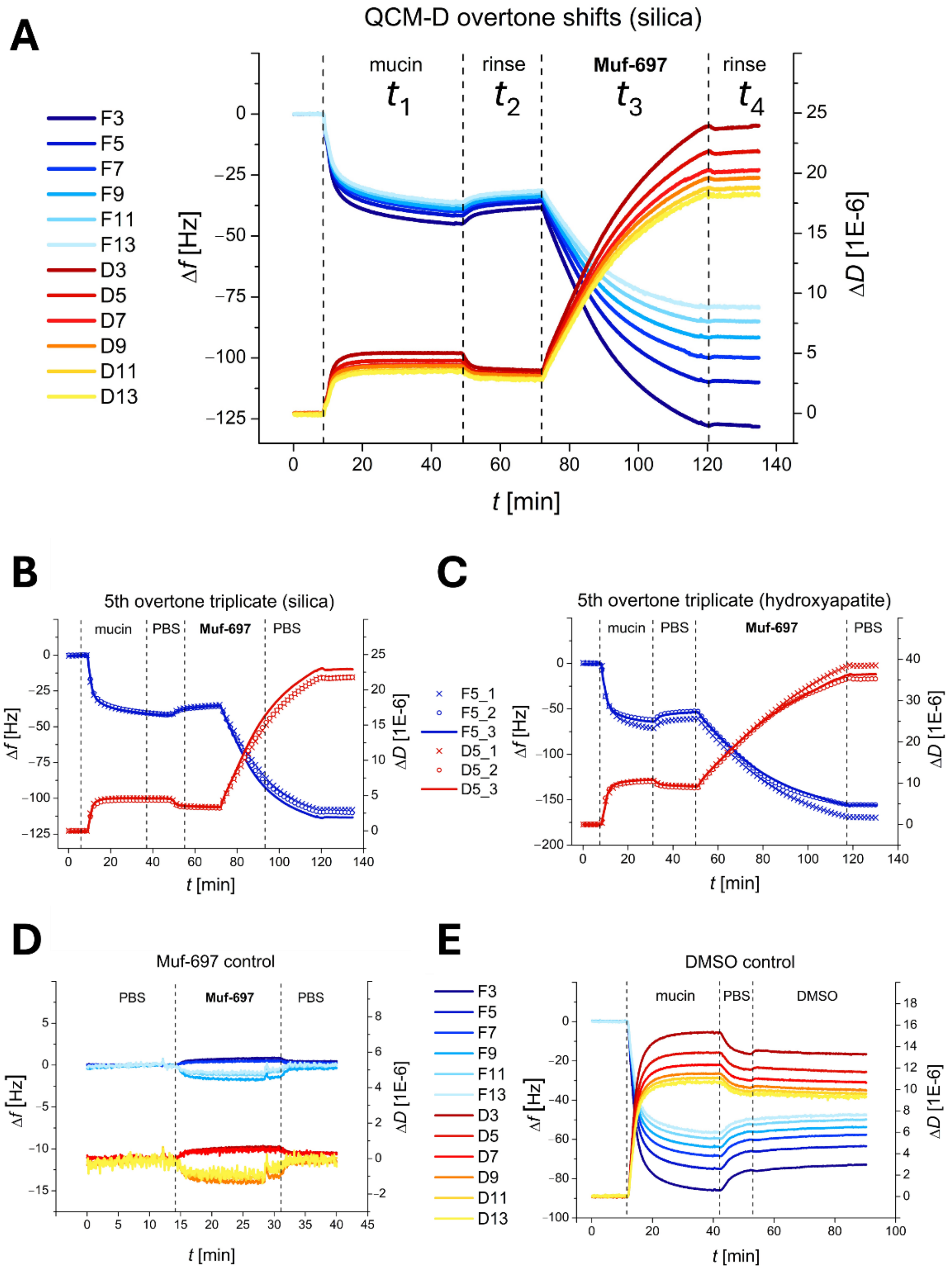

Figure S15. (A) Frequency and dissipation overtones 3 - 13 on silica. Cold colors: frequency shifts; warm colors: dissipation shifts. (B) Technical triplicate of QCM-D experiments on silica, (C) on hydroxyapatite. Blue: frequency shifts; red: dissipation shifts. The fifth overtone is shown as a representative example (D) Muf-697 on bare hydroxyapatite. (E) Mucin layer exposed to DMSO. Vertical lines mark when a component is introduced into the system.

Atomic Force Microscopy

Atomic force microscopy was performed using a JPK NanoWizard 3 (Bruker). The mucin layer was prepared on a glass cover slip that was cleaned by sonication in solutions of 2% SDS, EtOH, and MQ-H_2O for 5 min each, followed by 15 min exposure to UV/ozone. Then, the cover slip was submerged in a mucin suspension (2.5 mg mL^{-1} in 10 mM PBS) and placed on a rotary shaker for one hour at 70 rpm. The formed mucin layer was then carefully rinsed with PBS and kept in PBS for the measurement. During the measurement, a force map of 16 x 16 pixels was recorded in a 2000 x 2000 µm square in contact mode using a MLCT cantilever (C triangular, Bruker) with a nominal spring constant of 0.015 N m^{-1}. Cantilever calibration was performed with the contact-based calibration method and the JPK Nanowizard control software (Bruker). The force curves were recorded with a setpoint force of 1.0 nN. Each point was approached from a z-length of 0.5 µm and with an approach speed of 0.5 µm s^{-1}. After recording a force map on the pure mucin layer, it was incubated with a Muf-697 solution (6 µM in 10 mM PBS) protected from light for one hour, and the measurement repeated. Evaluation of the recorded force curves was performed with a custom-made Python code, as described below. The code is available as supplementary file to this article.

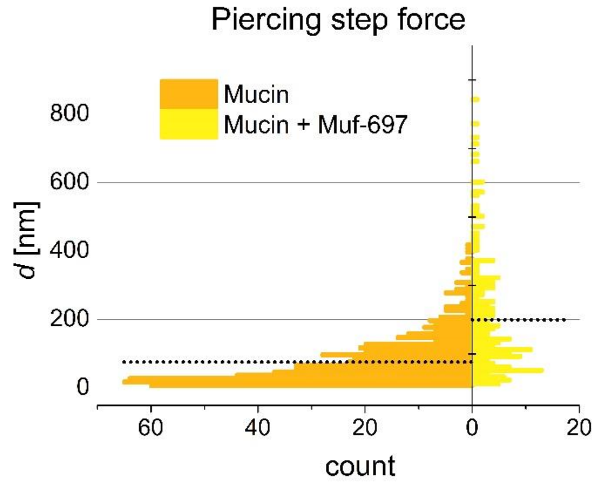

Figure S16. Mucin layer piercing step force measured by AFM. Yellow: mucin layer; orange: Muf-697 incubated mucin layer. Dotted lines mark mean values.

Analysis of AFM force spectroscopy data:

Data analysis was performed in Jupyter notebooks[48] using the Python programming language. For numerical calculations, the numpy[49] scipy,[50] and polars[51] packages were utilized. Data visualization was backed by matplotlib[52]. Interactive widgets for browsing data and obtaining feedback on algorithmic

[48] Granger, B. E.; Pérez, F. Jupyter: Thinking and Storytelling With Code and Data. *Comput. Sci. Eng.* **2021**, *23* (2), 7–14. DOI: 10.1109/MCSE.2021.3059263.
[49] Harris, C. R.; Millman, K. J.; van der Walt, S. J.; Gommers, R.; Virtanen, P.; Cournapeau, D.; Wieser, E.; Taylor, J.; Berg, S.; Smith, N. J.; et al. Array programming with NumPy. *Nature* **2020**, *585* (7825), 357–362. DOI: 10.1038/s41586-020-2649-2.
[50] Virtanen, P.; Gommers, R.; Oliphant, T. E.; Haberland, M.; Reddy, T.; Cournapeau, D.; Burovski, E.; Peterson, P.; Weckesser, W.; Bright, J.; et al. SciPy 1.0: fundamental algorithms for scientific computing in Python. *Nat. Methods* **2020**, *17* (3), 261–272. DOI: 10.1038/s41592-019-0686-2.
[51] Ritchie Vink, S. d. G., Alexander Beedie, Marco Edward Gorelli, Weijie Guo, J van Zundert, Gert Hulselmans, universalmind303, Orson Peters, Marshall, chielP, nameexhaustion, Matteo Santamaria, Daniël Heres, Josh Magarick, ibENPC, Moritz Wilksch, Jorge Leitao, Jonas Haag, Marc van Heerden, cmdlineluser, Oliver Borchert, Chris Pryer, Ion Koutsouris, Ryan Russell, Liam Brannigan, Joshua Peek, Colin Jermain, Adrián Gallego Castellanos. pola-rs/polars: Python Polars 0.20.7. *Zenodo* **2024**. DOI: 10.5281/zenodo.10616464.
[52] Hunter, J. D. Matplotlib: A 2D Graphics Environment. *Comput. Sci. Eng.* **2007**, *9* (3), 90–95. DOI: 10.1109/MCSE.2007.55.

parameters were crafted using ipywidgets[53]. Force spectroscopy data were loaded from files produced by JPK's AFM control software via the nanite[54] package.

Cantilever sensitivity, i.e., the proportionality constant relating measured vertical deflection voltage and cantilever bending, was determined as follows: From each deflection–vs.–height curve, the section starting 100 nm away from the turning point (i.e., maximum extension) and ending 10 nm away from the turning point was extracted from the approach segment. A polynomial of degree 1 was fit to each section using the numpy.polyfit function. Each resulting first order polynomial coefficient (i.e., slope) s_i represented an estimate of the negative reciprocal sensitivity. Therefore the sensitivity was calculated according to mean$[-\frac{1}{s_i}]$. In conjunction with the cantilever spring constant determined by JPK's software, this allowed for conversion of deflection voltage to force.

For each recorded force spectroscopy run, the tip position z was computed from the measured height h, force F, and spring constant k via the relation $z = h + F/k$. Contact point detection was performed employing the frechet_direct_path method from *nanite*. The measured height h was normalized by subtracting the height at the contact point. Force F was normalized by subtracting the mean of approach segment force values $F(h)$ for which 10 nm $\leq h \leq 50$ nm (i.e., values close to the contact point with some margin). Tip position z was normalized by subtracting the mean of approach segment tip position values $z(F)$ for which 0.5 nN $\leq F \leq 1.0$ nN (i.e, high force values for which the cantilever mostly sensed the glass substrate).

Subsequently, normalized force–vs.–tip position curves were assessed by eye. Curves exhibiting excessive noise or oscillations, e.g., due to building vibrations, were excluded from further analysis.

A custom implementation of a step finding algorithm[55] was utilized to detect incidents of the AFM tip piercing through the mucin layer on the glass substrate. The algorithm was applied to the approach segment of tip position–vs.–height curves starting at 7 nm before the contact point (to allow for some inaccuracy in contact point detection) up to the turning point. Parameters (smoothing width $\sigma = 2.0$ and window size $w = 20$) were chosen to ensure high sensitivity and specificity. Peaks in the resulting θ curve above a threshold of 0.08 were taken as indicators of break-through events.

In order to determine the mucin layer's brush height, a modified Alexander-de Gennes model[56] was fit to a section of each force–vs.–tip position approach curve via a non-linear least squares method (scipy.optimize.curve_fit). The segments were defined by 5 nm $\leq z \leq 20$ nm and $F \leq 0.2$ pN to (approximately) fulfill the model's condition $0.2 < d/h < 0.9$. Sections were further shortened if necessary to exclude break-through events. Only segments comprised of at least 50 datapoints and featuring a maximum force above 50 pN were fitted to ensure reliable results.

---

[53] https://github.com/jupyter-widgets/ipywidgets, accessed 22.07.2024

[54] https://github.com/AFM-analysis/nanite, accessed 22.07.2024

[55] Opfer, J.; Gottschalk, K.-E. Identifying Discrete States of a Biological System Using a Novel Step Detection Algorithm. *PLOS ONE* **2012**, *7* (11), e45896. DOI: 10.1371/journal.pone.0045896.

[56] Mumtaz Virk, M.; Hofmann, B.; Reimhult, E. Formation and Characteristics of Lipid-Blended Block Copolymer Bilayers on a Solid Support Investigated by Quartz Crystal Microbalance and Atomic Force Microscopy. *Langmuir* **2019**, *35* (3), 739–749. DOI: 10.1021/acs.langmuir.8b03597.

6 NMR Spectra

^{1}H NMR (500 MHz, $CDCl_3$) of Primary amide SI_1:

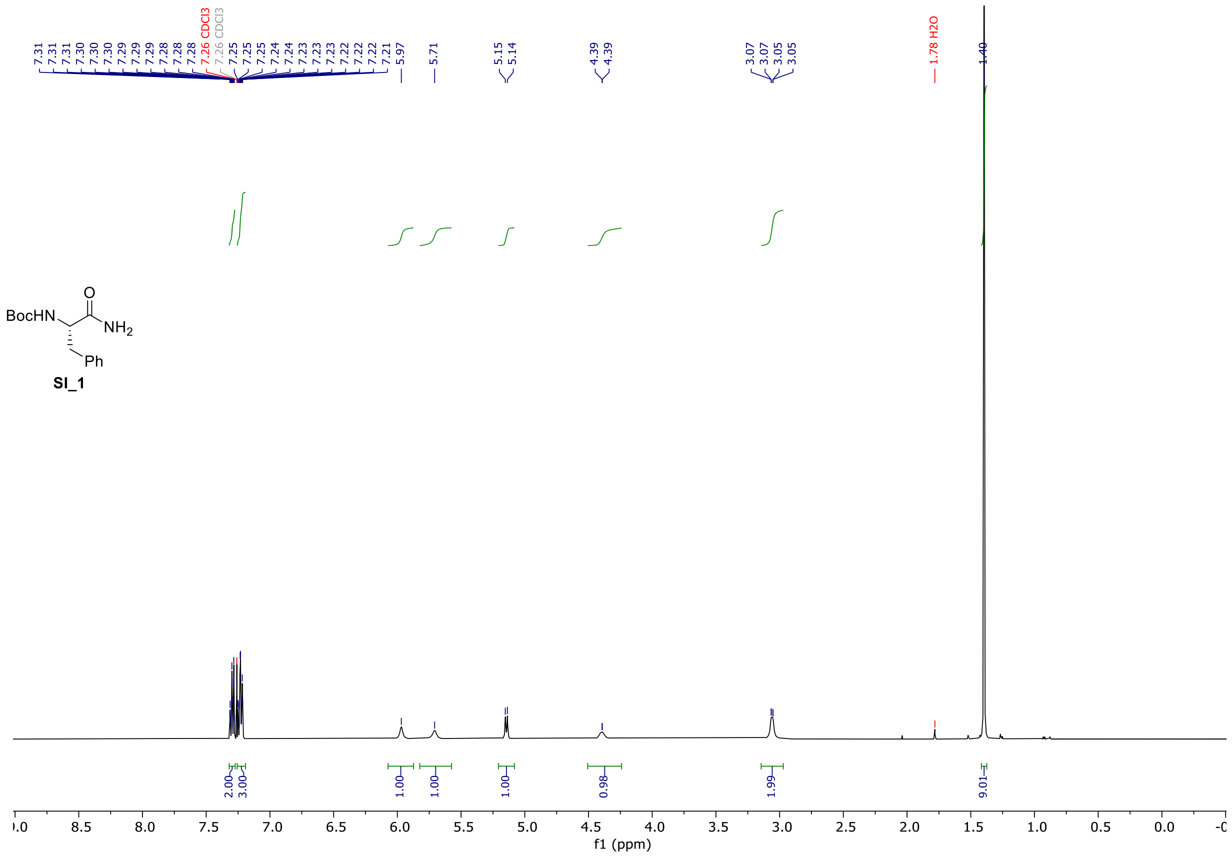

^{13}C NMR (151 MHz, $CDCl_3$) of Primary amide SI_1:

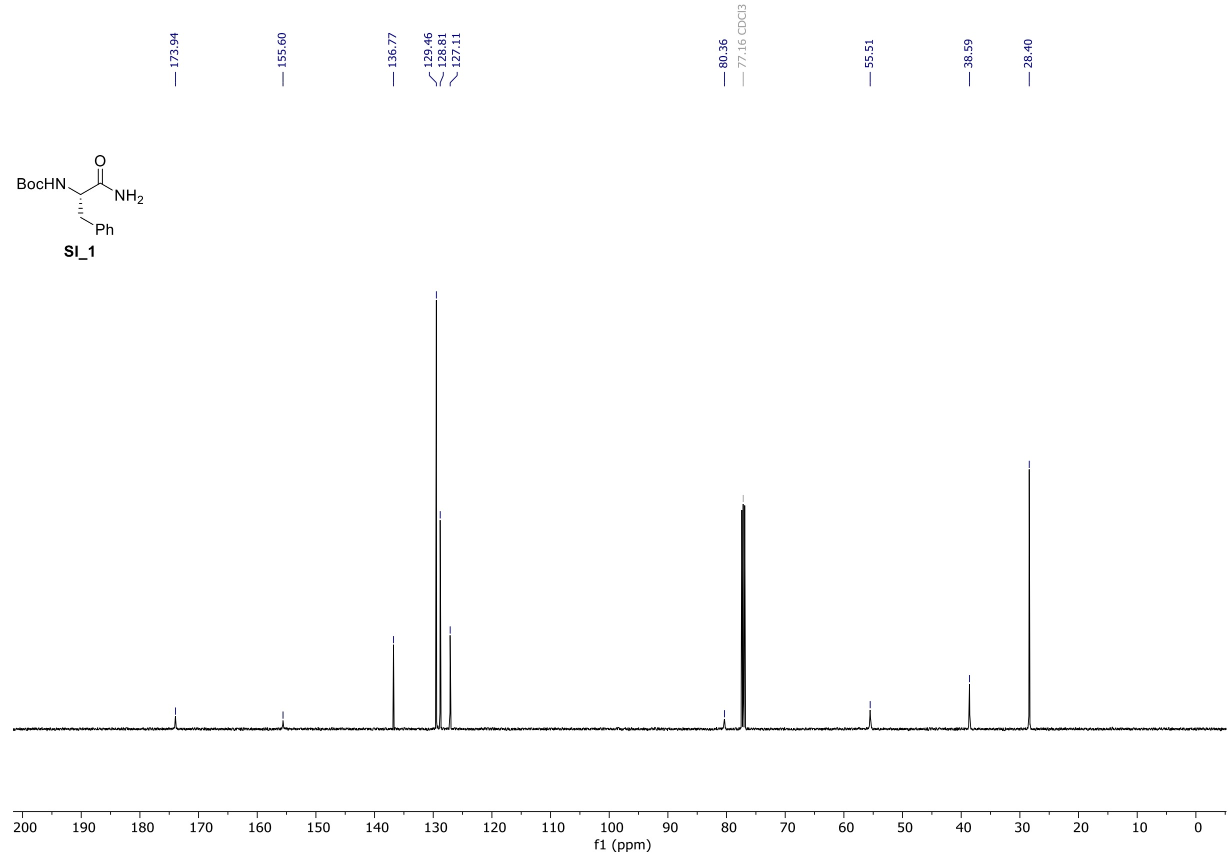

^1H NMR (400 MHz, $CDCl_3$) of Thioamide 1:

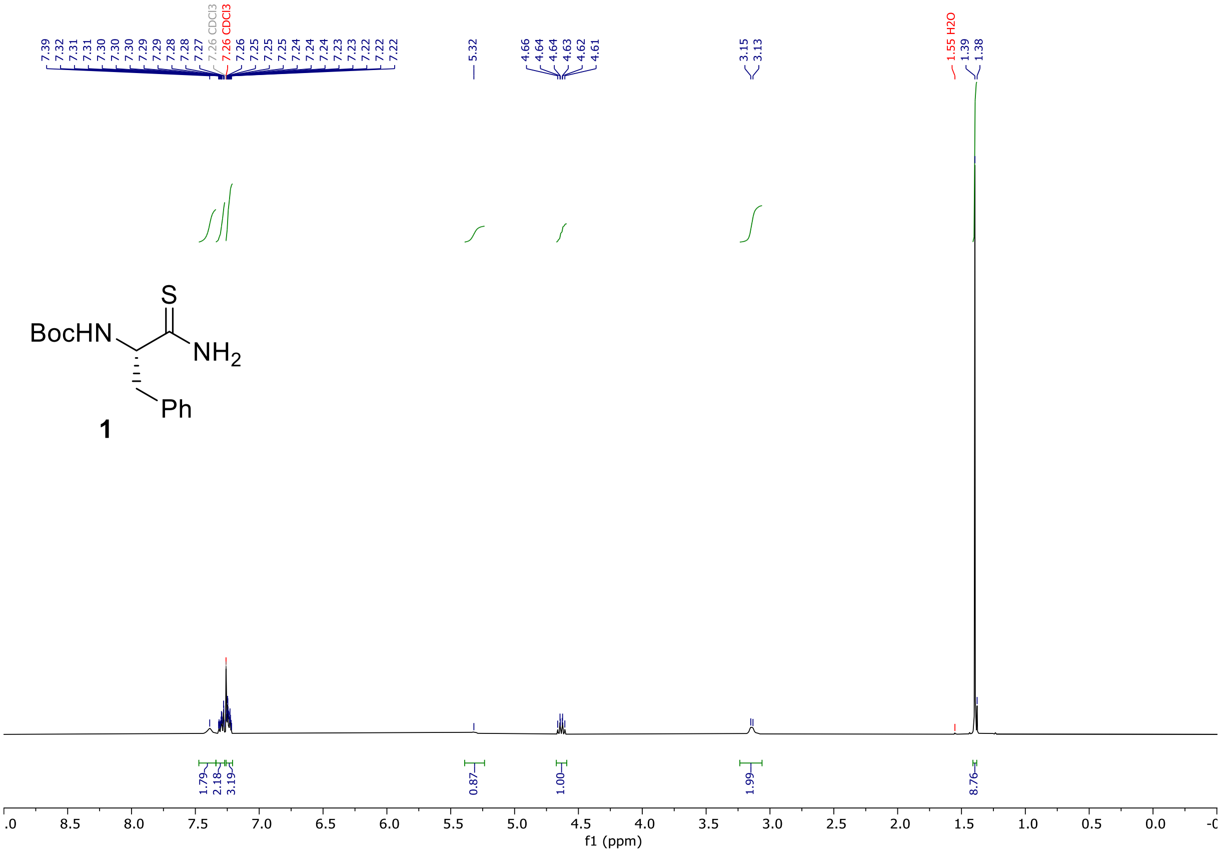

^1H NMR (151 MHz, $CDCl_3$) of Thioamide 1:

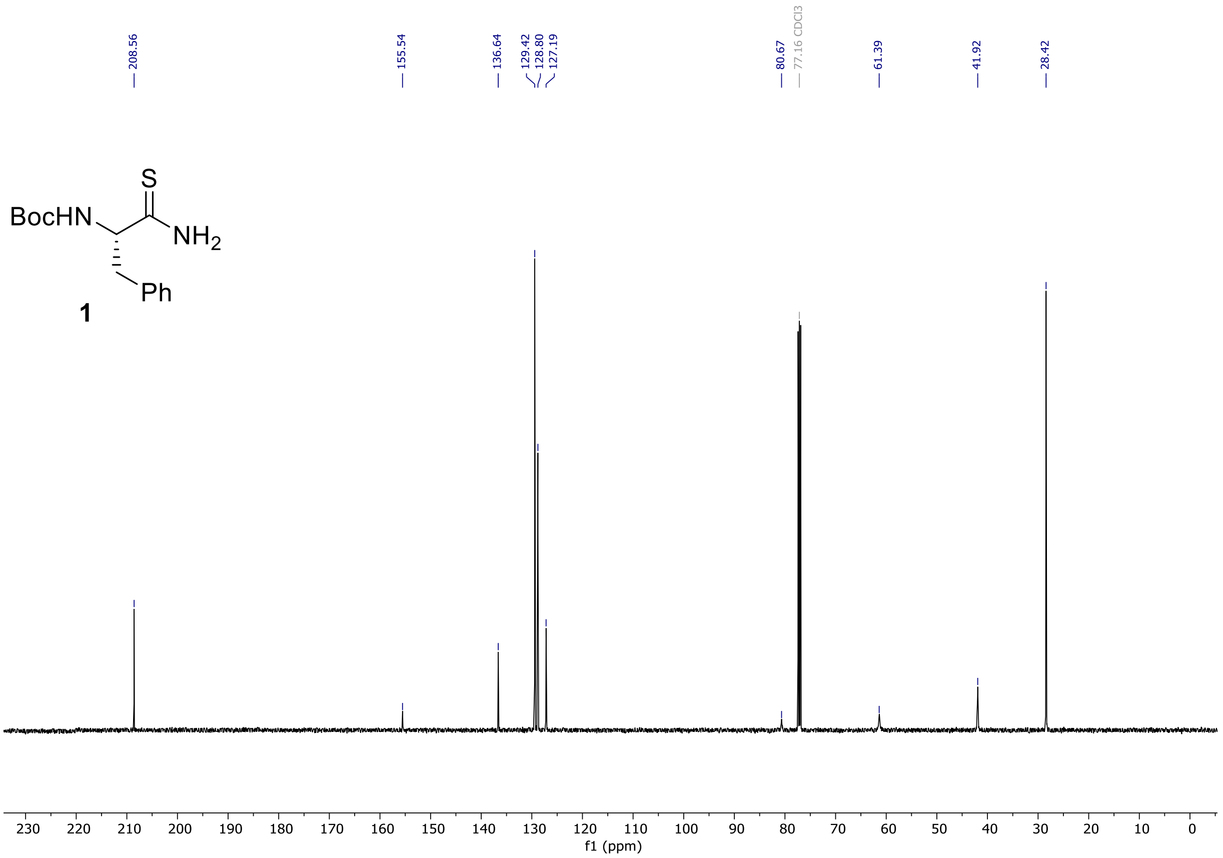

^{1}H NMR (500 MHz, $CDCl_3$) of Thiazole 3:

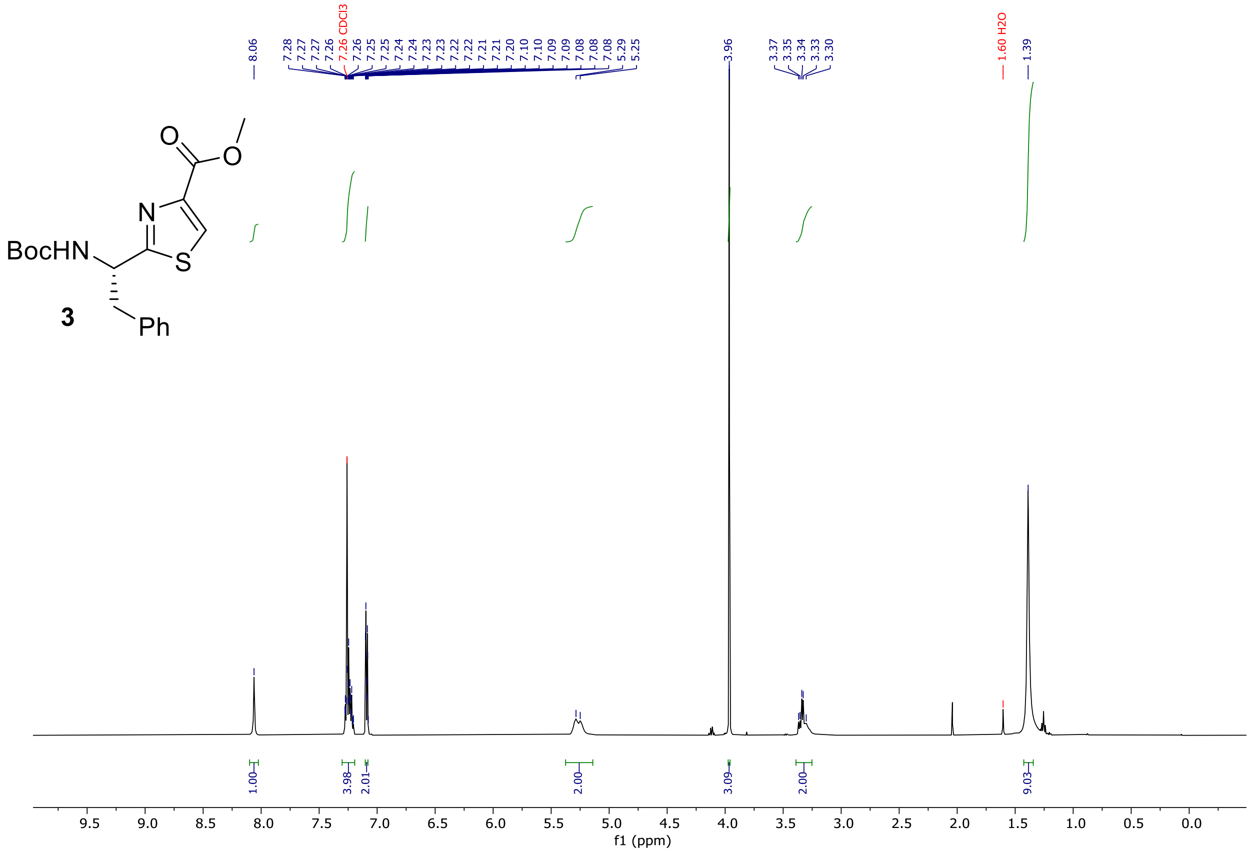

^{1}H NMR (151 MHz, $CDCl_3$) of Thiazole 3:

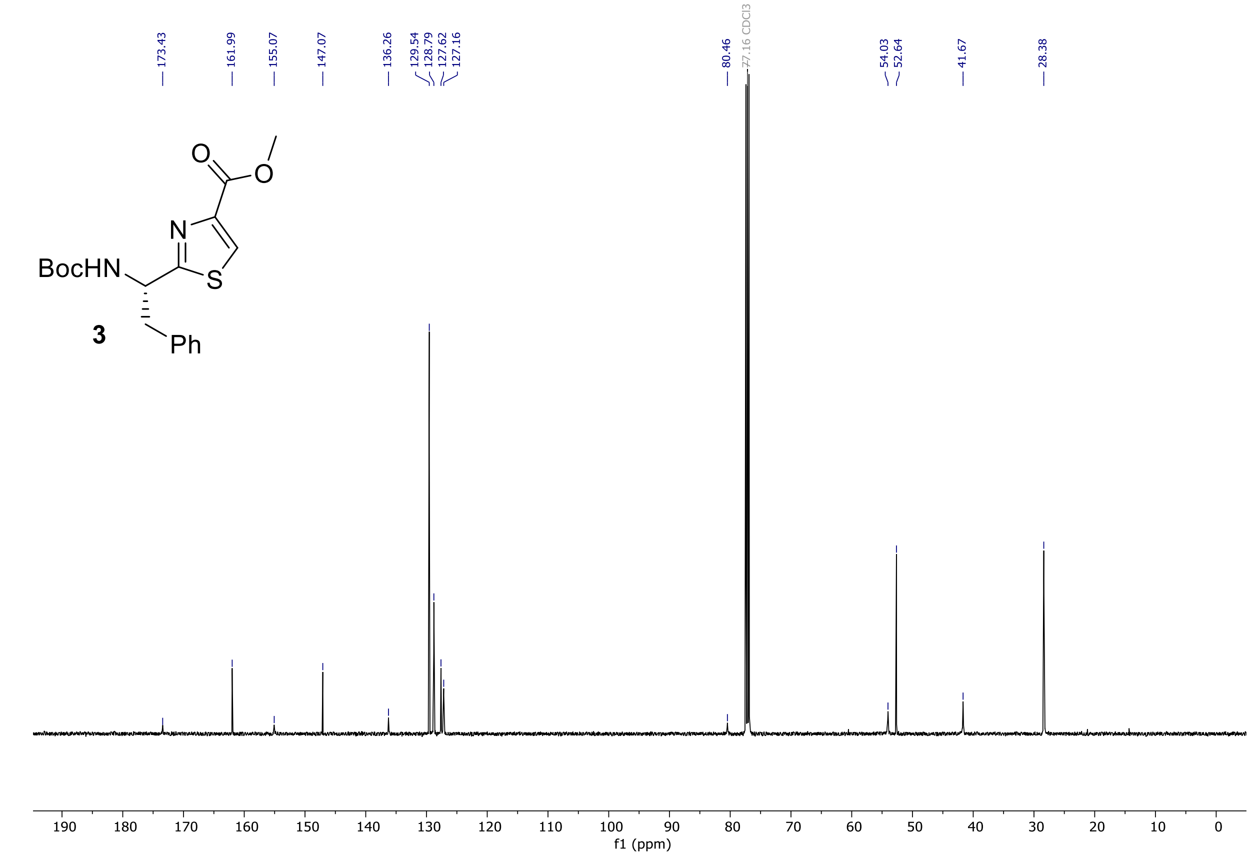

^{1}H NMR (500 MHz, $CDCl_3$) of Dipeptide 4:

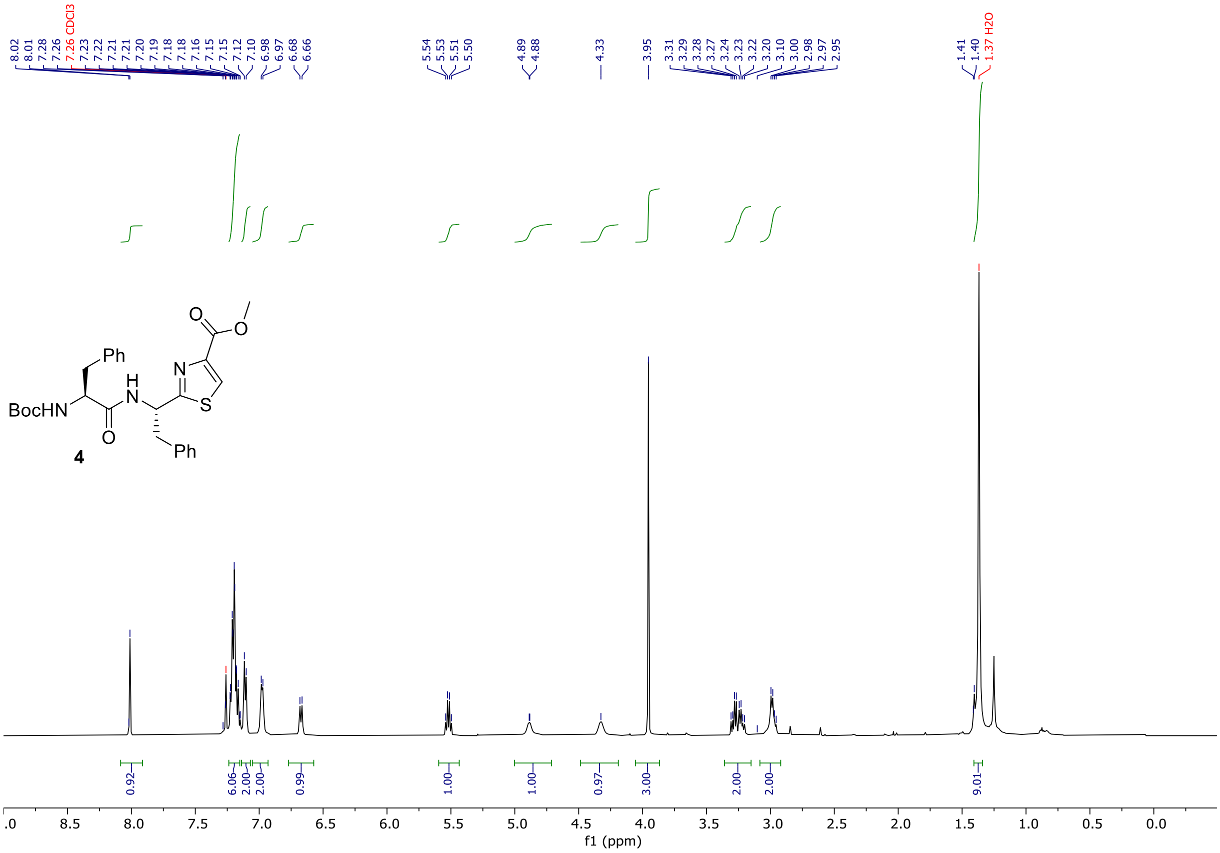

^{1}H NMR (151 MHz, $CDCl_3$) of Dipeptide 4:

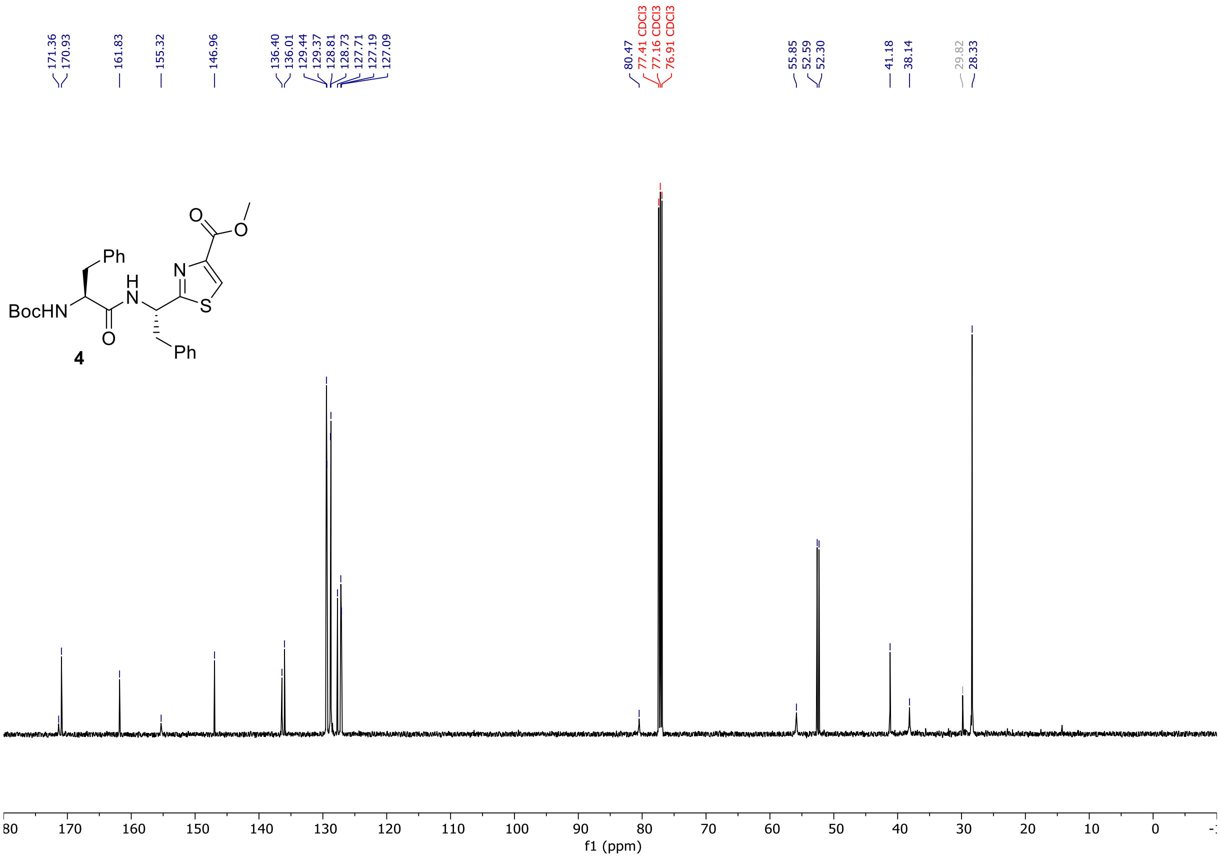

^{1}H NMR (400 MHz, $CDCl_3$) of Aldehyde 5:

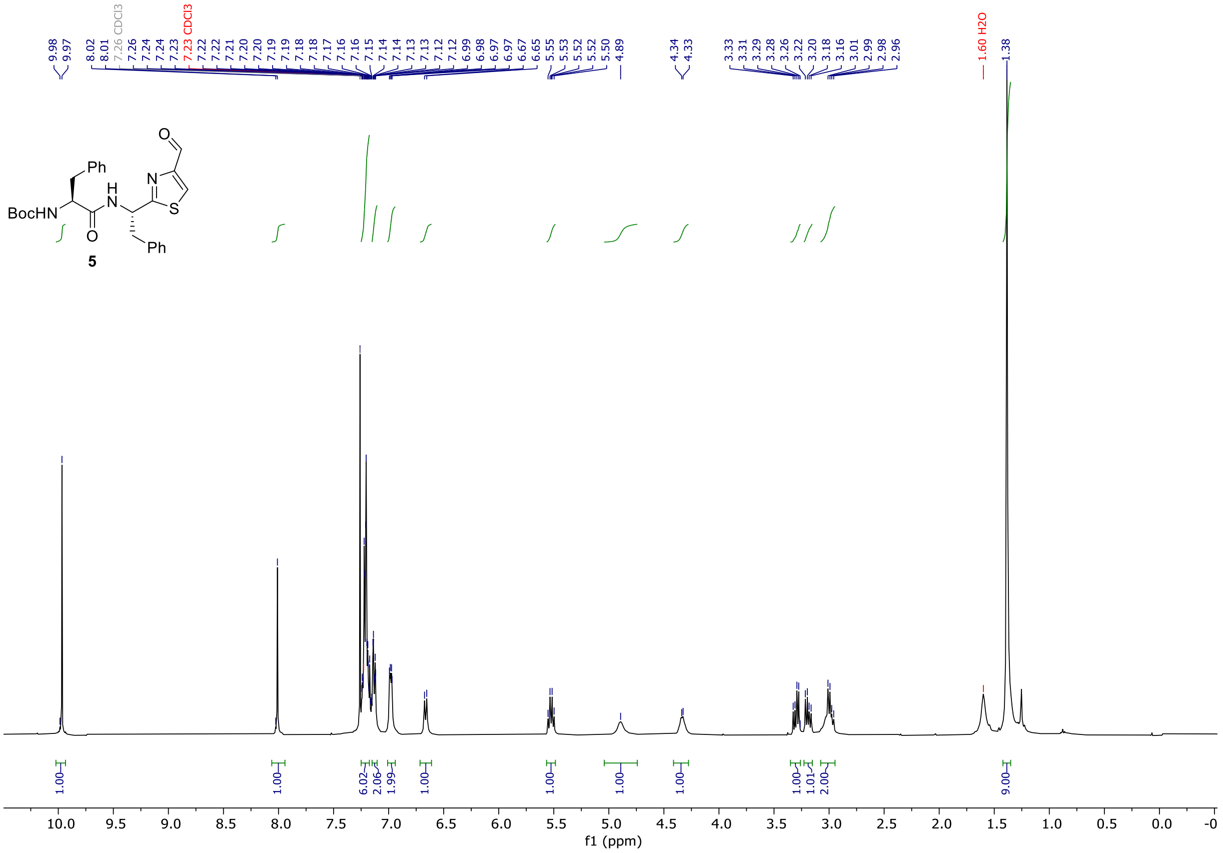

^{1}H NMR (101 MHz, $CDCl_3$) of Aldehyde 5:

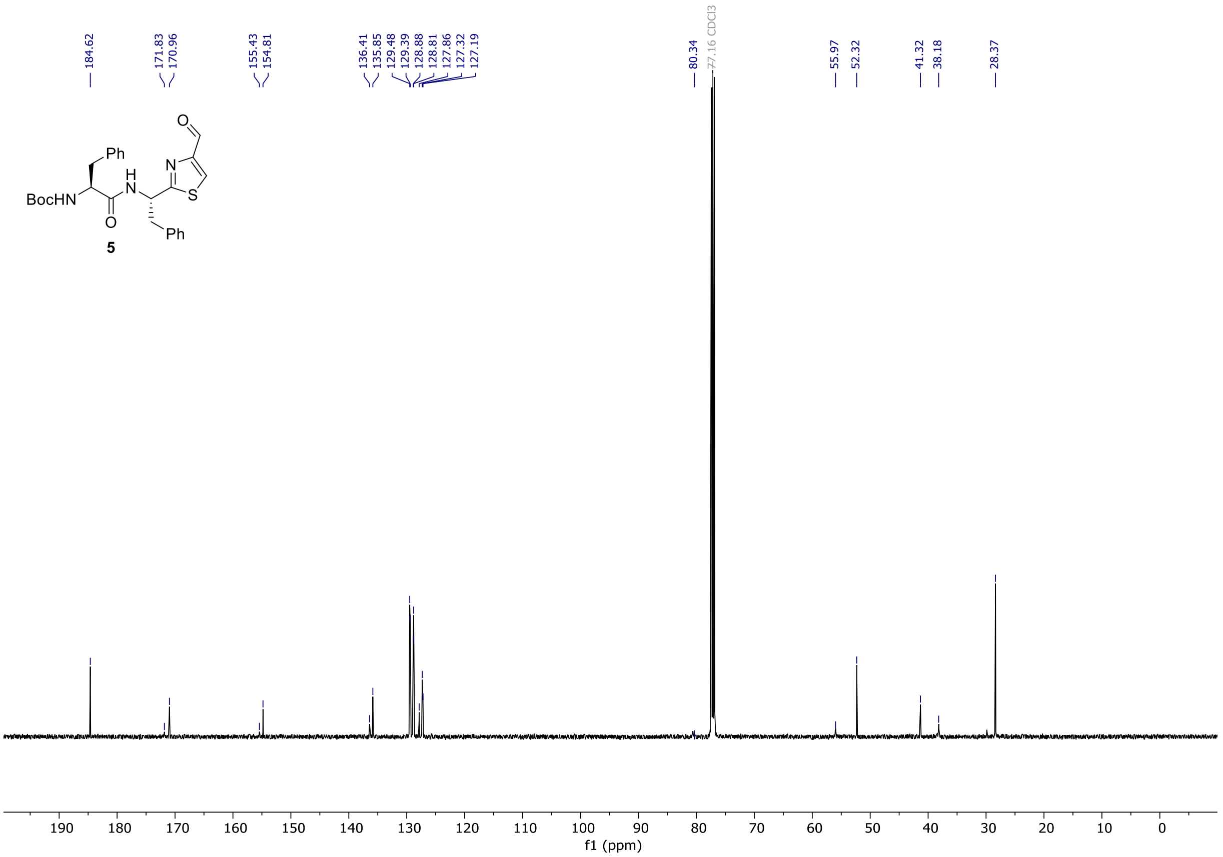

^{1}H NMR (500 MHz, $CDCl_3$) of Alcohol SI_2:

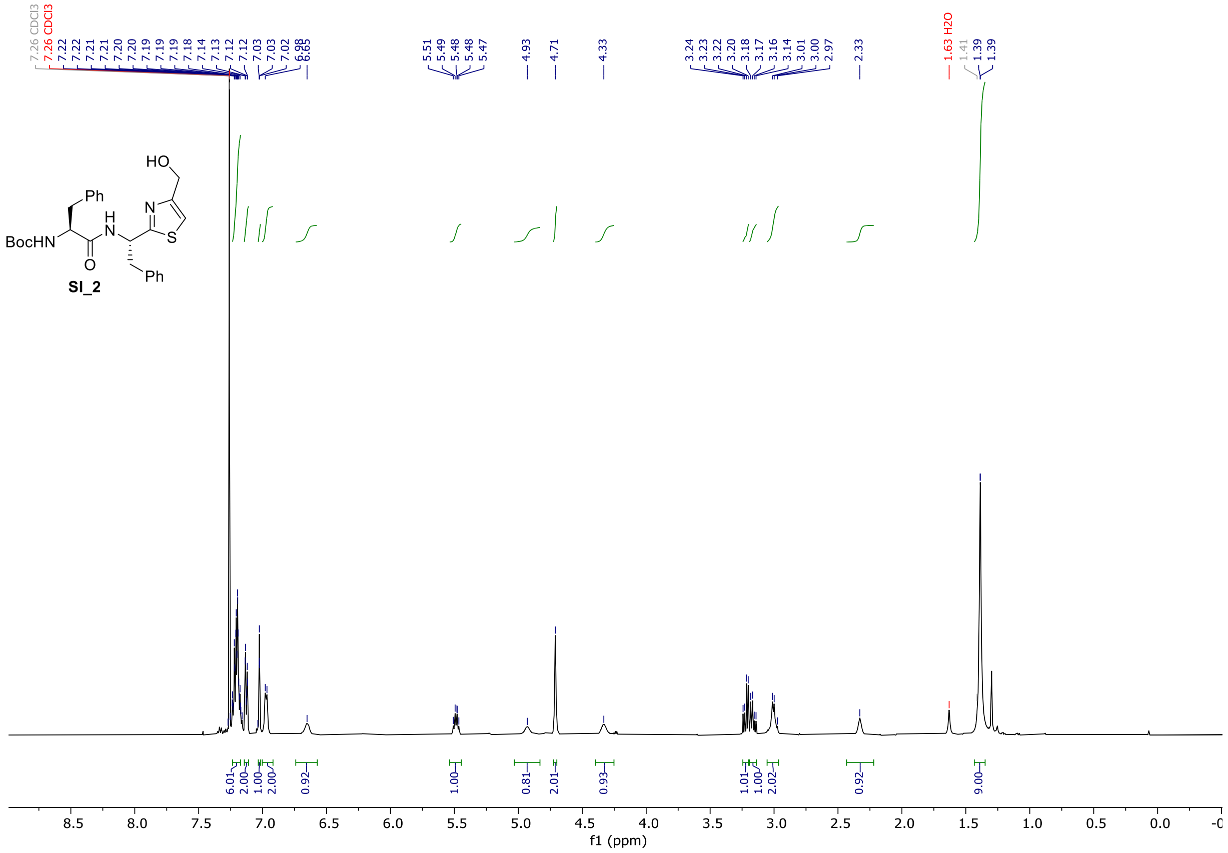

^{13}C NMR (151 MHz, $CDCl_3$) of Alcohol SI_2:

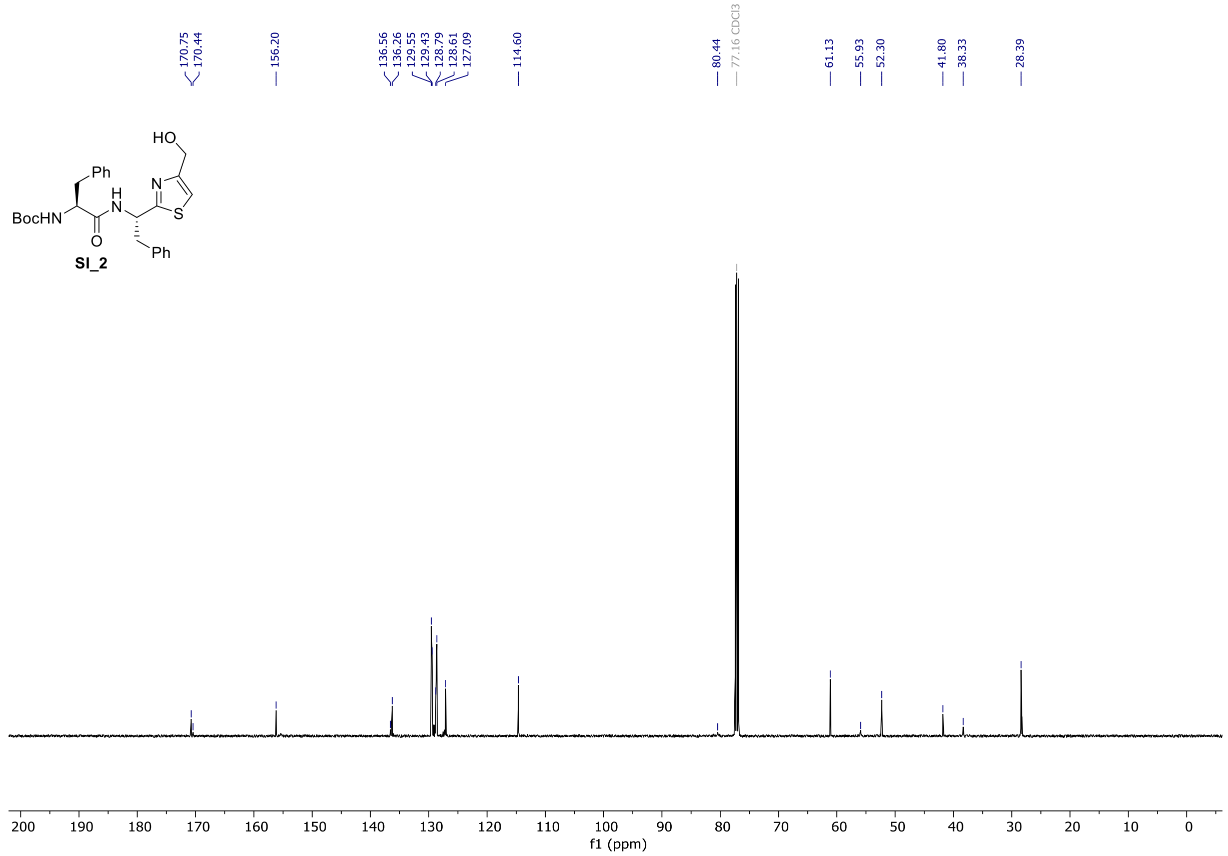

^{1}H NMR (500 MHz, $CDCl_3$) of Mukayiama adduct SI_3:

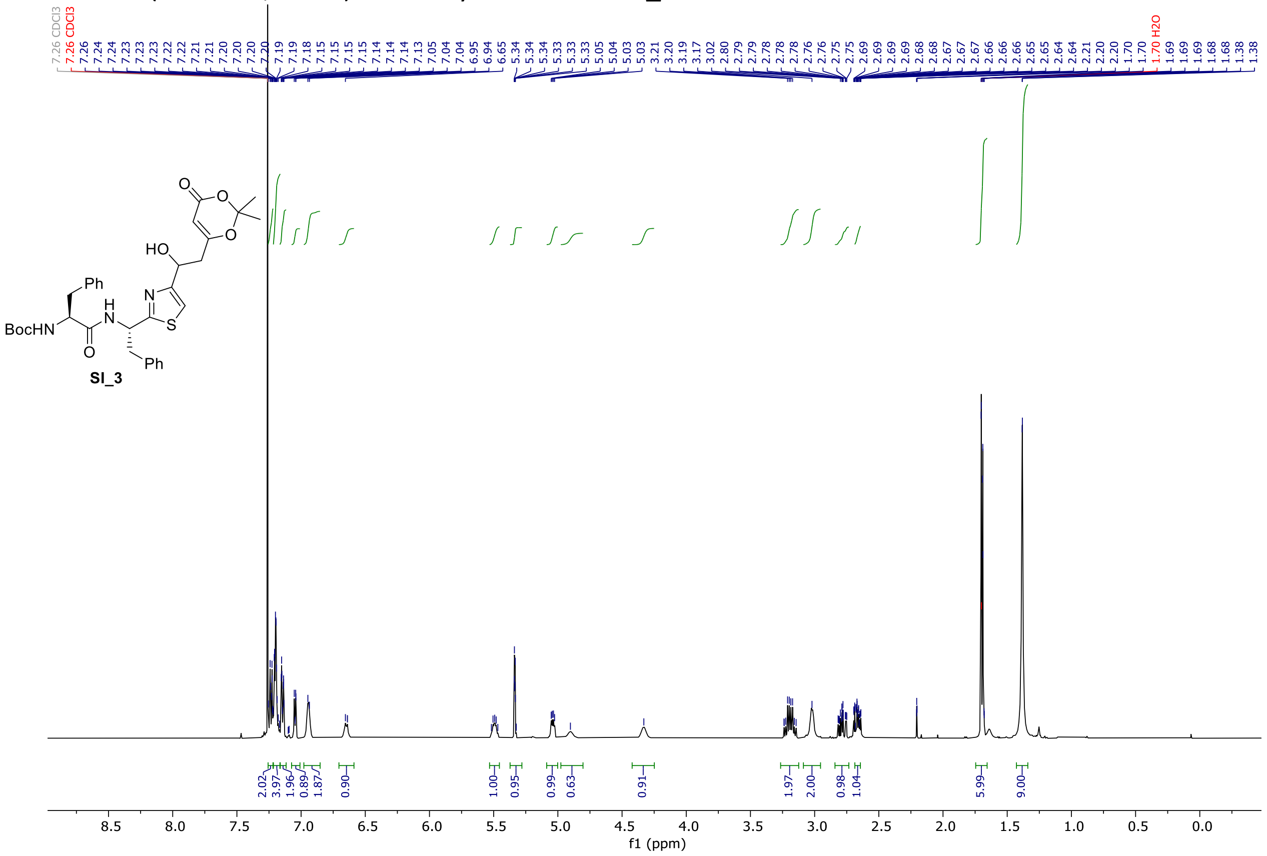

^{13}C NMR (151 MHz, $CDCl_3$) of Mukayiama adduct SI_3:

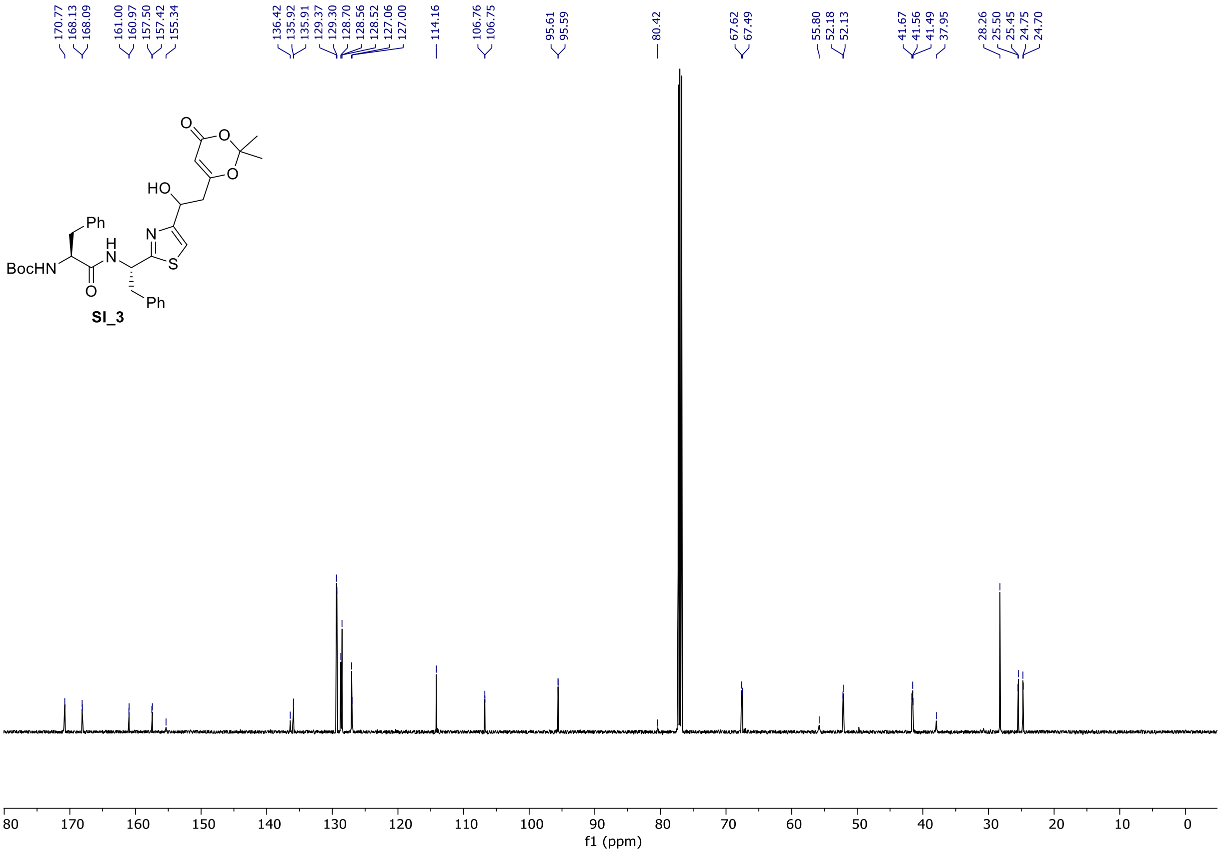

^{1}H NMR (400 MHz, $CDCl_3$) of Mukayiama Adduct 7:

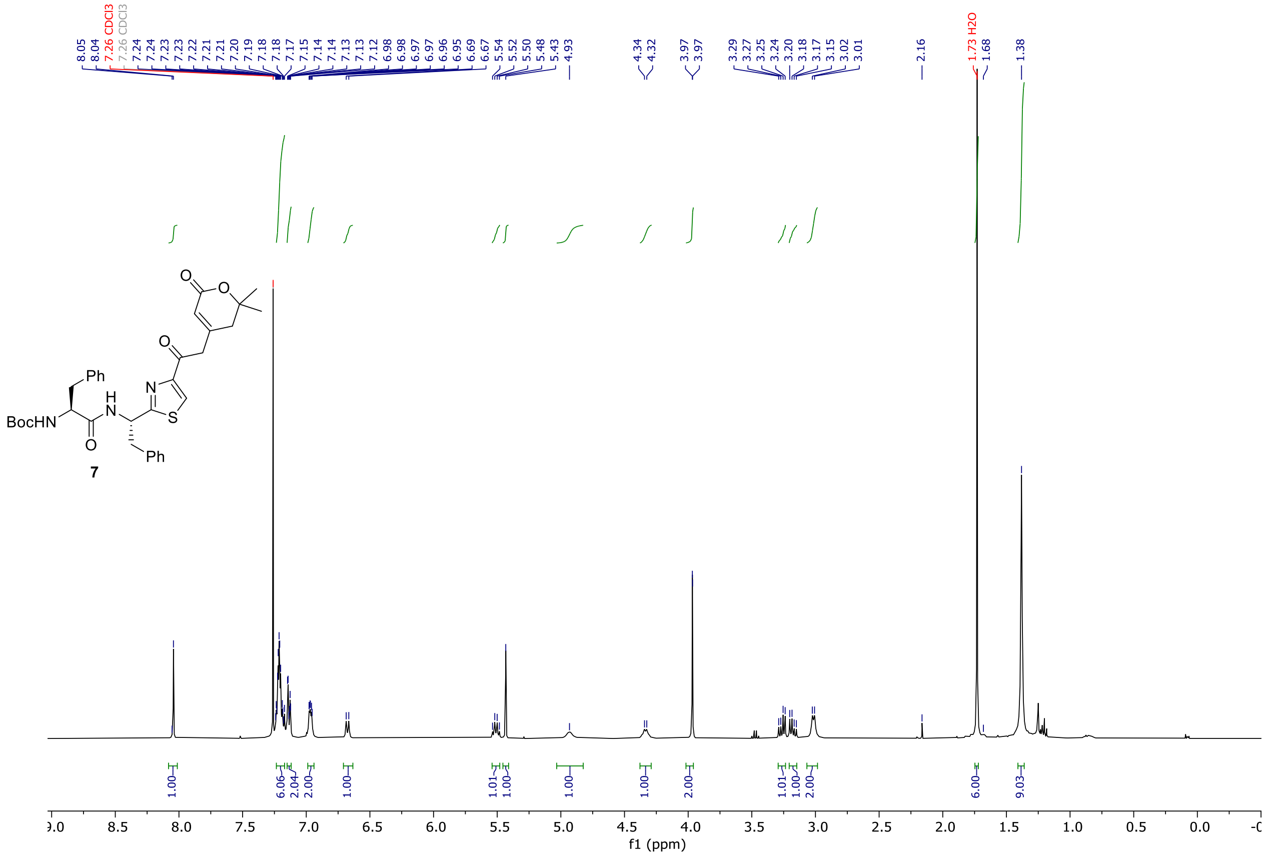

^{13}C NMR (101 MHz, $CDCl_3$) of Mukayiama Adduct 7:

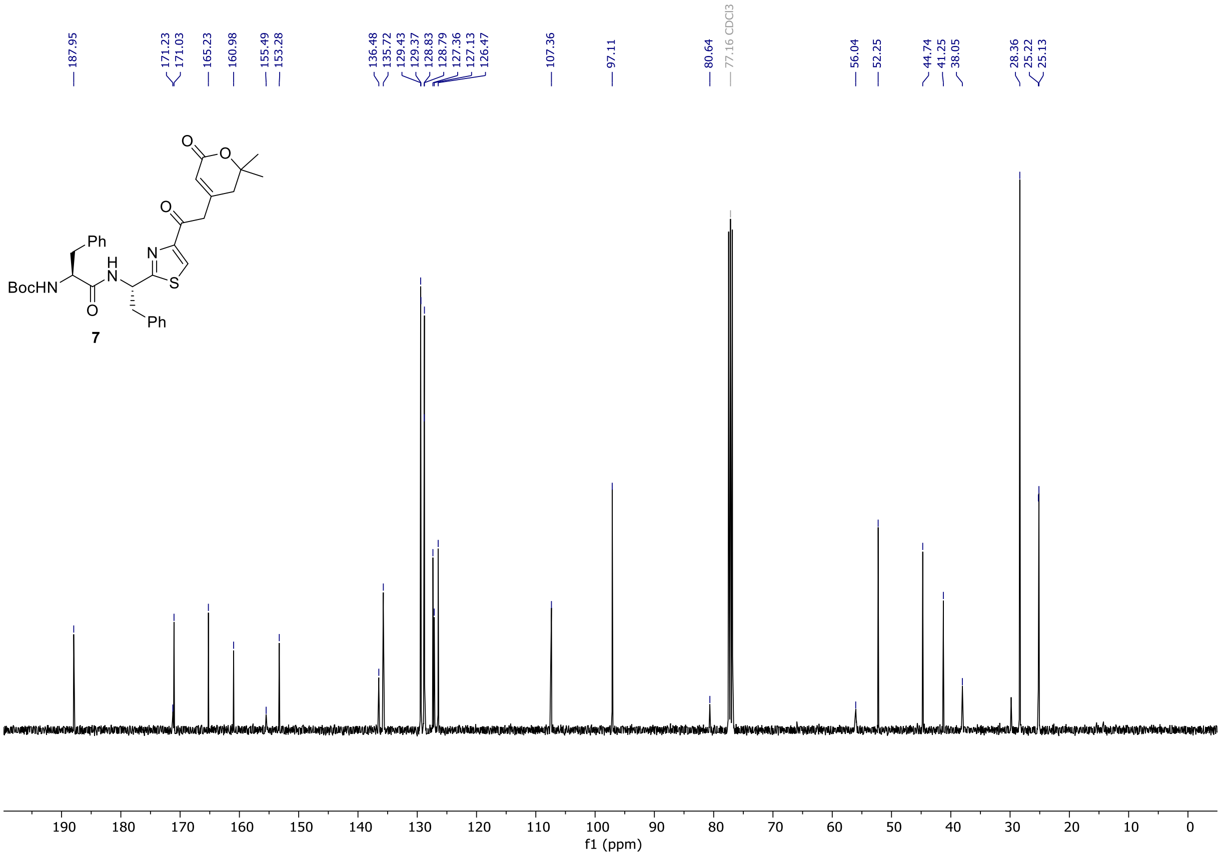

note: some peaks (171.2, 80.6, 56.0, 38.1) are considerably broadened due to presumably boc-induced rotamers.

^{1}H NMR (400 MHz, $CDCl_3$) of Phosphonate 13:

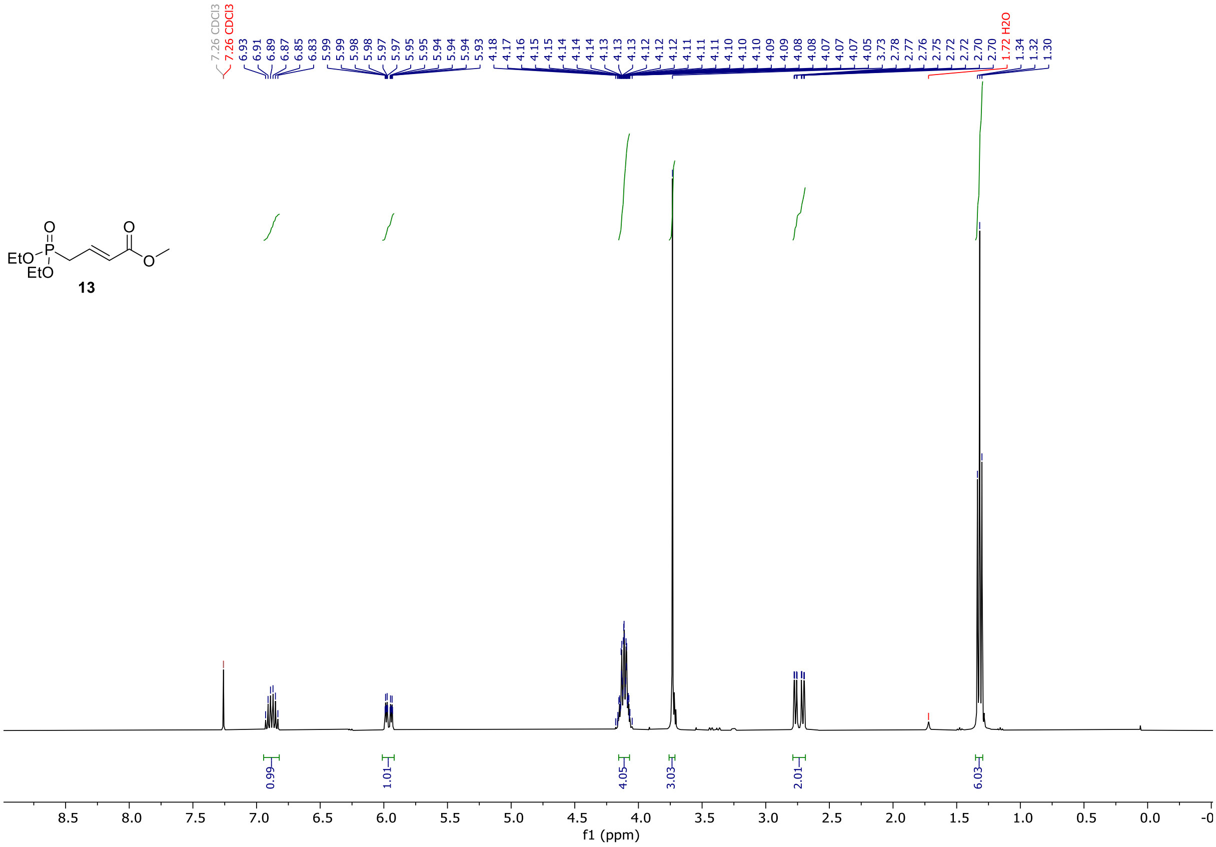

^{31}P NMR (162 MHz, $CDCl_3$) of Phosphonate 13:

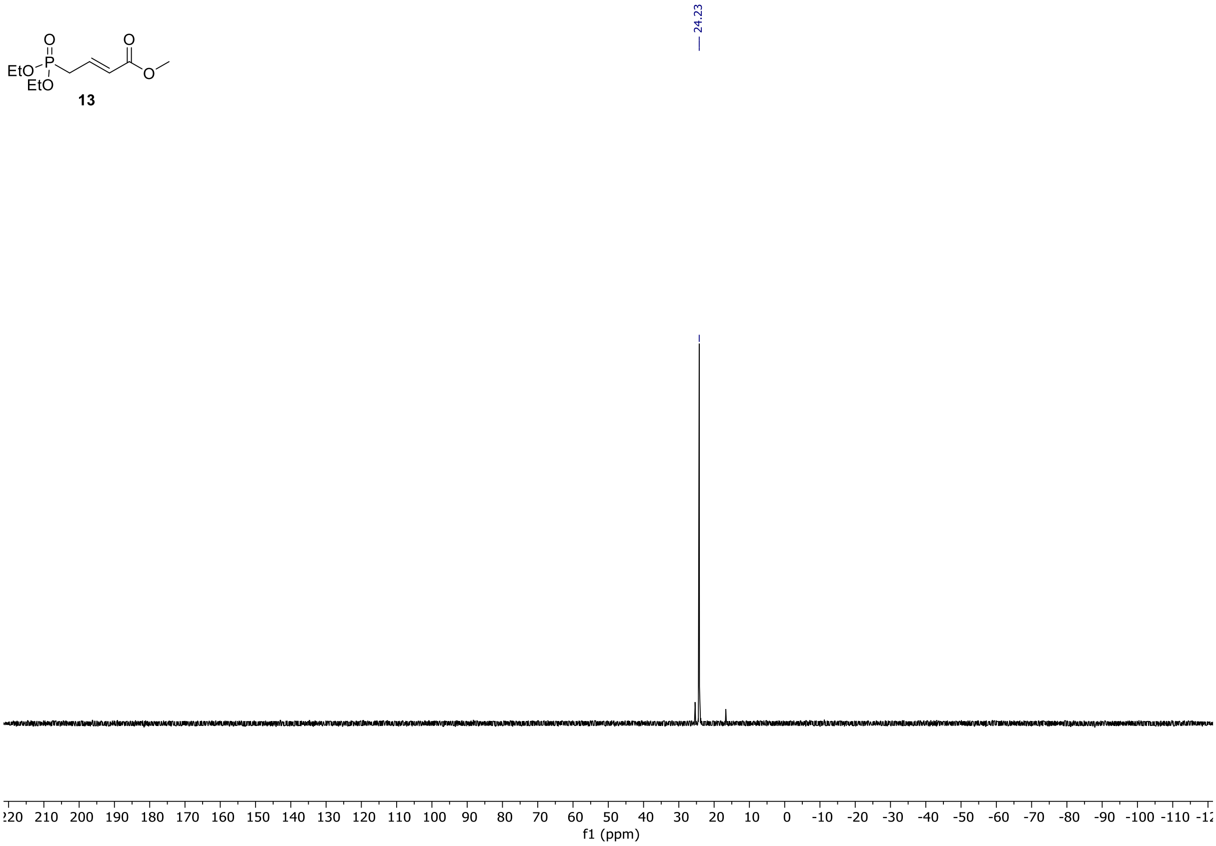

^{13}C NMR (101 MHz, $CDCl_3$) of Phosphonate 13:

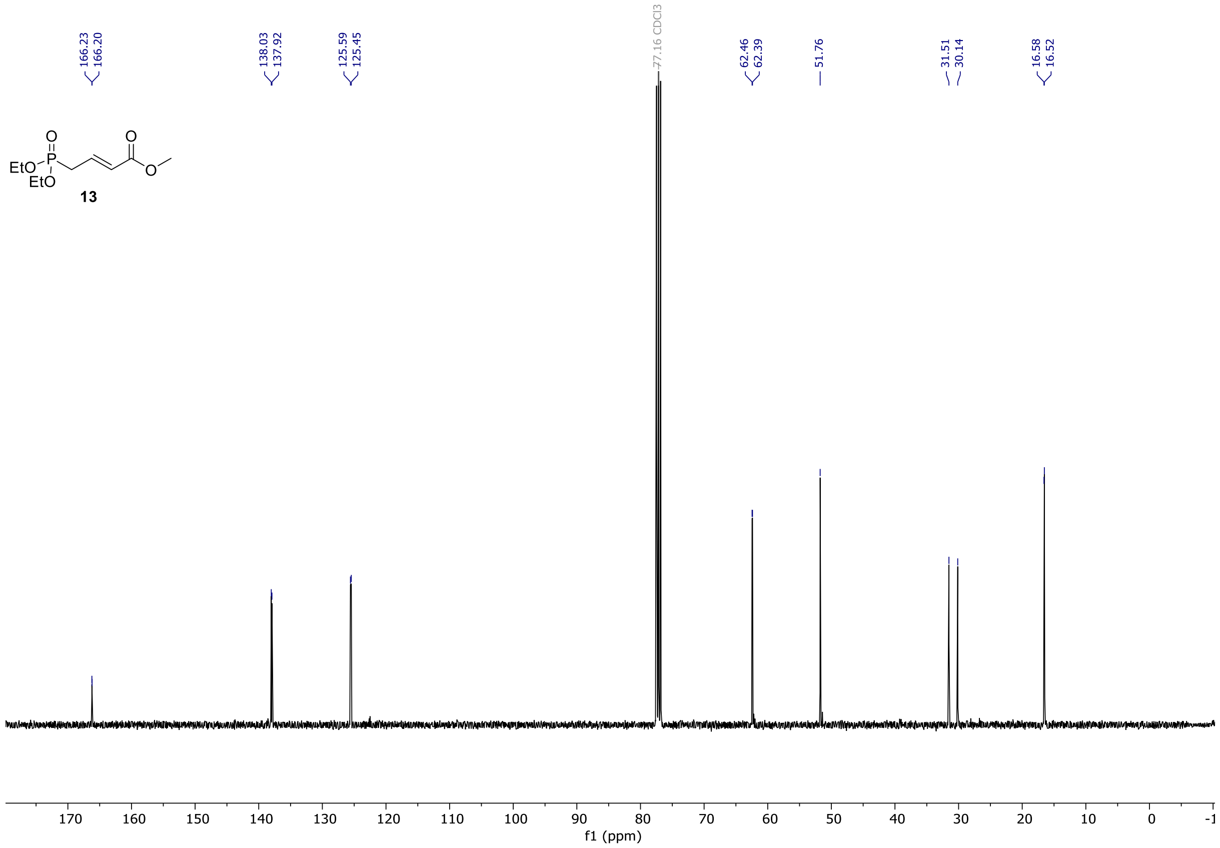

^1H NMR (400 MHz, $CDCl_3$) of Methyl ester SI_4:

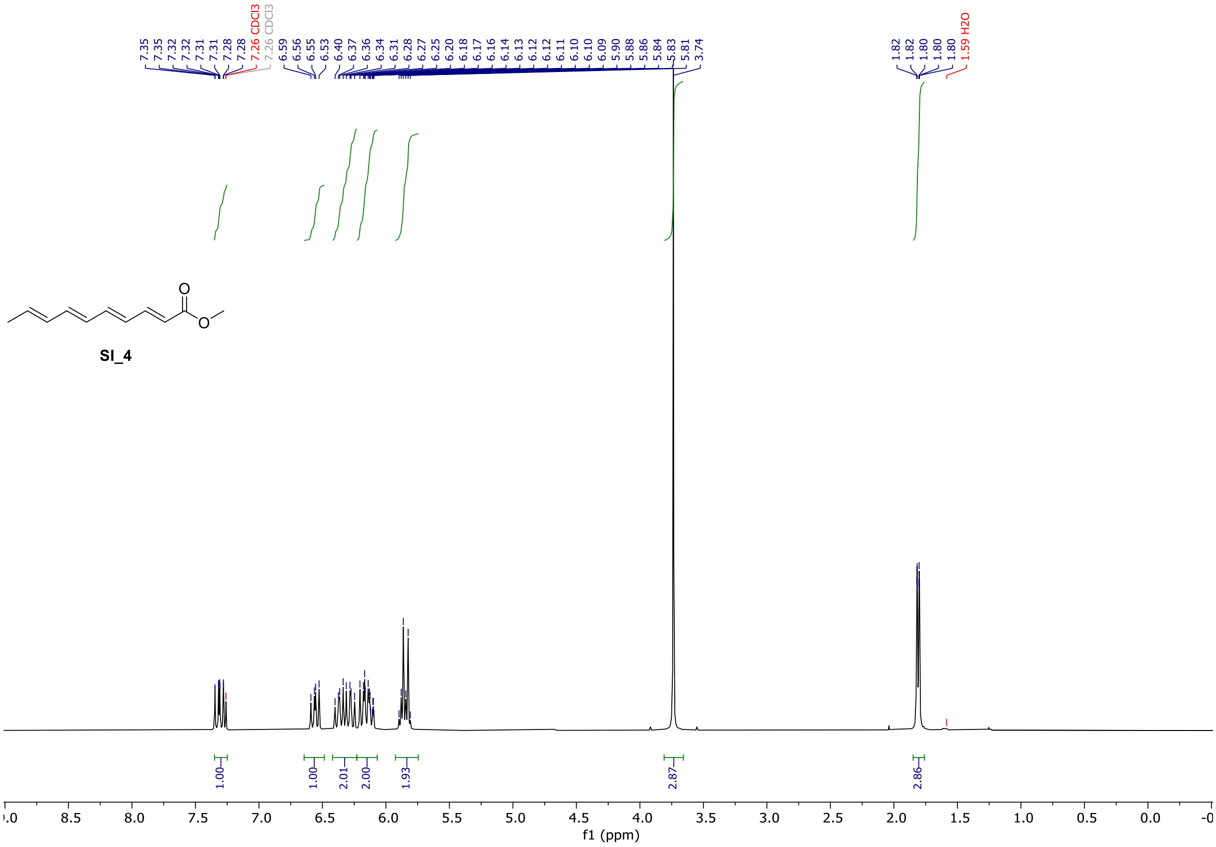

^{13}C NMR (101 MHz, $CDCl_3$) of Methyl ester SI_4:

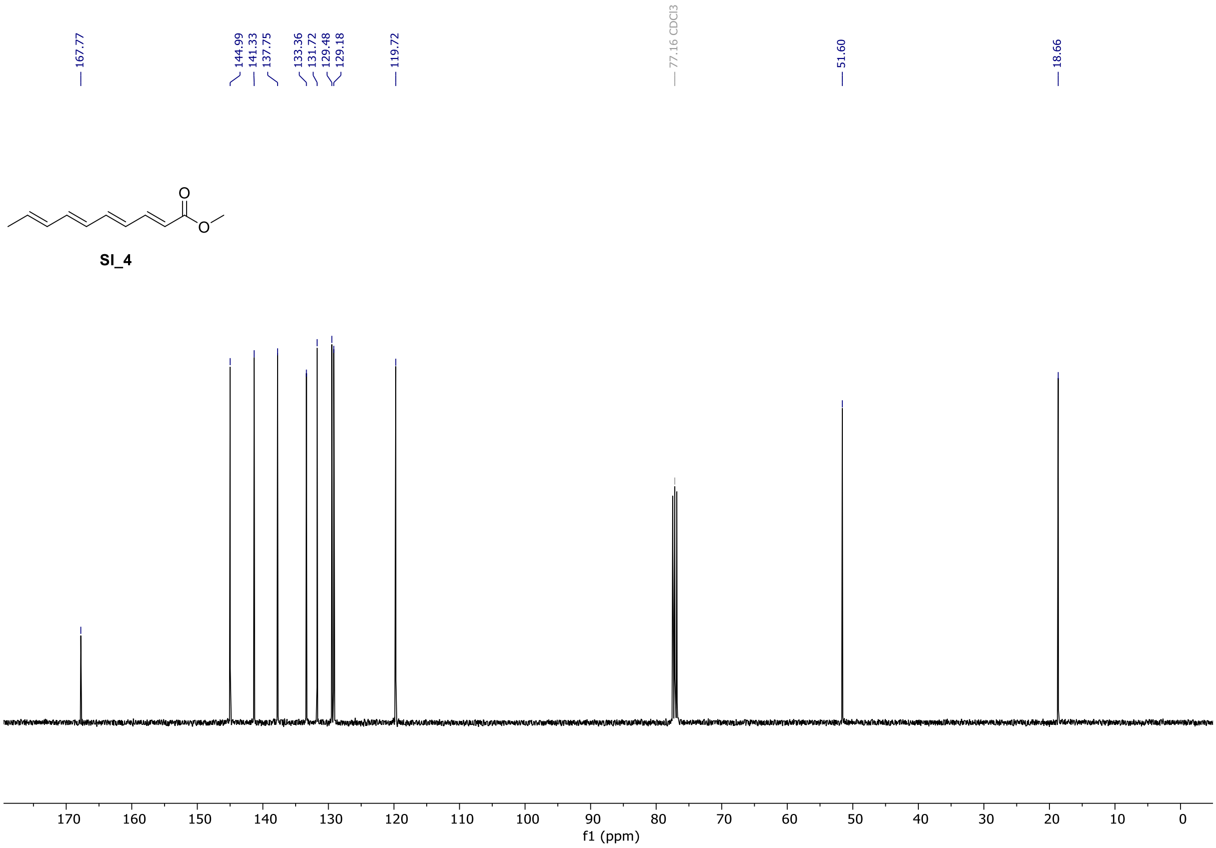

^{1}H NMR (500 MHz, DMSO-d6) of Tetraenoic Acid 14:

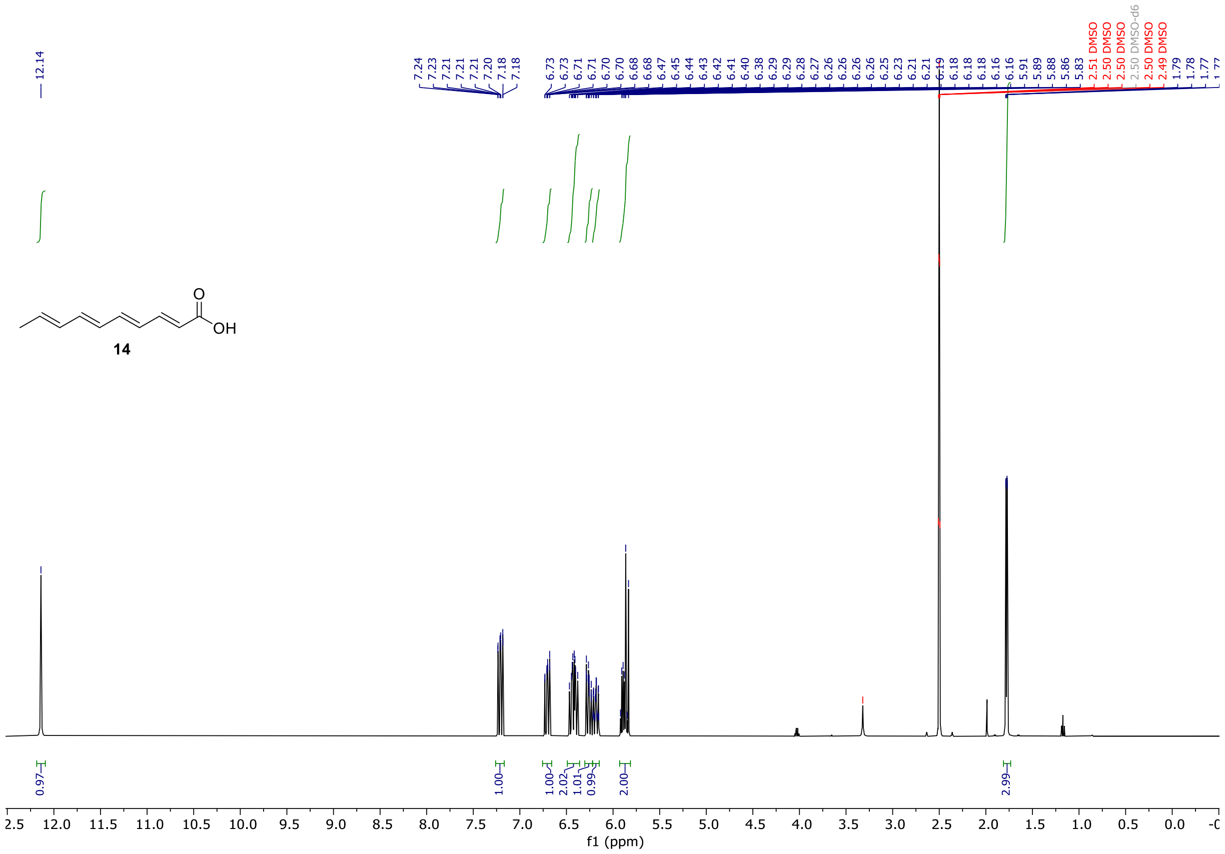

^{13}C NMR (151 MHz, DMSO-d6) of Tetraenoic Acid 14:

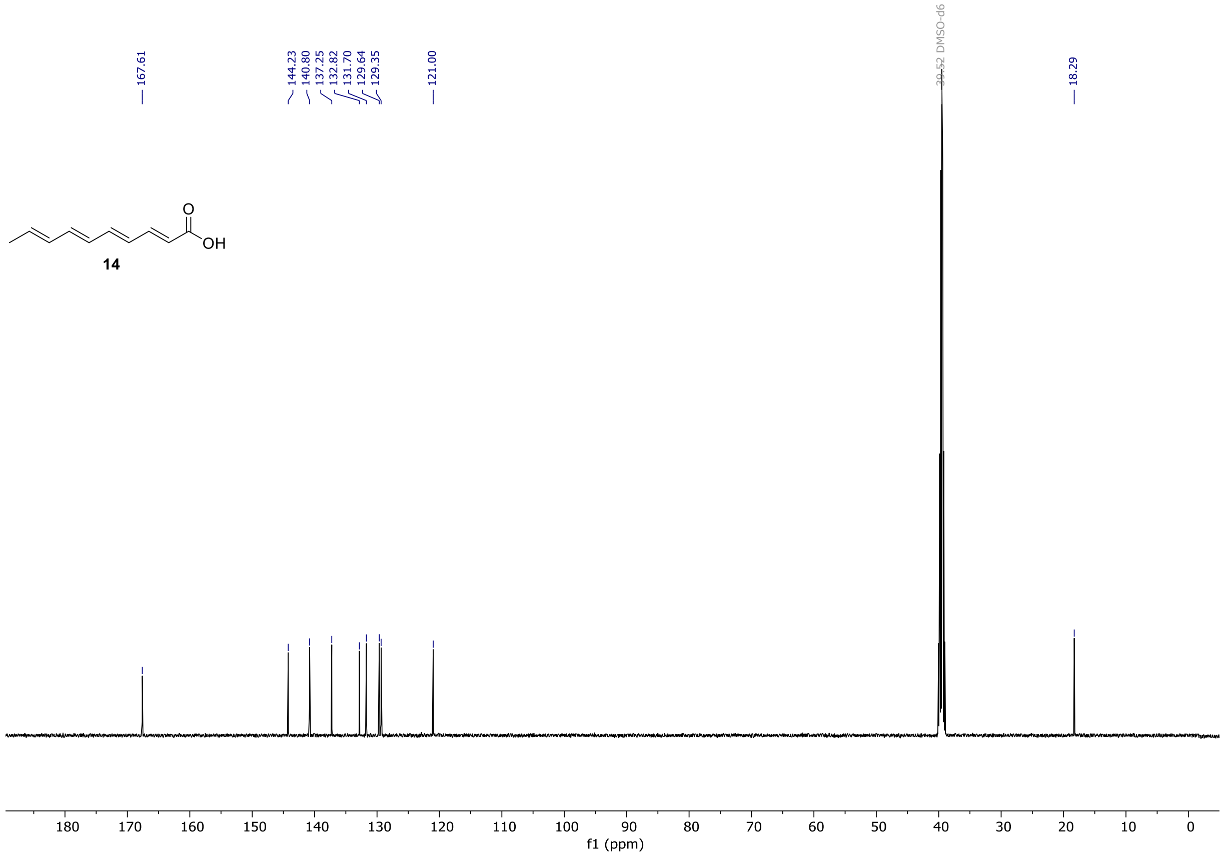

^{1}H NMR (400 MHz, $CDCl_3$) of Acyl fluoride 15:

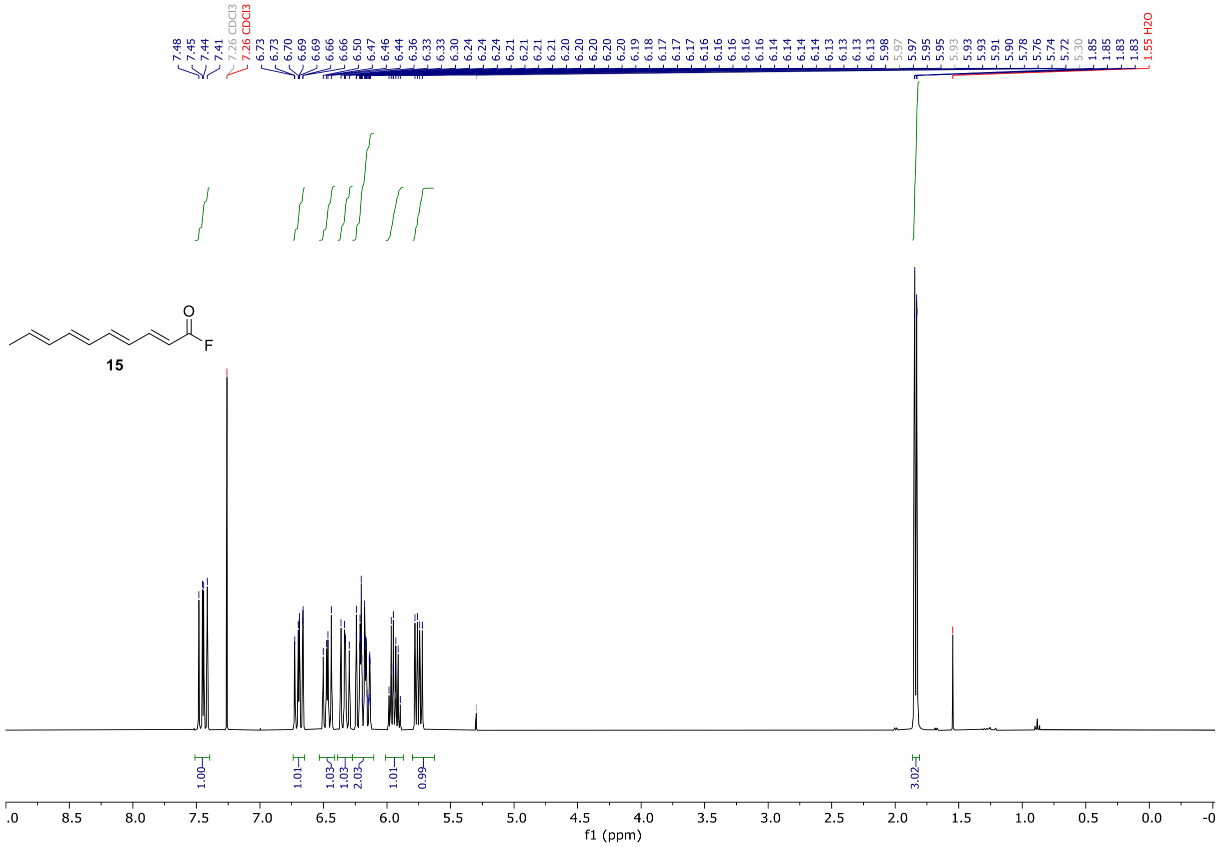

^{13}C NMR (101 MHz, $CDCl_3$) of Acyl fluoride 15:

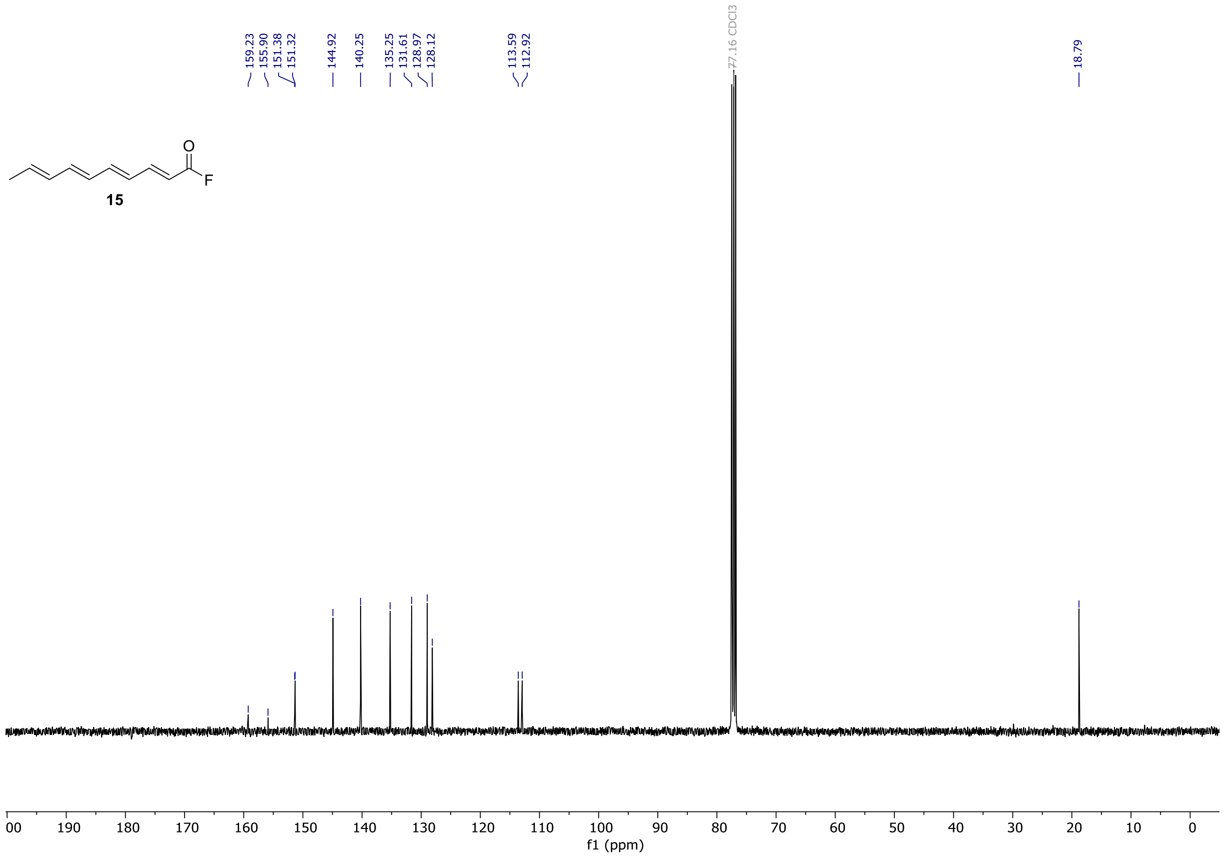

^{19}F NMR (282 MHz, $CDCl_3$) of Acyl fluoride 15:

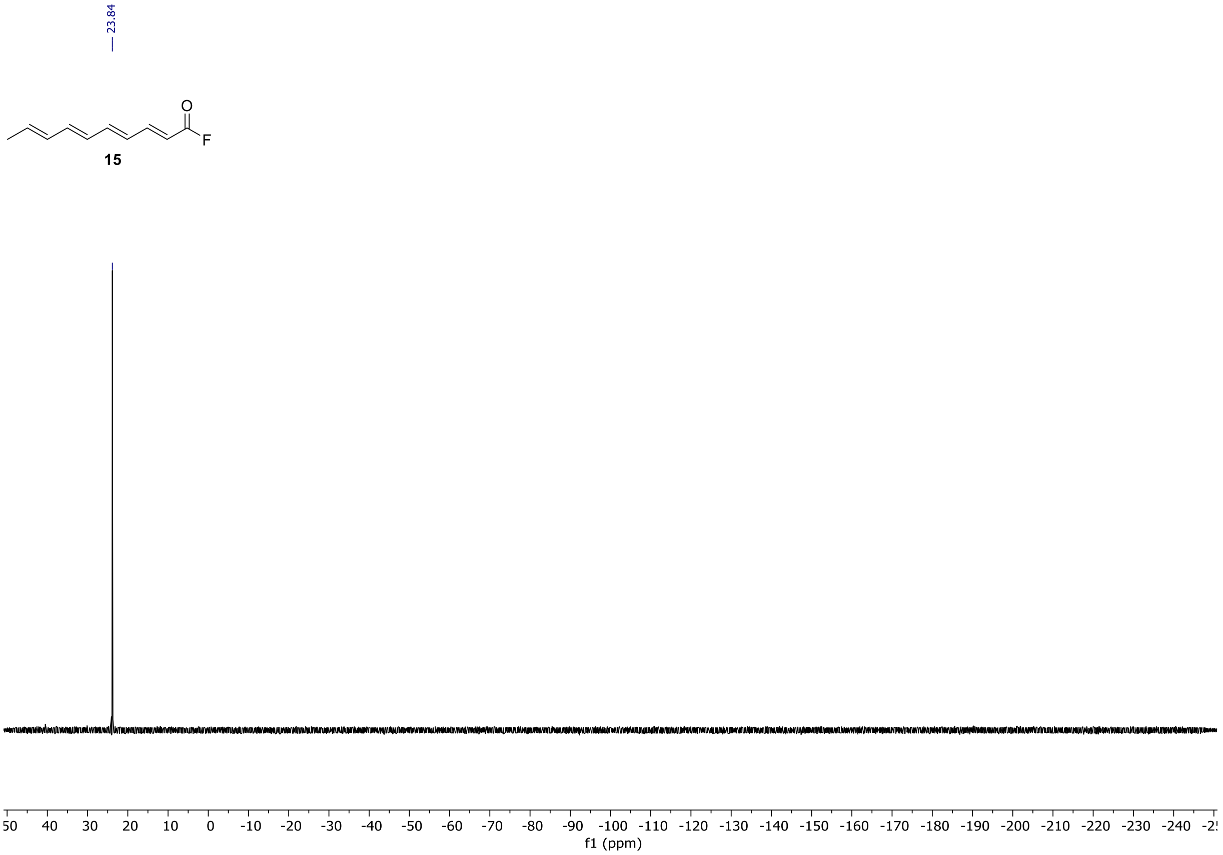

^{1}H NMR (500 MHz, $CDCl_3$) of Pyrone SI_5:

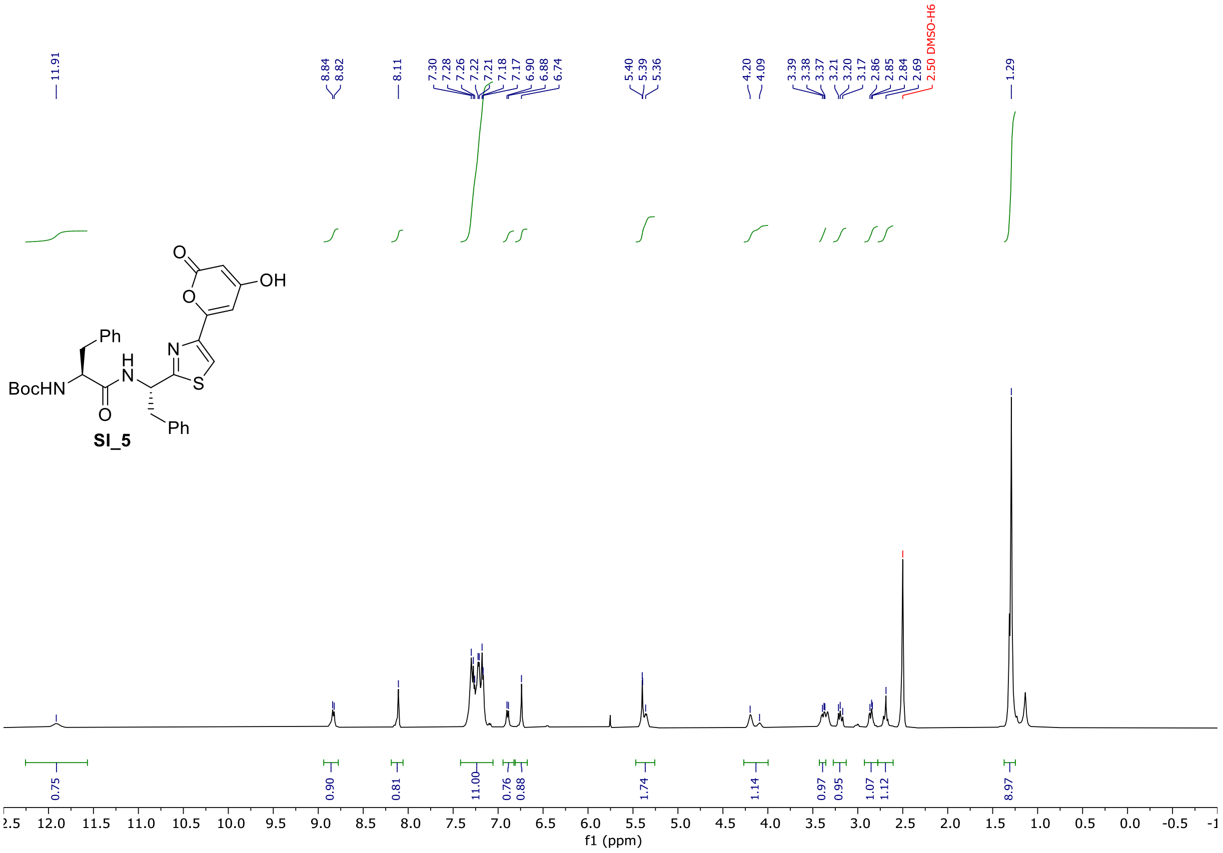

^{13}C NMR (151 MHz, $CDCl_3$) of Pyrone SI_5:

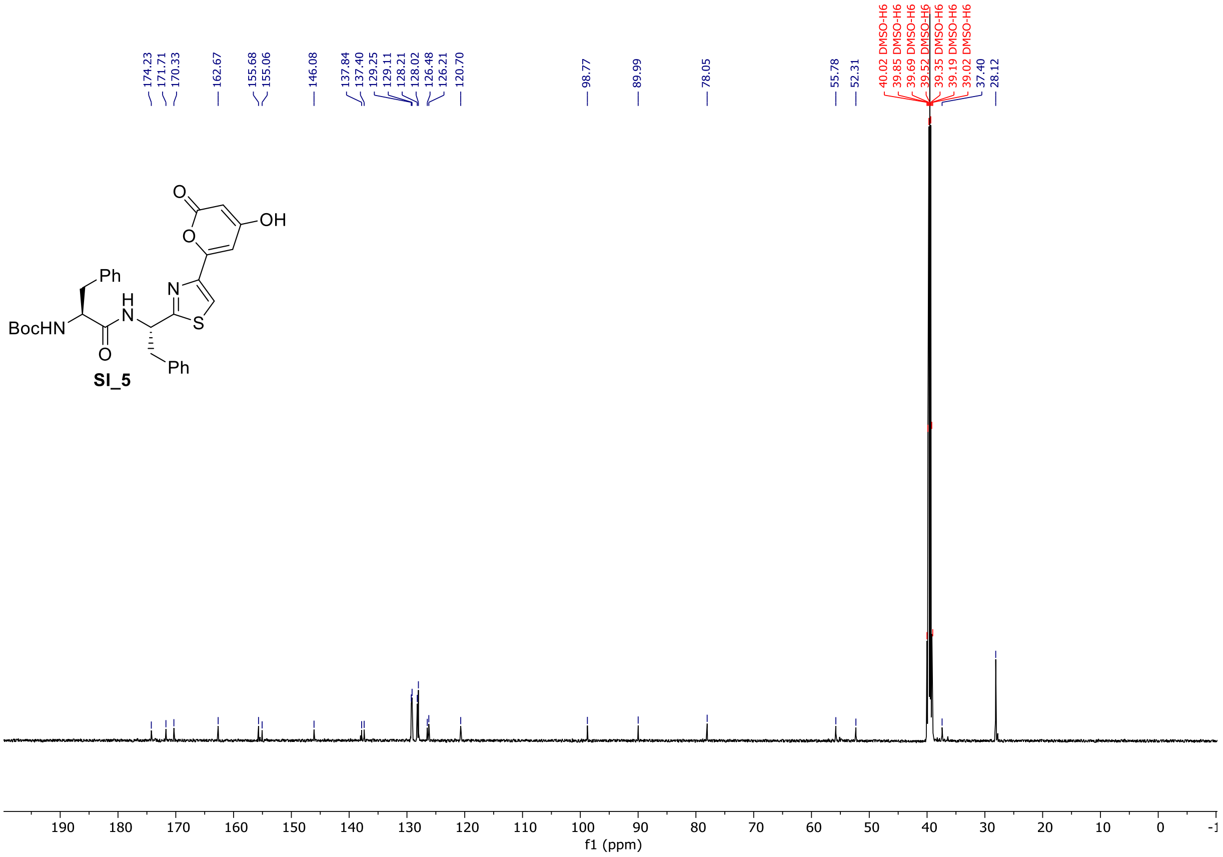

^{1}H NMR (500 MHz, $CDCl_3$) of Mutanofactin 607:

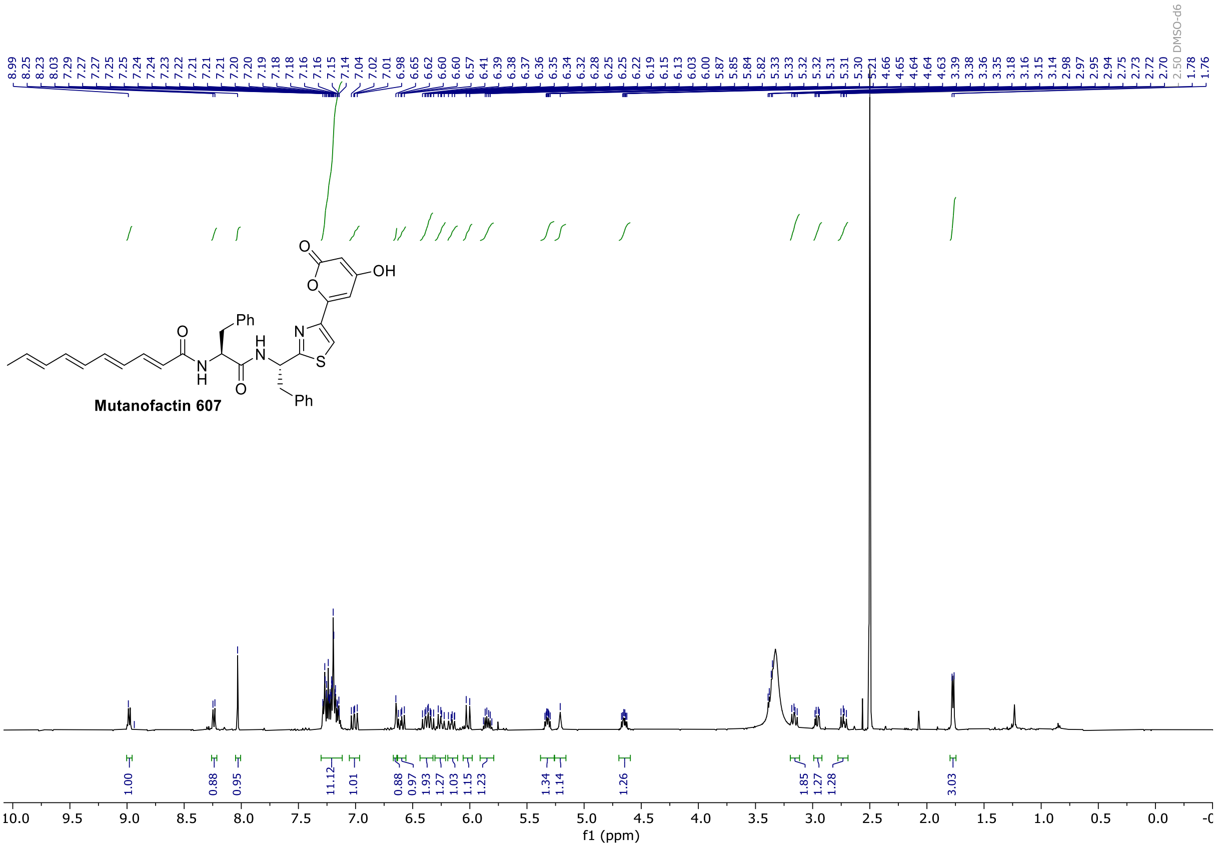

[13]H NMR (151 MHz, $CDCl_3$) of Mutanofactin 607:

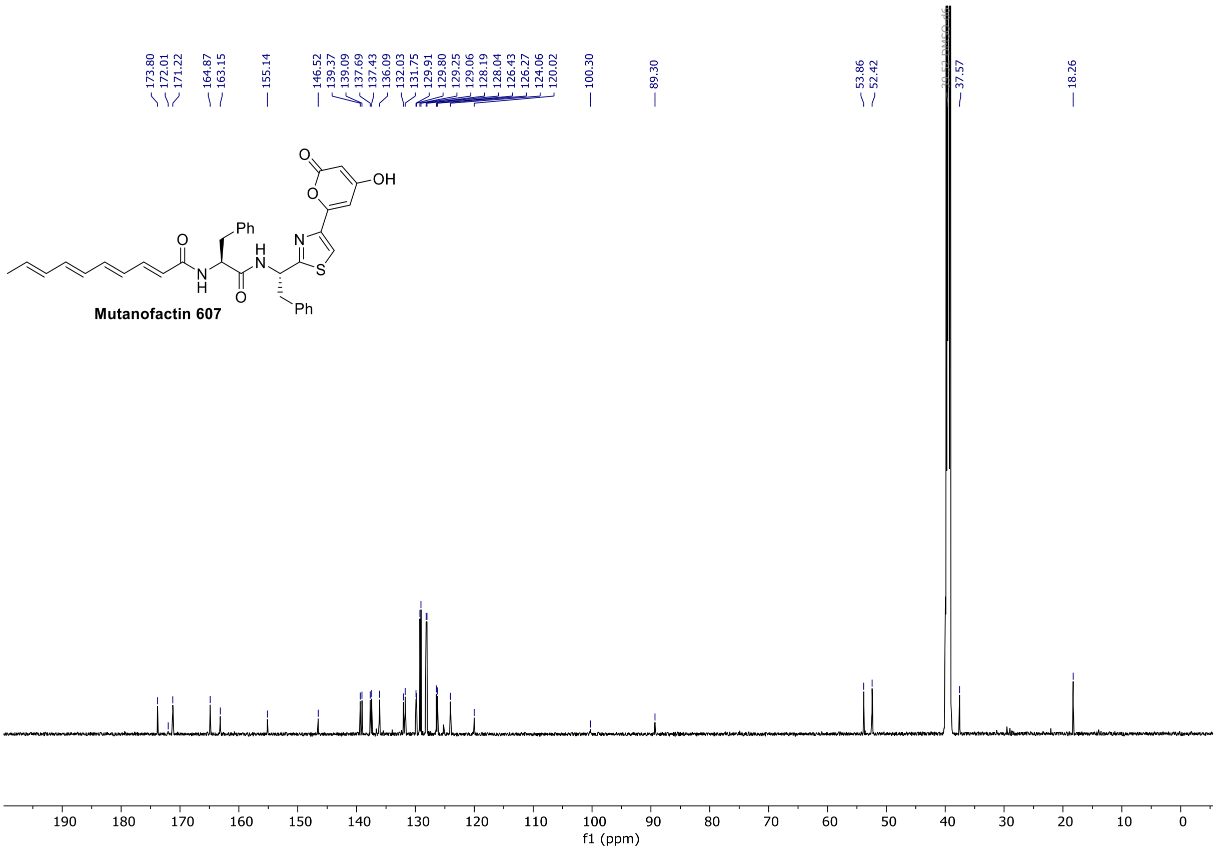

COSY (DMSO-d6) of Mutanofactin 607:

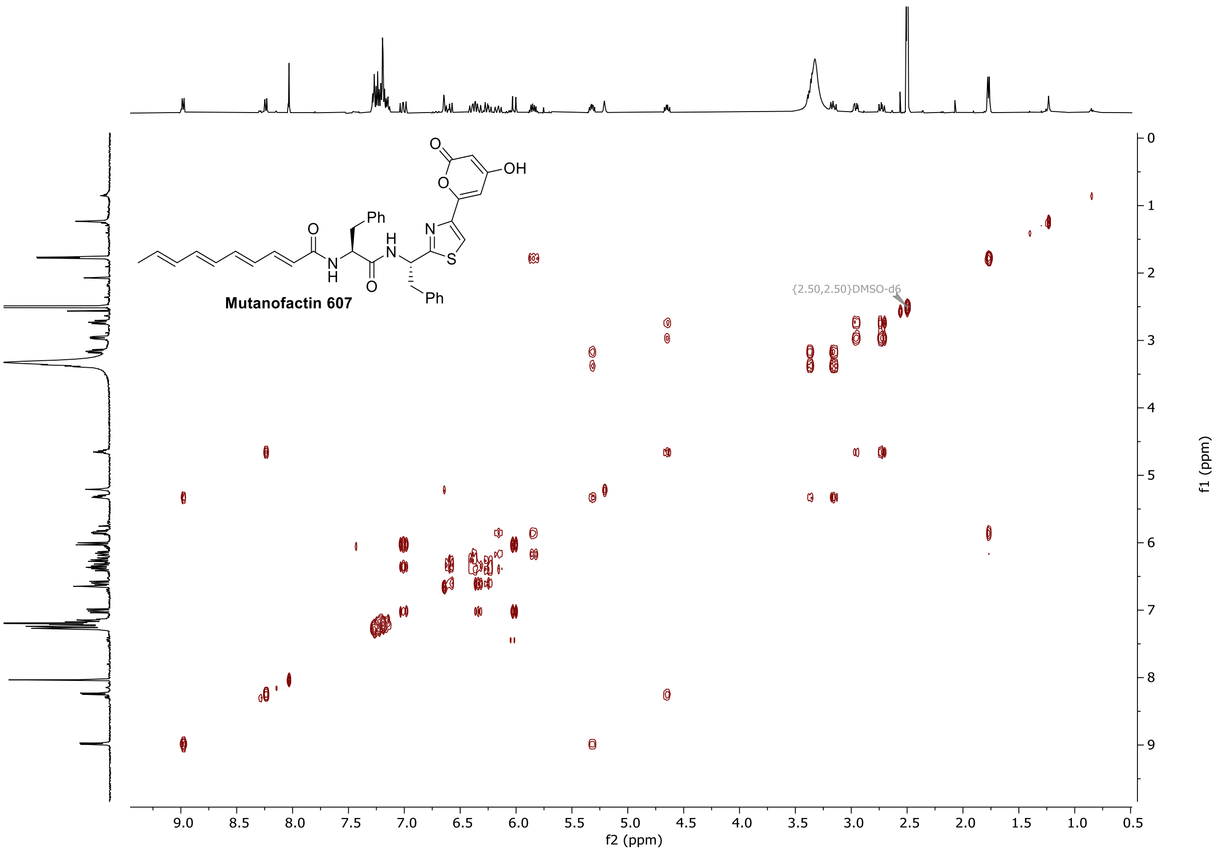

HSQC (DMSO-d6) of Mutanofactin 607:

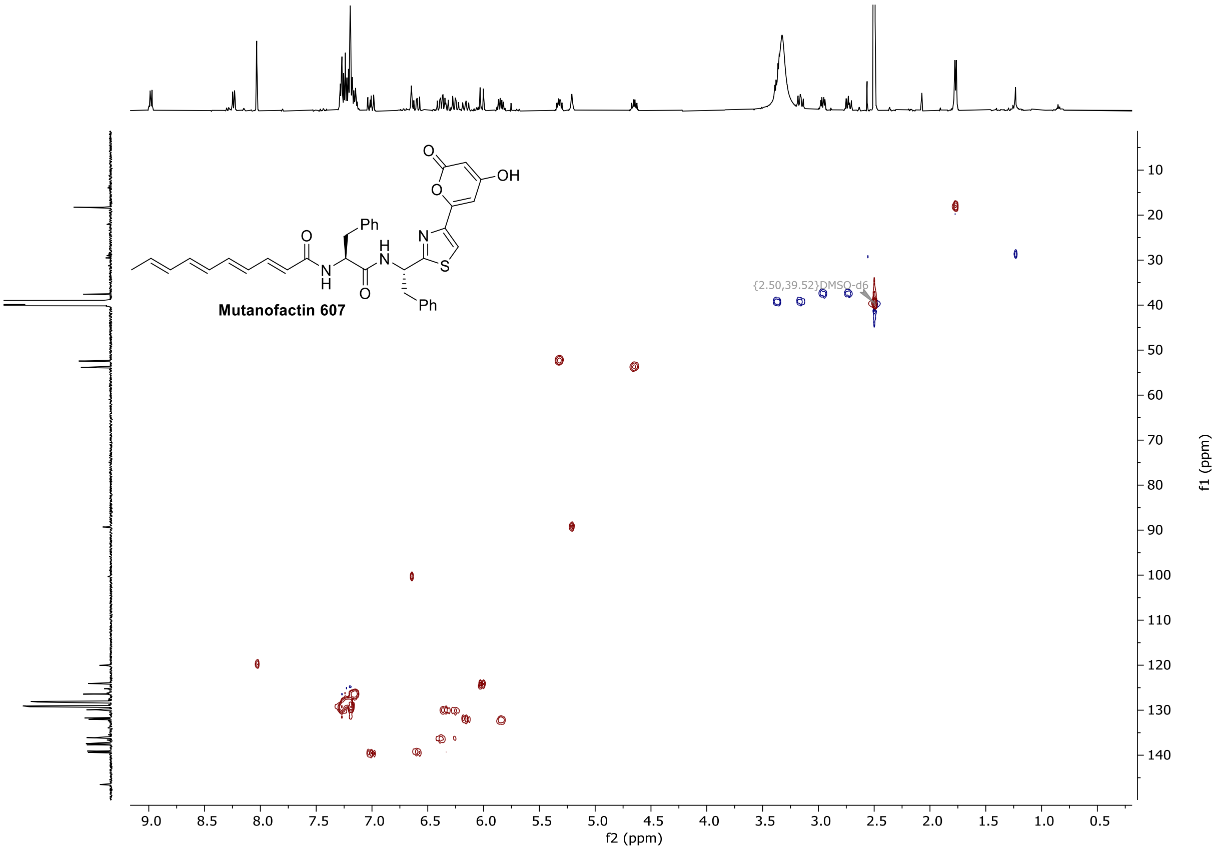

HMBC (DMSO-d6) of Mutanofactin 607:

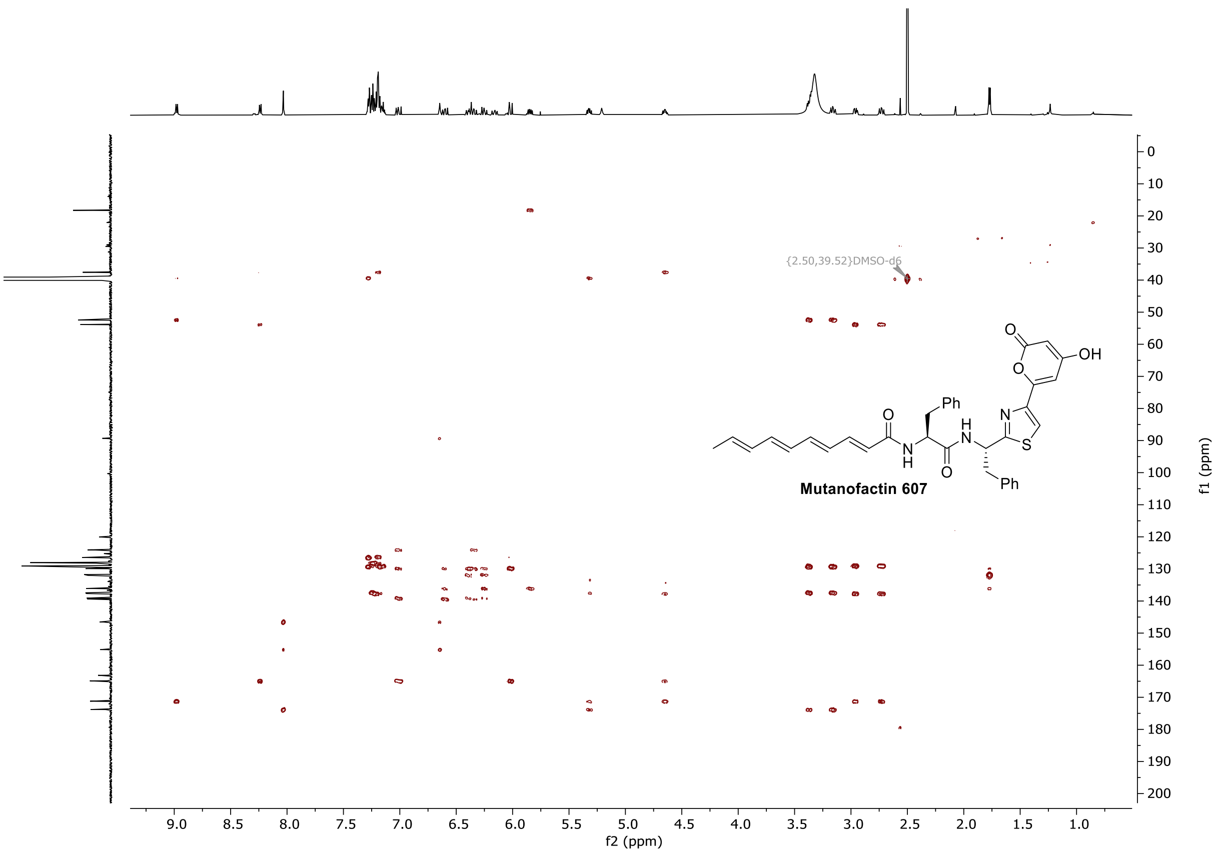
{2.50,39.52}DMSO-d6
Mutanofactin 607
f1 (ppm)
f2 (ppm)

^{1}H NMR (400 MHz, $CDCl_3$) of PMB-ether SI_6:

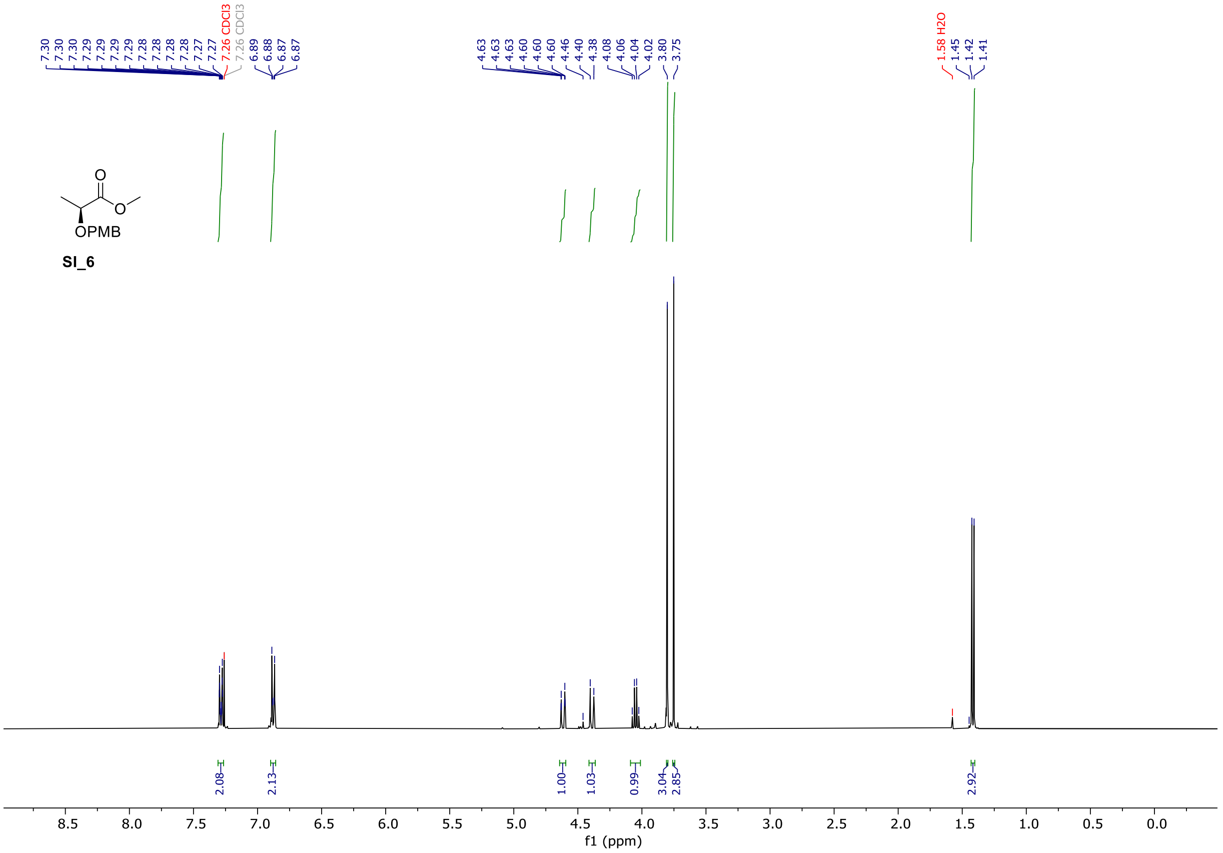

^{13}C NMR (400 MHz, $CDCl_3$) of PMB-ether SI_6:

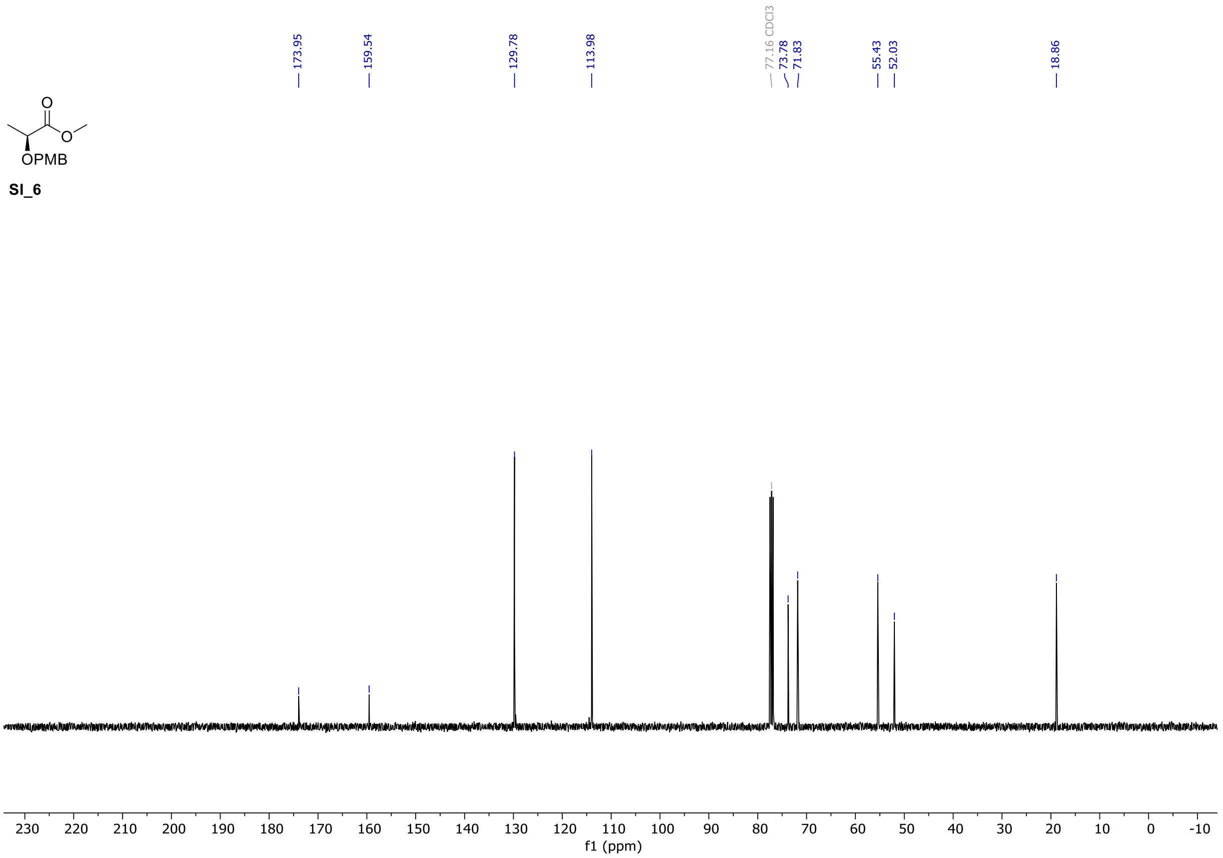

^1H NMR (400 MHz, DMSO-d6) of Acid S_7:

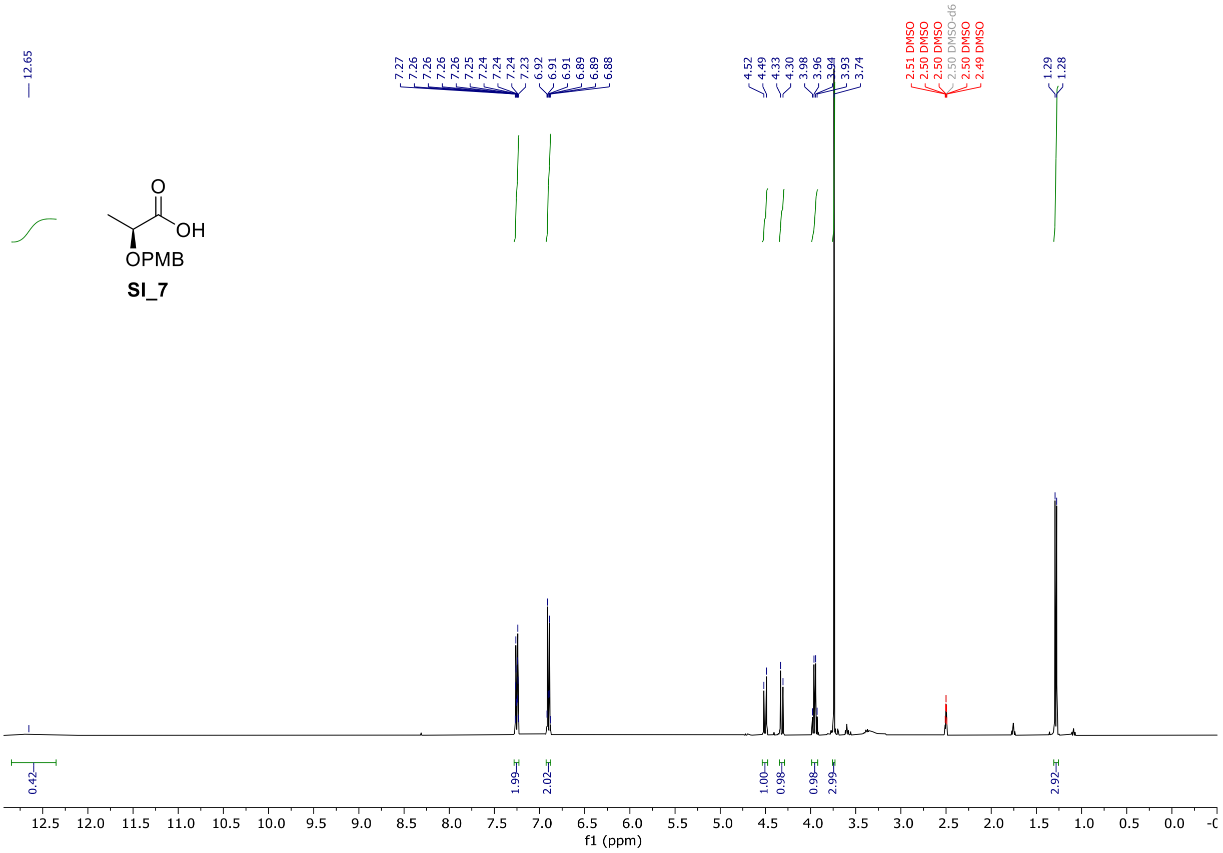

^{13}C NMR (101 MHz, DMSO-d6) of Acid S_7:

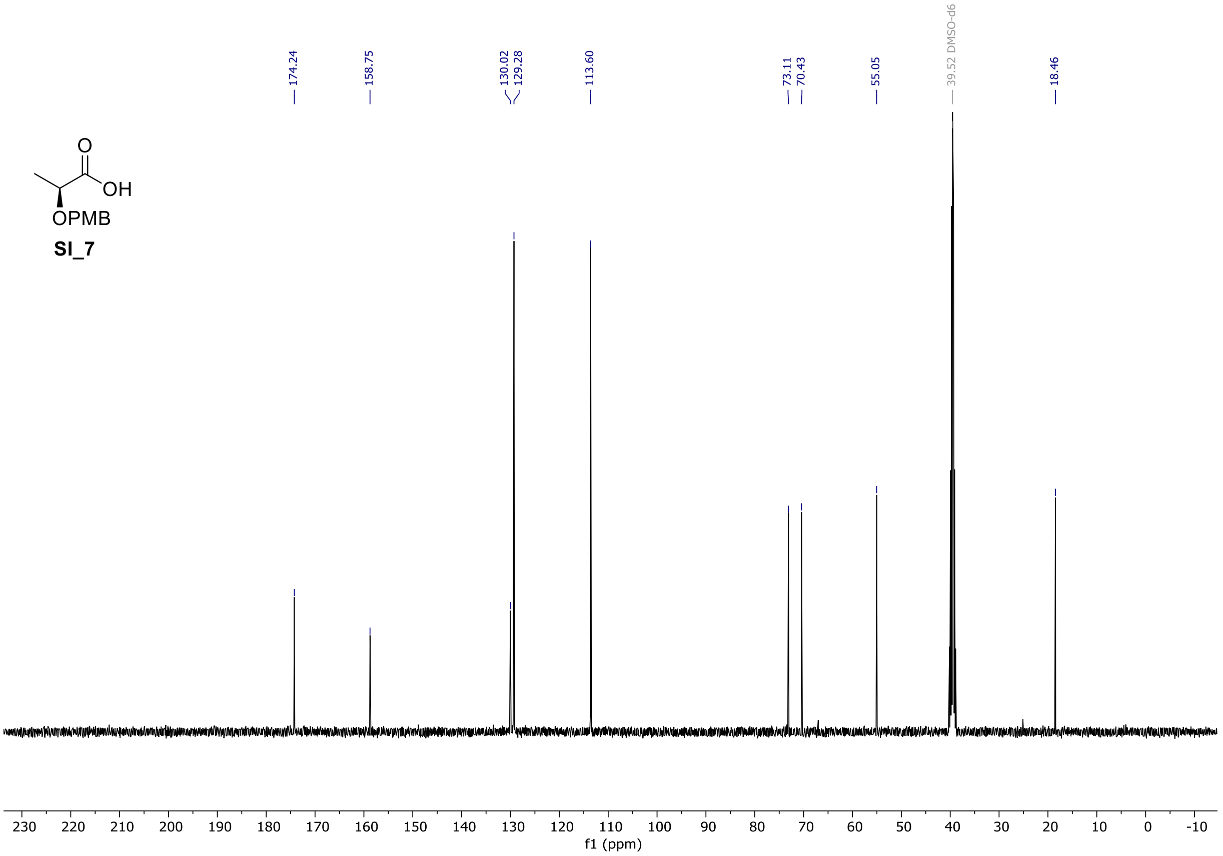

^{1}H NMR (400 MHz, $CDCl_3$) of *tert*-butyl ester SI_8:

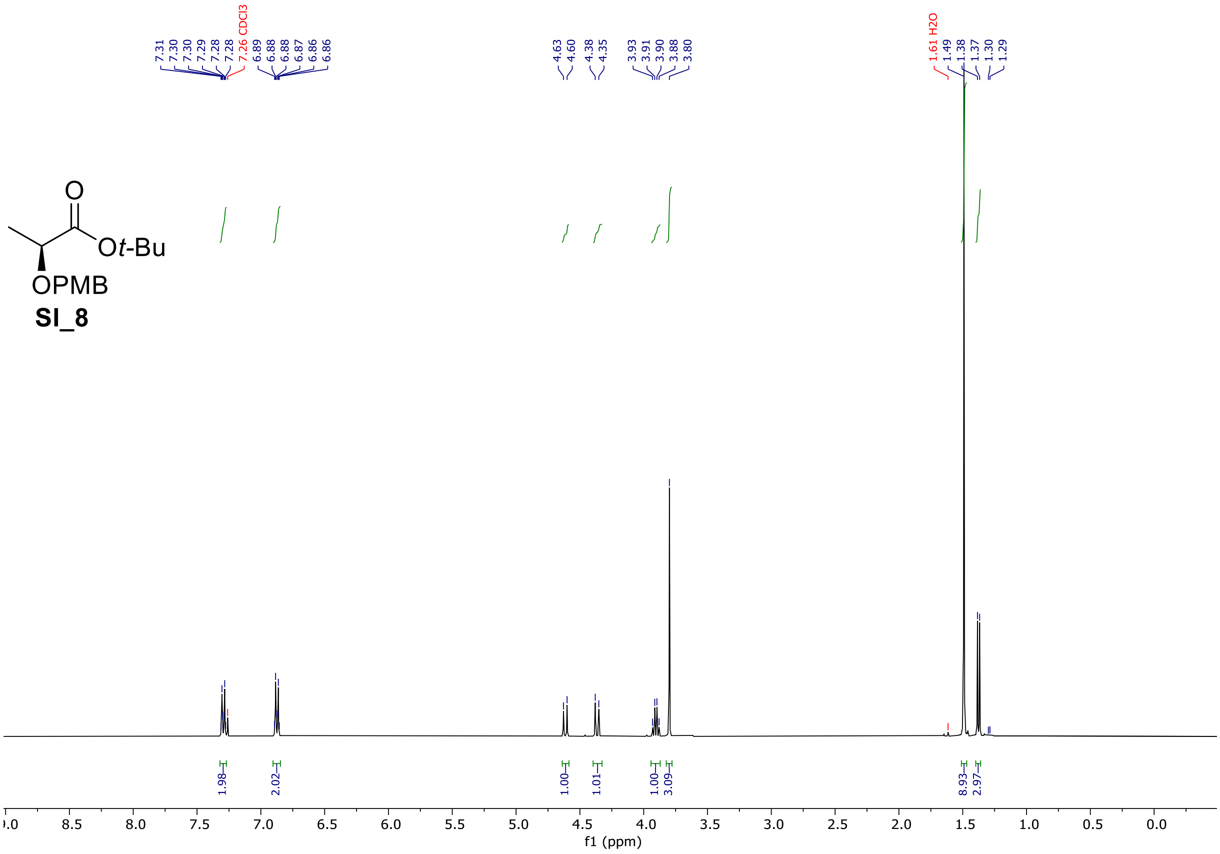

^{13}C NMR (101 MHz, $CDCl_3$) of *tert*-butyl ester SI_8:

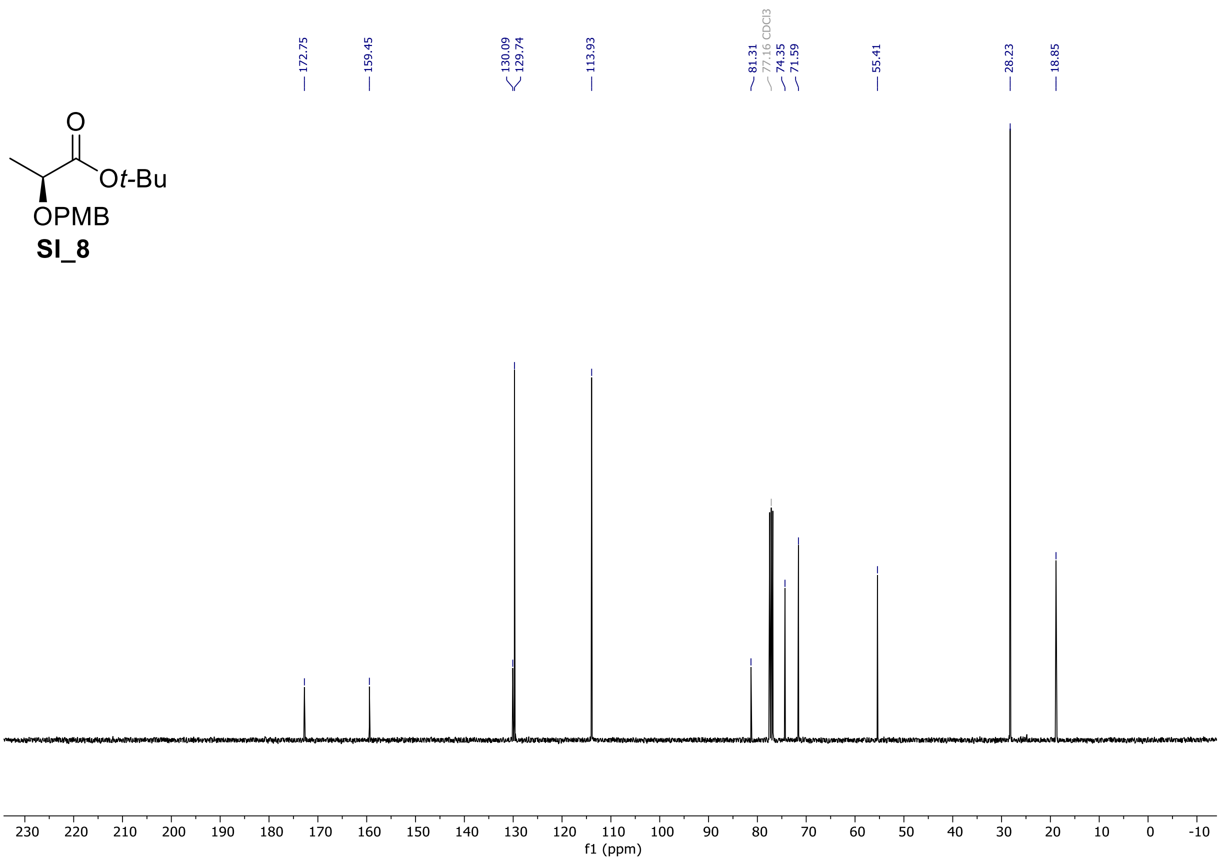

^1H NMR (400 MHz, $CDCl_3$) of Lactate SI_9:

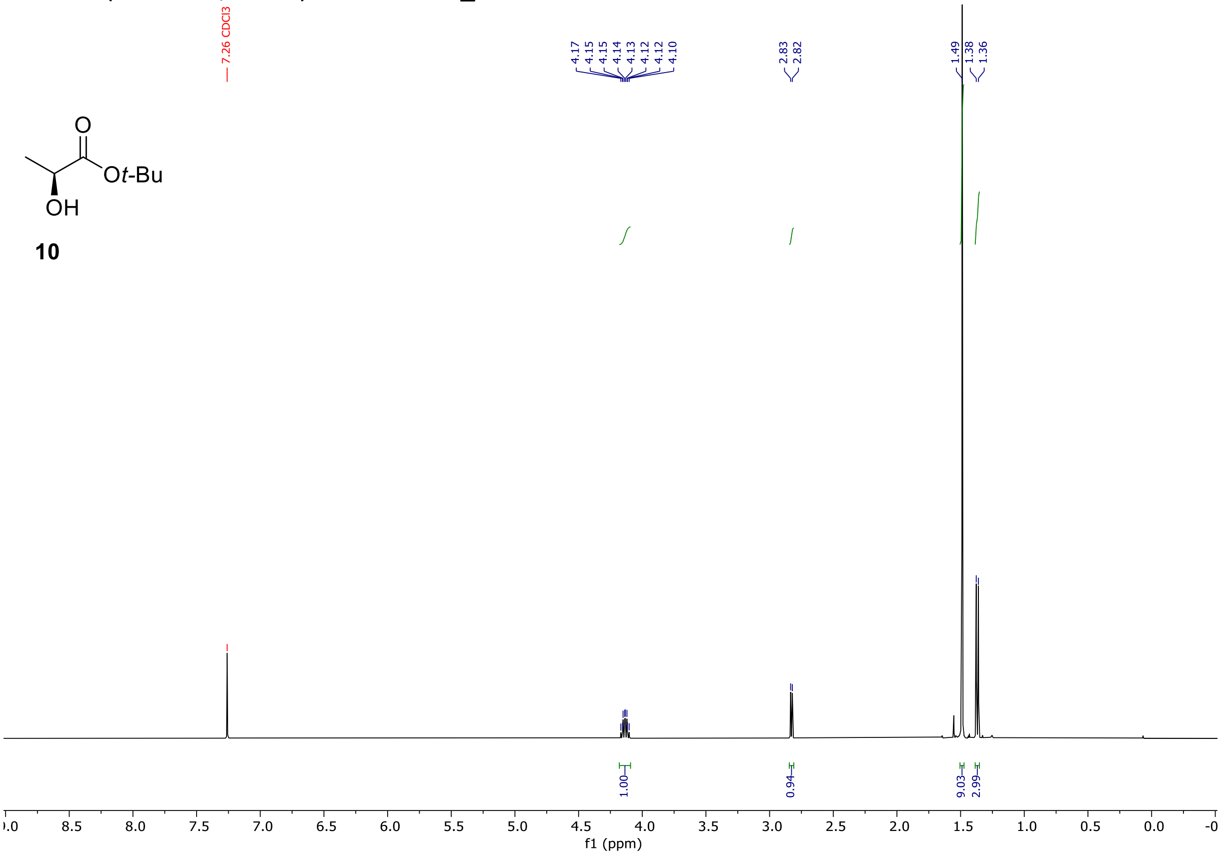

^{13}C NMR (101 MHz, $CDCl_3$) of Lactate SI_9:

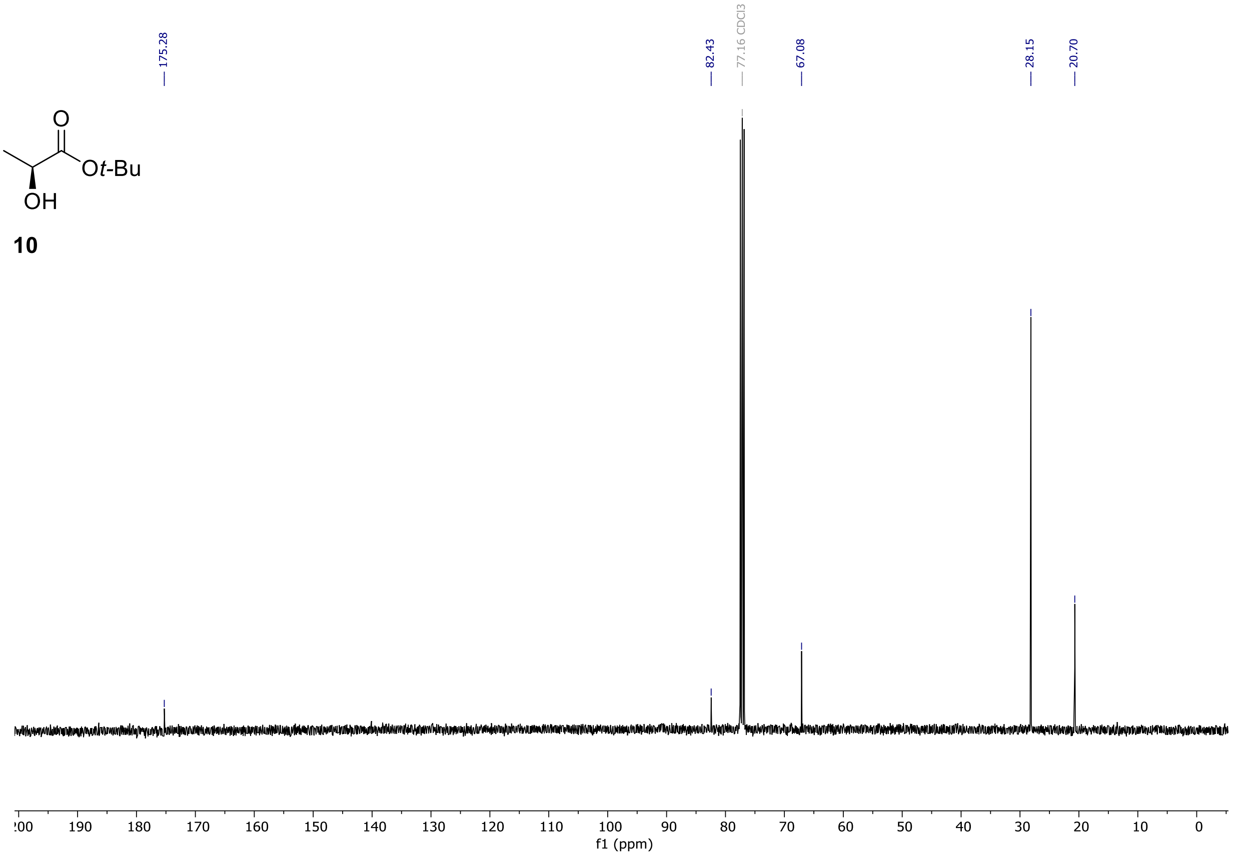

^{1}H NMR (500 MHz, DMSO-d6) of Lactate adduct **SI_9**:

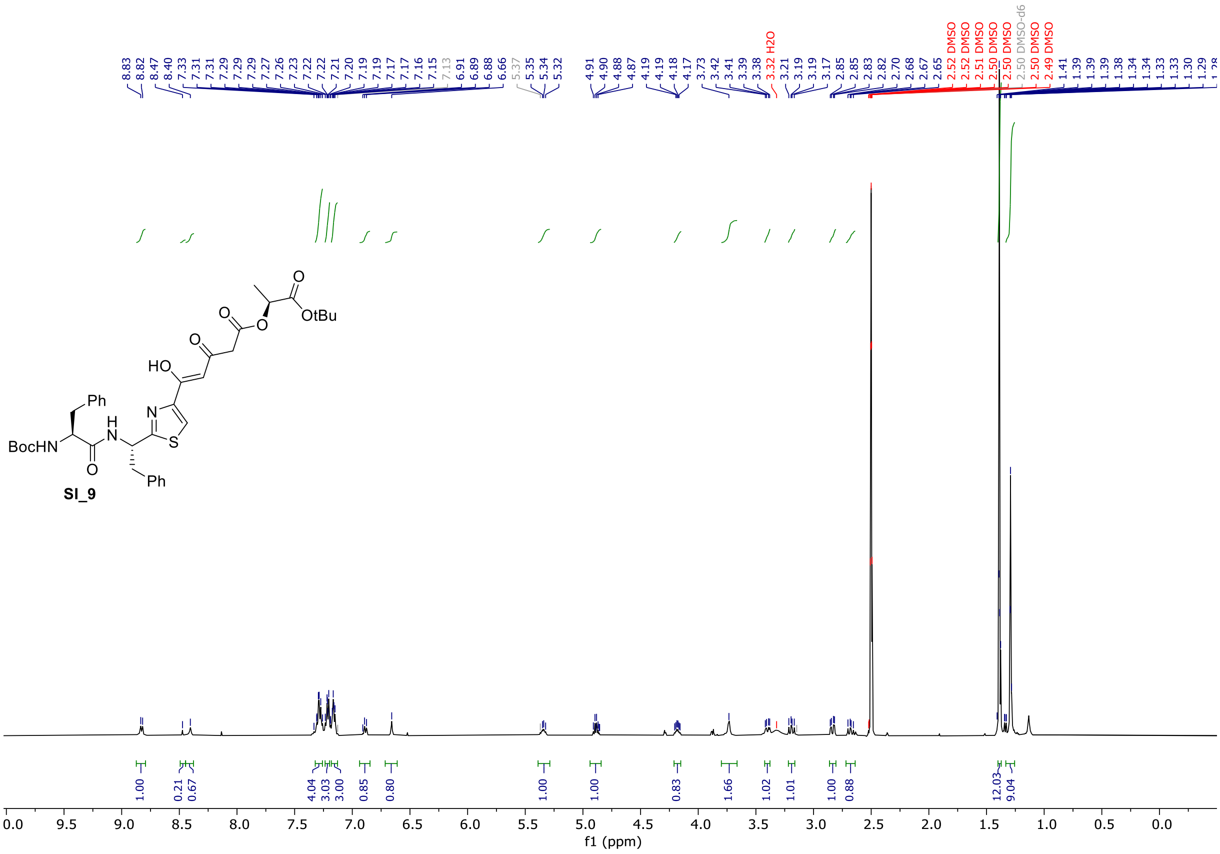

^{13}C NMR (151 MHz, DMSO-d6) of Lactate adduct **SI_9**:

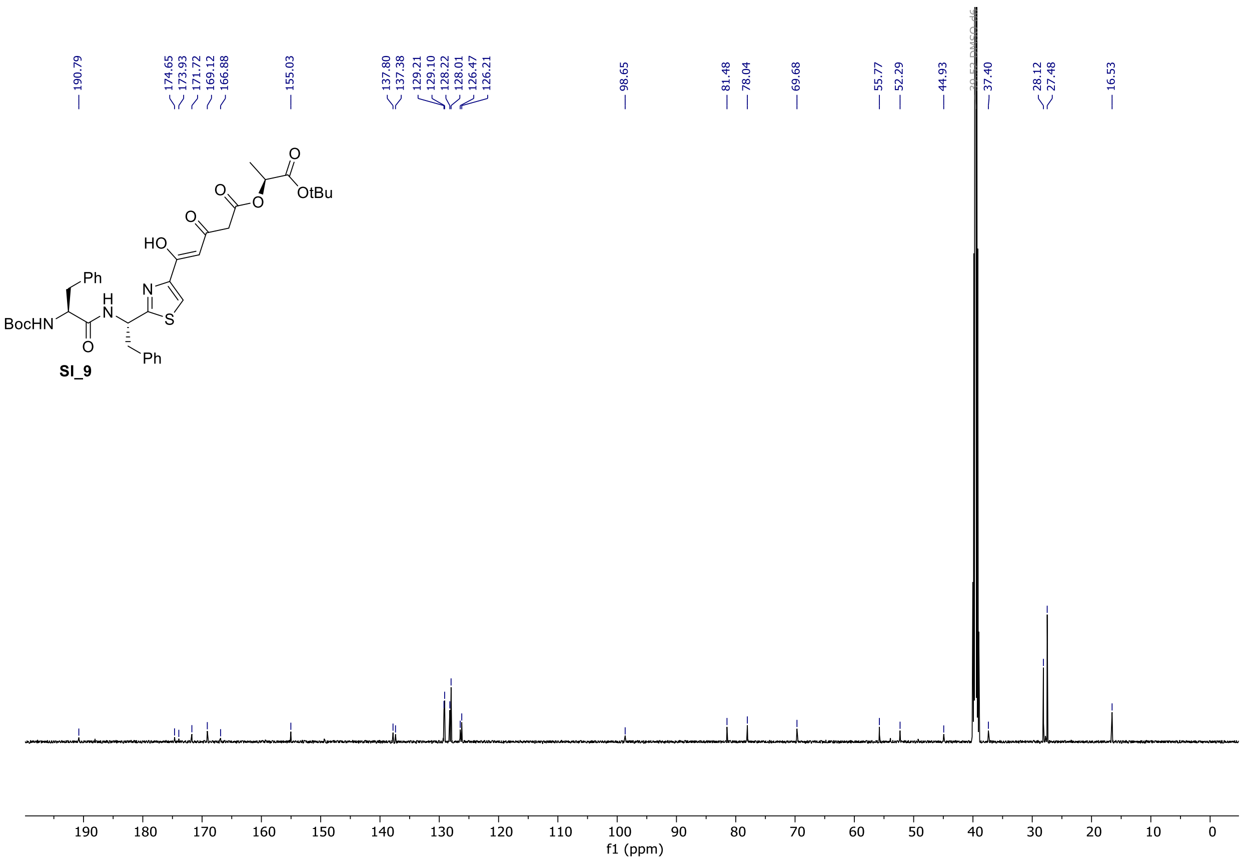

^{1}H NMR (600 MHz, DMSO-d6) of Mutanofactin 697:

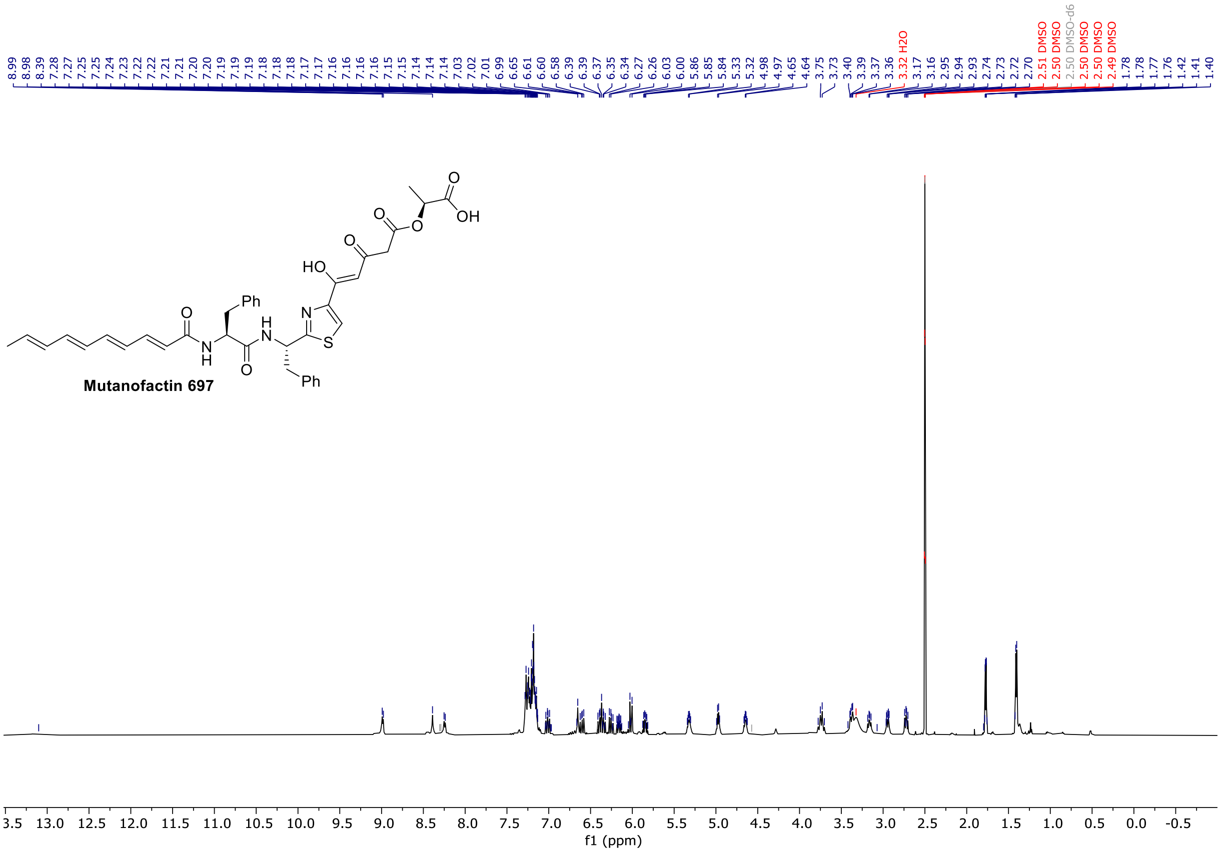

^{13}C NMR (200 MHz, DMSO-d6) of Mutanofactin 697:

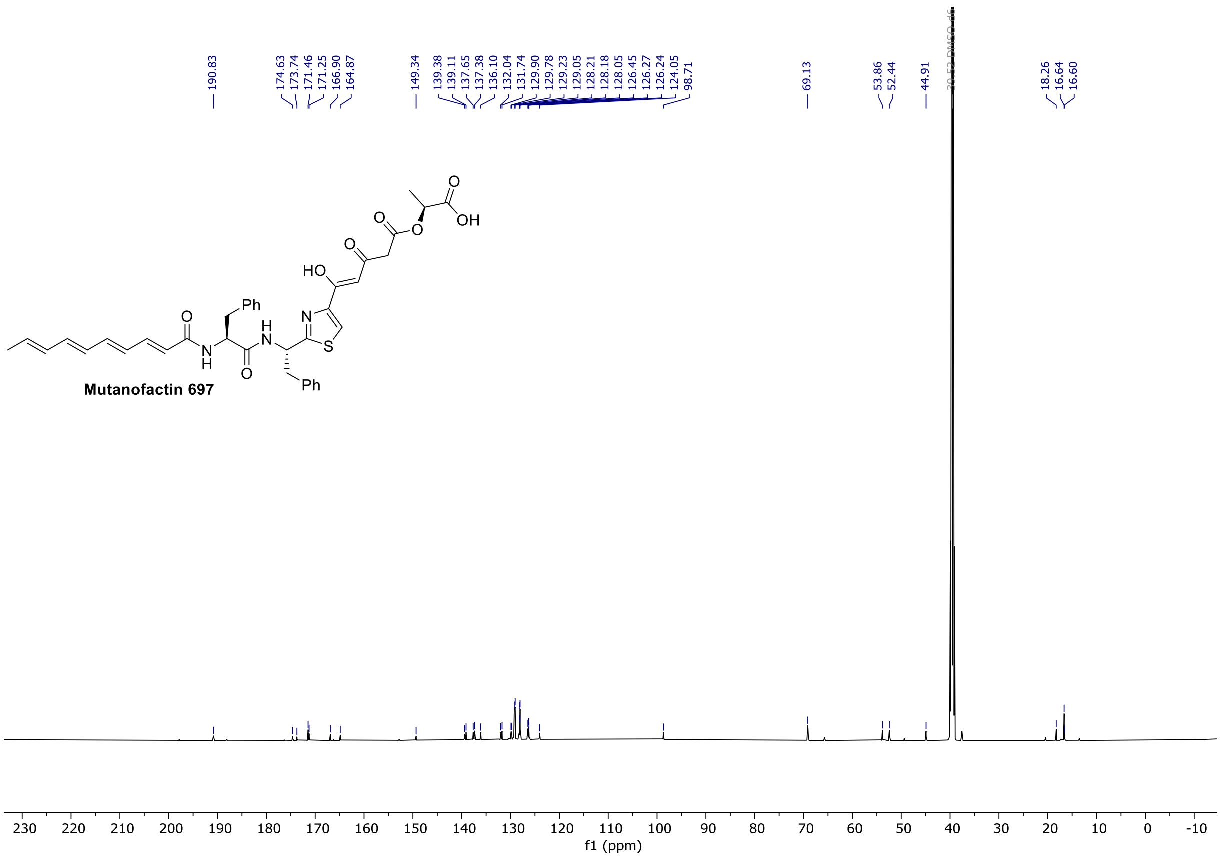

HSQC (DMSO-d6) of Mutanofactin 697:

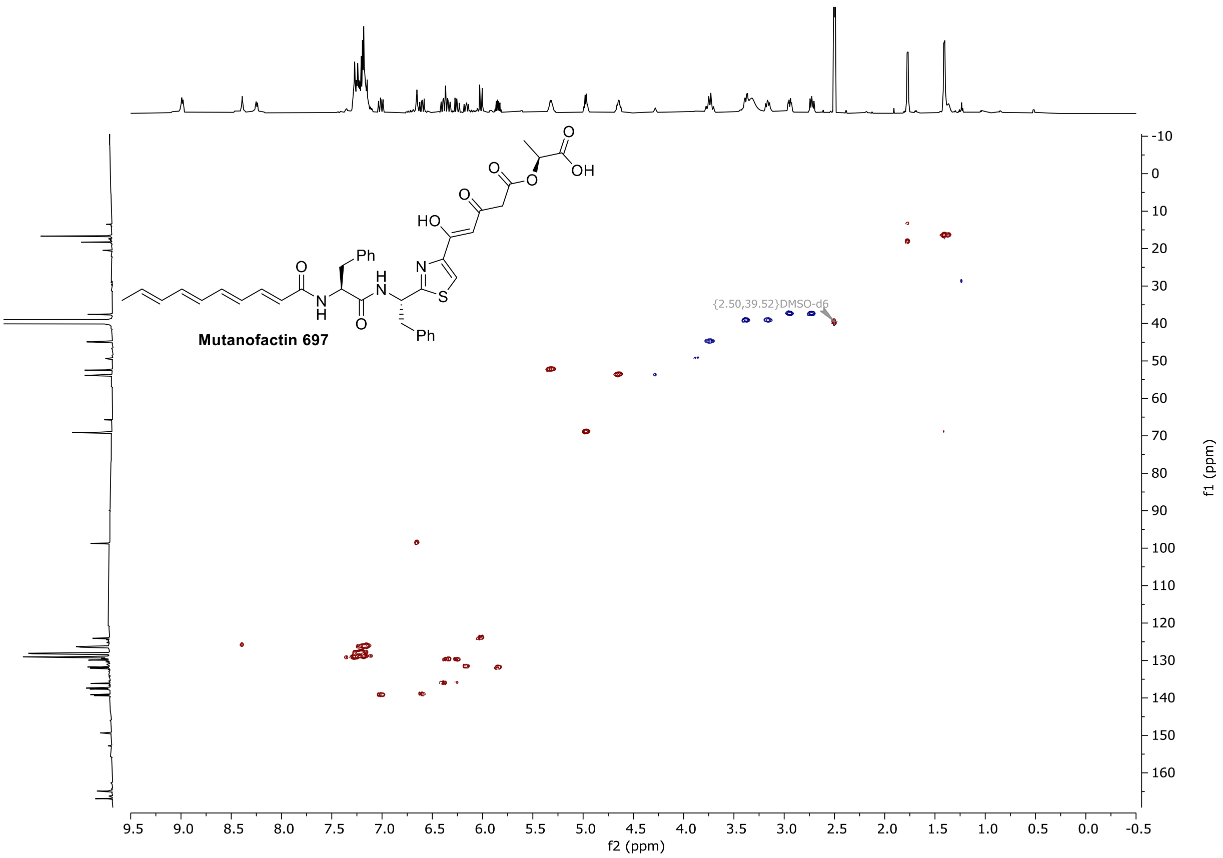

DQFCOSY (DMSO-d6) of Mutanofactin 697:

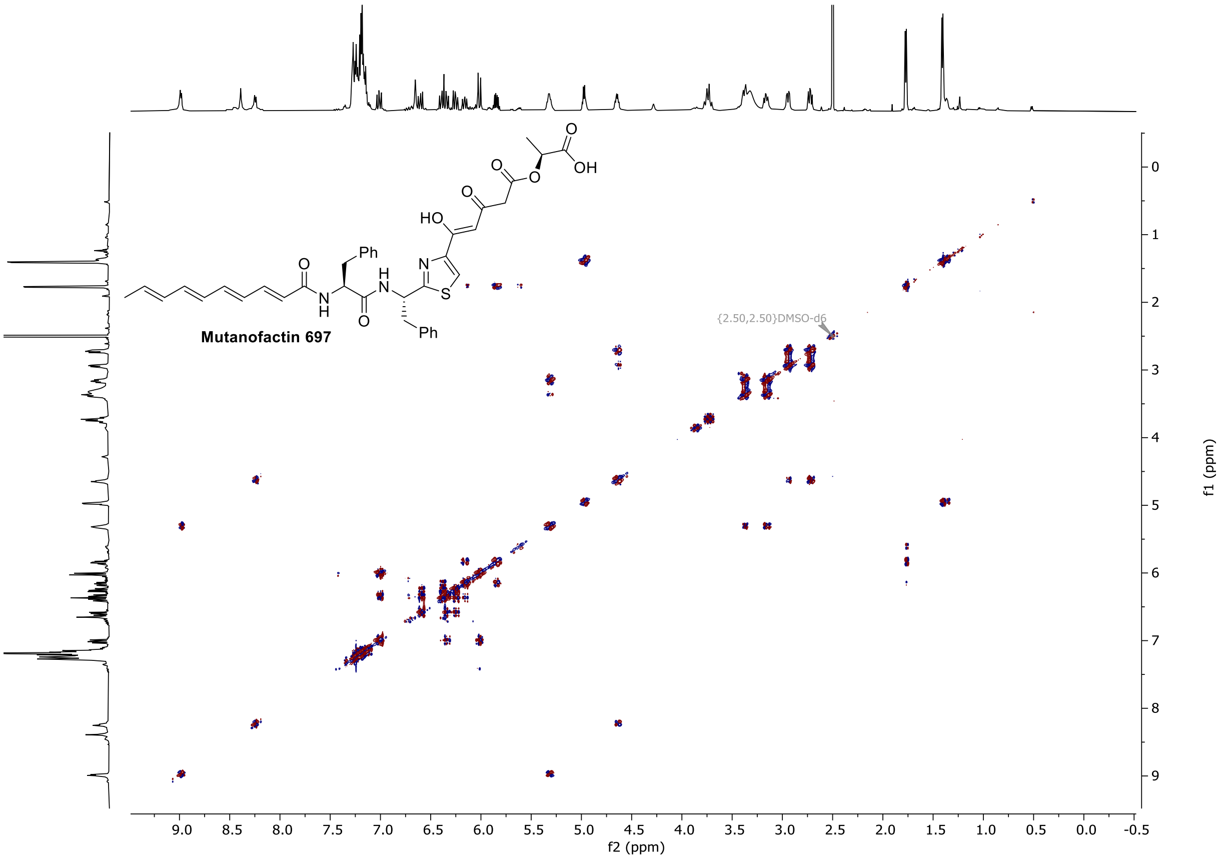

HMBC (DMSO-d6) of Mutanofactin 697:

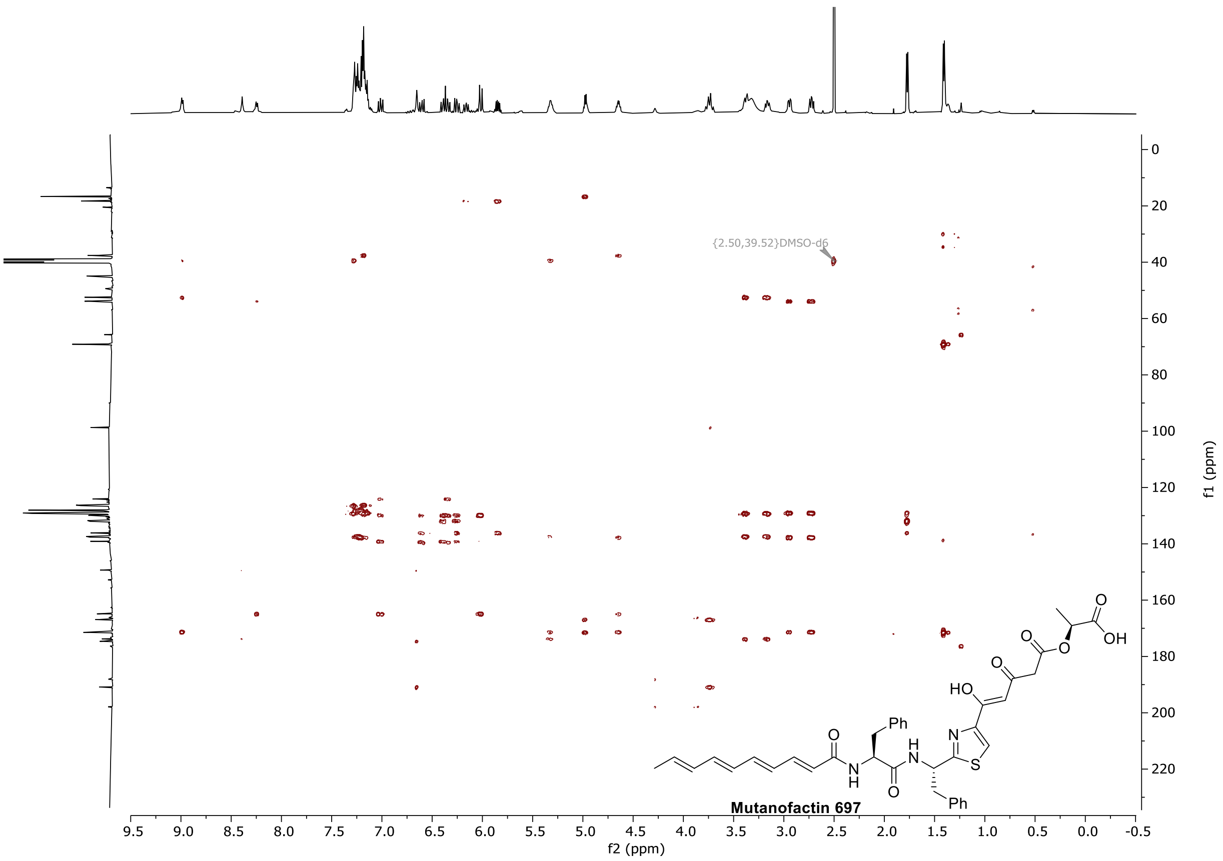

ROESY (DMSO-d6) of Mutanofactin 697:

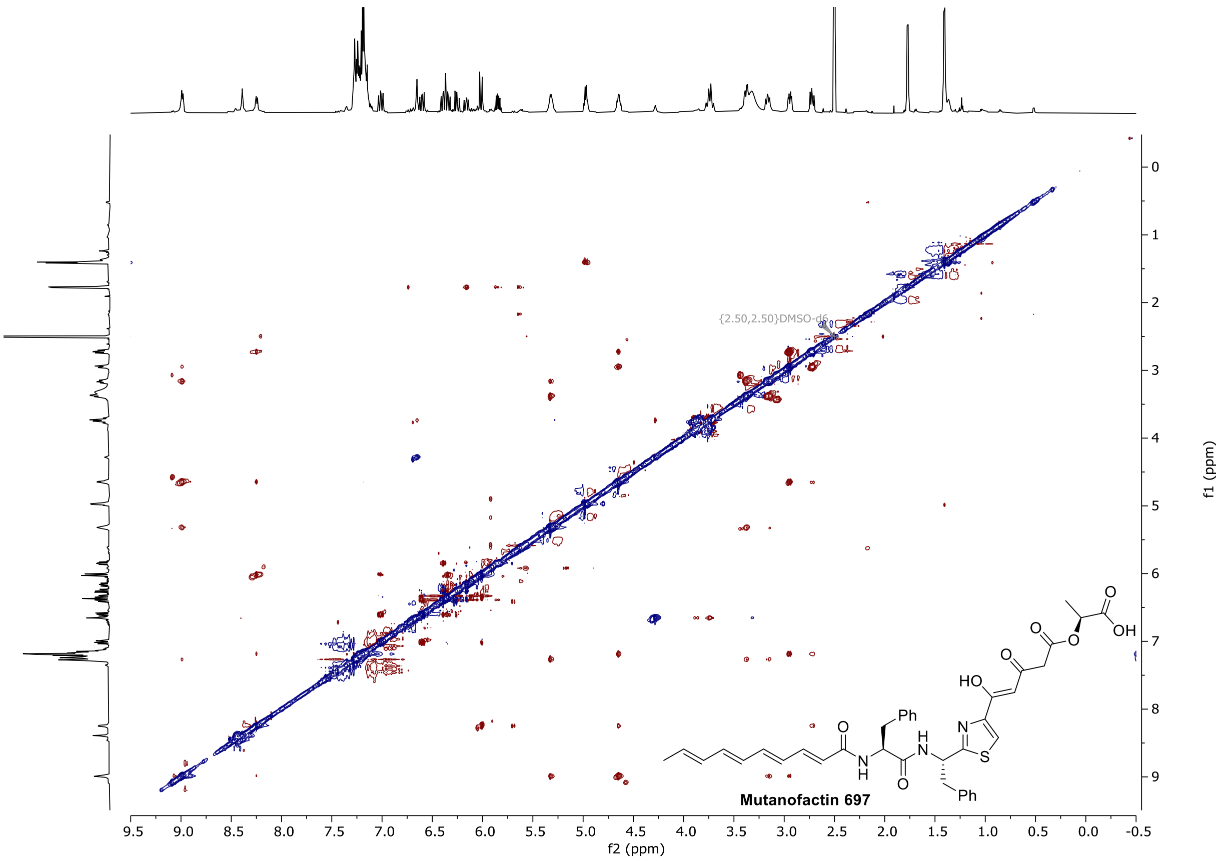

^{1}H NMR (500 MHz, $CDCl_3$) of Alcohol **SI_10**:

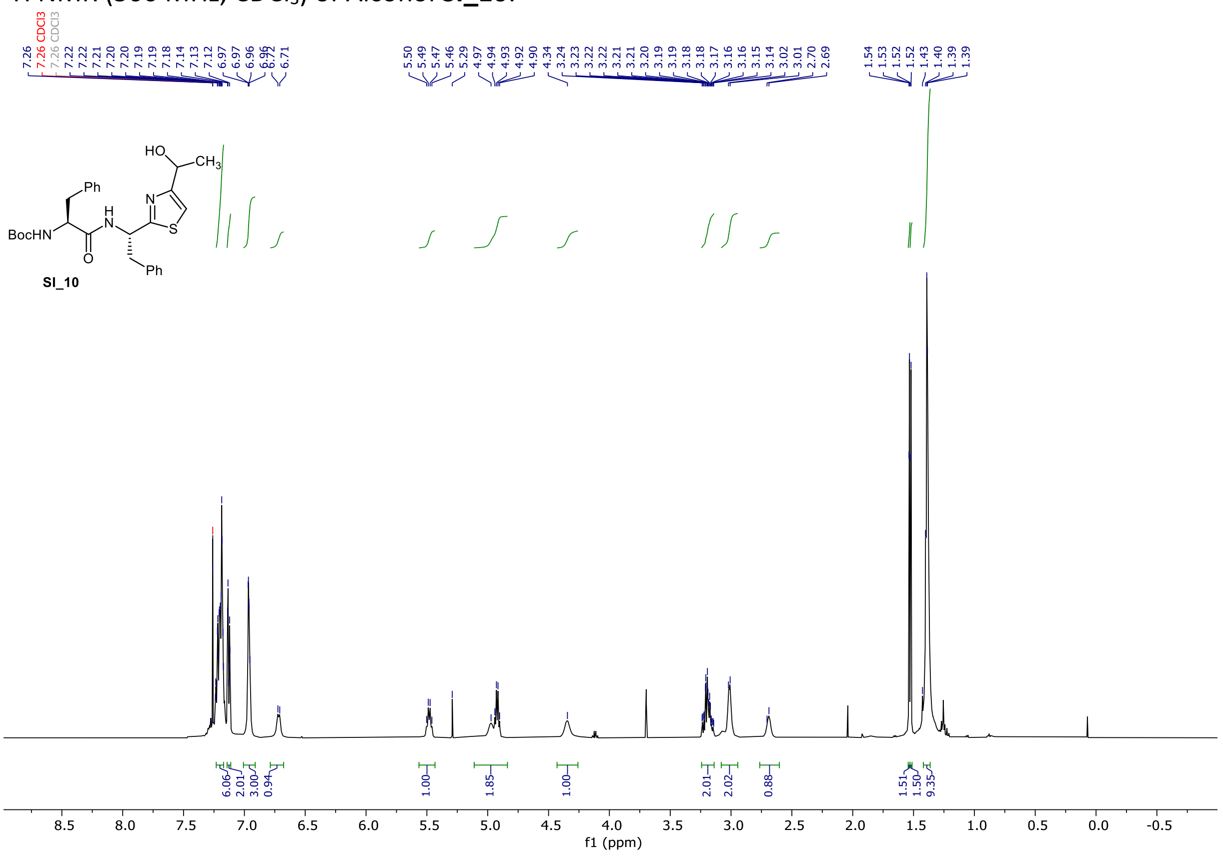

^{13}C NMR (151 MHz, $CDCl_3$) of Alcohol **SI_10**:

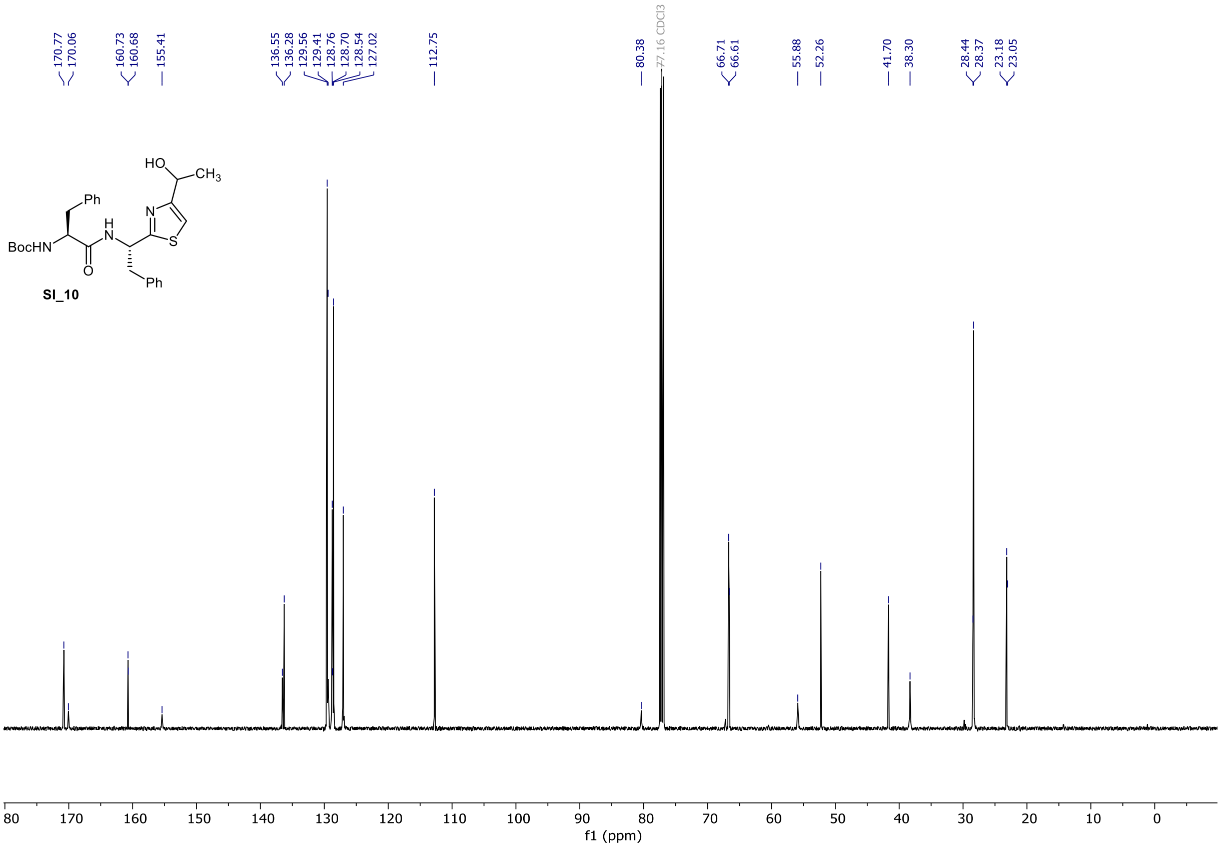

^{1}H NMR (400 MHz, $CDCl_3$) of Methyl ketone **SI_11**:

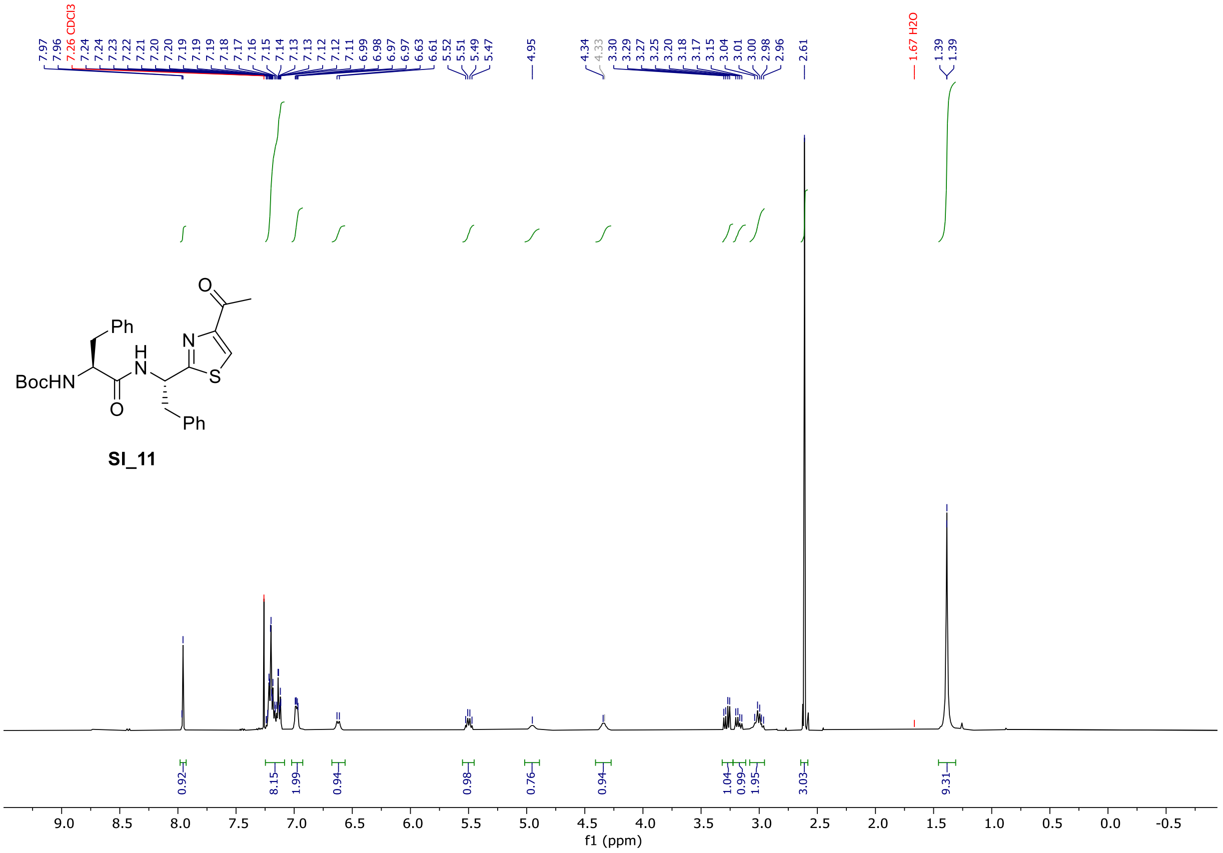

^{13}C NMR (101MHz, $CDCl_3$) of Methyl ketone **SI_11**:

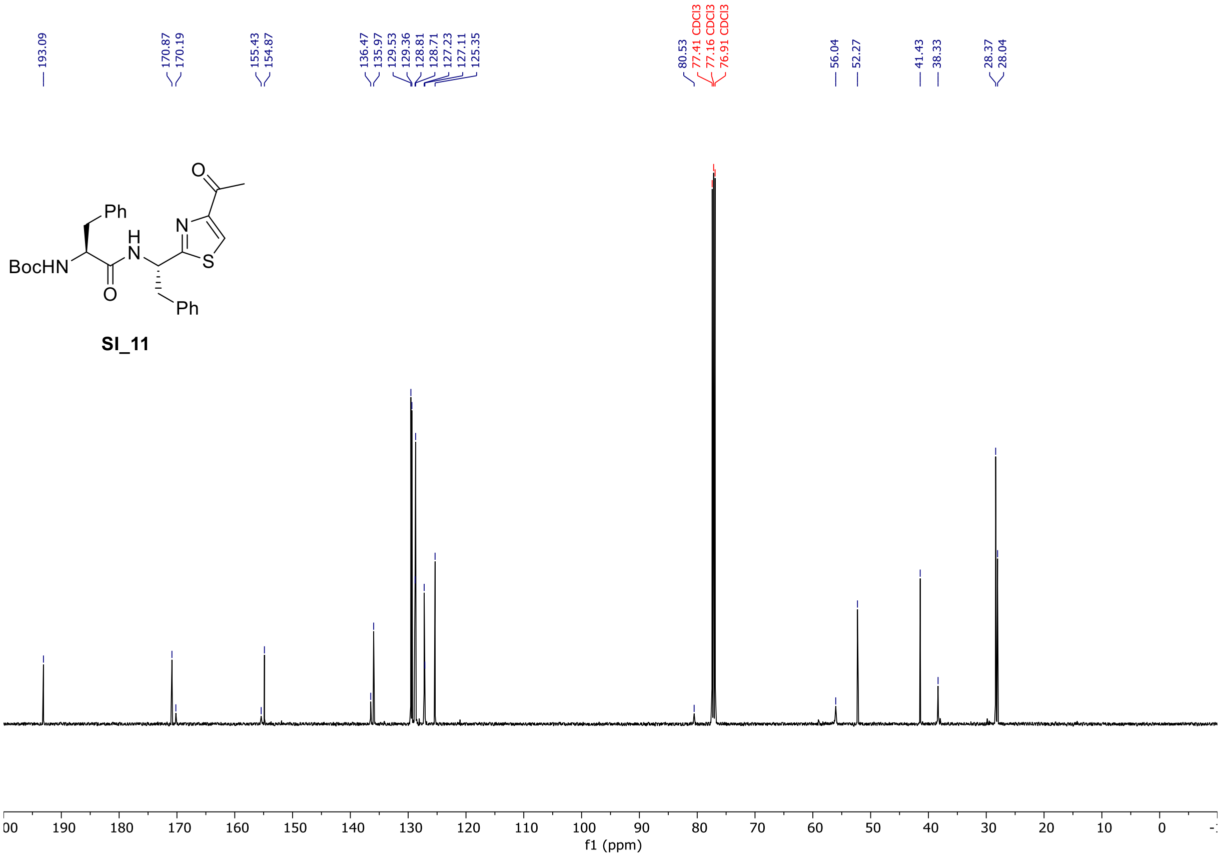

^{1}H NMR (500 MHz, DMSO-d6) of Mutanofactin 539:

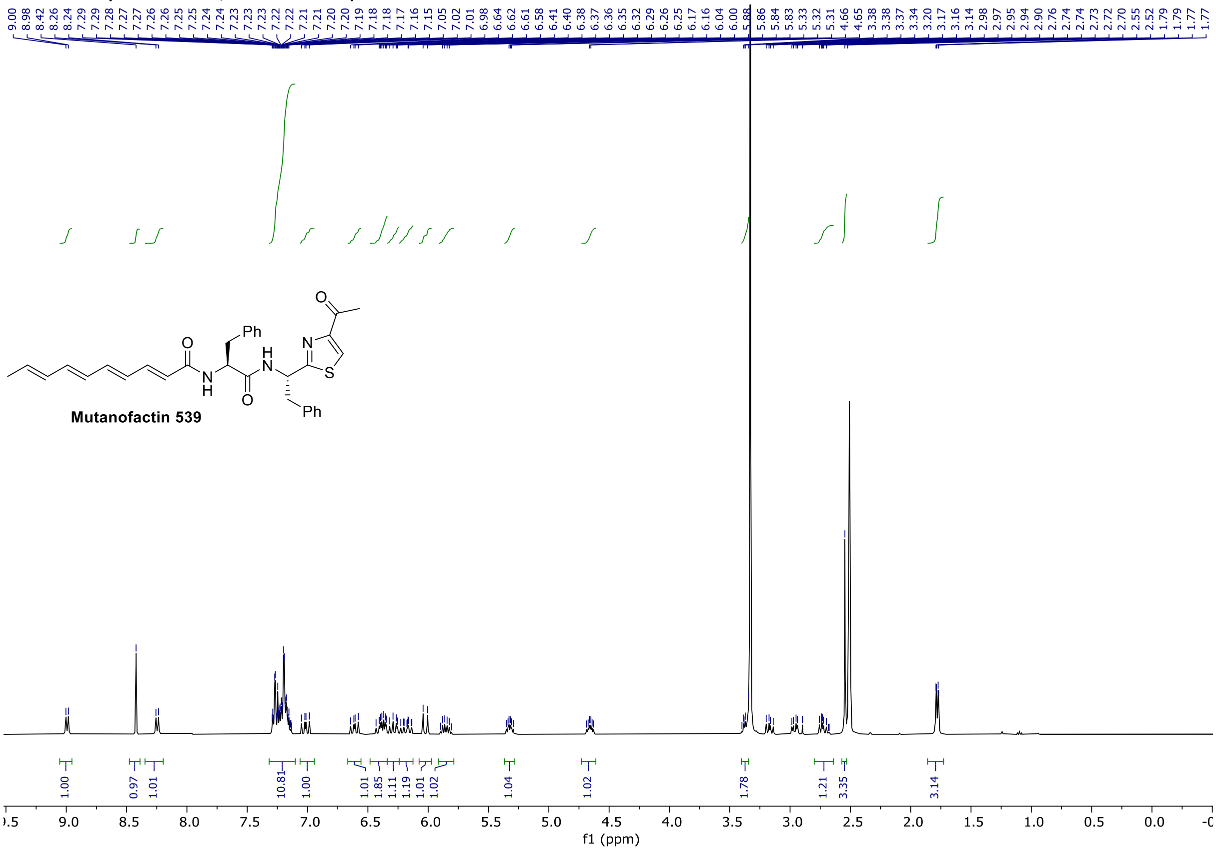

^{13}C NMR (151 MHz, DMSO-d6) of Mutanofactin 539:

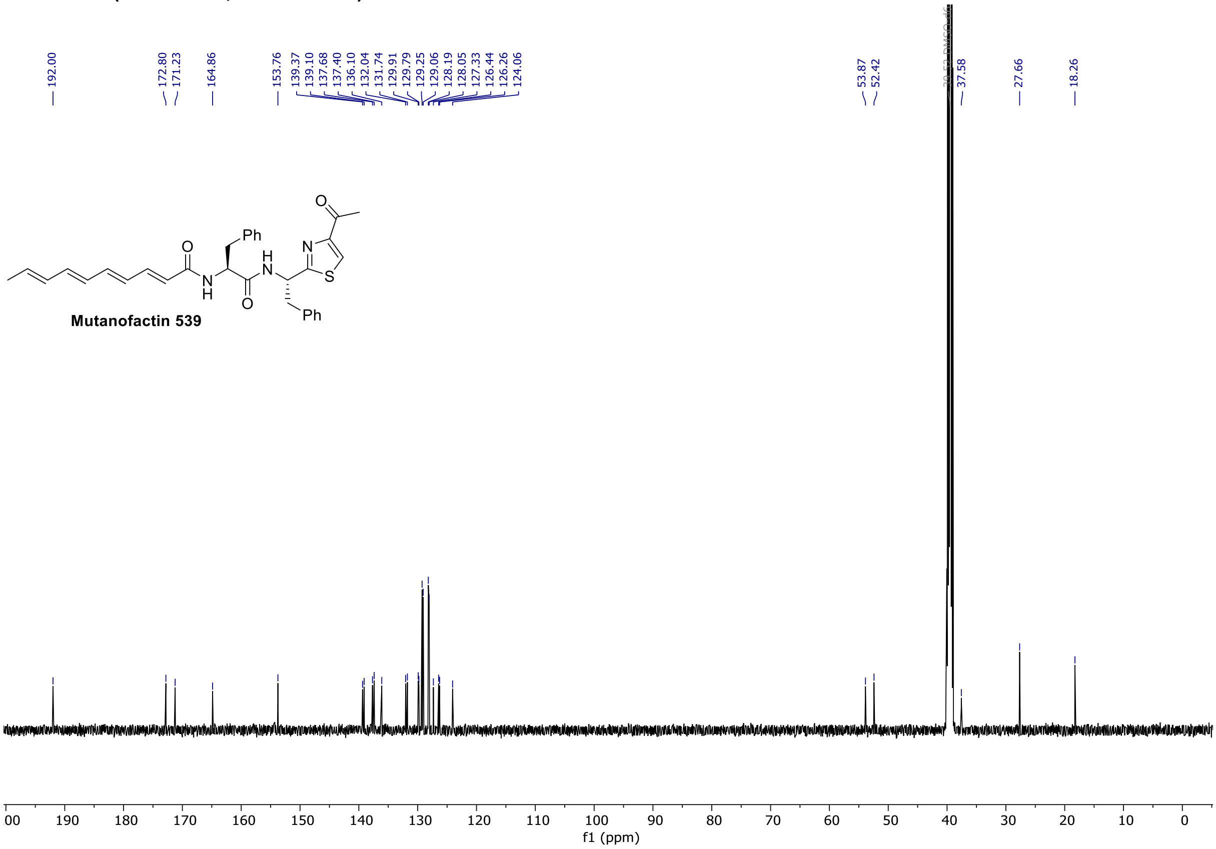

HSQC (DMSO-d6) of Mutanofactin 539:

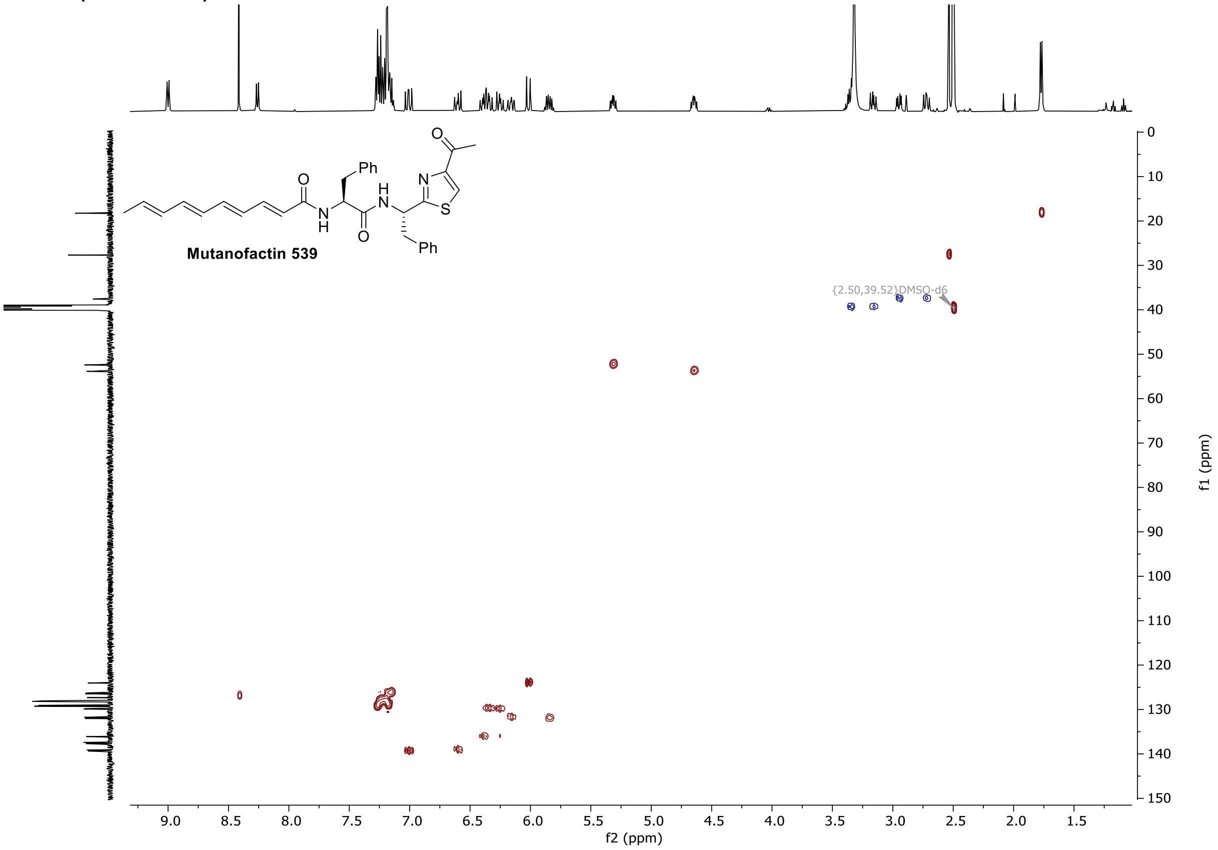

HMBC (DMSO-d6) of Mutanofactin 539:

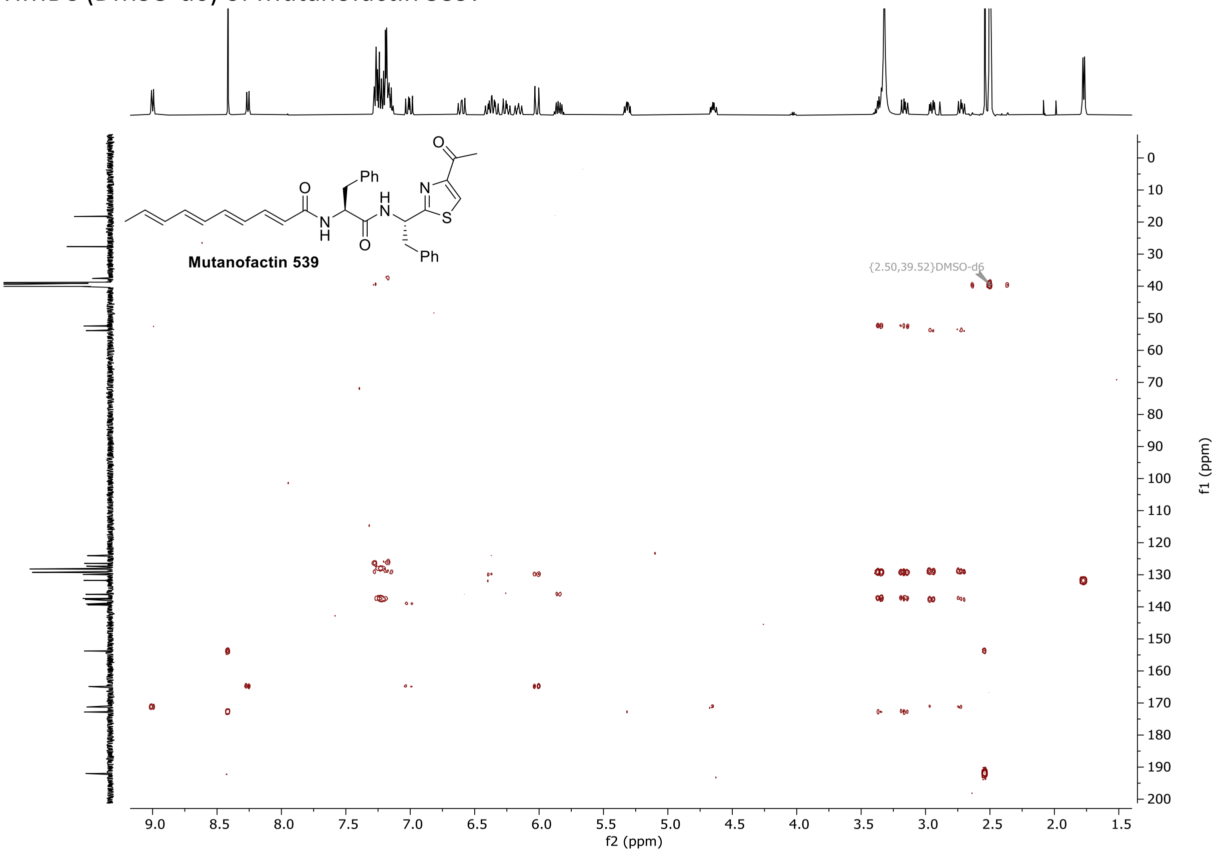

COSY (DMSO-d6) of Mutanofactin 539:

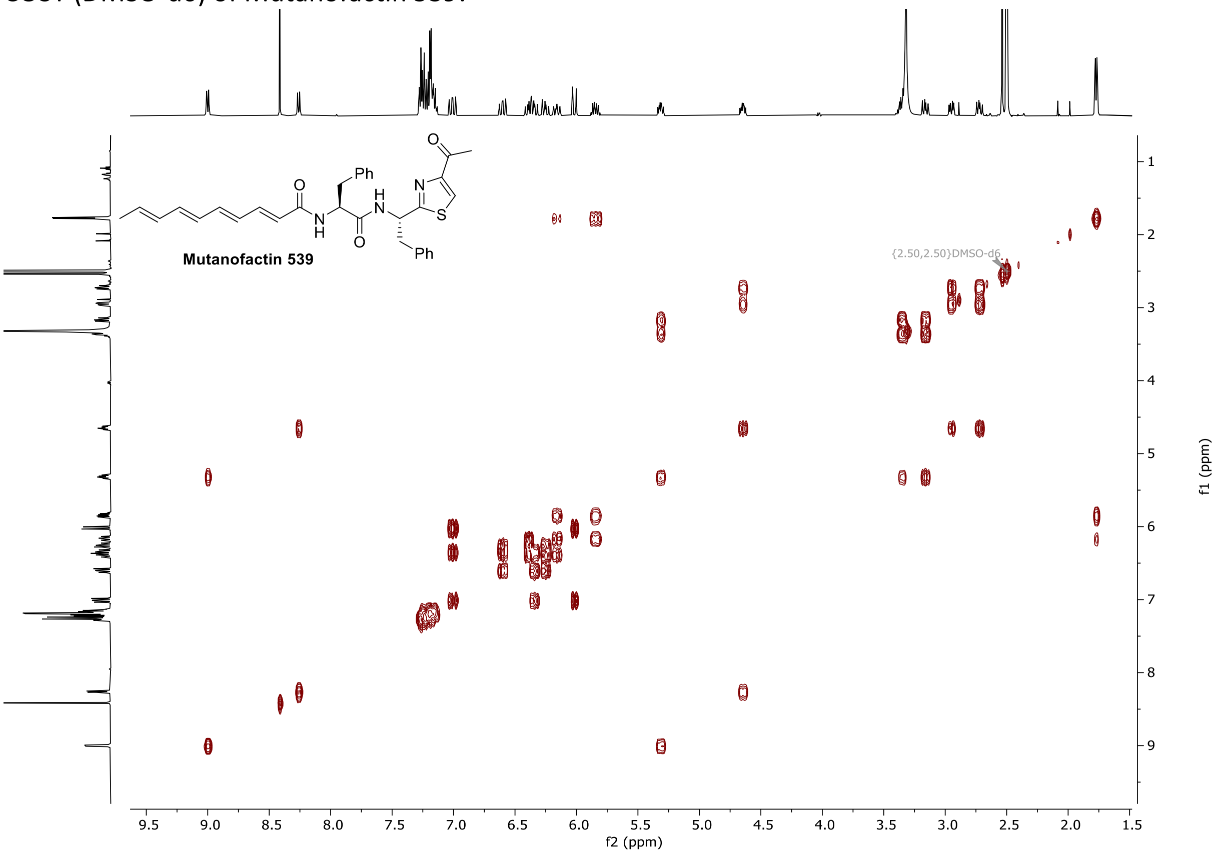

^{1}H NMR (400 MHz, MeOD) of Acid **SI_12**:

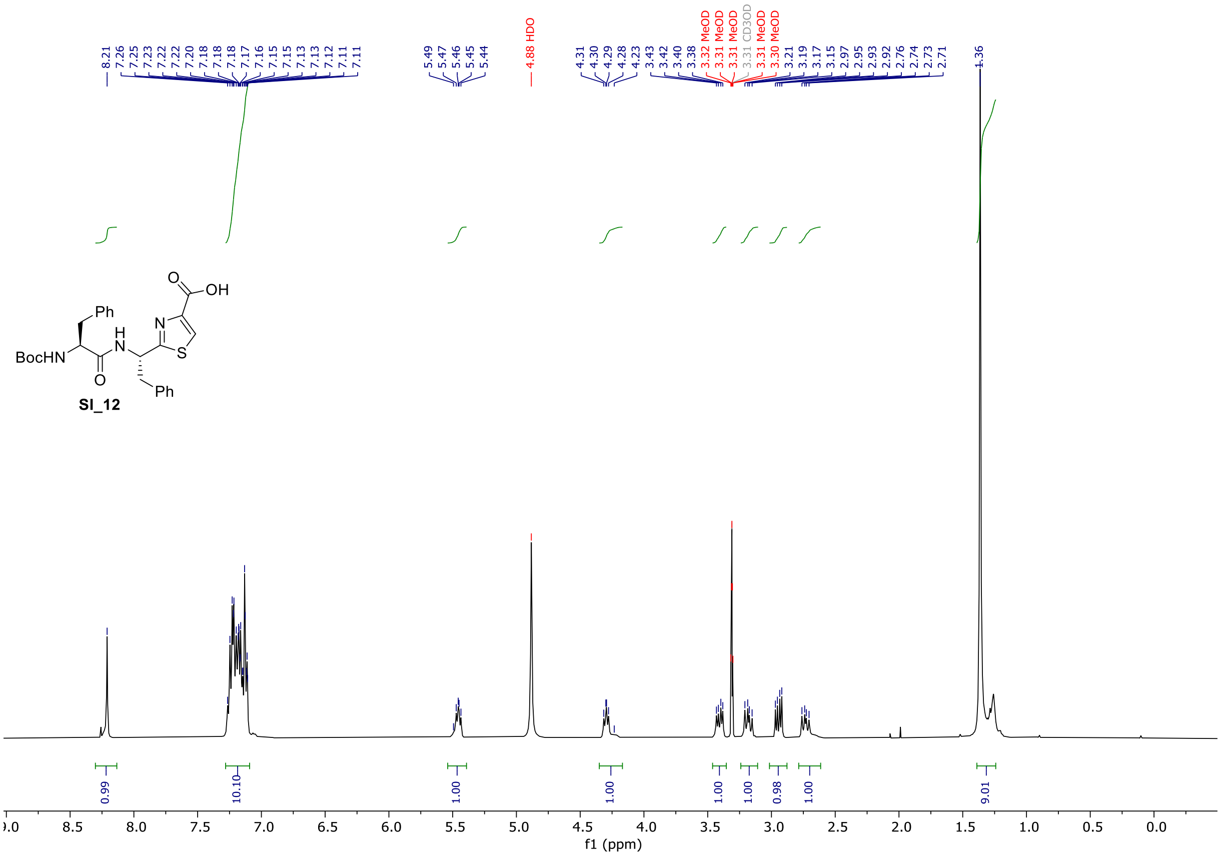

^{1}H NMR (101 MHz, MeOD) of Acid **SI_12**:

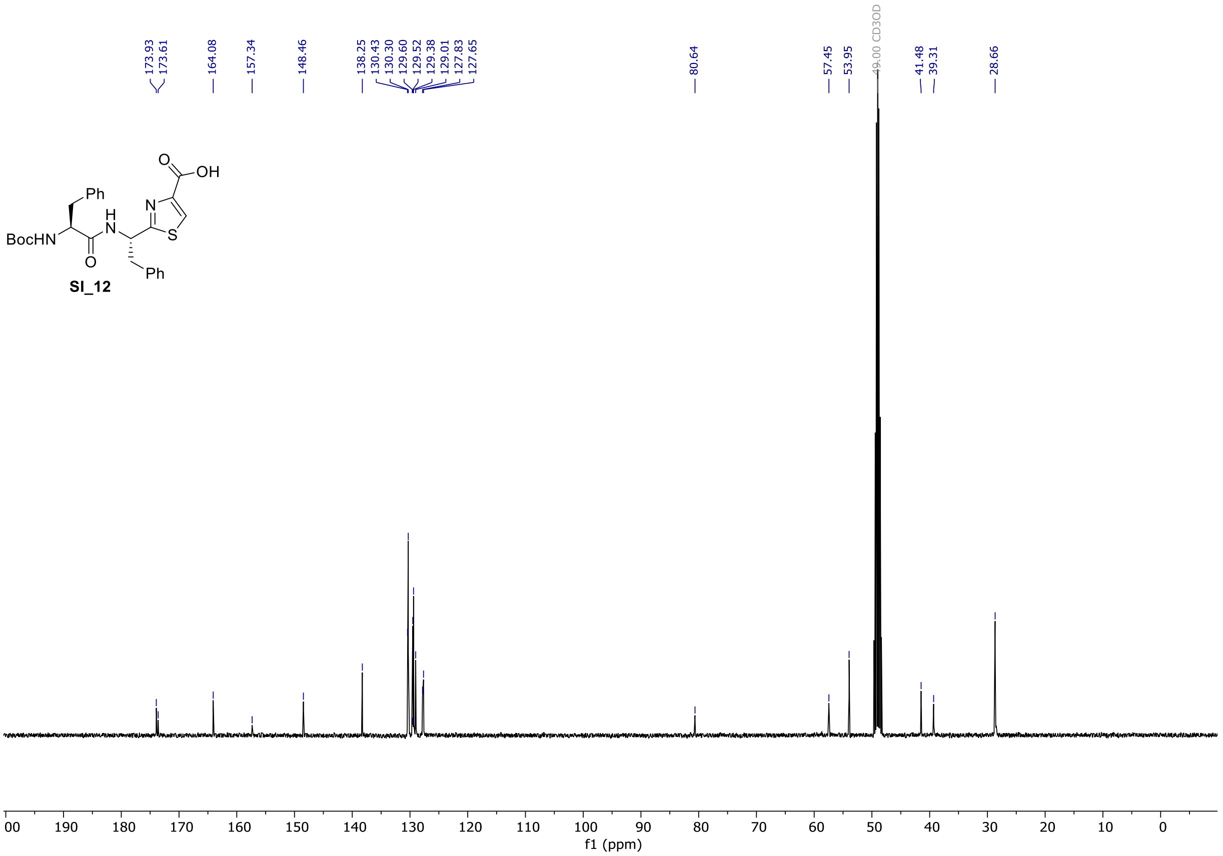

Note: Some Boc-Induced Rotamers are present.

^{1}H NMR (400 MHz, DMSO-d6) of Mutanofactin 541:

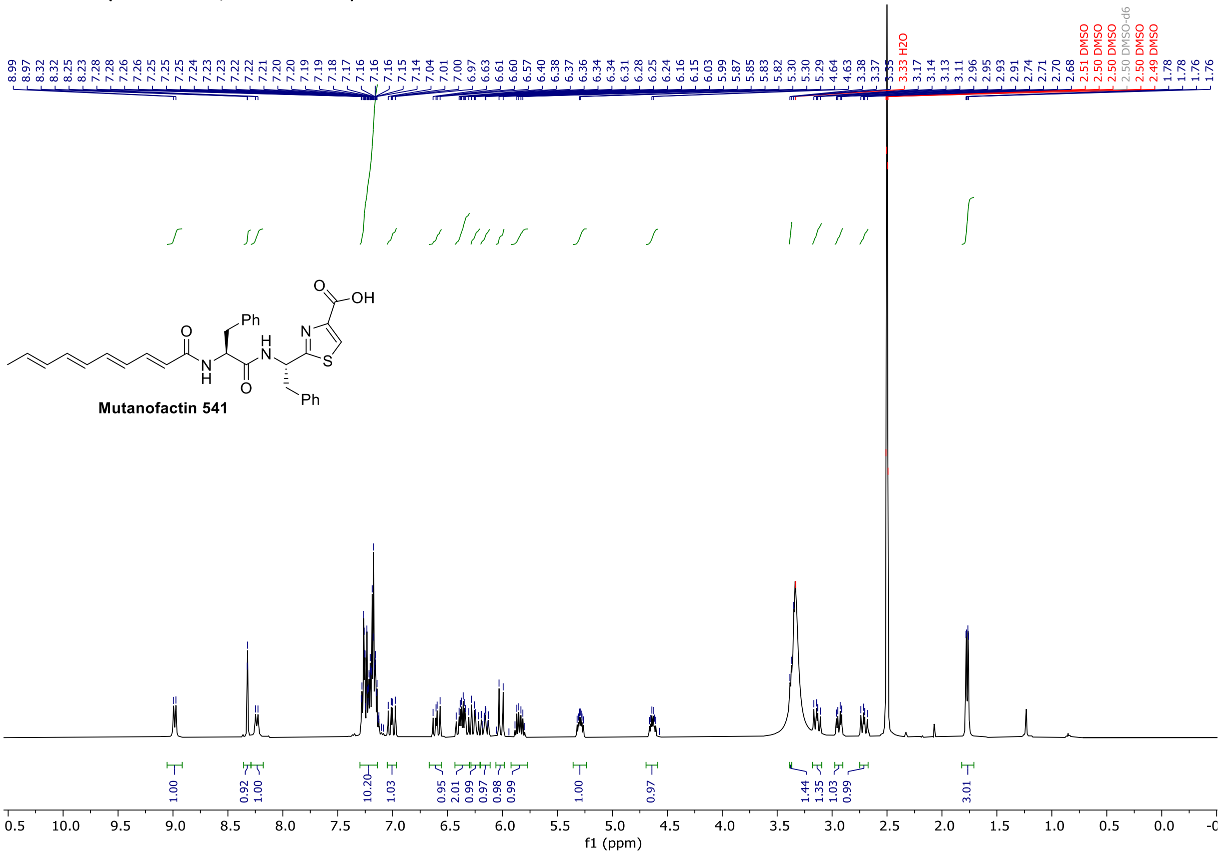

^{13}C NMR (101 MHz, DMSO-d6) of Mutanofactin 541:

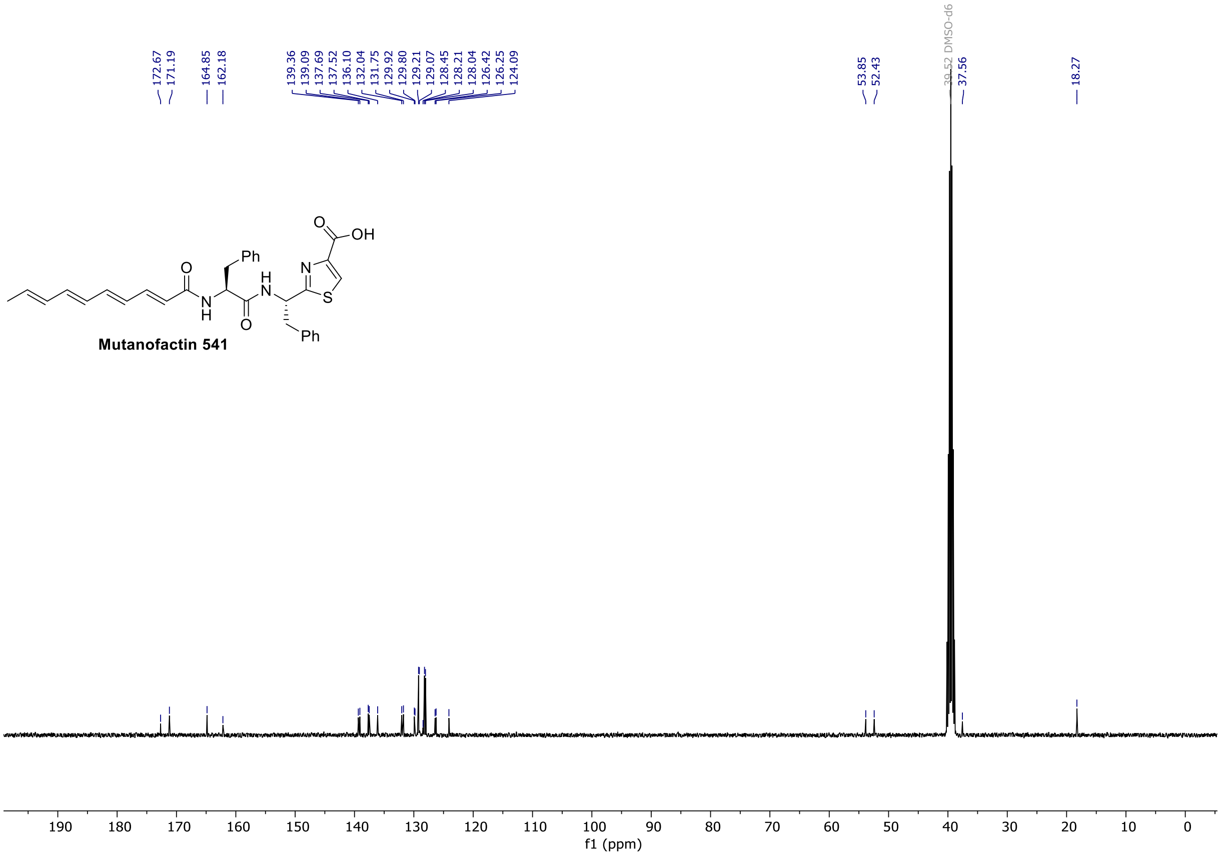

HSQC (DMSO-d6) of Mutanofactin 541:

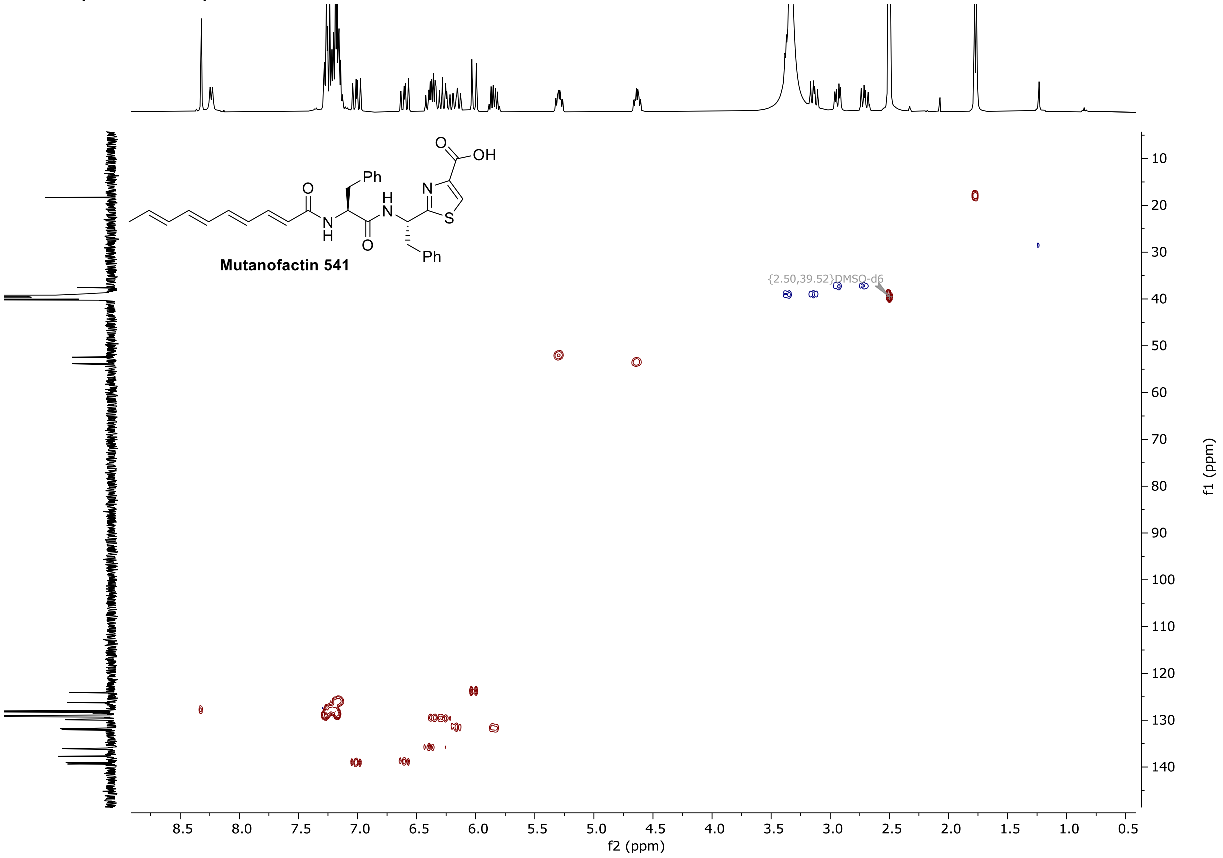

COSY (DMSO-d6) of Mutanofactin 541:

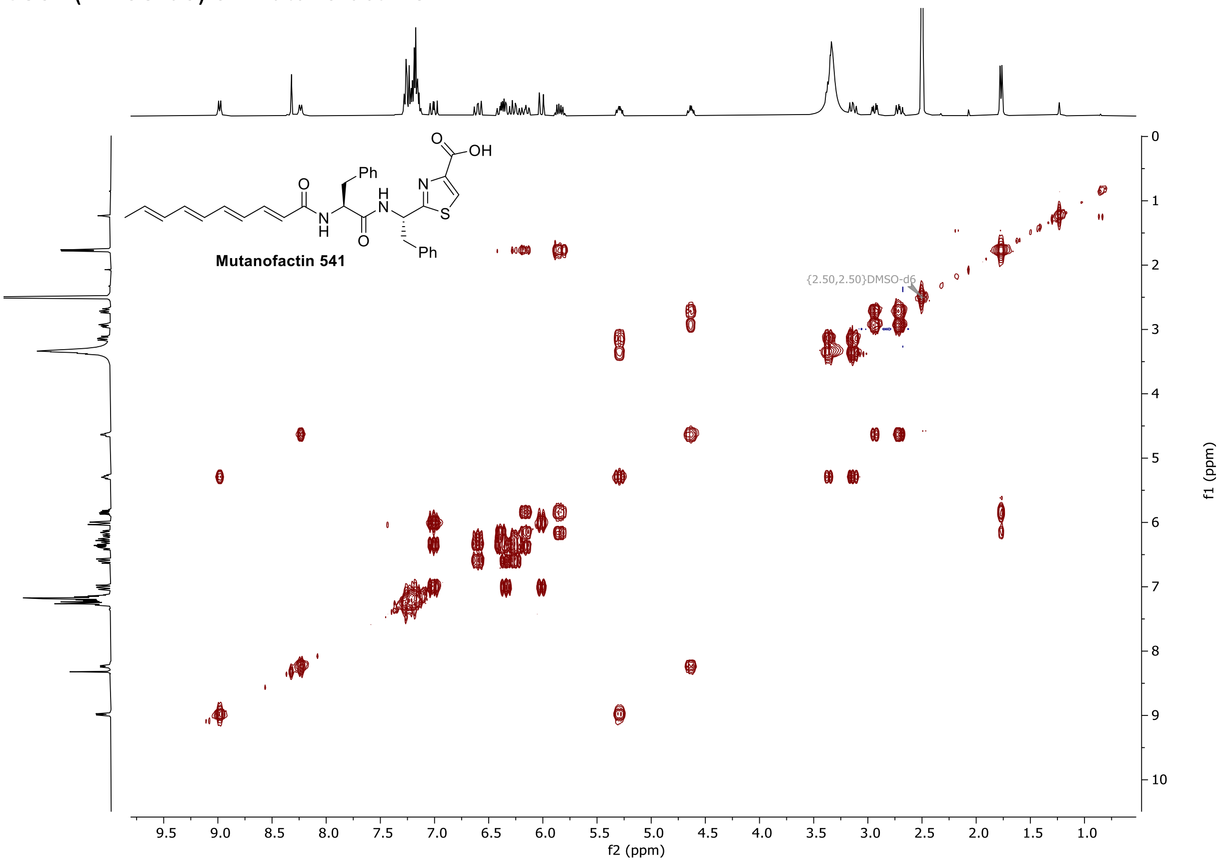

HMBC (DMSO-d6) of Mutanofactin 541:

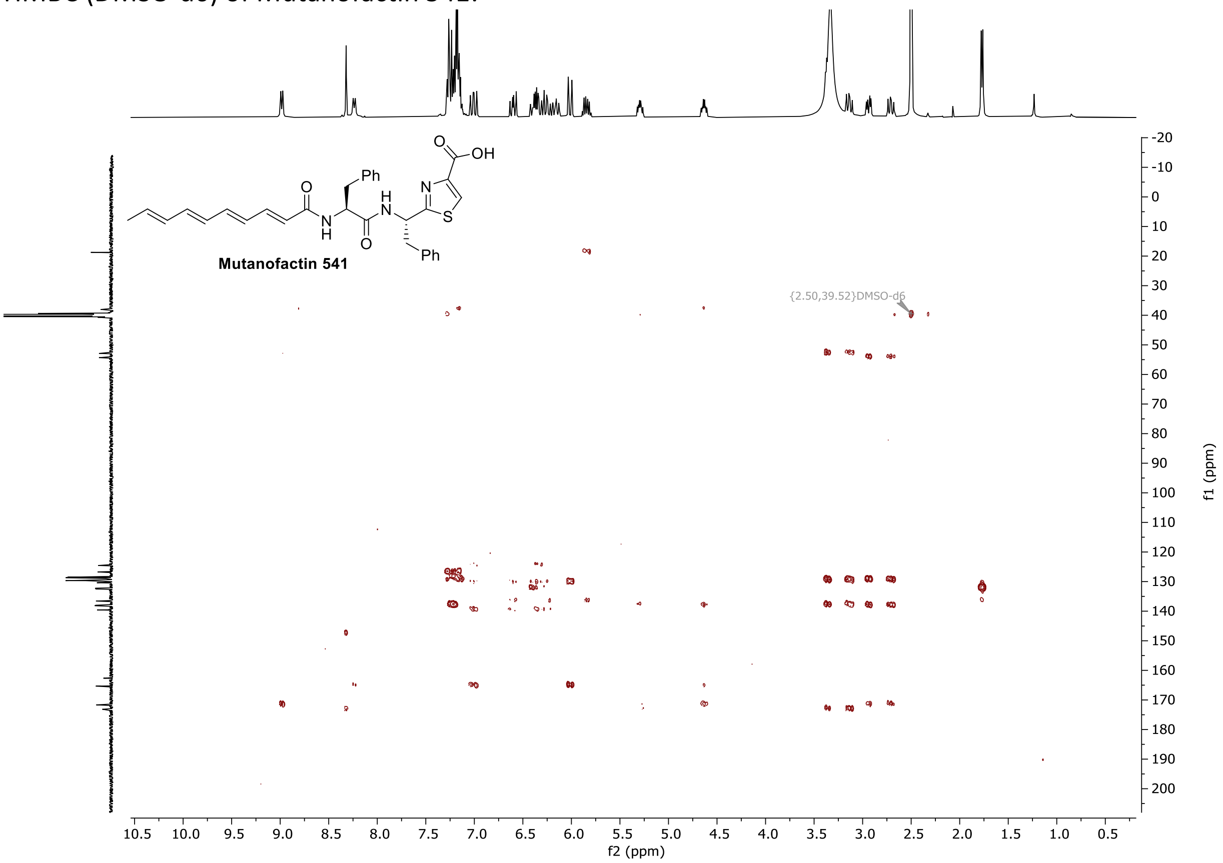

^{1}H NMR (400 MHz, DMSO-d6) of Dipeptide **17**:

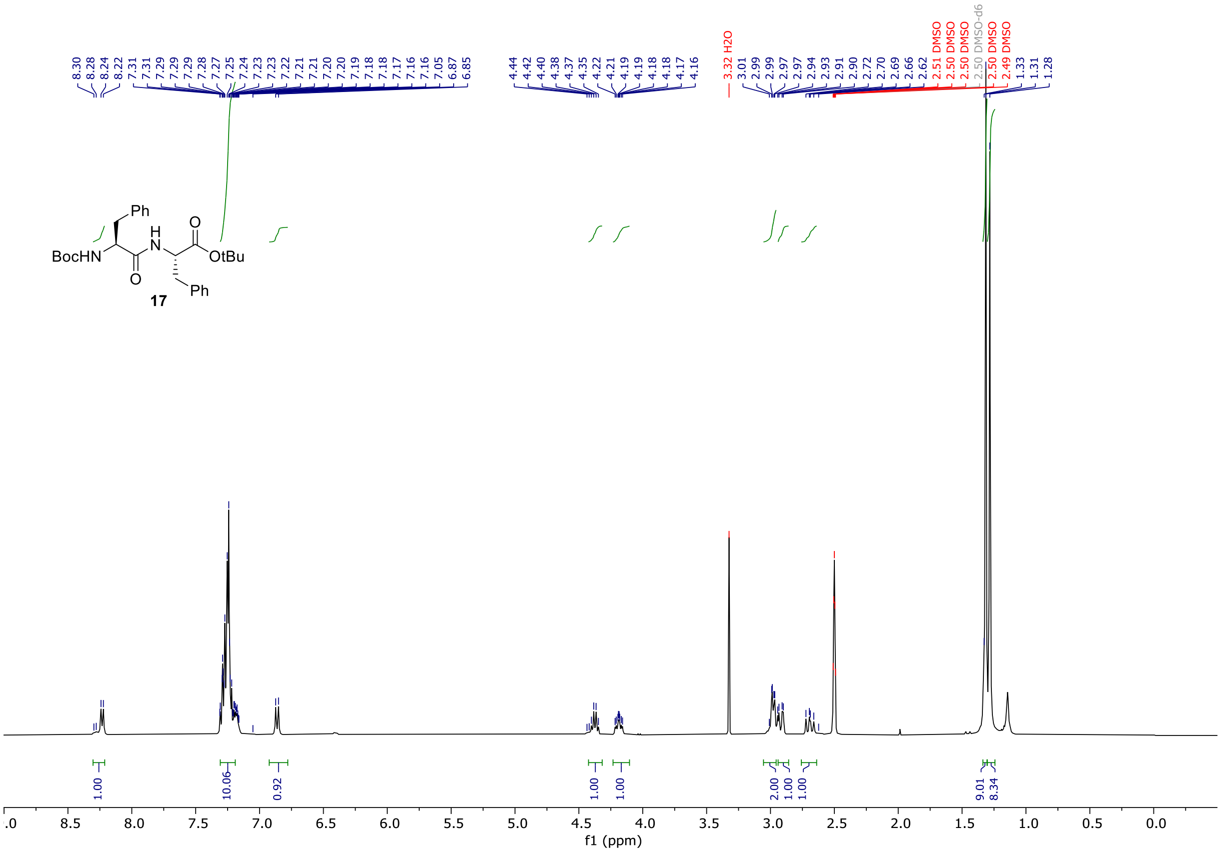

^{13}C NMR (101 MHz, DMSO-d6) of Dipeptide **17**:

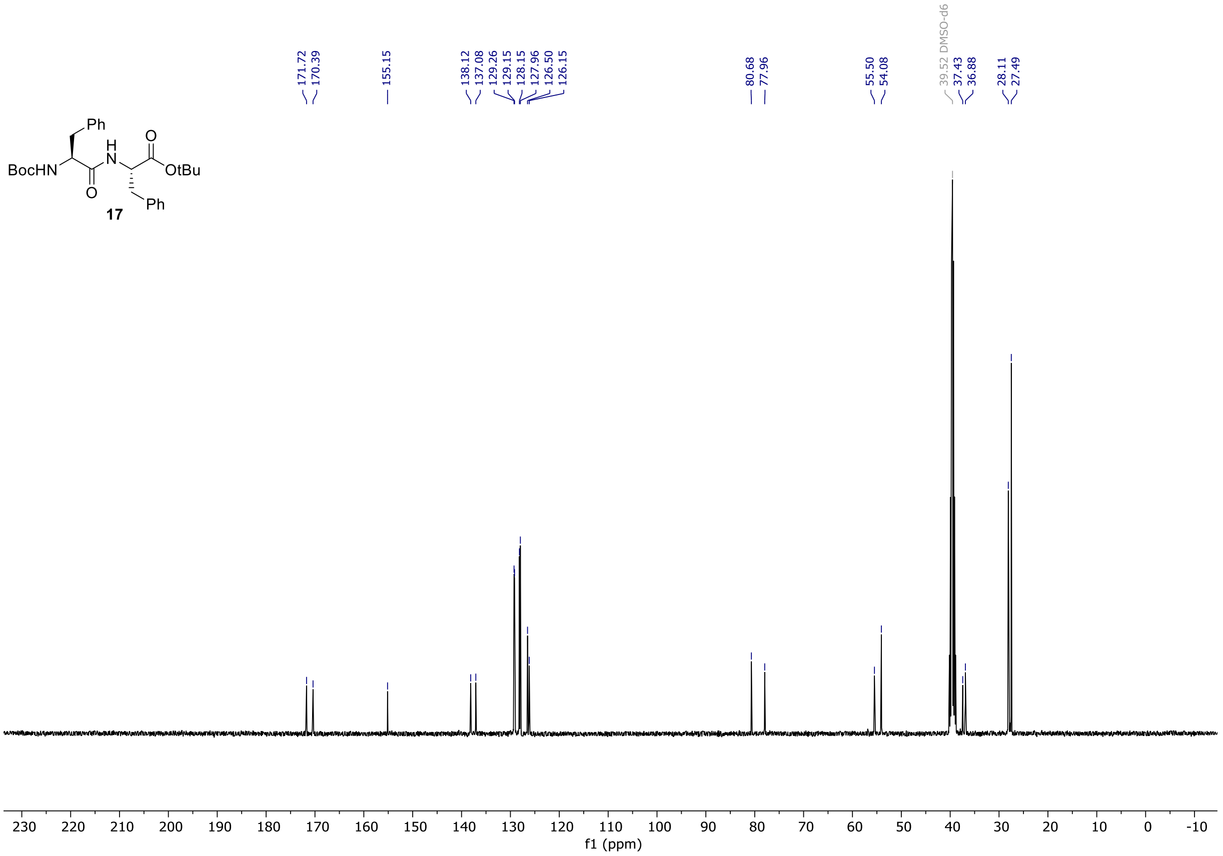

^{1}H NMR (500 MHz, DMSO-d6) of Mutanofactin 458:

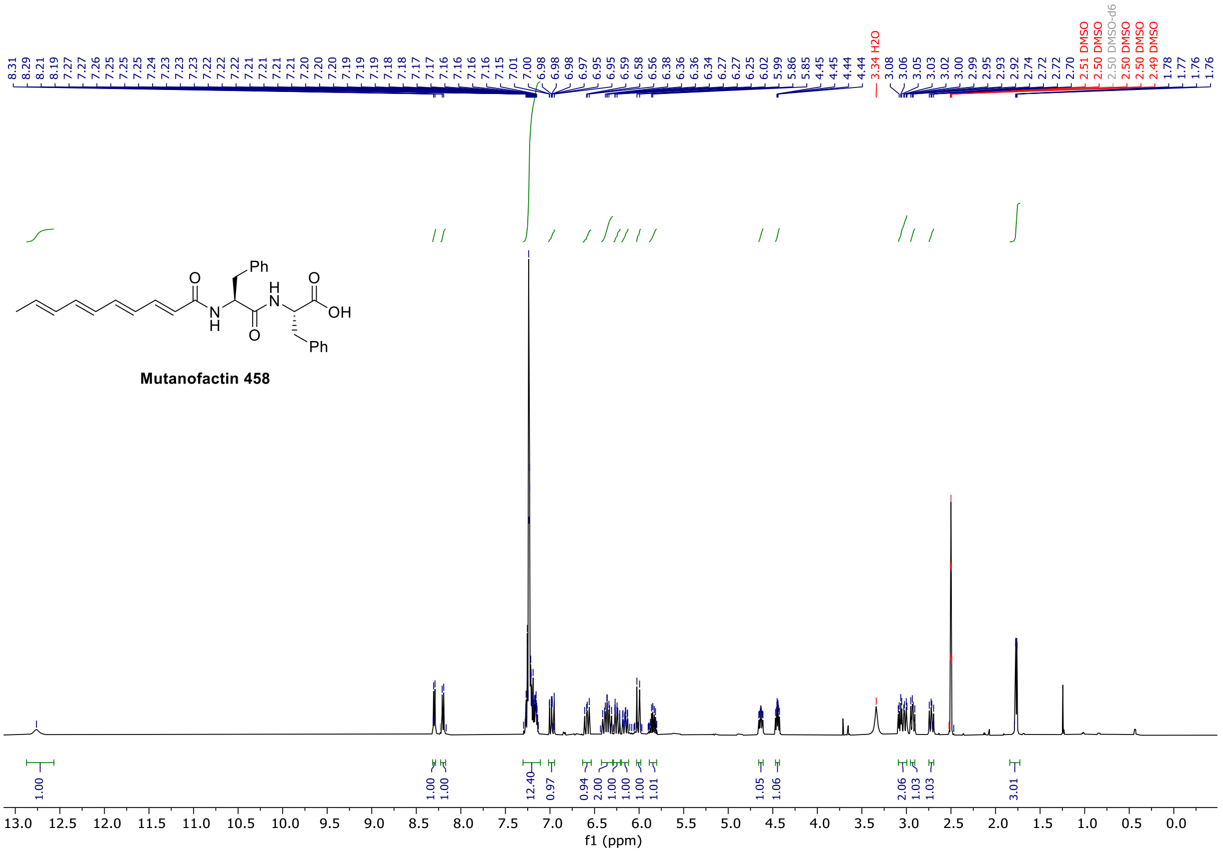

^{13}C NMR (151MHz, DMSO-d6) of Mutanofactin 458:

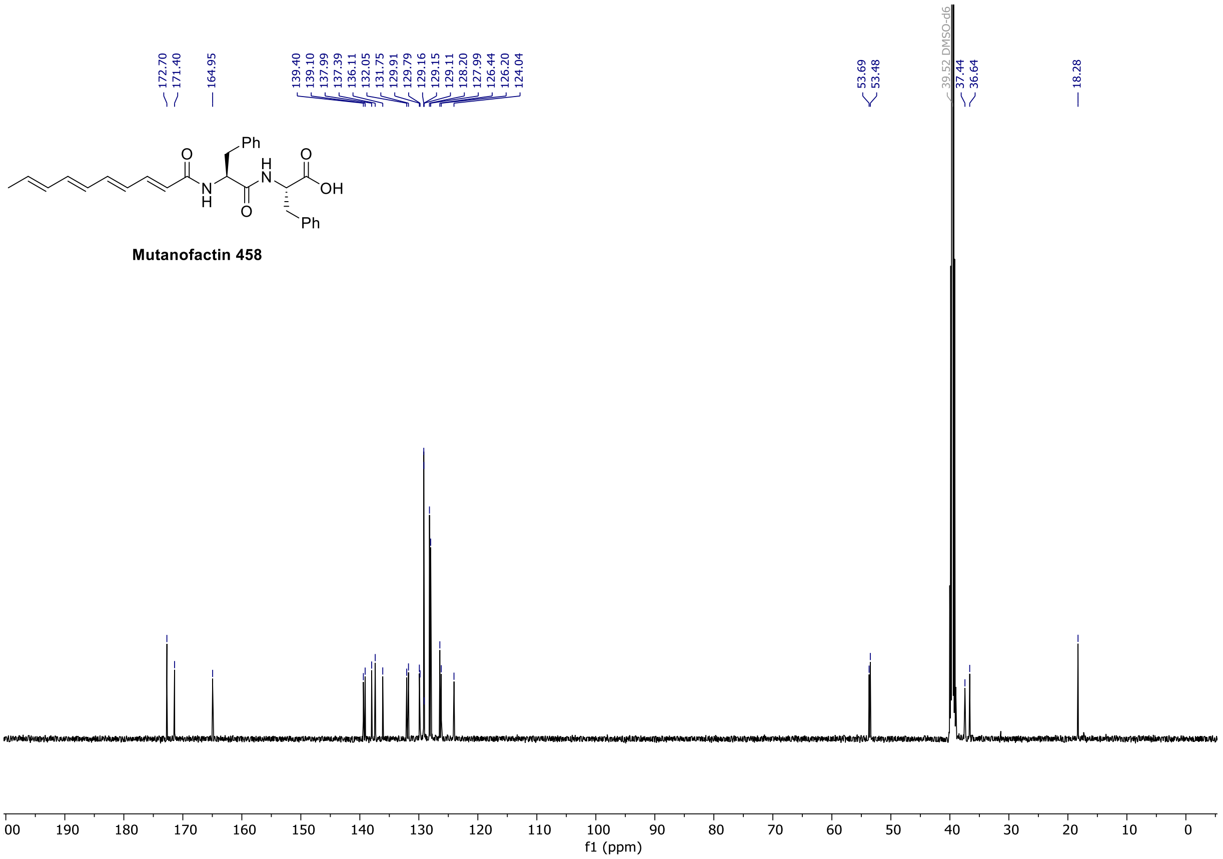

HSQC (DMSO-d6) of Mutanofactin 458:

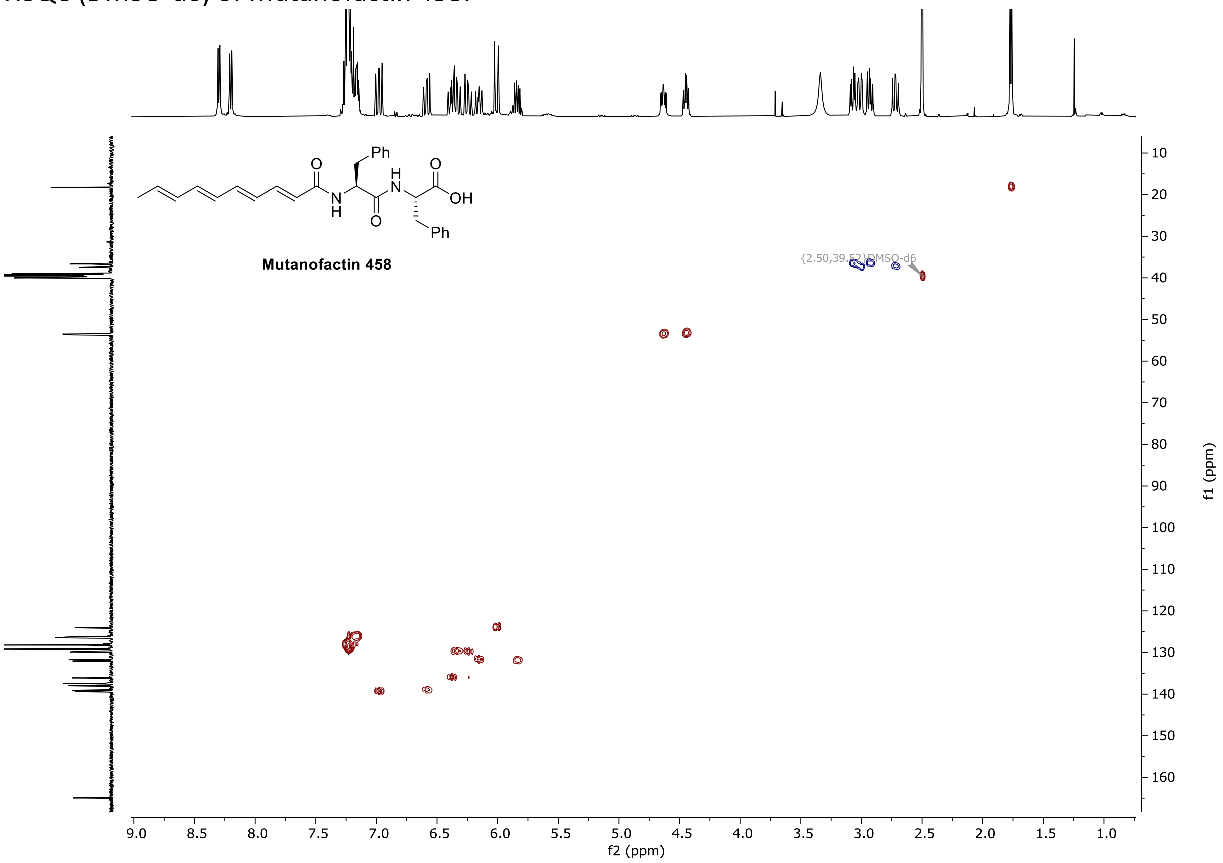

HMBC (DMSO-d6) of Mutanofactin 458:

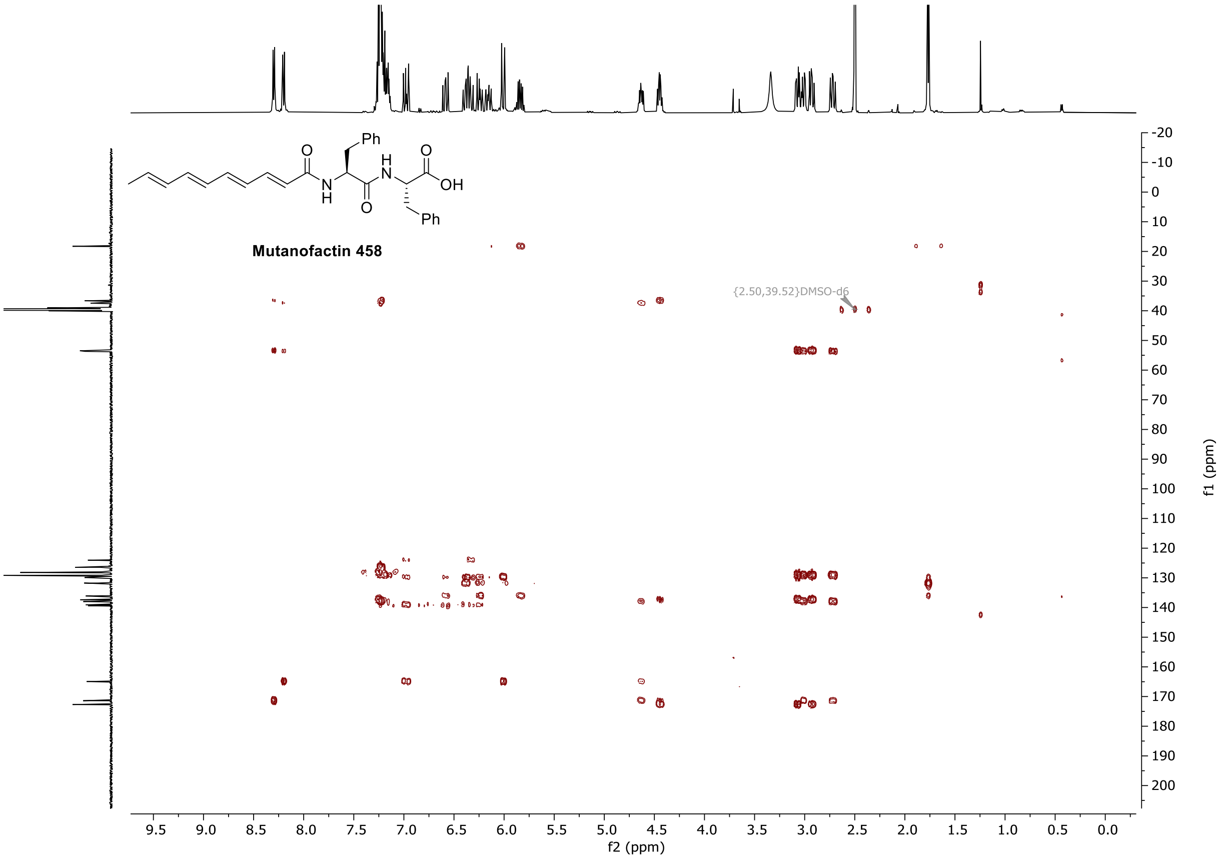

COSY (DMSO-d6) of Mutanofactin 458:

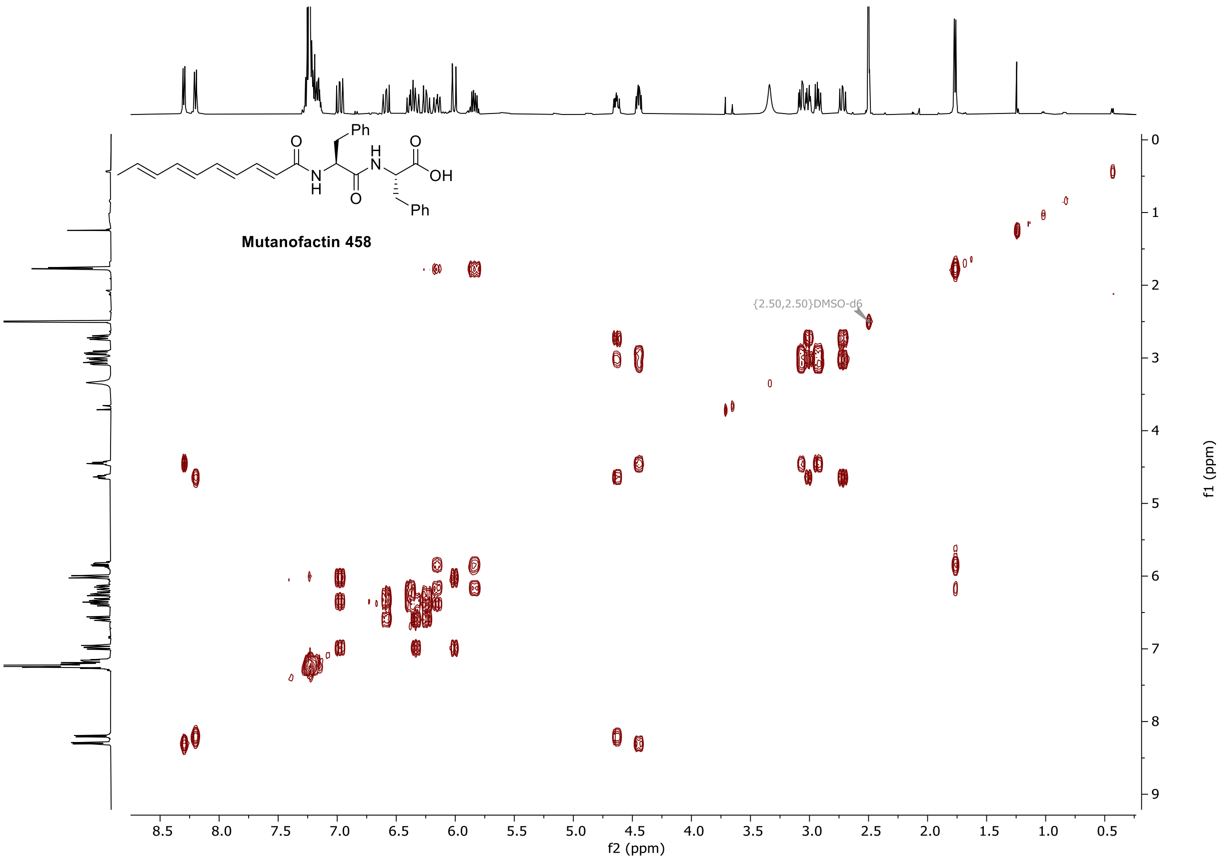

Supplement: Supplementary file 1 — oc4c02184_si_001.pdf [file oc4c02184_si_001.pdf]
